# Abstracts from the 13th International Conference on Cerebral Vascular Biology (CVB 2019)

**DOI:** 10.1186/s12987-019-0135-8

**Published:** 2019-06-20

## I1 13th International Conference on Cerebral Vascular Biology

### Michal Toborek

#### University of Miami School of Medicine, Miami, FL

##### **Correspondence:** Michal Toborek - mtoborek@med.miami.edu

*Fluids and Barriers of the CNS* 2019, **16(Suppl 1):**I1

The 13th International Conference on Cerebral Vascular Biology (CVB 2019; http://www.CVB2019.com) is being organized at the Marriott Miami Biscayne Bay hotel from June 25–28, 2019 in Miami, FL. The CVB conferences are bi-annual meetings that provide a forum for scientists from around the world to discuss their cutting edge research on cerebral vascular biology with a focus on CNS barriers, primarily the blood–brain barrier (BBB). CVB 2019 in Miami will be a continuation of a very successful conference series that was initiated in 1992. To emphasize the importance and international focus of the CVB series, the conferences rotate between North America, Europe, and Asia/Australia (1992, Duluth, MN; 1995, Paris, France; 1998, Salishan, OR; 2001, Cambridge, UK; 2003, Amarillo, TX; 2005, Muenster, Germany; 2007, Ottawa, Canada; 2009, Sendai, Japan; 2011, Leiden, The Netherlands; 2013, Montreal, Canada; 2015, Paris, France; 2017, Melbourne, Australia). Since the formation of the International Brain Barriers Society (IBBS) in 2006, CVB conferences are organized under the general auspices of the IBBS.

CVB 2019 is being attended by scientists from a broad range of backgrounds and disciplines who share a common interest in cerebral vascular biology. By bringing together scientists from diverse backgrounds in basic, translational, and clinical research, the meeting promotes the emergence of common themes across cerebrovascular topics. This will structure strategies for successful therapeutic interventions in the brain diseases that have strong cerebrovascular components and/or are underlined by the dysfunction of the BBB. The overall goal of CVB 2019 is to serve as a catalyst for exchange of information on the latest scientific discoveries related to the bioengineering of the BBB, efficient drug delivery into the brain, and involvement of the BBB in the physiology and pathology of the brain, including neuroinfections, neurodegenerative diseases, and addiction research. Consistent with this goal, the conference is focused on current and future research surrounding cerebral vascular biology, such as biology and structure of the neurovascular unit and cell junction proteins, constructing and modeling the BBB, delivery of various types of drugs across the BBB, the role of brain barriers in the pathology of neurological diseases, and therapeutic strategies to reverse these diseases by targeting the brain barriers. Emphasis are being placed on integrative science, translational aspects, and clinical research on disorders involving cerebral vasculature that can be applied to therapy. Other emerging topics discussed during the conference will involve impact of life style on modulation of brain barriers, cerebrovascular pathology of the aging brain, targeting cerebral vasculature for regenerative medicine, and the role of the gut-brain axis. Several activities will be dedicated to trainees, early stage investigators, and the inclusion of researchers from under-represented groups. The conference strongly promotes ethnic and gender diversity among the speakers and participants.

The major sponsors of CVB 2019 include the NIH (NINDS, NIA, and NHLBI) that supports, via the R13 grant mechanism, participation of trainees and early stage investigators, with the emphasis on individuals from under-represented groups. In addition, the NIMH sponsors the session on strategies of drug delivery into the brain in order to eradicate HIV reservoirs. The Platinum Sponsors of CVB 2019 are the University of Miami Clinical and Translational Science Institute (CTSI) and the Jerzy Kukuczka Academy of Physical Education in Poland. The Gold Sponsors are the Department of Biochemistry and Molecular Biology, the Miami Project to Cure Paralysis, the McKnight Brain Institute (all at the University of Miami), Florida International University (FIU), and Biogen. Several commercial companies, the Nagai Foundation Tokyo, the Johns Hopkins Malaria Research Institute, the Nebraska Center for Substance Abuse Research at the University of Nebraska Medical Center, and the Department of Surgery at the University of Miami are the Silver Sponsors. Finally, the IBBS, Fluids and Barriers of the CNS, and private donors are the Bronze Sponsors and provide poster and research awards to trainees.

## A1 A novel human immortalized cell-based blood–brain barrier triple co-culture model for predicting brain permeability of CNS drug candidates

### Keita Kitamura^1^, Kenta Umehara^1^, Ryo Ito^2^, Shota Suzuki^1^, Yoshiyuki Yamaura^2^, Takafumi Komori^3^, Naohiko Anzai^1^, Hidetaka Akita^1^, Tomomi Furihata^1^

#### ^1^Chiba University, Chiba, Japan; ^2^Ono Pharmaceutical Co., Ltd, Osaka, Japan; ^3^Eisai Co., Ltd., Tokoyo, Japan

##### **Correspondence:** Keita Kitamura - ahha4394@chiba-u.jp

*Fluids and Barriers of the CNS* 2019, **16(Suppl 1):**A1

**Objective:** In vitro human blood–brain barrier (BBB) models are expected to provide powerful tools for predicting in vivo human brain penetration of central nervous system (CNS) drug candidates, while it has not yet been fully established a practical model that possesses an optimal BBB phenotype and is readily scalable. To this end, we have developed and characterized a human BBB triple co-culture model comprising brain microvascular endothelial cells (BMEC), astrocyte and pericyte by taking advantage of immortalized cell line utility that allows various experimental approaches.

**Methods:** A human BBB model was constructed using immortalized human BMEC (HBMEC/ci18), astrocyte (HASTR/ci35), pericyte (HPBC/ci37). The gene expressions were determined by qPCR and immunocytochemistry. The BBB functions were examined by determining transendothelial electric resistance (TEER), lucifer yellow (LY) permeability and P-glycoprotein (P-gp) bi-directional transport. In the permeability assay, compounds were quantified by LC–MS/MS system.

**Results:** HBMEC/ci18 co-cultured with HASTR/ci35 and HBPC/ci37 showed elevated TEER (2.0-fold), reduced LY permeability (0.7-fold) and increased P-gp function (1.4-fold) compared with mono-culture. In support of these findings, expression levels of multiple BBB-specific genes, including barrier formation, transporters and receptors, were increased as well as their typical cellular localization in co-culture model. Notably, we performed BBB permeability assays using a set of 9 compounds with known CNS permeability characteristics. Expectedly, CNS-positive compounds (memantine, diphenhydramine, propranolol and pyrilamine) displayed high permeability coefficient values (Pe) (522 ± 100 to 1398 ± 324 × 10^−6^ cm/s). In contrast, CNS-negative compounds (quinidine, desloratadine, rhodamine 123, LY, sodium fluorescein) showed low Pe (21 ± 11 to 161 ± 31 × 10^−6^ cm/s).

**Conclusion:** We successfully developed a functional and scalable human BBB model. We hope that such research efforts are likely to open up new possibility of quantitative prediction of human CNS action based on in vitro experiment.

## A2 A novel reusable, versatile, microelectric organ-on-a-chip device to study blood–brain barrier functions

### Ana R. Santa-Maria, András Kincses, Fruzsina R. Walter, Sándor Valkai, András Dér, Maria A. Deli

#### Biological Research Centre of the Hungarian Academy of Sciences, Szeged, Hungary

##### **Correspondence:** Ana R. Santa-Maria - anaraquel.santamaria@brc.mta.hu

*Fluids and Barriers of the CNS* 2019, **16(Suppl 1):**A2

**Objective:** Biological barriers-on-a-chip models are cutting edge microengineered devices, but only a few combine the crucial parameters to study transport mechanisms, drug delivery and pathologies. Our laboratory developed a microelectric device (Walter et al. 2016), which enables visual observation, transendothelial electrical resistance (TEER) and permeability measurements on several biological barriers. The objective of our study was to further improve the device to make it more user-friendly and add novel functions.

**Methods:** The device was built up from a porous cell culture membrane sandwiched between two layers of PDMS and a top and bottom plastic slide coated with gold electrodes. After an automatic feeding period when the cells became confluent, a peristaltic pump was used to circulate the cell culture medium to mimic the blood flow. To verify the integrity, TEER was assessed with custom electrodes connected to an EVOM2 device. The endothelial surface charge was measured using silver electrodes connected to the outlets of the device. To validate our biochip we cultured the hCMEC/D3 human brain endothelial cell line and the stem cell derived CD34+ human endothelial cells in co-culture with bovine pericytes.

**Results:** We improved and optimized the biochip by (i) redesigning the shape of the electrodes, (ii) using universal luer-outlets, (iii) introducing small screws around the edges of the biochip we eliminated the use of the adhesive glue and (iv) could disassemble and reuse the device. The resistance was measured in real time using a custom made application compatible with cell phones. In the biochip under flow conditions the TEER elevated significantly in both BBB models which was also confirmed by ZO-1 and β-catenin immunostainings. A gene expression study was performed to investigate the differences between static and dynamic conditions on brain endothelial cells. Moreover a novel measurement of surface potential was also introduced.

**Conclusion:** This novel in vitro device for BBB culture models provides users with a standardized, reliable and reusable platform to perform pathology and pharmacology experiments. With advancement of the electrode layout, TEER automation and surface potential measurement our device is a cutting edge invention in the barrier-chip field.

**Grant Support:** This work was supported by the European Training Network H2020-MSCA-ITN-2015, grant number 675619, and NNE-129.

**Reference**

1. Walter FR, Valkai S, Kincses A, Petneházi A, Czeller T, Veszelka S, Ormos P, Deli MA, Dér A. A versatile lab-on-a-chip tool for modeling biological barriers. Sens Actuators B Chem. 2016; 222:1209–19.

## A3 Absolute computed tomography indicators of blood brain barrier permeability and cerebral microperfusion improve long term after carotid stenting in symptomatic patients

### Paweł J. Winklewski^1^, Mariusz Kaszubowski^2^, Grzegorz Halena^1^, Agnieszka Sabisz^1^, Kamil Chwojnicki^1^, Maciej Piskunowicz^1^, Marta A. Małkiewicz^1^, Edyta Szurowska^1^, Arkadiusz Szarmach^1^

#### ^1^Medical University of Gdansk, Gdansk, Poland; ^2^Gdansk Tecchnical University, Gdansk, Poland

##### **Correspondence:** Paweł J. Winklewski - pawel.winklewski@gumed.edu.pl

*Fluids and Barriers of the CNS* 2019, **16(Suppl 1):**A3

**Objectives:** Strong interdependence between severity of microcirculation impairment and time-based perfusion parameters has been recently reported. In particular, mean transit time (MTT) appears to show good association with cerebral small vessel disease (1, 2). Blood brain barrier (BBB) damage, in turn, is considered as a main initial pathogenic mechanism in cerebral small vessel disease (3, 4).

We tested the hypothesis that absolute computed tomography (CT) markers of BBB and cerebral microcirculation would improve 36 months after internal carotid artery stenting for symptomatic carotid stenosis while results obtained 6–8 weeks after stenting procedure would yield predictive value.

**Methods:** We recruited consecutive eligible patients with > 70% symptomatic single carotid stenosis with a complete circle of Willis and normal vertebral arteries to observational cohort study. We detected changes in cerebral blood flow (CBF), cerebral blood volume (CBV), MTT, time to peak (TTP) and permeability surface area-product (CT marker of BBB permeability, PS) before and after carotid stenting. We compared absolute differences in ipsilateral and contralateral CT perfusion markers before and after stenting. The search for regression models of “36 months after stenting” results were based on stepwise analysis with bidirectional elimination method.

**Results:** A total of 34 patients completed 36 months follow-up (15 females, mean age of 69.68 ± SD 7.61 years). At 36 months after stenting, absolute values for CT perfusion markers had improved: CBF (ipsilateral: + 7.76%, contralateral: + 0.95%); CBV (ipsilateral: + 5.13%, contralateral: + 3.00%); MTT (ipsilateral: − 12.90%; contralateral: − 5.63%); TTP (ipsilateral: − 2.10%, contralateral: − 4.73%) and PS (ipsilateral: − 35.21%, contralateral: − 35.45%). MTT and PS assessed 6–8 weeks after stenting predicted the MTT (ipsilateral: R2 = 0.867, contralateral R2 = 0.688) and PS (R2 = 0.314 for ipsilateral side and R2 = 0.394) value 36 months after stenting.

**Conclusions:** Improvements in BBB permeability and cerebral microcirculation persist for at least 3 years after carotid artery stenting in symptomatic patients. MTT and PS measured 6–8 weeks after stenting provides predictive value with respect to MTT and PS 36 months after stenting. Diminished PS values may suggest a decline in overall oxidative and inflammatory status.

**References**

1. Arba F, Mair G, Carpenter T, Sakka E, Sandercock PAG, Lindley RI, Inzitari D, Wardlaw JM; IST-3 Trial Collaborators. Cerebral White Matter Hypoperfusion Increases with Small-Vessel Disease Burden. Data From the Third International Stroke Trial. J Stroke Cerebrovasc Dis. 2017;26:1506–13.
2. Cao W, Yassi N, Sharma G, Yan B, Desmond PM, Davis SM, Campbell BC. Diagnosing acute lacunar infarction using CT perfusion. J Clin Neurosci. 2016;29:70–2.
3. Ding L, Hong Y, Peng B. Association between large artery atherosclerosis and cerebral microbleeds: a systematic review and meta-analysis. Stroke Vasc Neurol. 2017;2:7–14.
4. Szarmach A, Halena G, Kaszubowski M, Piskunowicz M, Studniarek M, Lass P, Szurowska E, Winklewski PJ. Carotid Artery Stenting and Blood–Brain Barrier Permeability in Subjects with Chronic Carotid Artery Stenosis. Int J Mol Sci. 2017;18:E1008.

## A4 Acetaminophen modulates blood–brain barrier permeability by altering tight junction protein expression in brain vasculature

### Junzhi Yang, Robert D. Betterton, Bianca G. Reilly, Jeffrey J. Lochhead, Thomas P. Davis, Patrick T. Ronaldson

#### University of Arizona, Tucson, AZ, USA

##### **Correspondence:** Junzhi Yang - jzyang345@email.arizona.edu

*Fluids and Barriers of the CNS* 2019, **16(Suppl 1):**A4

**Objective:** Our laboratory has extensively studied changes in critical blood–brain barrier (BBB) tight junction (TJ) proteins (i.e., claudin-5, occludin) in response to diseases. Specifically, we have shown that protein expression and/or trafficking of claudin-5 and occludin are modulated by pain, an effect that increases brain uptake of the opioid analgesic drug codeine via the paracellular route (i.e., “leak”). We have also reported that attenuation of TJ disruption in pain can decrease CNS codeine uptake, thereby reducing its analgesic effectiveness. Acetaminophen (APAP; *N*-acetyl-p-aminophenol; paracetamol) has been incorporated into many therapeutic products with opioids, or used in conjunction with opioids, in an effort to provide effective analgesia while reducing opioid dosages; however, it is unknown if APAP can alter BBB functional integrity. Therefore, our objective in this study is to investigate effects of APAP administration on TJ protein expression (i.e., claudin-5, occludin) and BBB “leak.” Such knowledge is critical to inform development of safer analgesic treatment regimens and to develop improved medications for treatment of acute and chronic non-cancer pain.

**Methods:** Female Sprague–Dawley rats (200–250 g) were administrated APAP (500 mg/kg, i.p.) or vehicle (100% DMSO, i.p.). After 3 h, animals were euthanized and brain microvessels were isolated. Protein expression of claudin-5 and occludin were determined by western blot analysis. Paracellular “leak” was measured using the established in situ brain perfusion technique with the established vascular tracer [14C]sucrose (0.5 p. Ci/ml).

**Results:** We show increased claudin-5 protein expression in brain microvessels following administration of APAP (3 h time point). We also observed that APAP had no effect on occludin expression at this same time point. In situ perfusion experiments showed increased brain uptake of [14C]sucrose, suggesting enhanced paracellular “leak” that correlates with altered TJ protein expression following APAP administration.

**Conclusions:** Taken together, our observations indicate that TJ dysregulation in response to APAP administration is a critical factor in controlling paracellular “leak” at the level of the brain microvasculature. Studies are ongoing in our laboratory to assess the impact of APAP-induced changes in BBB functional integrity of blood-to-brain delivery and analgesic effectiveness of opioids that are commonly co-administered with APAP (i.e., codeine, hydrocodone, oxycodone).

## A5 Acrylamide toxicity and cholinergic nervous system

### Marta Kopańska^1^, Joanna Czech^1^, Renata Muchacka^2^, Marta Batoryna^2^, Grzegorz Formicki^2^

#### ^1^University of Rzeszow, Rzeszow, Poland; ^2^Pedagogical University of Cracow, Kraków, Poland

##### **Correspondence:** Marta Kopańska - martakopanska@poczta.onet.pl

*Fluids and Barriers of the CNS* 2019, **16(Suppl 1):**A5

**Objective:** Acrylamide (ACR) is a chemical compound, that forms in starchy food products during cooking at high-temperatures, including frying, baking, and roasting. Despite many years of research, acrylamide an influence of acrylamide on the central-peripheral distal axonopathy remains poorly understood. Based on the accumulating evidence, it is possible that the disorder of elemental homeostasis represents an important component of the mechanism of ACR neurotoxicity. The mechanism of ACR neurotoxicity may be related to an impaired cholinergic transmission in the central and peripheral nervous system and a redox imbalance.

**Methods:** The research was conducted on Swiss male mice 12 weeks old segregated into 6 experimental and 3 control groups. Animals from experimental groups were injected intraperitoneally with ACR doses of 20 mg/kg body weight and 40 mg/kg (b.w.). All structures were taken 24, 48, and 192 h after the injection. Acrylamide’s influence on the acetylcholinesterase (AChE) activity was measured in right and left hemisphere, brain stem, cerebellum, hypothalamus, heart muscle, skeletal muscles of the thigh and smooth muscle of the small intestine in relation to the thiol groups and malondialdehyde (MDA) concentration. AChE activity was measured by the Ellman’s et al. colorimetric procedures. General protein concentration was determined using the Bradford method. MDA concentrations was measured by using TBARS method. The GSH was determined according to the Ellman colorimetric method.

**Results:** AChE activity was significantly lower (P < 0.001 to P < 0.05) in all structures. It was accompanied by the statistically significant (P < 0.001 to P < 0.05) increase in MDA concentrations in most of the studied structures time periods and ACR doses. -SH groups concentrations were significantly depleted in selected structures (P < 0.001 to P < 0.05).

**Conclusion:** Acrylamide has significant influence on redox balance in selected brain areas and other structures. The results of our study provide evidence for the occurrence of oxidative stress after intraperitoneal injection of ACR These observations suggest that intoxication with ACR may not only affect on‐going brain functions, by AChE inhibition, but may also participate in etiology of neurodegeneration.

## A6 Activation of the WNT/β-Catenin signaling in glioma stem cells impacts brain endothelial cell–cell interaction

### Amelie Vezina, Orieta Celiku, Mark Gilbert, Sadhana Jackson

#### National Institutes of Health, Bethesda, MD, USA

##### **Correspondence:** Amelie Vezina - amelie.vezina@nih.gov

*Fluids and Barriers of the CNS* 2019, **16(Suppl 1):**A6

**Background:** Glioma stem cells (GSC) are involved in glioblastoma (GBM) resistance to therapy and recurrence. GBM treatment is impacted by poor drug delivery across the selectively permeable blood–brain barrier (BBB). Moreover, increased WNT/β-catenin signaling in GBM is associated with resistance to therapy. It has also been shown that secreted WNT modulators regulate migration, invasion, and proliferation of GBM cells, as well as self-renewal of GSCs. Interestingly, activating mutations of β-catenin in the favorable WNT-subtype of medulloblastoma are associated with good prognosis and a highly permeable BBB. We observed high expression of WNT inhibitors in the favorable proneural subtype of GBM. Therefore, we propose that activation of WNT/β-catenin signaling in GSCs will have a paracrine effect on brain endothelial cell junctional integrity, to enhance BBB drug permeability.

**Methods:** We used the primary GSC line (GSC923) derived from classical GBM. In the GSC923, we activated the WNT/β-catenin signaling for 24 h with CHIR99021, a glycogen synthase kinase 3β inhibitor. Human brain microvascular endothelial cells (HBMEC) were then treated with GSC923 conditioned media (GSC-CM ± CHIR99021). Endothelial barrier function was evaluated by electrical cell impedance using the xCELLigence system. Data was reported by changes in cell index to reflect cell–cell interaction. BBB permeability markers (Claudin-5, VE-Cadherin, ZO-1, PLVAP) were evaluated by qPCR, immunoblotting and immunofluorescence.

**Results:** WNT/β-catenin signaling activation by CHIR99021 in GSC923 was validated by increased levels of β-catenin in the cytosol and nucleus, and increased secretion of downstream WNT inhibitors, DKK1 and sFRP1. Paracrine inhibition of WNT signaling in HBMEC was confirmed by decreased gene expression of transcription factor TCF4, compared to vehicle. HBMEC cell index was decreased by approximately 60% when treated with GSC923-CM + CHIR99021. Additionally, GSC923-CM + CHIR99021 decreased expression of HBMEC junctional proteins Claudin-5, ZO-1, and VE-Cadherin by 15%, 40%, and 50%, respectively, while increasing fenestration related protein (PLVAP) expression.

**Conclusion:** Our findings suggest disruption of brain endothelial junctional interactions occur in a paracrine manner under the WNT/β-catenin signaling axis from GSCs to endothelium. Targeting WNT/β-catenin signaling in GSCs to inhibit brain endothelial junctional interactions is of potential interest to increase drug delivery and responsiveness to improve GBM prognosis.

**Reference**

1. Pheonix, T.N. et al. (2016) Medulloblastoma genotype dictates blood brain barrier phenotype. Cancer Cell, 29(4):508–22.

## A7 Alteration of blood brain barrier (BBB) integrity in senescent cells and its association with senescent markers expression

### Carola Förster, Ellaine Salvador, Malgorzata Burek, Mario Löhr, Carsten Hagemann

#### University Hospital Würzburg, Würzburg, Ba, Germany

##### **Correspondence:** Carola Förster - foerster_c@ukw.de

*Fluids and Barriers of the CNS* 2019, **16(Suppl 1):**A7

**Objective:** The blood brain barrier (BBB) composed of endothelial cells maintain the homeostasis of the central nervous system by regulating the influx of compounds into the brain from the vascular circulation. That being the case, when the BBB is compromised, homeostatic breakdown may occur, leading to degenerative effects on BBB function.

Therefore, using an in vitro BBB model derived from murine cerebrum (cEND), this work aimed to investigate whether the expression of senescent markers is associated with BBB integrity and permeability decline.

**Methods:** Juvenile, juvenile induced to senescence and senescent cEND cells were compared for the expression of senescence as well as blood brain barrier integrity and permeability markers by immunofluorescence microscopy and Western Blot analysis. Cell proliferation was assessed by immunofluorescence staining of Ki67. Appraisal of DNA damage was performed by evaluating DNA double strand breaks as shown by the biomarker γ-H2AX. Transcripts associated with the senescence-associated secretory phenotype were evaluated using quantitative real time PCR (qRT-PCR). Juvenile cells were induced to senescence by treatment with H2O2.

**Results:** Senescent marker expression varied between juvenile and senescent cEND cells. Moreover, induction of senescence in juvenile cells led to expression of senescent markers as well as a decline in BBB integrity and permeability.

**Conclusion:** BBB integrity and permeability in senescent cEND cells is relative to senescent markers expressions. Due to that, establishment of the relationship between aging and central nervous system diseases could be drawn out.

**Grant Support:** HEALTH-2009-2.2.1-4 NEUROBID European Commission.

## A8 Alterations in the junctions between brain endothelial cells and pericytes during chronic sleep restriction

### Fernanda Medina-Flores^1^, Gabriela Hurtado-Alvarado^2^, Beatriz Gomez-Gonzalez^1^

#### ^1^Universidad Autónoma Metropolitana, Mexico city, Mexico; ^2^Instituto de Investigaciones Biomédicas, UNAM, Mexico

##### **Correspondence:** Fernanda Medina-Flores - fer_charlot@hotmail.com

*Fluids and Barriers of the CNS* 2019, **16(Suppl 1):**A8

**Objective:** As our previous reports indicate that pericytes may detach from the capillary wall in sleep restricted animals, we aimed to evaluate the changes in brain endothelial cell-pericyte interactions and its consequences on barrier function during sleep restriction.

**Methods:** Male Wistar rats were subjected to chronic sleep restriction using the multiple platform technique during 20 h with 4 h daily sleep opportunity. After 10 days of chronic sleep restriction animals were euthanized by decapitation and the brain was removed to isolate brain microvessels from the cerebral cortex and hippocampus. Those samples were used to evaluate the expression of claudin-5, occludin, connexin 43 and PDGFR by western blot. Another group of rats was used to perform permeability assays to Na-fluorescein (10 mg/mL). For this, the animals were euthanized by a lethal dose of ip. sodium pentobarbital, Na-fluorescein (0.2 mL/100 g body weight) was administered in the left ventricle, after 5 min circulation the subjects were perfused with 0.9% saline solution (5 min) and the brain was obtained. Absorbance of cerebral cortex and hippocampus supernatant were measured in a plate reader.

**Results:** In the cerebral cortex, sleep restriction reduced the expression of Connexin 43 in isolated blood-vessels as compared to intact controls; meanwhile, in the hippocampus there was a trend to reduction in connexin 43 expression with respect to the control group. Likely, sleep restriction reduced PDGFR expression in the isolated blood vessels of the cerebral cortex and hippocampus as compared to the controls sleeping ad libitum. Sleep loss decreased the expression of claudin-5 in the isolated blood vessels of the cerebral cortex but not of the hippocampus; while it decreased occludin expression in the isolated blood vessels of the hippocampus but not in the cerebral cortex as compared to the control group. Both regions presented an increase in blood–brain barrier permeability to Na-fluorescein.

**Conclusion:** Chronic sleep restriction decreases the interactions between endothelial cells and pericytes concomitantly decreasing tight junction protein expression in isolated blood vessels and increasing blood–brain barrier permeability to Na-fluorescein.

## A9 Altering HBMEC cell–cell junctions through various fluid shear regimes

### Dilshan Ranadewa

#### University of Central Florida, Orlando, FL, United States

##### **Correspondence:** Dilshan Ranadewa - dilshan@knights.ucf.edu

*Fluids and Barriers of the CNS* 2019, **16(Suppl 1):**A9

**Objective:** Introduction The blood brain barrier (bbb) is a specialized type of vasculature that runs throughout the brain and consists of pericytes and astrocytes, which surround an inner layer of human brain microvascular endothelial cells (HBMECs). HBMECs exist in arguably one of the most mechanically enriched environments in the body due to the fluid shear forces that are exerted onto them. It is well understood fluid shear stress influences HBMEC permeability, which in turn is dependent on endothelial cell–cell junctions. In fact, HMBEC cell–cell junctions (specifically tight junctions) have been demonstrated to also influence intercellular communication and cell–cell adhesion. However, the influence of various fluid shear-induced flow regimes on HMBEC cell–cell junction structural organization is currently unknown. These various fluid shear-induced regimes can be found in bifurcated regions of vasculature especially in areas of atherosclerotic plaque buildup Therefore, our objective was to investigate the influence of various fluid flow regimes on HBMEC cell–cell junction structure and organization.

**Methods:** Immortalized HBMECs were used at passage 25 and placed in a t25 cell culture flask for 48 h to reach a concentration of 2.50 × 105 cells/mL. Once confluent, cells were cultured in laminar flow chamber and incubated for 24 h. After this time, a peristaltic flow pump was used with a range flow rates that exerted a shear stress from 1 to 10 dyne/cm^2^. After the cessation of experiments HBMECs were fixed and stained for the tight junctions ZO-1 & Claudin-5 as well as adherens junctions JAM-A & VE-Cadherin.

**Results:** Our findings of a more intense staining to be observed under oscillatory fluid flow suggests that bbb structure is flow-regime dependent and we therefore believe these findings will be useful to the field of drug delivery and the bbb.

**Conclusion:** Cell–cell junctions are well known to be an integral part of BBB vascular regulation via the endothelium by allowing intercellular communication and regulation of permeability through adherens junctions, gap junctions, and tight junctions. Thought true for this study we focused on tight junctions as they have been suggested to be the most important in the regulation of bbb permeability.

## A10 Amyloid beta transfer via brain endothelial extracellular vesicles: the role of RAGE

### Ibolya E. Andras, Marta Garcia-Contreras, Christopher Janick, Paola Perez, Brice B. Sewell, Leonardo M. Durand, Michal Toborek

#### University of Miami, Miami, FL, USA

##### **Correspondence:** Ibolya E. Andras - IAndras@med.miami.edu

*Fluids and Barriers of the CNS* 2019, **16(Suppl 1):**A10

**Objective:** Elevated amyloid beta (Aβ) deposition was demonstrated in the brains of HIV-infected patients. The blood–brain barrier is believed to be critical for Aβ homeostasis and may contribute to Aβ accumulation in the brain. Our previous data have shown that HIV-1 increased extracellular vesicle (ECV) shedding from human brain microvascular endothelial cells carrying Aβ. This process may increase Aβ exposure of the nearby neural progenitor cells (NPC) and affect their differentiation. In this work we evaluated the role of RAGE in these events.

**Methods:** We exposed NPC to brain endothelial ECV carrying Aβ and evaluated the involvement of RAGE in ECV-Aβ transfer. We also investigated the impact of these factors on the inflammasome pathway and neuronal differentiation.

**Results:** Blocking RAGE with a high-affinity specific inhibitor (FPS-ZM1) resulted in significantly decreased ECV-Aβ transfer to NPC. Interestingly, ECV-Aβ was taken up by the NPC nuclei and partly colocalized with the inflammasome markers ASC and NLRP3. This colocalization was greatly affected by RAGE inhibition and the presence of HIV-1. To see whether this phenomenon will affect NPC fate, we further examined NPC differentiation into neurons. We found that both ECV-Aβ and RAGE inhibition altered neuronal differentiation.

**Conclusion:** Our data suggests that RAGE may modulate ECV-mediated amyloid pathology of the HIV-infected brain. Because NPC are critical in adult neurogenesis, our data may be relevant to the HIV-1 associated neurocognitive disorders.

**Grant Support:** MH098891, MH072567, DA039576, DA027569, and HL126559.

## A11 ApoA-I deficiency increases cortical amyloid deposition, cerebral amyloid angiopathy, cortical and hippocampal astrogliosis and astrocyte reactivity to amyloid in APP/PS1 mice

### Emily B. Button^1^, Guilaine Boyce^1^, Anna Wilkinson^1^, Sophie K. Stukas^1^, Jerome Robert^1^, Arooj Hayat^1^, Jianjia Fan1^1^, Kris Martens^2^, Cheryl L. Wellington^1^

#### ^1^University of British Columbia, Vancouver, BC, Canada; ^2^West Virginia University, Morgantown, WV, USA

##### **Correspondence:** Emily B. Button - ebbutton@mail.ubc.ca

*Fluids and Barriers of the CNS* 2019, **16(Suppl 1):**A11

**Background:** Alzheimer’s disease (AD) is defined by amyloid beta (Aβ) plaques and neurofibrillary tangles and characterized by neurodegeneration and memory loss. The majority of AD patients also have Aβ deposition in cerebral vessels known as cerebral amyloid angiopathy (CAA), microhemorrhages, and vascular co-morbidities, suggesting that cerebrovascular dysfunction contributes to AD etiology. Promoting cerebrovascular resilience may therefore be a promising therapeutic or preventative strategy for AD. Plasma high-density lipoproteins (HDL) have several vasoprotective functions and are associated with reduced AD risk in epidemiological studies and with reduced Aβ deposition and Aβ-induced inflammation in 3D engineered human cerebral vessels. In mice, deficiency of apoA-I, the primary protein component of HDL, leads to increased CAA and reduced cognitive dysfunction whereas overexpression of apoA-I from its native promoter in liver and intestine has the opposite effect and lessens neuroinflammation. Similarly, acute administration of reconstituted HDL reduces soluble Aβ pools in the brain and some have observed reductions in CAA as well. In this study we expand upon the known effects of plasma HDL in mouse models and in vitro 3D artery models to investigate the interaction of amyloid, astrocytes, and HDL on the cerebrovasculature in APP/PS1 mice.

**Methods:** APP/PS1 mice with one or two mutated apoa1 alleles were aged to 12 months. Plasma lipids, amyloid plaque deposition, Aβ protein levels, protein and mRNA markers of neuroinflammation, and astrogliosis were assessed using ELISA, qRT-PCR, and immunofluorescence.

**Results:** In APP/PS1 mice, apoA-I deficiency increased total and vascular Aβ deposition in the cortex but not the hippocampus. Neuroinflammatory markers including Il1b mRNA, ICAM-1 protein, PDGFRβ protein, GFAP protein and GFAP positive staining in both cortex and hippocampus were also elevated in apoA-I-deficient APP/PS1 mice. A striking observation was that astrocytes associated with cerebral vessels, in particular in vessels laden with Aβ, showed increased reactivity in APP/PS1 mice lacking apoA-I.

**Conclusion:** Circulating HDL can reduce amyloid pathology and astrocyte reactivity to parenchymal and vascular amyloid in APP/PS1 mice.

## A12 Assessing the physiological effects of low-intensity ultrasound on the blood–brain barrier

### Rucha Pandit, Jürgen Götz

#### Queensland Brain Institute, Brisbane, QL, Australia

##### **Correspondence:** Rucha Pandit - r.pandit@uq.edu.au

*Fluids and Barriers of the CNS* 2019, **16(Suppl 1):**A12

**Objective:** The use of low-intensity ultrasound (US) in conjunction with microbubbles is an emerging therapeutic strategy to transiently open the blood–brain barrier (BBB), delivering blood-borne factors into the brain parenchyma [1]. While the potential of US as a drug delivery system and a therapy by itself for neurodegenerative disorders is firmly established, the mechanism of its action on the components of the BBB have not been studied in detail. Here, we elucidated the effect of US on a range of proteins that form the BBB junction and further investigated paracellular and transcellular transport mechanisms.

**Methods:** We utilised human-derived induced pluripotent stem cells differentiated into endothelial cells as an in vitro model of the BBB to study the effects of US on cell morphology and the localisation of junction proteins. We further used C57BL6J mice for validation by dissecting microvessels and performing density gradient separation following US. To understand the effect of US on transport mechanisms, we used a mouse model deficient in the vesicle forming protein caveolin and different sized cargoes for comparison with wild-type mice following US.

**Results:** Following US, we observed a significant reduction in the tight junction proteins claudin-5 and occludin, together with a change in their localisation. We also revealed the importance of paracellular transport following US in transporting larger cargoes, contributing to the established fact of tight junction disruption and increased transcellular transport following US.

**Conclusion:** The therapeutic potential of low-intensity ultrasound is firmly established for neurodegenerative disorders, in addition to its utility for drug delivery [1]. Here, we elucidated the effects of US on the BBB physiology in an in vitro cell culture and in mouse models. We revealed a role of paracellular transport in transporting larger cargoes, in addition to transcytosis.

**Reference**

1. Leinenga G, Langton C, Nisbet R, Götz J. Ultrasound treatment of neurological diseases–current and emerging applications. Nat Rev Neurol. 2016; 12:161–74. 10.1038/nrneurol.2016.13.

## A13 Association between relative computed tomography indicators of cerebral microperfusion and clinical symptoms reported by patients undergoing internal coronary artery stenting

### Marta Anna Małkiewicz^1^, Arkadiusz Szarmach^1^, Agata Zdun-Ryżewska^1^, Grzegorz Halena^1^, Kamil Chwojnicki^1^, Adam Muc^2^, Piotr Łyźniak^1^, Maciej Piskunowicz^1^, Edyta Szurowska^1^, Paweł Winklewski^1^

#### ^1^Medical University of Gdansk, Gdansk, Poland; ^2^Gdynia Maritime University, Gdynia, Poland

##### **Correspondence:** Marta Anna Małkiewicz - marta.malkiewicz@gumed.edu.pl

*Fluids and Barriers of the CNS* 2019, **16(Suppl 1):**A13

**Objectives:** Carotid artery stenting and subsequent reperfusion diminish blood brain barrier (BBB) permeability and mean transit time (MTT), computed tomography (CT) markers of cerebral small vessel disease (1, 2, 3, 4). Headache, dizziness, tinnitus, blurred vision, transient blindness, a sense of gravity of the head and pain in the eyeballs are among the most commonly reported clinical symptoms in patients with internal carotid artery (ICA) stenosis. We hypothesised that an alleviation of neurological symptoms 36 months after ICA stenting may be interrelated with an improvement in BBB and MTT parameters.

**Methods:** Thirty-four subjects (15 females) with > 70% stenosis within a single internal carotid artery and neurological symptoms, who underwent a carotid artery stenting procedure, were studied. Differences in the following CT relative parameters (the ratio of appropriate values from ipsilateral side to contralateral side to stenosis) were compared before and 36 months after ICA stenting: cerebral blood flow (CBF), cerebral blood volume (CBV), mean transit time (MTT), time to peak (TTP) and permeability surface area-product (CT marker of BBB permeability, PS). A survey was conducted to assess the frequency of patients’ reported clinical symptoms.

**Results:** Relative CBF, CBV, MTT, TTP and PS values did not change 3 years after stenting, compared to the values measured before stenting. However, subgroup analysis distinguished three separate groups of patients: (1) patients with a rMTT reduction greater than 5%; (2) patients with a rMTT reduction between 2 and 5%; (3) patients with a rMTT reduction less than 2%. A prominent reduction in clinical symptoms was reported by patients with a relative MTT decline greater than 2%. The reported clinical symptoms of patients without an improvement in relative MTT (i.e. relative MTT decline less than 2%) either did not change or actually worsened.

**Conclusion:** Improvement in relative MTT parameters is related to a reduction in clinical symptoms, as reported by patients. Consequently, subjective clinical symptoms reported by patients suffering from ICA stenosis might be related to changes in cerebral microcirculation. Further studies are warranted to assess how alterations in brain microcirculation and BBB permeability are related to patient-reported quality of life and overall patient functional status.

**References**

1. Szarmach A, Halena G, Kaszubowski M, Piskunowicz M, Studniarek M, Lass P, Szurowska E, Winklewski PJ. Carotid artery stenting and blood–brain barrier permeability in subjects with chronic carotid artery stenosis. Int J Mol Sci. 2017;18:E1008.
2. Arba F, Mair G, Carpenter T, Sakka E, Sandercock PAG, Lindley RI, Inzitari D, Wardlaw JM; IST-3 Trial Collaborators. Cerebral white matter hypoperfusion increases with small-vessel disease burden. Data from the third international stroke trial. J Stroke Cerebrovasc Dis. 2017;26:1506–13.
3. Cao W, Yassi N, Sharma G, Yan B, Desmond PM, Davis SM, Campbell BC. Diagnosing acute lacunar infarction using CT perfusion. J Clin Neurosci. 2016;29:70–2.
4. Ding L, Hong Y, Peng B. Association between large artery atherosclerosis and cerebral microbleeds: a systematic review and meta-analysis. Stroke Vasc Neurol. 2017;2:7–14.

## A14 BACE1 and SCD1 convergence in the Neurovascular unit of dementia brains: understanding tau pathogenesis

### Gloria Patricia Cardona Gómez^1^, Mar Pacheco-Herrero^2^, Angelica Maria Sabogal-Guaqueta^2^, Javier Villamil-Orti^2^, Johana Gutierrez-Vargas^2^

#### ^1^University of Antioquia, Medellín, An, Colombia; ^2^Cellular and Molecular Neurobiology Area. Group of Neuroscience (GNA). SIU. School of Medicine, University of Antioquia, Medellín, Colombia

##### **Correspondence:** Gloria Patricia Cardona Gómez - patricia.cardonag@udea.edu.co

*Fluids and Barriers of the CNS* 2019, **16(Suppl 1):**A14

Proinflammatory cerebral environment is cause-effect of vascular commitment, which kinases/phosphatases dysregulation, alteration of tissue clearance and reduced plasticity. Our previous studies suggest an imbalance of the saturated and unsaturated fatty acid composition of phosphatidylethanolamine (PE) and phosphatidylcholine (PC) in animal models and human brains with cognitive impairment and dementia independently of the vascular arise (stroke, CADASIL and Alzheimer); involving pro-inflammatory markers and neurovascular unit impairment. In addition, b-secretase 1 (BACE1), an enzyme conventionally involved in the accumulation of amyloid, it is also key in the integrity of the blood–brain barrier. Previously, we found that BACE1 knockdown (BACE1-KD) reverted tauopathy1 and pro-inflammatory phospholipidomic profile in the hippocampus of old 3xTgAD mice, specially the lysophosphatidylethanolamine (LPE) 22:6/18:1 accompanied of cognitive improvement 2. In the present study we determine the convergence between BACE1, desaturases and their relationship with tauopathy in the brain parenchyma of different types of human dementia, and if there is an association with neurovascular unit components, as part of the triggering of hyperphosphorylation of tau (AT-8). Our current data shows a high correlation of LPE/PEs sublipidic species, particularly in LPE 22:5 in the cerebral cortex and PE 32:2 (16:1/16:1) in the white matter of CADASIL and sporadic Alzheimer disease compared to healthy brains. Also, in vitro BACE1-KD prevented the proinflammatory response and AT-8 in stearoyl-coenzyme A desaturase 1 (SCD1) and fatty acid desaturase 6 (FADS6) dependent-manner in an in vitro model by glutamate excitotoxicity. Interestingly, we found a convergence between BACE-1, SCD1 and AT-8, but not FADS6 with a neuronal pattern in the subiculum, and in astrocytes and vessels on the CA4 area from the hippocampus of Familial Alzheimer Disease (FAD) and Sporadic Alzheimer disease (SAD) and CADASIL compared to healthy controls, which were also positive for phosphorylated cytosolic phospholipase A2 (p-cPLA2). Therefore, protein association analysis and functional assays will be developed, to support their interdependency. Altogether our results suggest a more direct relationship between BACE-1 and hyperphosphorylated tau through monounsaturation imbalance on a proinflammatory environment in the subiculum, and involving vessels and astrocytes, as a transversal phenomenon of the neurovascular impairment in different types of dementia.

**Grant Support:** Colciencias Project # 111577757084, Asociacion Universitaria Iberoamericana de Postgrado Fellowship.

**References**

1. Piedrahita D et al. Frontiers in Aging Neuroscience. 2016; 9:498. 10.3389/fncel.2015.00498
2. Villamil-Ortiz J et al. Frontiers in Cellular Neuroscience. 2016; 10:260. 10.3389/fncel.2016.00260

## A15 Benefits of modulating cerebrovascular mechanisms through CCL11 signaling in a model of Parkinson’s disease

### Sanket Rege, S. Sakura Minami, Arnaud Teichert, Juliet Masumi, Steven P. Braithwaite

#### Alkahest, Inc, San Carlos, CA, USA

##### **Correspondence:** Sanket Rege - srege@alkahest.com

*Fluids and Barriers of the CNS* 2019, **16(Suppl 1):**A15

**Objective:** CCL11 (Eotaxin) is a chemokine whose levels in plasma increase with age and that has detrimental effects on brain functions. MPTP (1-methyl-4-phenyl-1,2,3,6-tetrahydropyridine) treatment is a well-established and characterized model of Parkinson’s Disease exhibiting loss of dopaminergic neurons and motor function. We aimed to test whether antagonizing CCR3, the primary receptor for CCL11, could improve motor function and neuroinflammation in MPTP mice, using the CCR3 selective antagonist AKST4290, to assess potential efficacy for Parkinson’s Disease.

**Methods:** 8-week old male C57Bl/6 J mice were given either saline or 20 mg/kg MPTP twice per day IP at 3-h intervals for two consecutive days. In addition, mice were dosed with vehicle or 30 mg/kg AKST4290 twice per day PO for 12 days. Motor function was assessed via kinematic gait analysis after 10 days of treatment. Peripheral immune cell infiltration was assessed in a separate experiment following 4 days of treatment with AKST4290.

**Results:** Treatment with AKST4290 for 10 days improved gait of MPTP treated animals. Reduction in neuroinflammation in the substantia nigra, with a decrease in activated microglia and astrocytes, was also observed with treatment. AKST4290 reduced the number of infiltrating CD3+ T-cells in the substantia nigra of MPTP treated mice after 4 days of treatment. These results indicate AKST4290 has an important effect on T-cell mediated inflammation in MPTP-treated mice.

**Conclusion:** CCL11 in the systemic circulation is identified as one of the key drivers of aging and is implicated in Parkinson’s disease. Targeting the CCL11/CCR3 pathway in an MPTP mouse model ameliorates neuroinflammation and peripheral infiltration of T cells, whose effects were associated with improvements in motor function. These results clearly suggest aging factors in the vasculature can effect potent changes in the brain through cerebrovascular mechanisms that have significant functional consequences.

**Grant Support:** Michael J. Fox Foundation.

**Reference**

1. Chandra G. et al. J Biol Chem. 2016;291(29):15267–81

## A16 Bioenergetic signature of brain endothelial cells

### Lester R. Drewes, Cade McDonald, Zachary Blankenheim

#### University of Minnesota Medical School Duluth, Duluth, MN, USA

##### **Correspondence:** Lester R. Drewes - ldrewes@d.umn.edu

*Fluids and Barriers of the CNS* 2019, **16(Suppl 1):**A16

**Objective:** Endothelial cells cover the surface of the brain vasculature from major arteries to arterioles, to the capillary bed, and finally to venules and the venous drainage. It is firmly documented that a network of inter-endothelial cell tight junctions restricts pericellular diffusion and that an endothelial specific expression program of influx and efflux transporters enables maintenance of homeostasis within the CNS by transcellular transport of nutrients and metabolites and export of waste products and circulating toxicants. However, brain endothelial cells are emerging as a very complex cell type that are endowed with unique physiological features including regional phenotypes, structures, receptors and signaling pathways. Dynamically induced alterations also occur with stress and disease states in functions, phenotypes, structures, signaling, and maintaining the neuro-microenvironment. The foundation for these endothelial cell responses to changing physiological and pathophysiological conditions is cellular metabolism and the generation of metabolic energy in the form of ATP. Very little is known about the underlying energy metabolism of brain endothelial cells regarding glycolysis versus mitochondrial respiration (ox-phos), regulatory mechanisms, alternative fuels, and the influence of hypoxia, amyloid-beta, inhibitors, and other conditions associated with disease. Although energy metabolism has been studied in various tissues and cells, little is known about the energy producing pathways of brain endothelial cells.

**Methods:** Therefore, using extracellular flux analysis, we characterized the bioenergetics of human brain microvascular endothelial cells (BMECs) in vitro.

**Results:** BMECs are primarily glycolytic and d-glucose, pyruvate, and glutamine are preferred metabolic substrates. Inhibitors of monocarboxylate transporter (MCT1), the mitochondrial pyruvate carrier, and glycolysis significantly alter ATP production, cellular respiration, and/or glycolytic rates.

**Conclusion:** These findings contribute to our understanding of endothelial cell metabolism, metabolic plasticity in normal and diseased brain as well as active angiogenesis during development or tumorigenesis. Understanding endothelial cell bioenergetics will be useful for future studies regarding development of therapies, that target endothelial cell energetics, for neurological disorders.

**Grant Support:** Whiteside Foundation and the Department of Defense.

## A17 Biology and pathology of the cerebral arteries in terms of neurosurgical treatment

### Kentaro Hayashi

#### Sasebo City General Hospital, Sasebo City, Nagasaki, Japan

##### **Correspondence:** Kentaro Hayashi - kenkunijp@yahoo.co.jp

*Fluids and Barriers of the CNS* 2019, **16(Suppl 1):**A17

We have performed thousands of neurological surgery and endovascular treatment for the cerebrovascular disease. In addition, We have investigated arterial disease and the blood brain barrier (BBB) using both in vivo and in vitro model. We found tendencies of region specific reaction in each portion of carotid and cerebral arteries. For instance, Common carotid bifurcation is a transition of elastic artery and muscular artery, and atherosclerotic plaque is formed there in elderly people. We are treating with carotid endarterectomy and carotid artery stenting for the carotid artery stenosis and evaluating the pathogenesis of the stenosis with histological studies as well as imaging studies. The cervical portion of the internal carotid artery (ICA) is so sensitive for the mechanical stress and that resulted in intra-procedural vasospasm or arterial dissection. The aneurysm arises from cavernous portion of the ICA. The aneurysm dose not rupture but growths as giant and compress surrounding cranial nerves. On the other hands intracranial aneurysms tend to rupture and result in subarachnoid hemorrhage. Cerebral aneurysms are treated with neck clipping or coil embolization. The terminal portion of the ICA is sensitive for the inflammation or irradiation, and tends to be steno-occlusive lesion. Secondary collateral vessels develop around the lesion so called moyamoya vessel and manifest unique moyamoya disease. By pass surgery is a option for the ischemic condition. Peripheral cerebral artery is affected with arteriosclerosis and resulted in cerebral infarction or intracerebral hemorrhage. The feature of the cerebral capillary is of course the BBB. The dysfunction of the BBB may be related to the chronic cerebral disease such as dementia. Carotid artery is not simple tube structure but has original function in each portion. Lasjaunias suggested those phenomenon as “segmental identity” in term of embryology. We preset clinical cases and discuss the mystery of cerebrovascular disease.

## A18 Blood brain barrier protection by miR-98 microRNAs during stroke

### Slava Rom, David Bernstein, Sachin Gajghate, Viviana Zuluaga-Ramirez, Nancy Reichenbach, Yuri Persidsky

#### Lewis Katz School of Medicine, Temple University, Philadelphia, PA, USA

##### **Correspondence:** Slava Rom - srom@temple.edu

*Fluids and Barriers of the CNS* 2019, **16(Suppl 1):**A18

**Objective:** Most neurological diseases, particularly including stroke, lead to blood brain barrier (BBB) dysfunction. A significant role in BBB injury belongs to inflammation, due to pro-inflammatory factors produced in the brain or leukocyte engagement of brain endothelium. Recently, microRNAs (miRNAs) have appeared as major regulators of gene expression in a variety inflammatory conditions in brain microvascular endothelial cells (BMVEC) that comprise the BBB. However, miRNAs’ role during cerebral ischemia/reperfusion is still underexplored.

**Methods:** In this study we utilized a mouse transient middle cerebral artery occlusion (tMCAO) in vivo model of stroke. In an in vitro stroke model, we used human primary BMVEC and oxygen–glucose deprivation (OGD) followed by reperfusion.

**Results:** We have recently identified a highly modified miRNA, known as miR-98, which is critically involved in regulating endothelial maintenance and in mediating inflammatory responses. Its presence has been postulated to promote recovery. Endothelial levels of miR-98 are significantly altered following ischemia/reperfusion insults. Overexpression of miR-98 reduced the infarct size after tMCAO. Further, miR-98 lessened proinflammatory Ly6CHI leukocyte infiltration into the brain following stroke and diminished the prevalence of M1 (activated) microglia within the impacted area. miR-98 attenuated BBB permeability, as demonstrated by changes to fluorescently-labeled dextran penetration in vivo and improved transendothelial electrical resistance (TEER) in vitro.

**Conclusion:** Finally, we show that post-stroke treatment with miR-98 may improve the integrity of tight junctions within the vasculature of the cortex. Our study provides identification and functional assessment of miRNAs in brain endothelium and lays the groundwork for improving therapeutic approaches for patients suffering from ischemic attacks.

**Grant Support:** The study was supported by R01NS101135 (SR).

## A19 Blood–brain barrier disruption during normal cognitive aging

### Inge C. M. Verheggen, Joost J. de Jong, Martin P. J. van Boxtel, Jacobus F. A. Jansen, Walter H. Backes, Frans R. J. Verhey

#### Maastricht University, Maastricht, The Netherlands

##### **Correspondence:** Inge C.M. Verheggen - inge.verheggen@maastrichtuniversity.nl

*Fluids and Barriers of the CNS* 2019, **16(Suppl 1):**A19

**Objective:** Recent advances in imaging techniques now make it possible to measure even subtle blood–brain barrier leakage, as occurs for instance during Alzheimer’s disease. Imaging studies indicate that these subtle leakages can already be found in the early stages of this disorder. It has been suggested that blood–brain barrier disruption could already be present in those who experience age-related cognitive decline, without having a neurological condition, and might be part of the underlying mechanism of cognitive aging. The current study uses a newly developed imaging technique to investigate the association between blood–brain barrier disruption and cognitive decline during normal aging.

**Methods:** 61 normal aging individuals (age between 46 and 91 years) were selected from the Maastricht Aging Study, in which participants were cognitively followed from 1993 to 2005. Blood–brain barrier leakage was assessed using dynamic contrast-enhanced MRI with a dual time resolution protocol. The association between leakage values and cognitive decline is examined using linear regression analysis.

**Results:** The computational analysis is currently ongoing and results will be available at the conference.

The hypothesis is tested whether people who suffer from age-related cognitive decline also show higher blood–brain barrier leakage values relative to people who age without significant cognitive setback. Blood–brain barrier leakage was previously found during early Alzheimer’s disease and in patients with mild cognitive impairment, and the results can now demonstrate whether this pathology is even present during normal cognitive aging, to reveal whether blood–brain barrier breakdown is a potential early event in the cascade leading to cognitive decline.

**Conclusion/interpretation:** A significant association would support the existence of a mechanism of blood–brain barrier disruption underlying cognitive aging. If blood–brain barrier disruption is indeed an early event in the pathological cascade, it would be a promising factor for the initiation of neurodegeneration independent of disease condition.

**Grant Support:** Personal talent fellowship of the Dutch Organization for Scientific Research, grant number NWO 406-15-031.

## A20 Blood–brain barrier permeability of [13C]sucrose in young adult and aged mice

### Ekram Ahmed Chowdhury^1^, Faleh Alqahtani^2^, Behnam Noorani^1^, Md Sanaullah Sajib^1^, Constantinos M. Mikelis^1^, Reza Mehvar^3^, Ulrich Bickel^2^

#### ^1^Texas Tech University Health Sciences Center, Amarillo, TX, USA; ^2^King Saud University, Riyadh 11451, Saudi Arabia; ^3^Chapman University, Orange, CA, USA

##### **Correspondence:** Ekram Ahmed Chowdhury - Ekram-Ahmed.Chowdhury@ttuhsc.edu

*Fluids and Barriers of the CNS* 2019, **16(Suppl 1):**A20

**Objective:** Blood–brain barrier (BBB) permeability is frequently measured in human studies and animal models because the integrity of the BBB is compromised in a range of neurological disorders. Whether the physiological aging process alone also impacts BBB tightness is still under discussion. We have recently introduced [13C]sucrose as a suitable hydrophilic low molecular weight marker for passive permeability. The objective of the present study was to compare BBB permeability of [13C]sucrose in young and aged mice.

**Methods:** The brain uptake of [13C]sucrose in young (2–3 months old) and aged (18 months old) female mice was measured after IV bolus administration of 10 mg/kg [13C12]sucrose via the tail vein. [13C6] sucrose was injected shortly before the terminal sampling time to serve as a vascular marker. Terminal blood and brain samples were collected between 5 min and 8 h in groups of 3–4 mice per time point. The analytes in brain and plasma samples were measured simultaneously using our established UPLC-MS/MS method [1]. Apparent brain uptake clearance, Kin, was then calculated by the single time point technique, the Patlak multiple time point graphical method, and a 3-compartment semiphysiologic pharmacokinetic model (central and peripheral compartment, plus one brain compartment).

**Results:** The brain uptake clearance values estimated by fitting the PK model (in units of µL min^−1^ g^−1^) in aged mice remained stable compared to young animals (0.092 vs. 0.090, respectively). When only ≤ 30 min terminal sampling points were used to ensure that the prerequisite of unidirectional brain uptake of sucrose holds true, the Kin values estimated by either single time point analysis (0.090 vs. 0.083, respectively) or graphical analysis (0.083 vs. 0.081, respectively) resulted in similar values.

**Conclusion:** We found no evidence for an age-related increase in sucrose permeability. Using relatively short terminal sampling time points, the unidirectional influx estimates obtained by single time point analysis or Patlak analysis match well the brain influx clearance estimated using a 3-compartment model.

**Grant Support:** Seed Grant from the Office of Sciences, TTUHSC School of Pharmacy.

**Reference**

1. Chowdhury EA, Alqahtani F, Bhattacharya R, Mehvar R, Bickel U. Simultaneous UPLC–MS/MS analysis of two stable isotope labeled versions of sucrose in mouse plasma and brain samples as markers of blood–brain barrier permeability and brain vascular space. J Chromatogr B. 2018;1073:19–26

## A21 Brain vascular heterogeneity: potential relevance of microvascular findings to clinical significance and differential pathologies

### Andres Villabona-Rueda, Carlos Pardo-Villamizar, Monique Stins

#### Johns Hopkins University, Baltimore, MD, USA

##### **Correspondence:** Andres Villabona-Rueda - avillab1@jhmi.edu

*Fluids and Barriers of the CNS* 2019, **16(Suppl 1):**A21

**Objective:** Neurovascular pathologies differ in specific brain regions. Abnormal patterns have been observed in particular brain areas in a variety of neurological disorders which are evident when using MRI and postmortem histological analysis. This is particularly evident in gray matter (GM) and white matter (WM) areas. These differing pathologies may be due to vascular heterogeneity, and conditional on where the vessels reside as well as their size. We aim to determine the structural and physiological differences of the blood brain barrier (BBB) vasculature in GM and WM brain regions. Here we focus on differences that can potentially affect the inflammatory response during infection and/or other neuroimmunological disorders.

**Methods:** Samples from five different brain regions were obtained from two non-pathological human brain specimens: WM, GM areas, deep white matter (DWM), basal ganglia (BG) and corpus callosum (CC). Brain microvessels were isolated by density gradient centrifugation followed by RNA extraction. To determine global gene expression differences, human gene expression microarrays were used (AGILENT Human FE 4 × 44 k V2 Microarray Kit). To identify differentially expressed genes one-way ANOVA and Gene-Ontology (GO) analysis was performed using Partek Genomic Suite v.7.0. Potential signaling pathways and upstream regulators related to differentially expressed genes were evaluated using Ingenuity Pathway analysis v.1.

**Results:** When comparing WM against GM samples 813 genes were significantly differentially expressed (p < 0.05). GO-enrichment analysis identified potential differences in transporter proteins (61 genes), junctional molecules (50 genes), cell adhesion molecules (43 genes), immune response proteins (54 genes) and hemostasis and coagulation molecules (7 genes). The top canonical pathway related to these genes was the regulation of actin-based motility mediated by Rho, including integrins, RHO, PIP5K, IRS P53, PAK, WASP and GSN. Networks involving molecular transport, lipid metabolism and cell death and survival were also found to be differentially enriched.

**Conclusion:** Preliminary data supports our hypothesis of a heterogenous BBB in WM versus GM areas, varying in structure and function along the microvascular vessel bed. Further analysis of the differential gene expression in particular brain vascular regions may help to understand the mechanisms and patterns of some diseases that affect differently the WM and GM.

**References**

1. Noumbissi ME, Galasso B, Stins MF. Brain vascular heterogeneity: Implications for disease pathogenesis and design of in vitro blood–brain barrier models. Fluids Barriers CNS. 2018;15(1):1–12. 10.1186/s12987-018-0097-2
2. Keaney J, Campbell M. The dynamic blood–brain barrier. FEBS J. 2015;282(21):4067–79. 10.1186/s12987-018-0097-2
3. Nyúl–Tóth Á, Suciu M, Molnár J, Fazakas C, Haskó J, Herman H, et al. Differences in the molecular structure of the blood–brain barrier in the cerebral cortex and white matter: an in silico, in vitro, and ex vivo study. Am J Physiol Hear Circ Physiol. 2016;310(11):H1702–14. http://ajpheart.physiology.org/lookup/doi/10.1152/ajpheart.00774.2015

## A22 Brain vasculature form an anticoagulant domain during vascular malformations

### Miguel Alejandro Lopez-Ramirez^1^, Angela Pham^1^, Romuald Girard^2^, Tine Wyseure^3^, Preston Hale^1^, Ignacio A. Romero^4^, Issam A. Awad^1^, Laurent O. Mosnier^3^, Mark H. Ginsberg^1^

#### ^1^University of California, San Diego, La Jolla, CA, USA; ^2^The University of Chicago, Chicago, IL, USA; ^3^Scripps Research Institute, San Diego, CA, USA; ^4^The Open University, Walton Hall, UK

##### **Correspondence:** Miguel Alejandro Lopez-Ramirez - malopezramirez@ucsd.edu

*Fluids and Barriers of the CNS* 2019, **16(Suppl 1):**A22

**Objective:** Cerebral cavernous malformations (CCMs) are common brain vascular malformations prone to acute and chronic hemorrhage with significant clinical sequelae. The pathogenesis of recurrent bleeding in CCM is incompletely understood. We sought to identify the molecular mechanism that contribute to bleeding in CCMs.

**Methods and results:** Using human CCM obtained at surgery we detected high protein and mRNA levels of two major anticoagulant endothelial receptors thrombomodulin (TM) and endothelial protein C receptor (EPCR) as assessed by immunostaining and by laser capture microdissection, respectively. We also observed that plasma levels of soluble TM were significantly increased in patients with CCMs compared with healthy subjects. In mice, endothelial-specific genetic inactivation of Krit1 (Krit1ECKO) or Pdcd10 (Pdcd10ECKO), which cause CCM formation, result in increased levels of vascular TM and EPCR and in enhanced generation of activated protein C (APC) on endothelial cells. Increased TM expression is due to upregulation of transcription factors KLF2 and KLF4 consequent to the loss of KRIT1 or PDCD10. Increased TM expression contributes to CCM hemorrhage, because genetic inactivation of one or two copies of the TM gene decreases brain hemorrhage in Pdcd10ECKO mice. Moreover, administration of blocking antibodies against TM and EPCR significantly reduced CCM hemorrhage in Pdcd10ECKO mice.

**Conclusions:** Our findings demonstrate that central nervous system (CNS) hemorrhage in CCMs is associated with locally elevated expression of the anticoagulant endothelial receptors TM and EPCR. Thus, a local increase in the endothelial co-factors that generate anticoagulant APC can contribute to bleeding in CCMs and plasma soluble TM may represent a biomarker for hemorrhagic risk in CCMs.

**Grant support:** This work was supported by National Heart, Lung, and Blood Institute grants K01 HL133530 (M.A.L.-R.), HL104165, HL130678, and HL142975 (L.O.M.), and HL078784 (M.H.G.), and National Institutes of Health grants NS092521 (M.H.G. and I.A.A.), as well as by the UCSD School of Medicine Microscopy Core (P30 NS047101).

**Grant Support:** NHLBI, K01 HL133530 (M.A.L.-R.), HL104165 HL104165, HL130678, and HL142975 (L.O.M.), and HL078784 (M.H.G.).

## A23 Cannabinoid receptor 2 agonists protected blood brain barrier, decreased neuroinflammation and altered immune responses in chronic HIV infection in a humanized mouse model and a model of encephalitis

### Yuri Persidsky

#### Lewis Katz School of Medicine at Temple University, Philadelphia, PA, USA

##### **Correspondence:** Yuri Persidsky - yuri.persidsky@tuhs.temple.edu

*Fluids and Barriers of the CNS* 2019, **16(Suppl 1):**A23

**Objectives:** Despite effective control of HIV replication by antiretroviral therapies, a significant number of patients develop HIV-associated neurologic disorders (HAND). HAND is attributed to chronic immune activation, ongoing neuroinflammation and blood brain barrier (BBB) compromise. We have recently shown that factors implicated in HAND pathogenesis (chronic neuroinflammation, secretion of pro-inflammatory factors and BBB impairment) could be mitigated by cannabinoid receptor 2 (CB2) stimulation 1, 2.

**Methods:** We have tested the effectiveness of three novel non-toxic and orally bioavailable CB2 agonists provided by Roche Pharmaceuticals, in chronic HIV infection using the ‘humanized’ NSG mouse model. Using non-forceful feeding as the method of daily administration 3, we studied effects on HIV infection up to 12 weeks. In parallel, we evaluated BBB protective effects of agonists in models of aseptic encephalitis and systemic inflammation.

**Results:** We demonstrate that HIV-infected humanized huNSG mice are a reliable and relevant model for longitudinal studies of HIV infection. CB2 agonists attenuated immune activation markers in the blood; however, none had an effect on HIV viral loads per se. The CB2 agonists diminished immune activation in the spleen and normalized the cytokine profile in the blood. CB2 agonists dampened microglial activation and improved expression of occludin that stabilizes the BBB. To further assess CB2 agonists on the BBB, utilizing non-forceful feeding in a model of aseptic encephalitis and in vivo microscopy, we demonstrated high efficacy of the new agonists to diminish leukocyte adhesion to and migration across the BBB. Agonists were able to offset BBB permeability in an in vivo model of systemic inflammatory response (LPS-induced). CB2 agonists down regulated expression of the adhesion molecules, VCAM-1 and ICAM-1, as well as attenuated de-regulation of a variety of genes involved in inflammation and endothelial injury responses in microvessels isolated from LPS-treated mice.

**Conclusion:** Our studies indicate that novel orally bioavailable CB2 agonists are BBB-protective and anti-inflammatory in a model of aseptic encephalitis and a systemic inflammation model. Further, they have potential in suppression of excessive immune activation in chronic HIV infection, both systemic and in the brain, and warrant further investigation as candidates to be included in a HIV treatment regimen.

**Grant Support:** AA023552, AA015913.

**References**

1. Ramirez SH, Hasko J, Skuba A, Fan S, Dykstra H, McCormick R, Reichenbach N, Krizbai I, Mahadevan A, Zhang M, Tuma R, Son YJ, Persidsky Y. Activation of cannabinoid receptor 2 attenuates leukocyte-endothelial cell interactions and blood–brain barrier dysfunction under inflammatory conditions. J Neurosci. 2012, 32:4004–16.
2. Ramirez SH, Reichenbach NL, Fan S, Rom S, Merkel SF, Wang X, Ho WZ, Persidsky Y. Attenuation of HIV-1 replication in macrophages by cannabinoid receptor 2 agonists. J Leukoc Biol. 2013, 93:801–10.
3. Rom S, Zuluaga-Ramirez V, Reichenbach NL, Erickson MA, Winfield M, Gajghate S, Christofidou-Solomidou M, Jordan-Sciutto KL, Persidsky Y. ecoisolariciresinol diglucoside is a blood–brain barrier protective and anti-inflammatory agent: implications for neuroinflammation. J Neuroinflammation. 2018, 15:25.

## A24 Cerebral angiogenesis in the brain endothelial Nemo deleted mouse as a small vessel disease model

### Yun Jiang

#### Institute for Experimental and Clinical Pharmacology and Toxicology, Luebeck, Germany

##### **Correspondence:** Yun Jiang - yun.jiang@pharma.uni-luebeck.de

*Fluids and Barriers of the CNS* 2019, **16(Suppl 1):**A24

**Objective:** Inactivating mutations of the NF-κB essential modulator (NEMO) cause incontinentia pigmenti (IP) which manifests with severe neurological symptoms in humans. Previous work showed that a specific Nemo knock-out in mouse brain endothelium (NemobeKO) leads to a disturbed blood–brain barrier (BBB) and microvascular pathologies, suggesting NemobeKO mice is a potential model for small vessel diseases. Recently, we observed angiogenesis in the NemobeKO mice. Herein, we would like to investigate the role of angiogenesis in the pathologies induced by Nemo deletion.

**Methods:** NemoFL;Slco1c1-CreERT2 mice were treated with tamoxifen to induce Nemo deletion (NemobeKO). EdU and pimonidazole-HCl were used for labeling of proliferation and hypoxia, respectively. The body weight and brain weight were measured. Microvascular pathologies were investigated by immunostainings. The behavioral changes were investigated by voluntary running wheels and open field test, whereas the cognitive function was evaluated in the object place recognition test.

**Results:** We observed robust angiogenesis in NemobeKO mice after injecting tamoxifen for 5 days. Angiogenesis peaked on day 15 after inducing the knockout, and was associated with apoptosis, increased vessel death and hypoxia. The proliferation gradually decreased, disappearing at later time points, followed by diminished hypoxia, ameliorated brain edema and other improved outcomes including lower mortality, recovered body weight, increased locomotion and decreased anxiety-like behaviors. The observed recovery suggests a therapeutic effect of angiogenesis in the microvascular pathologies.

The chronic oral tamoxifen treatment led to prolonged microvascular pathologies and to cognitive impairment, suggesting a potential model for vascular cognitive impairment. Persistent angiogenesis with more severe hypoxia was observed. No sign of recovery was found. This is presumably due to the fact that the maintenance of the Nemo deletion, which led to vascular pathology, impaired neovessel stability and interfered with the functional vasculature restoring, suggesting the importance of vascular stability in cerebral vessel remodeling as a self-rescue strategy.

**Conclusion:** The NemobeKO mouse is a novel model for the study of angiogenesis and vascular remodeling in the adult brain during the onset of small vessel diseases. Stimulating angiogenesis may offer an intriguing therapeutic approach for IP and related diseases.

## A25 Cerebrocortical proteome profile of female rats subjected to the western diet and forced physical activity

### Daniela Liśkiewicz^1^, Marta Nowacka-Chmielewska^2^, Arkadiusz Liśkiewicz^3^, Łukasz Marczak^4^, Anna Wojakowska^4^, Marta Przybyła^5^, Konstancja Jabłońska^5^, Jarosław J. Barski^5^, Andrzej Małecki^2^

#### ^1^The Jerzy Kukuczka Academy of Physical Education, Katowice, Polska; ^2^Laboratory of Molecular Biology, The Jerzy Kukuczka Academy of Physical Education, Katowice, Polska; ^3^Department of Physiology, Medical University of Silesia, Katowice, Poland; Laboratory of Molecular Biology, The Jerzy Kukuczka Academy of Physical Education; ^4^Institute of Bioorganic Chemistry, Polish Academy of Sciences, Poznan, Poland; ^5^Department of Experimental Medicine, Medical University of Silesia, Katowice, Poland

##### **Correspondence:** Daniela Liśkiewicz - d.liskiewicz@awf.katowice.pl

*Fluids and Barriers of the CNS* 2019, **16(Suppl 1):**A25

**Objective:** It is well known that energy-dense and composed of processed foods western diet, popular in developed countries, significantly increases the risk of obesity, type 2 diabetes, cardiovascular episodes, stroke, and cancer. But recently more attention has been paid to the contribution of an unhealthy diet to the development of the central nervous system (CNS). The positive effect of physical activity on CNS has been demonstrated in numerous clinical and experimental studies and growing evidence supports the role of physical activity as a brain and nervous system disease-preventing factor. The aim of our study was to verify the hypothesis that regular physical activity can mitigate the changes in cerebrocortical proteome induced by exposure to the western diet.

**Methods:** 9-weeks old female Long Evans rats (n = 18) alongside with standard rodent chow received snacks typical for human western diet (crackers, chips, cheese, sausage, candy bars) for 6 weeks. During this time seven animals were also subjected to forced physical activity (wheels with electric motor; 5 days a week, 1 h daily). Animals in the control group received standard rodent chow and did not have access to running wheels (n = 12). LC–MS/MS global proteomic profiling was performed in samples collected from temporal cortices of the studied animals. Functional annotation of identified proteins was analyzed by DAVID Bioinformatics Resources 6.8.

**Results:** Analysis using a one-way ANOVA revealed that the levels of 80 proteins significantly differed between groups. Approximately 55%, 52%, 24%, and 17% of this proteins were identified as occurring in the cytoplasm (GO:00057370, FDR = 0.00106), extracellular exosomes (GO:0070062, FDR = 4.01E−12), mitochondrion (GO:0005739, FDR = 1.34E−06) and cell membranes (GO0016020, FDR = 0.037076824) respectively. Alerted proteins were functionally annotated as factors involved in i.a. metabolic pathways, glutamatergic neurotransmission, Wnt signaling, oxidative phosphorylation, VEGF signaling pathway, long therm potentiation and vasopressin-regulated water reabsorption. Among proteins that differed between control and western diet fed animals three were upregulated and one was downregulated. Exposition to western diet and physical activity resulted in decreased level of 20 protiens and increase of 15 proteins.

**Conclusion:** Our results provide valuable contribution to the understanding of changes in brain structure and function induced by western diet and physical activity.

**Grant Support:** This research is supported by the National Science Center grant no. 2015/19/D/NZ7/02408.

## A26 Cerebrospinal fluid outflow resistance is increased following small-moderate ischaemic stroke

### Adjanie Patabendige, Nick MacKovski, Debbie Pepperall, Rebecca Hood, Neil Spratt

#### University of Newcastle, Callaghan, NS, Australia

##### **Correspondence:** Adjanie Patabendige - adjanie.patabendige@newcastle.edu.au

*Fluids and Barriers of the CNS* 2019, **16(Suppl 1):**A26

**Introduction:** We have previously demonstrated that intracranial pressure (ICP) is elevated ~ 24 h after small-moderate ischaemic strokes in rats. Oedema or cerebral blood volume increase was not the primary cause of this ICP rise, suggesting a role for cerebrospinal fluid (CSF) volume increase.

**Objective:** To determine whether resistance to CSF outflow is responsible for ICP elevation post ischaemic stroke in rats.

**Methods:** Outbred male Wistar rats (aged > 12 weeks) were subjected to photothrombotic stroke or sham procedure, and ICP was measured from 18 h post-stroke using a fibre optic pressure sensor probe (Opsens, Canada) inserted through a burr hole to access the epidural space. Another burr hole was made, and a catheter was inserted into the left lateral ventricle. Artificial CSF was then infused at rates that were increased step-wise. Resistance to CSF outflow (Rout) was determined by modifying the original constant rate infusion technique [Davson et al. 1970] to establish a continuous, low infusion rate (up to 30 μl/min) method.

**Results:** Photothrombotic stroke technique resulted in reproducible, small-moderate sized infarcts with well-defined boundaries. ICP was significantly higher (p = 0.0002) in rats subjected to stroke compared to sham animals (9.8 ± 1.1 mmHg (n = 11), and 3.3 ± 0.4 mmHg (n = 10), respectively) 18 h after intervention. CSF Rout was significantly increased (p = 0.0004) in rats subjected to stroke compared to the sham group (0.3 ± 0.04 mmHg/ul/min, and 0.54 ± 0.04 mmHg/ul/min, respectively). Values are mean ± SD.

**Conclusion:** CSF volume is very hard to measure accurately, but is dependant on CSF production and outflow. Our previous preliminary data showed that CSF production rates were not significantly different between stroke and control groups. The results from the current study strongly suggest that resistance to CSF outflow is increased in rats subjected to stroke compared to sham animals. These data support our hypothesis that ICP elevation post-stroke is most likely due to CSF volume increase caused by reduced CSF outflow and not because of increased CSF production.

**Grant Support:** Supported by a NSW Health EMC Fellowship to AP and the NHMRC (NS).

**Reference**

1. Davson H, Hollingsworth G, Segal MB. The mechanism of drainage of the cerebrospinal fluid. Brain. 1970; 93:665–78.

## A27 Changes of blood brain barrier (BBB) permeability by Tumor Treating Fields (TTFields) in vitro and in vivo

### Ellaine Salvador^1^, Almuth F. Kessler^1^, Malgorzata Burek^2^, Catherine Tempel Brami^3^, Tali Voloshin Sela^3^, Moshe Giladi^3^, Ralf-Ingo Ernestus^1^, Mario Löhr^1^, Carola Förster^2^, Carsten Hagemann^1^

#### ^1^University of Wuerzburg, Department of Neurosurgery, Wuerzburg, Ba, Germany; ^2^University of Wuerzburg, Department of Anaesthesia and Critical Care, Ba, Germany; ^3^Novocure Ltd., USA

##### **Correspondence:** Ellaine Salvador - Salvador_E@ukw.de

*Fluids and Barriers of the CNS* 2019, **16(Suppl 1):**A27

**Objective:** The blood brain barrier (BBB) tightly controls the influx of most compounds from blood to brain. Due to this regulation, delivery of drugs for treatment of brain tumors, may be obstructed. Tumor Treating Fields (TTFields), alternating electric fields with intermediate frequency and low intensity, have been established as a novel adjuvant therapy for GBM patients. Here, the influence of TTFields on BBB permeability is examined in vitro and in vivo.

**Method:** Immortalized murine brain capillary endothelial cells (cerebEND) that were grown on cover slips and transwell inserts were exposed to TTFields with a frequency of 100 300 kHz. For assessment of cell morphology immunofluorescent staining of the tight junction proteins Claudin 5 and ZO-1 was applied. For evaluation of BBB integrity and permeability transendothelial electrical resistance (TEER) and fluorescein isothiocyanate (FITC) staining, was utilized. For analysis of vessel permeability in vivo, rats were treated with 100 kHz TTFields for 72 h. At the end of treatment, rats were i.v. injected with Evan´s Blue (EB), which binds Albumin (~ 70 kDa) upon injection to the blood. After brain homogenization EB was extracted and quantified at 610 nm.

**Results:** Upon treatment with TTFields, tight junction proteins were delocalized from the cell membrane to the cytoplasm with maximal effects at 100 kHz. BBB integrity was significantly reduced by 65%. In line with these results, significantly increased BBB permeability for 4 kDa large molecules was observed. Cell morphology recovery was first detected at 48 h post-treatment and completely recovered to normal state after 96 h, pointing to a reversibility of the effect of TTFields on the BBB. In vivo, EB accumulation in the brain was significantly increased by application of TTFields to the rat head.

**Conclusion:** The alteration of BBB integrity and permeability induced by the application of TTFields supports an increased potential for delivering drugs to the brain, even those that are generally unable to cross the barrier. Therefore, TTFields could be utilized as an innovative approach of delivering drugs to treat malignant brain tumors and other related diseases of the central nervous system. These results should be further validated in clinical studies.

**Grant Support:** Novocure Ltd.

## A28 Characterization of the blood–brain barrier integrity and the brain transport of SN-38 in an experimental orthotopic xenograft rat model of diffuse intrinsic pontine glioma

### Salvatore Cisternino^1^, Catarina Chaves^1^, Xavier Declèves^1^, Meryam Taghi^1^, Marie-Claude Menet^1^, Joelle Lacombe^2^, Pascale Varlet^2^, Nagore G. Olaciregui^3^, Angel M. Carcaboso^3^

#### ^1^Université Paris Descartes, INSERM UMRS1144, PARIS, FRANCE; ^2^Université Paris Descartes, Department of Neuropathology, Hôpital Sainte-Anne, Paris, France; ^3^Institut de Recerca Sant Joan de Déu, Barcelona, Spain

##### **Correspondence:** Salvatore Cisternino - salvatore.cisternino@aphp.fr

*Fluids and Barriers of the CNS* 2019, **16(Suppl 1):**A28

**Objective:** Diffuse intrinsic pontine glioma (DIPG) constitutes the major cause of pediatric brain tumor mortality, with no effective treatment. SN-38, a camptothecin derivative and active metabolite of irinotecan, proved to be active in vitro against DIPG cells but not in vivo. The blood–brain barrier (BBB), preventing the tissue delivery of systemically administered drugs, may also represent further difficulty in the establishment of optimized chemotherapeutic strategies in DIPG.

**Methods:** We investigated BBB Integrity with Texas red-conjugated 3 kDa dextran (TRD) and [14C]-sucrose distribution, and the ABC-transporter functions involved in the [3H]-SN-38 BBB efflux in tumor-free and DIPG-bearing athymic RH-Foxn1rnu rats, and ABC-transporters knock-out mice.

**Results:** Capillary-confined TRD and unchanged [14C]-sucrose volume in cerebellum, brainstem and cerebrum regions infiltrated with tumor cells suggested an intact BBB. The expression of P-gp, BCRP and MRP1,4 evaluated by targeted proteomics with UHPLC-MS–MS in brain region homogenates remained unaltered in DIPG rats whereas DIPG cells express significant amount of BCRP. P-gp/Bcrp and/or MRPs inhibition increased [3H]-SN-38 BBB transport with quantitative differences according to the regions: in the cerebrum (8.3- and 3-fold, respectively), cerebellum (4.2- and 2.8-fold) and brainstem (2.6- and 2.2-fold).

**Conclusion:** The BBB integrity and ABC-transporters (e.g. P-gp, Bcrp, Mrps) function/expression have demonstrated to be conserved in DIPG and involved in SN-38 BBB efflux. Interestingly, elacridar, a dual P-gp/Bcrp inhibitor, improves the [3H]-SN-38 BBB transport independently of P-gp and Bcrp suggesting the role for an additive ABC BBB efflux. Altogether these results justify the strategy to inhibit/circumvent the ABC mediated efflux to improve anticancer drug therapy against DIPG.

## A29 Circulating extracellular vesicles from familiar and sporadic Alzheimer’s disease affects neurovascular components integrity

### Rafael Posada-Duque, Juan Villar-Vesga, Gloria Patricia Cardona-Gomez, Andrés Villegas

#### University of Antioquia, Medellín, 04, Colombia

##### **Correspondence:** Rafael Posada-Duque - rafael.posada@udea.edu.co

*Fluids and Barriers of the CNS* 2019, **16(Suppl 1):**A29

**Objective:** To determine the effect of circulatory extracellular vesicles (EVs) from familiar (FAD) and sporadic (SAD) Alzheimer’s disease patients on neurovascular components.

**Methods:** Venous whole blood samples of postmortem patients with FAD (n = 6), SAD (n = 4) and healthy controls (HC) (n = 6) were anti-coagulated with citrate and circulatory EVs were separated from platelet poor plasma by centrifugation 17,000×*g* for 60 min. Count and size distribution of vesicles was determined by flow cytometry. Brain microvasculature cell cultures were performed using bEnd.3 cell line and neuronal and astrocytes primary cultures were obtained from cortical brain dissections. Cell co-cultures (endothelium-astrocytes, neuron-astrocytes) were stimulated with EVs from SAD, FAD and HC in 1:1 ratio for 24 h. LDH cytotoxicity assay was performed and immunofluorescence of p120 catenin, GFAP, F-actin and Hoechst was used to determine gaps and adherens junctions (endothelium), branching and reactivity (astrocyte) and arborization and condensed nuclei (neuron) through epifluorescent microscopy.

**Results:** FAD patients showed a significant increase of circulatory EVs (major part of EVs increased were 0.5–1 um) and SAD-EVs shown an increase tendency of EVs compared to HC. FAD and SAD-EVs induced cytotoxicity on neuronal-astrocytes cultures; neuronal cultures showed a decreased confluency, less processes and increased condensed nuclei. On another hand, just SAD-EVs increased LDH release on endothelia and astrocytes. However, endothelium and astrocytes co-cultures showed cellular response to SAD and FAD-EVs; these was related with a gap formation, decrease in p120 catenin, actin stress fibers depolymerization and nuclei condensation on endothelium. Astrocytes showed an upregulation of GFAP, decreased in morphological branching and nuclei condensation in response to SAD and FAD-EVs.

**Conclusion:** Despite FAD patients have a strongest increased in circulatory EVs, SAD patients EVs showed higher cytotoxicity on all neurovascular components. Interestingly, both FAD and SAD-EVs induce endothelial gaps, neuronal death and astrocytic reactivity which is a common characteristic of sporadic and familiar AD pathology. Further studies involving EVs generation, cargo and phenotype could explain their action mechanism and generate possible target for pharmacological intervention.

**Grant Support:** COLCIENCIAS PROJECT 111577757128.

**Reference**

1. Posada-Duque, et al. Protection after stroke: cellular effectors of neurovascular unit integrity. Front Cell Neurosci. 2014.

## A30 Claudin-3 and claudin-12 deficient C57BL/6 mice display intact brain barriers

### Britta Engelhardt

#### University of Bern, Bern, BE, Switzerland

##### **Correspondence:** Britta Engelhardt - bengel@tki.unibe.ch

*Fluids and Barriers of the CNS* 2019, **16(Suppl 1):**A30

**Objective:** In addition to claudin-5, claudin-3 and claudin-12 have been suggested to be involved in regulating brain barriers integrity. Here we studied the impact of the absence of claudin-3 or claudin-12 on blood–brain and blood-cerebrospinal fluid barrier integrity in vitro and in vivo.

**Methods:** We established claudin-3−/− and claudin-12-lacZ knock in C57BL/6J mice to study the role of the respective claudins in brain barrier integrity in health and during neuroinflammation.

**Results:** In vitro and in vivo permeability studies demonstrate that claudin-3−/− and claudin-12-lacZ knock-in C57BL/6J mice display intact brain barriers. RNA sequencing and direct comparative qRT-PCR analysis of brain microvascular samples from WT and claudin-3−/− mice show beyond doubt that brain endothelial cells do not express claudin-3 mRNA. Detection of claudin-3 protein at the BBB in vivo and in vitro is rather due to junctional reactivity of anti-claudin-3 antibodies to an unknown antigen still detected in claudin-3−/− brain endothelium. Our study confirms expression and junctional localization of claudin-3 at the BCSFB of the choroid plexus. Making use of our claudin-12-lacZ knock-in mouse we detected lacZ activity in many tissues including the CNS. In the CNS, beta-galactosidase activity was not limited to blood vessels and very low when localized in vascular endothelial cells. Anti-claudin-12 antibodies failed to specifically detect claudin-12 in WT but not in claudin-12 KO tissues. Lack of either claudin-3 or claudin-12 did not affect brain barriers function during autoimmune neuroinflammation.

**Conclusion:** Our study clarifies that claudin-3 is not expressed at the BBB and suggests very low expression of claudin-12 in brain vascular endothelial cells. It also shows that absence of claudin-3 or claudin-12 does not impair brain barriers function during health and neuroinflammation in C57BL/6J mice.

**Grant Support:** European Union: 241861 -JUSTBRAIN, 607962 - nEUROinflammation), 675619 BtRAIN.

## A31 CNS delivery of intrathecally-administered antisense oligonucleotides and factors affecting distribution

### Brynna Wilken-Resman^1^, Michelle Pizzo^1^, Robert Thorne^2^

#### ^1^University of Wisconsin-Madison, Madison, WI, USA; ^2^Denali Therapeutics, University of Wisconsin-Madison, WI, USA

##### **Correspondence:** Brynna Wilken-Resman - bwilkenresma@wisc.edu

*Fluids and Barriers of the CNS* 2019, **16(Suppl 1):**A30

**Objective:** Antisense oligonucleotide (ASO) therapies for certain CNS indications have advanced in recent years, including FDA approval for an ASO therapy for spinal muscular atrophy and clinical trials for other neurological disorders. ASOs are ~ 7 kDa, single-stranded DNA oligomers and are attractive therapeutic candidates for diseases caused by known genetic abnormalities because they can interact with target RNA to modify protein production. ASOs are unable to cross the blood–brain barrier on their own, so they are administered centrally, typically achieved by intrathecal administration. Here, we investigated ASO transport and distribution in the CNS following intrathecal administration, focusing on the effects of different ASO chemistries and two transport mechanisms: diffusion in brain extracellular spaces and convection/dispersion in perivascular spaces surrounding the cerebral vasculature.

**Methods:** Three fluorescently-labeled ASOs differing in chemical modification and sequence were used for the following experiments. ASO1 was a fully phosphorothioated oligonucleotide. ASO2 was partially phosphorothioated and contained a 2′MOE modification. ASO3 had phosphorodiamidate morpholino modifications. Integrative optical imaging (Thorne et al. PNAS 2006, 2008) was used to measure ASO diffusion coefficients and hydrodynamic parameters. Intrathecal administration of ASOs was conducted in rats and the distribution was visualized using ex vivo fluorescence and confocal microscopy. Signal corresponding to perivascular ASO was quantified using a custom Fiji/ImageJ program.

**Results:** ASOs were found to have apparent hydrodynamic diameters in the range of 3–4 nm. Ex vivo fluorescence imaging after intrathecal infusion in rats revealed a substantially more limited diffusion gradient and perivascular distribution for ASO1 compared to ASO2. The difference in perivascular signal between the two ASOs was significant when quantified, and was particularly apparent in the dorsal cortex as well as in subcortical brain regions such as the striatum and hippocampus.

**Conclusion:** ASOs may experience additional sources of hindrance aside from molecular weight (i.e., secondary structure, binding, or electrostatic interactions) that could limit their distribution in the brain via diffusion and perivascular access. A more granular understanding of the mechanisms underlying ASO biodistribution in the CNS will ultimately yield new translational strategies to optimize their delivery.

## A32 Cognitive impairment seen in diabetes type 1 and 2 models paralleled blood brain barrier compromise and neuroinflammation and is reversed by poly(ADP-ribose) polymerase-1 inhibition

### Yuri Persidsky, V. Zuluaga-Ramirez, Alecia Seliga, S. Gajghate, M. Winfield, X. Jin, S. Rom

#### Lewis Katz School of Medicine at Temple University, Philadelphia, PA, USA

##### **Correspondence:** Yuri Persidsky - yuri.persidsky@tuhs.temple.edu

*Fluids and Barriers of the CNS* 2019, **16(Suppl 1):**A32

**Objective:** End organ injury in diabetes mellitus (DM) is driven by microvascular compromise (including diabetic retinopathy and nephropathy). Cognitive impairment is a well-known complication of DM types 1 and 2; however, its mechanisms are not known. We hypothesized that blood brain barrier (BBB) compromise plays a role in cognitive decline in DM.

**Methods:** In the 1st set of experiments, we used a DM type 1 model (streptozotocin injected C57BL/6 mice) and type 2 model (leptin knockout obese db/db mice), and assessed for cognitive performance, BBB permeability, gene profiling in brain microvessels, and neuroinflammation by immunohistochemistry 12 weeks after establishment of DM. In the 2nd set of experiments we treated DM type 1 and 2 animals with poly(ADP-ribose) polymerase-1 (PARP) inhibitor (olaparib) shown previously by us to be BBB protective and anti-inflammatory 1, 2. After behavior assessment at 8 weeks, PARP inhibitor was continuously administered by osmotic pump. At 12 weeks, animals were assessed for cognitive performance and other parameters as in the 1st set of experiments.

**Results:** in the 1st set of experiments, we showed enhanced BBB permeability and memory loss (Y-maze, water maze) that were associated with hyperglycemia. Gene profiling in brain microvessels of DM type 1 and type 2 animals demonstrated deregulated expression of more than fifty genes related to angiogenesis, inflammation, vasoconstriction/vasodilation, and platelet activation pathways by at least twofold (including eNOS, TNF, TGF1, VCAM-1, E-selectin, endothelin, several chemokines and MMP9). Brain tissues from DM type 1 and 2 mice showed microglial activation, upregulated ICAM-1 expression, down regulation tight junction (TJ) proteins and diminution of pericytes coverage as compared to controls. Treatment with PARP inhibitor resulted in complete reversal of cognitive deficits by week 12 (seen DM type 1 and 2 at 8 weeks) without any effect on hyperglycemia. We are analyzing BBB permeability, gene expression, and microglia reaction, expression of TJ and pericyte markers.

**Conclusions:** Our findings indicate BBB compromise in DM in vivo models and its association with memory deficits, gene alterations in brain endothelium and neuroinflammation. Prevention of BBB injury by PARP inhibition may be a new therapeutic approach to prevent cognitive demise in DM.

**Grant Support:** MH115786.

**References**

1. Rom S, Zuluaga-Ramirez V, Dykstra H, Reichenbach NL, Ramirez SH, Persidsky Y. Poly(ADP-ribose) polymerase-1 inhibition in brain endothelium protects the blood–brain barrier under physiologic and neuroinflammatory conditions. J Cereb Blood Flow Metab. 2015; 35:28–36.
2. Rom S, Zuluaga-Ramirez V, Reichenbach NL, Dykstra H, Gajghate S, Pacher P, Persidsky Y. PARP inhibition in leukocytes diminishes inflammation via effects on integrins/cytoskeleton and protects the blood–brain barrier. J Neuroinflam. 2016; 13:254.

## A33 Communication of cells of the neurovascular unit through inflammasome activation

### Istvan Krizbai, Mihaly Kozma, Adam Meszaros, Fanni Gyori, Attila E. Farkas, Imola Wilhelm, Adam Nyul-Toth

#### Biological Research Centre, Hungarian Academy of Sciences, Szeged, Hungary

##### **Correspondence:** Istvan Krizbai - krizbai.istvan@brc.mta.hu

*Fluids and Barriers of the CNS* 2019, **16(Suppl 1):**A33

**Objective:** Inflammation is a central element affecting cells of the neurovascular unit (NVU) in brain infections, neurodegeneration and aging. During this process, pathogen- or damage-associated molecular patterns are sensed by pattern recognition receptors, including Toll-like and NOD-like receptors (TLRs and NLRs). We have previously shown that cerebral endothelial cells and pericytes express these receptors which can be activated by danger signals. Activation of certain NLRs results in assembly of inflammasomes, which initiate caspase-mediated cleavage and maturation of inflammatory cytokines, like IL-1β. In the present study we aimed at understanding which infectious signals or endogenous alarmins induce canonical or non-canonical inflammasome activation in cells of the NVU and how the danger signal is transmitted from one cell type to the other.

**Methods:** Mono- and co-cultures of human cerebral endothelial cells, pericytes and astrocytes were used. Inflammasome priming and activation was assessed by qPCR, western-blot and ELISA. We have also used mouse models of LPS-induced infection and aging and advanced microscopy techniques (super-resolution).

**Results:** In brain endothelial cells exposed to bacterial signals or extracellular ATP, we observed canonical inflammasome activation. Through internalization of LPS or bacteria, the non-canonical pathway could also be activated. In parallel, the transendothelial electrical resistance decreased and the tight junctions became discontinuous, which was more pronounced in response to activation of the non-canonical pathway. Brain pericytes were able to secrete active IL-1β only as a result of non-canonical inflammasome activation, which was induced by internalization of LPS, intact bacteria or bacterial outer membrane vesicles. Inflammasome activation in brain pericytes resulted in the activation of inflammatory pathways in endothelial cells and impairment of the barrier functions. In vivo, we observed NLRP3 activation in the cerebral vessels of animals challenged with infectious mediators.

**Conclusion:** Cerebral endothelial cells—which are the first to sense blood-borne infectious agents—may initiate inflammasome activation in response to both extracellular and intracellular infection. On the other hand, pericytes—which are phagocytic cells—only activate this very potent and potentially dangerous inflammatory response in the brain after intracellular detection of pathogens. This signal is afterwards transmitted to brain endothelial cells as well.

**Grant Support:** NKFIH K-116158, GINOP-2.3.2-15-2016-00034, GINOP-2.3.2-15-2016-00020 and UEFISCDI PN-III-P4-ID-PCE-2016-0408.

**References**

1. Nyul-Toth et al. Brain Behav Immun. 2017. 10.1016/j.bbi.2017.04.010
2. Nagyoszi et al. J Neurochem. 2015. 10.1111/jnc.13197
3. Nagyoszi et al. Neurochem Int. 2010. 10.1016/j.neuint.2010.07.002

## A34 Connexin 43 isoform, 20-Cx43, induces brain endothelial barrier permeability

### Chelsea M. Phillips, Allison M. Johnson, Anna Hu, Richard F. Keep, Anuska V. Andjelkovic

#### University of Michigan, Ann Arbor, MI, USA

##### **Correspondence:** Chelsea M. Phillips - chelsphi@umich.edu

*Fluids and Barriers of the CNS* 2019, **16(Suppl 1):**A34

**Objective:** Cerebral cavernous malformation type III (CCM3) is associated with null mutations in programmed cell death 10 (PDCD10 or CCM3) and is characterized by vessel dilation and increased blood–brain barrier (BBB) permeability, leading to cerebral hemorrhage. Our lab demonstrated that the absence of CCM3 in a mouse brain microvascular endothelial cell (mBEC) line leads to increased connexin 43 (Cx43) expression, gap and tight junction (TJ) remodeling, and increased permeability. Absence of CCM3 also leads to increased expression of a 20 kDa Cx43 isoform (20-Cx43). Our study objectives include analyzing the role of 20-Cx43 in increased permeability and TJ complex reorganization in CCM3 knockdown (CCM3KD) mBECs and dissecting signaling pathways involved in 20-Cx43 expression.

**Methods:** The role of 20-Cx43 in brain endothelial barrier permeability was analyzed in CCM3KD mBECs, transfected with CCM3 siRNA, and mBECs overexpressing 20-Cx43. Morphological and functional alterations of CCM3KD and 20-Cx43-overexpressing mBECs were analyzed through immunoblotting and immunofluorescent staining, FRET analysis, and transendothelial electrical resistance (TEER) assays. To assess whether the 20-Cx43 is a product of cleavage or internal translation, CCM3KD mBECs were treated with Batimastat, an MMP inhibitor, or transfected with mitogen-activated protein kinase (MAPK)-interacting kinase (MNK) or mammalian target of rapamycin (mTOR) siRNA. Following inhibitor treatment or transfection, 20-Cx43 expression was determined through immunoblotting.

**Results:** 20-Cx43 overexpression causes increased brain endothelial permeability and TJ reorganization, as 20-Cx43-overexpressing mBECs had decreased Claudin-5 and zonula occludens-1 (ZO-1) expression, disrupted ZO-1 incorporation into TJ complexes, and decreased Claudin-5/ZO-1 interaction. Treatment with the MMP inhibitor did not decrease 20-Cx43 expression in CCM3KD cells, indicating that 20-Cx43 is not a cleavage product. 20-Cx43 expression, however, was altered when signaling pathways regulating internal translation were inhibited. While inhibiting mTOR signaling upregulates 20-Cx43 expression, MNK inhibition decreases 20-Cx43 expression, suggesting 20-Cx43 is an internal translation product.

**Conclusion:** Generated through internal translation, 20-Cx43 is directly involved in TJ complex reorganization and brain endothelial barrier permeability in CCM3KD mBECs.

## A35 Contrasting intranasal IgG delivery to the CNS in wild-type and transgenic mice

### Geetika Nehra^1^, Tongzhen Xie^1^, Maj S. Thomsen^2^, Khoua Vang^1^, Niyanta N. Kumar^1^, Michelle Pizzo^1^, Brynna Wilken-Resman^1^, Torben Moos^2^, Robert G. Thorne^3^

#### ^1^School of Pharmacy, University of Wisconsin-Madison, Madiscon, WI, USA; ^2^Laboratory of Neurobiology, Department of Health Science and Technology, Aalborg University, Aalborg, Denmark; ^3^School of Pharmacy, University of Wisconsin-Madison, Madison, WI, USA; 3.Denali Therapeutics, South San Francisco, CA USA

##### **Correspondence:** Geetika Nehra - nehra@wisc.edu

*Fluids and Barriers of the CNS* 2019, **16(Suppl 1):**A35

**Objective:** Intranasal route can non-invasively transport IgG antibodies to the central nervous system (CNS) via olfactory and trigeminal nerve pathways in the nasal mucosae (1,2), bypassing CNS barriers to access perivascular spaces around cerebral blood vessels (3). Here, we investigated the variations in intranasal (IN) antibody distribution in the CNS with time and effect on pathology which remain largely undiscussed. We further examined expression differences of neonatal Fc receptor (FcRn) on primary cell cultures and brain sections of rats, mice and transgenic murine models.

**Methods:** Radiolabeled IgG was intranasally applied to similar-aged wild-type C57BL6/J (WT) and transgenic APPswe/PS1dE9 (AD) mice over 30 min or 6 h. A separate group of AD mice were subjected to 14 weeks of IN exposure of a targeted antibody (6E10). Animals were euthanized at 30 min/6 h/15 weeks post first IN dose by saline perfusion. Whole brains from acute and semi-chronic IgG exposure were post-fixed and processed for gamma-counting/autoradiography/immunohistochemistry (IHC). Hemibrains from chronic IgG exposure were either post-fixed for sectioning and Thioflavin-S (ThioS) staining or processed for ELISA. Images were quantified using a custom Fiji/ImageJ program. Cell cultures underwent IHC and fluorescence imaging to validate anti-FcRn antibodies (1G3, M-255, H-4) prior to ex vivo IHC.

**Results:** Rapid IgG exposure was observed in WT and AD cortical regions, albeit higher for AD mice. IgG exposure spread to subcortical regions over 6 h. Chronic IN exposure of a targeted IgG led to a significant reduction in Thioflavin-S staining in the olfactory bulb and frontal cortex regions. No significant differences were observed in Aβ levels with ELISA. In the parenchyma, FcRn immunoreactivity was mostly neuronal and astrocytic in rats, non-neuronal, non-astrocytic in WT mice and perivascular in AD mice. In vitro FcRn immunolabeling was negative for rat and mouse endothelial cells, negative for mouse astrocytes and inconclusive for rat astrocytes.

**Conclusion:** IgG distribution in the murine CNS increases with time and potentially leads to reduction in pathology via target engagement and redistribution. IgG exposure can be variable across species and transgenic models, potentially due to the alteration of Fc receptors in the CNS

**Grant Support:** UW-Madison School of Pharmacy; ICTR; MJFF; WARF; Lundbeck Foundation; Fonden for Lægevidenskabens Fremme.

**References**

1. Thorne et al. Neuroscience. 2004
2. Lochhead & Thorne. Adv Drug Deliv Rev. 2012
3. Lochhead et al. J Cerebral Blood Flow Metab. 2015

## A36 Decreased migration of Mycobacterium-infected dendritic cells facilitates cellular aggregate formation and bacterial dissemination in an in vitro model of the blood brain barrier

### Fruzsina R. Walter^1^, Trey E. Gilpin^2^, Aisha Mergaert^2^, Melinda Herbath^2^, Maria A. Deli^1^, Matyas Sandor^2^, Zsuzsanna Fabry^2^

#### ^1^Biological Research Centre of the Hungarian Academy of Sciences, Szeged, Hungary; ^2^University of Wisconsin-Madison, Madison, WI, USA

##### **Correspondence:** Fruzsina R. Walter - fruzsinawalter@gmail.com

*Fluids and Barriers of the CNS* 2019, **16(Suppl 1):**A36

**Objective:** Mycobacterium tuberculosis (Mtb) can disseminate from the lung to the central nervous system (CNS) leading to tuberculous meningitis, which causes serious neurological damage with a high mortality rate. The mechanism of bacterial dissemination into the CNS is not known. Previously we showed that infected dendritic cells (DCs) mediate systemic dissemination and subsequent granuloma formation of Mycobacterium bovis bacillus Calmette-Guérin (BCG) and Mtb. The objective of this study was to test the role of mycobacterium-infected DC driven cell aggregation and bacterial dissemination across the BBB in vitro.

**Methods:** Primary bone marrow-derived DCs from CD11c-eYFP mice were used to study the migration capacity of mycobacterium-infected DCs in real time with fluorescent confocal microscopy. To investigate the dissemination of BCG and Mtb into the brain we developed a unique in vitro granuloma model combined with an in vitro double BBB co-culture model consisting of primary mouse brain endothelial cells and astroglia. In this model, BCG or Mtb-infected DCs were co-cultured with peripheral blood mononuclear cells (PBMCs) isolated from P25 Mycobacterium antigen-specific T-cell receptor transgenic mice. To reveal the mechanism of cell migration and cellular aggregate formation on brain endothelial cells iNOS, MMP9 and TNFα-KO mice were used.

**Results:** First we show that BCG infection decreases the migration capacity of DCs. Co-culture of mycobacterium-infected DCs and P25 transgenic mouse-derived PBMCs leads to cellular aggregate formation on brain endothelial cells which facilitate transmigration of infected cells across the BBB. Barrier properties of the BBB are impaired in the vicinity of cell clusters and endothelial ICAM-1 and VCAM-1 adhesion molecule expression is elevated. We revealed a double role of iNOS in the system: it causes BBB damage through reactive oxygen stress but by itself it slows down DC cell migration through the endothelial monolayer.

**Conclusion:** This novel and unique in vitro BBB-granuloma combined model suggests that mycobacterium-infected DC induced cell aggregation on the surface of brain vessels promotes Mtb dissemination into the CNS.

**Grant Support:** NIH grants 3R01NS076946; R01HL128778-01; HC128778-01 and T32GM081061.

**Reference**

1. Harding JS, Rayasam A, Schreiber HA, Fabry Z, Sandor M. Mycobacterium-infected dendritic cells disseminate granulomatous inflammation. Sci Rep. 2015; 5:15248.

## A37 Dendrimers-new hope for the treatment of Alzheimer’s disease

### Jerzy Leszek

#### Wroclaw Medical University, Wroclaw, PO, Poland

##### **Correspondence:** Jerzy Leszek - jerzy.leszek@umed.wroc.pl

*Fluids and Barriers of the CNS* 2019, **16(Suppl 1):**A37

**Objective:** The lack of effective treatment for Alzheimer’s disease (AD) stems mainly from the incomplete understanding of AD causes. Currently there are several hypothesis which try to explain the early molecular mechanisms of AD pathogenesis.

**Methods:** Considering that AD is a multi-factorial disease with several pathogenic mechanisms and pathways, a multifunctional nanotechnology approach may be needed to target its main molecular culprits. To very early diagnosis of AD we need to have an affordable, ultrasensitive and selective molecular detection methods. Ultra-low concentrations of protein biomarkers (e.g. ADDL-amyloid-Beta-derived diffusible ligands) which have been implicated in the pathogenesis of AD, is possible to detect, owing to carrier dendrimers -polymeric molecules chemically synthesized with well-defined shape size and nanoscopic physicochemical properties reminiscent of proteins

**Results:** Our studies have revealed that dendrimers have ability to prevent aggregation and fibrillation of proteins involved in AD. Some of dendrimers were demonstrated to cross blood–brain barrier, which legitimized research research on these compounds as potential drugs for neurological disorders like AD. Recent our studies have revealed that dendrimers possess the intrinsic ability to localize in cells associated with neuroinflammation (activated microglia and astrocytes) and thus can be used in neuroinflammation therapy

**Conclusion:** Above/mentioned findings may be significance in the context of potential application of dendrimers as drug carriers or active compounds per se. According to opinion the authors of this presentation, they are promising macromolecules for further investigations on their applicable in neurodegenerative disorders, for instance AD

**Grant Support:** Nr ST.C230.16.087.

**Reference**

1. Aliev G et al. Alzheimers disease-future therapy based on dendrimers. Curr Neuropharmacol. 2018. 10.2174/1570159x16666180918116462

## A38 Design of experiments: an efficient method to guide sub-culture and maturation of human stem cell-derived brain microvascular endothelial cells

### Taylor Gray, Mercedes Beyna, Susanne Swalley, Robin Kleiman, Birgit Obermeier

#### Biogen, Cambridge, MA, USA

##### **Correspondence:** Taylor Gray - taylor.gray@biogen.com

*Fluids and Barriers of the CNS* 2019, **16(Suppl 1):**A38

**Objective:** To optimize post-differentiation freeze, thaw, and culture conditions for induced pluripotent stem cell-derived brain microvascular endothelial cell (iPSC-BMEC) maturation, functionality, and reproducibility.

**Methods:** Human iPSCs were spontaneously differentiated into BMECs (protocol based on Lippmann et al. Nat Biotech 2012). Dissociation and sub-culture conditions were investigated using Design of Experiments (DoE), an unbiased method based on structured statistical analysis of variance (ANOVA). DoE efficiently identifies interactions amongst experimental variables and predicts responses. Our design focused on maximizing trans-endothelial electrical resistance (TEER). We incorporated a Mantis liquid handler to execute precise culture conditions in 96-well transwell systems and measured TEER over time. Top hit conditions were validated on multiple batches of iPSC-BMECs, these included post-thaw cell viability, hourly TEER reads, and immunocytochemistry (ICC).

**Results:** We have identified a robust cryopreservation method, time course and medium formulation for sub-culturing spontaneously differentiated iPSC-BMEC. The optimized cryopreservation protocol yields high cell viability recovery allowing the ability to produce bulk batches of iPSC-BMECs, thereby minimizing interexperimental variability. Surprisingly, we found that the removal of retinoic acid and the extension to at least 7 days in culture reproducibly resulted in prolonged high TEER, approximately 500 ohms * cm^2^ greater than TEER peak at 48 h, in iPSC-BMEC mono-culture transwells. Our sub-culture method generates iPSC-BMECs that express the endothelial surface marker PECAM1, relevant blood–brain barrier (BBB) tight junction proteins (claudin-5, ZO-1, occludin), and transporters enriched on brain microvessels (Glut-1, transferrin receptor, insulin receptor).

**Conclusion:** We demonstrate the power of applying DoE to fine-tune complex culture conditions to maximize cell performance. Our cryopreservation and sub-culture protocols robustly produce functional endothelial cells of the BBB that are suited to address basic cerebral vascular biology questions, study the vascular phenotype of neurological disorders, and enable high-throughput screening for drug discovery.

## A39 Developing a human in vitro blood–brain barrier model for predictive safety

### Sheetal Kumar, Alexander Brown, Dan Adams, Jing Yuan

#### Amgen, Cambridge, MA, USA

##### **Correspondence:** Sheetal Kumar - skumar36@amgen.com

*Fluids and Barriers of the CNS* 2019, **16(Suppl 1):**A39

The blood brain barrier (BBB) protects the brain from toxic agents in the blood(Abbott and Friedman 2012). From a drug safety perspective, disruption to the BBB integrity or functionality by a therapeutic agent has been shown to be associated with the risks of neuroinflammation and neurodegeneration. For example, the administration of the CAR-T therapy has been shown to potentially increase BBB permeability, a risk factor for neurotoxicity (Gust, Hay et al. 2017). Therefore, it is important to have a functional in vitro human BBB model to understand and assess the effects of a drug candidate which potentially trigger immune cell infiltration and direct interactions of the endothelial cells in the BBB.

We have developed a human in vitro BBB model from the differentiation of human pluripotent stem cells (hiPSCs). When compared with the primary human brain endothelial cells, the human iPSC derived BBB model showed robust structural and functional characteristics. The in vitro BBB model exhibited barrier integrity through sustained trans endothelial electrical resistance (TEER) greater than 1500 Ohms.cm2, closely reflecting the in vivo BBB.

We are further investigating the effects of normoxia and hypoxia conditions, on the functionality and integrity of the BBB. Effects of inflammatory cytokines on BBB equilibrium is also being tested. The development of the human in vitro BBB model will provide a valuable tool to study drug related disruption of BBB and help in selecting compounds that are safe to BBB function.

**References**

1. Abbott NJ, Friedman A. Overview and introduction: the blood–brain barrier in health and disease. Epilepsia. 2012; 53(Suppl 6):1–6.
2. Gust J, et al. Endothelial activation and blood–brain barrier disruption in neurotoxicity after adoptive immunotherapy with CD19 CAR-T cells. Cancer Discov. 2017; 7(12): 1404–19.

## A40 Development of a human immortalized cell-based multicellular spheroidal blood–brain barrier model: a promising platform for evaluation of permeability of various drugs

### Kenta Umehara^1^, Saki Izumi^2^, Takafumi Komori^2^, Ryo Ito^2^, Kenichi Nunoya^2^, Yoshiyuki Yamaura^2^, Haruo Imawaka^2^, Naohiko Anzai^3^, Hidetaka Akita^4^, Tomomi Furihata^3^

#### ^1^Chiba University, Chiba, NA, Japan; ^2^Dept. Drug Metab. & Pharmacokinet., Eisai Co. Ltd, Japan; ^3^Dept. Pharmacol., Grad. Sch. Med., Chiba Univ, Japan; ^4^Lab. DDS Design & Drug Dispos., Grad. Sch. Pharm. Sci., Chiba Univ, Japan

##### **Correspondence:** Kenta Umehara - dive_k_um_sea@hotmail.com

*Fluids and Barriers of the CNS* 2019, **16(Suppl 1):**A40

**Objective:** The blood–brain barrier (BBB), which is formed by brain microvascular endothelial cells (BMECs) together with brain pericytes and astrocytes, strictly controls drug traffic between the blood and brain. For evaluation of drug permeability across the BBB in humans, in vitro BBB model is a useful experimental tool. A multicellular spheroidal BBB (MCS-BBB) model is expected as a highly functional in vitro BBB model. To develop such MCS-BBB model, immortalized cells are useful in terms of their scalability and functionality, and thus we have recently established human conditionally immortalized BMECs (HBMEC/ci18), brain pericytes (HBPC/ci37) [1], and astrocytes (HASTR/ci35) [2]. In this study, we aimed to develop a human immortalized cell-based MCS-BBB (hiMCS-BBB) model and characterize their BBB properties.

**Methods:** hiMCS-BBB models were prepared using the three immortalized cells by tri-co-culturing in 96-well V-bottom plates. For a comparison, hiMCS-BBB without HBMEC/ci18 models (hiMCS-BBB [w/o BMEC]) were also prepared. Cell localization was analyzed by a fluorescence microscopy. The protein expression was examined by immunocytochemistry. The BBB functions were examined by determining FITC-dextran, Rhodamine 123 (R123), and Angiopep-2 permeabilities.

**Results:** When seeded in 96-well V-bottom plates, the three cell types self-assembled into a spheroid, where HBMEC/ci18 cells formed the outer monolayer. While HASTR/ci35 cells accumulated in the core, the majority of HBPC/ci37 cells aligned along the inner side of HBMEC/ci18 cells. In examination of the BBB properties of the hiMCS-BBB, the results showed that HBMEC/ci18 cells clearly expressed the tight junction proteins (e.g., claudin-5 and zonula occludens-1) and efflux transporter proteins (P-glycoprotein and breast cancer resistance protein) at the plasma membrane. The hiMCS-BBB model showed significantly lower FITC-dextran and R123 permeabilities compared with those obtained in the hiMCS-BBB [w/o BMEC] model (0.3 ± 0.1 and 0.2 ± 0.1-fold, respectively). Furthermore, Angiopep-2 highly permeated into the hiMCS-BBB compared with the scramble peptide (4.4 ± 1.0-fold). These results suggest that HBMEC/ci18 cells form functional barriers in the hiMCS-BBB model.

**Conclusion:** We have developed the first hiMCS-BBB model which exhibits functional BBB properties. Therefore, the hiMCS-BBB model will be expected to provide a useful platform for evaluation of permeability of various drugs, including therapeutic peptides.

**References**

1. Umehara K, et al. Mol Neurobiol. 2018;55(7):5993–6006.
2. Kitamura K, et al. J Pharmacol Sci. 2018;137(4): 350–8

## A41 Development of a novel in vitro model of Alzheimer’s disease-like blood–brain barrier

### Sandrine Bourdoulous^1^, Haniaa Bouzinba-Ségard^1^, Francesca Re^2^, Wiep Scheper^3^, Claudia Guimas Almeida^4^, Thomas G Ohm^5^, Pierre-Oliver Couraud^1^

#### ^1^Institut Cochin, Inserm/CNRS/Université Paris Descartes, Paris, France; ^2^School of Medicine and Surgery, University of Milano-Bicocca, Italy; ^3^Amsterdam University Medical Centers, Amsterdam, The Netherlands; ^4^CEDOC, Chronic Diseases Research Center, NOVA Medical School|Faculdade de Ciências Médicas; ^5^Charite Universitätsmedizin Berlin, Germany

##### **Correspondence:** Sandrine Bourdoulous - sandrine.bourdoulous@inserm.fr

*Fluids and Barriers of the CNS* 2019, **16(Suppl 1):**A41

The process of discovering and developing drugs for neurological disorders, like Alzheimer’s disease (AD), is extremely challenging because of the presence of the blood–brain barrier (BBB), which prevents the access of over 98% of potential therapeutics from passing into the brain. In preclinical AD, changes in vascular biomarkers occur before the development of cognitive impairment and persist as the disease progresses. At late-onset, there is BBB dysfunction and diminished brain perfusion and effusion that, in turn, lead to neuronal injury and amyloid-beta (Aβ) accumulation in the brain, contributing to cognitive decline. Among observed alterations at the BBB, expression of the low-density lipoprotein receptor-related protein 1 (LRP1), which mediates the efflux of Aβ from the brain to the periphery, is decreased, whereas expression of the receptor for advanced glycation end products (RAGE), implicated in Aβ influx back into the central nervous system is increased, adding to the amyloid burden in the brain.

Our aim is to develop an in vitro model of human BBB that reflects the barrier alterations observed in AD and, in particular, alterations in the expression of the Aβ transporters.

We took advantage of the human brain endothelial cell line hCMEC/D3, that phenocopies the normal human BBB, in terms of low paracellular and transcellular permeability, polarized secretion and transport, architectural organization, and protein expression. We engineered hCMEC/D3 over-expressing RAGE or with down-regulated expression of LRP-1 and analyzed the impacts on functional properties of the BBB. Interestingly, we observed that RAGE overexpression increased the tightness of the hCMEC/D3 monolayers: it increased the organisation of adherens and tight junction components at cell/cell junctions and reduced monolayer permeability to diffusion markers.

These results show the interest of establishing an in vitro system modelling the feature of the AD-like human BBB to accelerate the development of novel drugs for efficient treatment of AD and potentially of other CNS diseases.

**Grant Support:** JPND: Joint Programme - Neurodegenerative Disease Research NAB3.

## A42 Dietary salt promotes cognitive impairment through endothelial dysfunction and tau phosphorylation

### Giuseppe Faraco^1^, Karin Hochrainer^1^, Steven G Segarra^1^, Samantha Schaeffer^1^, Monica M. Santisteban^1^, Ajay Menon^1^, Hong Jiang^2^, David M. Holtzman^2^, Josef Anrather^1^, Costantino Iadecola^1^

#### ^1^Weill Cornell Medicine, NY, USA; ^2^Washington University in St. Louis, MO, USA

##### **Correspondence:** Giuseppe Faraco - gif2004@med.cornell.edu

*Fluids and Barriers of the CNS* 2019, **16(Suppl 1):**A42

**Objective:** High salt diet (HSD) leads to a reduction in endothelial nitric oxide (NO) associated with profound cognitive impairment 1. However, how endothelial dysfunction leads to cognitive impairment is unclear. Accumulation of phosphorylated tau, a microtubule associated protein linked to Alzheimer’s disease, has also been linked to vascular cognitive impairment and endothelial dysfunction 2–4. Therefore, we tested the hypothesis that HSD affects cognition through tau phosphorylation 5.

**Methods:** C57BL/6 mice were fed HSD (8% NaCl) or normal diet (ND; 0.5% NaCl) for 12 weeks. Tau pathology in the neocortex was assessed by western blotting and immunohistochemistry. Cognitive function was tested by novel object recognition test and Barnes maze. Cerebrovascular function was measured by laser doppler flowmetry and ASL-MRI.

**Results:** HSD increased tau phosphorylation both at Ser202Thr205 (AT8) and Thr231 (RZ3) (AT8: 2.8 ± 0.7; RZ3: 3.1 ± 0.7-fold increase vs ND, p 0.05). HSD-induced cognitive impairment was not observed in mice treated with anti-tau antibodies (HJ8.8) (novel object exploration, HJ8.8: + 19 ± 5% vs HSD + IgG; p < 0.05; n = 12; Barnes Maze, primary latency, HJ8.8: − 41 ± 6% vs HSD + IgG, p < 0.05, n = 15), effects associated with a reduction in AT8 levels in the neocortex (− 46 ± 8% vs HSD + IgG, p < 0.05, n = 10).

**Conclusion:** The deficit in endothelial NO induced by HSD leads to calpain denitrosylation, Cdk5 activation and tau phosphorylation in neurons. The findings unveil a previously-unrecognized link between dietary habits, vascular dysfunction and tau pathology and suggest that avoiding excessive salt intake might be beneficial in maintaining both vascular and cognitive health.

**Grant Support:** R37-NS089323, 1R01-NS095441, SDG15SDG22760007.

**References**

1. Faraco G et al. Dietary salt promotes neurovascular and cognitive dysfunction through a gut-initiated TH17 response. Nat Neurosci. 2018; 21:240–9, 10.1038/s41593-017-0059-z.
2. Raz L et al. Hypoxia promotes tau hyperphosphorylation with associated neuropathology in vascular dysfunction. Neurobiol Dis. 2018; 10.1016/j.nbd.2018.07.009.
3. Qiu L et al. Chronic cerebral hypoperfusion enhances Tau hyperphosphorylation and reduces autophagy in Alzheimer’s disease mice. Sci Rep. 2016; 6:23964. 10.1038/srep23964.
4. Kim HJ et al. Assessment of extent and role of tau in subcortical vascular cognitive impairment using 18F-AV1451 positron emission tomography imaging. JAMA Neurol. 2018; 75:999–1007, 10.1001/jamaneurol.2018.0975.
5. Wang Y, Mandelkow E. Tau in physiology and pathology. Nat Rev Neurosci. 2016; 17:5–21. 10.1038/nrn.2015.1.
6. Patrick GN et al. Conversion of p35 to p25 deregulates Cdk5 activity and promotes neurodegeneration. Nature. 1999; 402:615–22. 10.1038/45159.
7. Etwebi Z, Landesberg G, Preston K, Eguchi S, Scalia R. Mechanistic role of the calcium-dependent protease calpain in the endothelial dysfunction induced by MPO (myeloperoxidase). Hypertension. 2018;71:761–70. 10.1161/hypertensionaha.117.10305.

## A43 Dishevelled interacts with ZO1 and contributes to norrin-induced BRB restoration

### Monica Diaz Coranguez, Cheng-Mao Lin, David A. Antonetti

#### University of Michigan, Kellogg Eye Center, Ann Arbor, MI, USA

##### **Correspondence:** Monica Diaz Coranguez - mdiazcor@med.umich.edu

*Fluids and Barriers of the CNS* 2019, **16(Suppl 1):**A43

**Objective:** Previous studies reveal that norrin reverses VEGF-induced permeability. Here, we explored the contribution of dishevelled (Dvl) isoforms 1, 2 and 3 in norrin-induced blood-retinal barrier (BRB) restoration. We hypothesized that Dvl promotes tight junction (TJ) stabilization through norrin signaling.

**Methods:** BRB properties in primary bovine retinal endothelial cells (BREC) were determined by measurements of transendothelial electrical resistence (TEER) or solute flux of 70 kDa RITC-dextran in control and siRNA treated samples. The interaction between Dvl and ZO1 was analyzed by co-immunoprecipitation (CoIP) assays in BREC, or in HEK293 cells co-transfected with Dvl mutants and ZO1.

**Results:** VEGF induced Dvl accumulation in BREC, specifically Dvl2 and 3, while norrin promoted Dvl phosphorylation. TEER measurements demonstrate that norrin was able to completely restore BRB properties after VEGF and that this effect can be delayed by the knockdown of Dvl1. Similar results were found in flux assays to a 70 kDa RITC-dextran molecule, suggesting that Dvl1 is required for norrin-induced BRB restoration. In contrast, Dvl2 knockdown resulted in decreased VEGF-induced permeability. Together, these results indicate a requirement of Dvl1 for norrin signaling in BREC and suggest an inhibitory role for Dvl2 during the induction of barrier properties. In addition, Dvl immunofluorescence staining showed co-localization of Dvl with ZO1 and claudin-5 at the TJ complex. CoIP experiments demonstrated that Dvl3 formed a complex with ZO1. Further, Dvl3/ZO1 interaction was most abundant in the presence of VEGF/norrin co-treatment. Using co-transfection of Dvl3 mutants and ZO1 in HEK293, mutational analysis identified regions of interaction. The C-terminal PDZ binding domain of Dvl3 but not DIX or PDZ domain deletions, prevented Dvl3/ZO1 interaction, suggesting a role of Dvl3 C-Terminal in its interaction with ZO1.

**Conclusion:** These results demonstrate that norrin signals though Dvl1 to stimulate barrier properties and also promotes Dvl3 direct interaction with ZO1 suggesting a potential role of Dvl with the tight junction. Meanwhile, Dvl2 acts negatively to norrin-induced BRB restoration after VEGF.

**Grant Support:** NIH EY012021, ADA 4-16-PMF-003 and Research to prevent blindness.

## A44 Disruption of the human blood–brain barrier by oxygen–glucose deprivation stress occurs via a HIF-1 independent mechanism

### Abraham Jacob Al-Ahmad, Shyanne Page

#### Texas Tech University Health Sciences Center, Amarillo, TX, USA

##### **Correspondence:** Abraham Jacob Al-Ahmad - abraham.al-ahmad@ttuhsc.edu

*Fluids and Barriers of the CNS* 2019, **16(Suppl 1):**A44

**Objective:** Stroke represents the 5th cause of death in the United States and a leading cause of disability. During and after cerebral ischemic stroke (the most common type of stroke), the blood–brain barrier (BBB) undergoes two major openings, leading to the formation of potential fatal cerebral edema. Targeting such opening may improve stroke patients’ outcomes and minimize the severity of such edema. Yet, current targeting strategies failed to translate, suggesting a possible difference between rodents and human BBB in regards of cellular response to ischemia/reoxygenation injury. The main objective of this study is to elucidate the cellular mechanisms involved at the human BBB eliciting its disruption.

**Methods:** In this study, we developed an in vitro model of the human BBB based on two induced pluripotent stem cell (iPSC)-derived brain microvascular endothelial cells (BMECs) cell lines. As a comparison, hCMEC/D3 cell line was used as an adult somatic brain endothelial cell line. Cells were exposed to oxygen–glucose deprivation (OGD) stress (1% O_2_, glucose-free medium) for 6 h followed by 18 h reoxygenation (21% O_2_, 1 g/L glucose). Changes in the barrier function was assessed by TEER and fluorescein permeability, tight junction complexes by immunocytochemistry whereas the activation of selective signaling pathways was assessed by ELISA.

**Results:** Our data indicates that iPSC-derived BMECs behave similarly than hCMEC/D3 cell lines, as we noted a decreased barrier function following OGD stress. Notably, we noted a differential response between the two iPSC-derived BMECs monolayers. Such disruption correlated with an increase in HIF-1 protein levels during OGD. However, increase in secreted VEGF levels showed a maximum during the reoxygenation phase.

Activation of HIF-1 under normoxia using prolylhydroxylases domain inhibitors (DMOG, FG-4492) only resulted in a mild disruption of the barrier function, whereas inhibition of HIF-1 or VEGF signaling pathways failed to block OGD-induced barrier disruption or worsened the barrier outcomes.

**Conclusion:** Our data indicates that iPSC-derived BMECs are capable to respond to ischemia/reoxygenation as somatic cells, such response occurs likely involve a HIF-1 independent mechanism. Preliminary data suggest a possible involvement of the canonical WNT signaling pathway in such response.

**Grant Support:** TTUHSC Institutional Support, Laura W. Bush Institute of Women’s Health.

## A45 Diverse fluid shear stress regimes alter HBMEC cell–cell junction structure

### Robert Steward Jr., DIlshan Ranadewa

#### University of Central Florida, Orlando, FL, USA

##### **Correspondence:** Robert Steward Jr. - rstewardjr@ucf.edu

*Fluids and Barriers of the CNS* 2019, **16(Suppl 1):**A45

**Objective:** HBMECs exist in arguably one of the most mechanically diverse environments in the body as they experience multiple fluid shear stress regimes. Fluid shear stress is well understood to influence HBMEC permeability, which in part dependent on endothelial cell–cell junctions. Furthermore, HBMEC cell–cell junctions have been demonstrated to also influence intercellular communication and cell–cell adhesion. However, the influence of various fluid shear stress regimes such as laminar flow and oscillatory flow on HMBEC cell–cell junction structural organization is currently unknown. Therefore, our objective was to investigate the influence of laminar and oscillatory flow on HBMEC cell–cell junction structure and organization.

**Methods:** HBMECs were seeded at a concentration of 2.50 × 105 cells/mL in a laminar flow chamber for 24 h. After this time, a programmable, peristaltic flow pump was used to expose HBMECS to independent trials of laminar and oscillatory fluid shear stress at high (10 dynes/cm^2^) and low (1 dynes/cm^2^) magnitudes. After the cessation of experiments HBMECs were fixed and stained for the tight junctions ZO-1 and Claudin-5 and the adherens junctions JAM-A & VE-Cadherin. Stained adherens junction and tight junction images were subsequently analyzed for localization and structure using ImageJ image processing software.

**Results:** Claudin-5 was observed to localize primarily around the cell periphery in static (no fluid flow) conditions, but was observed to localize throughout the entire cell under all forma of fluid shear stress. ZO-1, Ve-Cadherin, and JAM-A where observed to localize through the entire cell body in all fluid shear stress regimes as well as in static conditions. Further analysis of cell–cell junction structure revealed Z0-1 and Claudin-5 to exhibit the highest structural reorganization under a low oscillator flow of 1 dynes/cm^2^, while JAM-A and Ve-Cadherin exhibited the highest structural junctional reorganization at a low laminar flow of 1 dynes/cm^2^ and high laminar flow of 10 dynes/cm^2^, respectively.

**Conclusion:** Our findings of tight junction and adherens junction structure to be dictated by fluid shear stress regeimes suggests that bbb structure is fluid flow-dependent and we therefore believe these findings will be useful to the field of bbb and cerebrovascular biology.

**Reference**

1. Colgan O, Ferguson G, Collins NT. Regulation of bovine brain mi-crovascular endothelial tight junction assembly and barrier function by laminar shear stress. Am J Heart Circ Physiol. 2007. 10.1152/ajpheart.01177.2006.

## A46 Dual PI3K/Akt inhibition to overcome blood–brain barrier P-glycoprotein and breast cancer resistance protein

### Julia Schulz, Anika Hartz, Bjoern Bauer

#### University of Kentucky, Lexington, KY, USA

##### **Correspondence:** Julia Schulz - julia.schulz@uky.edu

*Fluids and Barriers of the CNS* 2019, **16(Suppl 1):**A46

**Objective:** Glioblastoma (GBM) is one of the deadliest cancers with a median survival of only 15–23 months after diagnosis. Even aggressive treatment that includes tumor resection, radiation and chemotherapy fails and does not substantially prolong patient survival.

One reason for therapeutic failure are the drug efflux transporters P-glycoprotein (P-gp) and Breast Cancer Resistance Protein (BCRP) at the blood–brain barrier (BBB). P-gp and BCRP limit anticancer drugs from entering the brain and eradicating remnant tumor cells, resulting in tumor recurrence. While transporter inhibition combined with chemotherapy is a successful treatment option in mice, this is not a viable approach in patients due to severe adverse effects. Thus, new therapeutic strategies are necessary to improve the brain delivery of anticancer drugs.

In the present study, we are testing a novel molecular switch approach: dual inhibition of PI3K/Akt to decrease P-gp and BCRP expression and activity levels at the BBB. We hypothesize that this approach will provide a window-in-time to allow delivery of anticancer drugs into the brain.

**Methods:** To test this hypothesis, brain levels of anticancer drugs were determined with brain perfusion. P-gp was inhibited with PSC833; BCRP was inhibited with fumitremorgin C. PI3K/Akt were inhibited with LY294002/triciribine. Mdr1a/b, Bcrp and triple KO mice were used as positive controls for drug brain uptake.

**Results:** Transporter inhibition with PSC833, fumitremorgin C, or a combination of both inhibitors significantly increased brain levels of anticancer drugs. Dual PI3K/Akt inhibition decreased P-gp and BCRP expression and activity levels, which resulted in significantly increased brain levels of anticancer drugs.

**Conclusion:** Dual PI3K/Akt inhibition is potentially a useful molecular switch to temporarily turn off P-gp/BCRP for the purpose of improving brain drug uptake. Future research will focus on testing this strategy in mouse glioblastoma models with the goal of reducing tumor size and prolonging survival.

**Grant Support:** Pharmaceutical Sciences Excellence Fellowship to JAS; ACS Institutional Grant and NINDS/NIH R01NS107548 to BB.

## A47 Dysregulation in the blood–brain barrier and neurovasculature induced by prenatal immune disturbances

### Hyang Mi Moon, Brooke Babineau, Jacob Umans, Hannah Nguyen, Theo D. Palmer

#### Stanford University, Palo Alto, CA, USA

##### **Correspondence:** Hyang Mi Moon - chorong@stanford.edu

*Fluids and Barriers of the CNS* 2019, **16(Suppl 1):**A47

**Objects:** Dysfunction of the blood–brain barrier (BBB) and neurovasculature has been implicated in the pathophysiology of neurodevelopmental disorders such as autism spectrum disorder (ASD) and schizophrenia (SCZ). BBB-associated endothelial cells regulates selective movements of ions, nutrients, metabolites, and immune cells. Prior genetic profiling from SCZ patient-derived endothelial cells uncovered dysregulation of the angiogenic pathways. Elevated peripheral neuroinflammatory biomarkers and immune cell infiltration in SCZ patients’ brains suggested aberrant BBB permeability. Downregulation of Claudins and Occludin, BBB-associated tight junction proteins, has been detected in the SCZ patients’ brains. Genetic studies also confirmed that a CLAUDIN-5 allele polymorphism is linked to SCZ psychosis and its haploinsufficiency was associated with 22qDel syndrome. Intriguingly, BBB abnormalities in ASD were reported in a recent GWAS study identifying LAT-1 mutations in rare ASD cases. Together, these previous findings suggest that BBB dysfunction may underpin the pathogenesis of neurodevelopmental disorders. However, the exact cellular mechanisms by which BBB disruptions cause the neuropathology of aforementioned diseases have not been elucidated.

**Methods and results:** We hypothesized that neuroinflammation may result in endothelial cell dysregulation and BBB disorganization, leading to neocortical patterning defects. To investigate mechanisms by which BBB disruptions impair fetal brains, we prenatally challenged mice with immune activators. Maternal immune activation elicited cytokine responses in the maternal periphery, placentas, and fetal brains. Through further fetal brain expression analyses, we demonstrated downregulation of multiple target genes encoding tight junctions, adherens junctions, and metalloproteinases at the BBB. These modulating effects on the BBB were also accompanied by suppression of the transcriptional factors of neural progenitors’ self-renewal and mislocalization of neural subtypes. In prenatally exposed neocortex, the blood vessel density and branching points were reduced with aberrant neurovascular patterning.

**Conclusions:** Our study identified novel molecular pathways which regulate BBB formation during early neurodevelopment. This study also highlights that these networks are highly vulnerable to prenatal immune disturbances during the critical time points of brain development. These findings provide important evidence that modulation of the BBB integrity and homeostasis may serve as a new therapeutic strategy to mitigate neuroinflammation by restoring endothelial cell functions and regulating crosstalk between the brain and periphery.

**Grant Support:** SFARI Pilot #32322, postdoc fellowship from the Stanford Child Health Research Institute, NIH-R01 MH108659.

## A48 Effect of exercise on cognition and behavioral changes in mice after methamphetamine induced neurotoxicity

### Marta Przybyła^1^, Arkadiusz Liśkiewicz^1^, Daniela Liśkiewicz^1^, Marta Nowacka-Chmielewska^1^, Andrzej Małecki^1^, Michał Toborek^2^

#### ^1^The Jerzy Kukuczka Academy of Physical Education, Katowice, ŚL, Poland; ^2^Miller School of Medicine, University of Miami, FL, USA

##### **Correspondence:** Marta Przybyła - mprzybyla666@gmail.com

*Fluids and Barriers of the CNS* 2019, **16(Suppl 1):**A48

Abuse of methamphetamine (METH) and related compounds are social and health global problem. Due to its high neurotoxicity the METH users develop cognitive disabilities by dysregulation of various systemic and cellular functions. In addition to its psychostimulatory impact, methamphetamine has also substantial neuro- and vascular toxicity and stimulates disruption of the blood–brain barrier (BBB).

The present study evaluates the protective effect of exercise on METH-induced neurotoxicity and related behavioral changes in mice. Active (exercised) or inactive animals were administered with METH or vehicle injections for 5 days to develop drug-related neurotoxicity. The active mice exercised voluntarily and were provided with running wheels for 2 weeks before injections. Anxiety of animals from both groups were analyzed by means of the open field task (OF) and elevated plus maze task (EPM). Cognitive abilities were evaluated by Morris water maze task (MWM). Based on these findings we have settled that neurotoxic effect of METH does not have a significant impact on behavior but the anxiolytic effect of exercise was observed. METH impaired spatial learning abilities in inactive mice but these cognitive changes were not present in exercised individuals. Overall, our results indicate that METH affects learning abilities but exercise may mitigate this effect. Furthermore physical exercise poses anxiolytic effect, independently to METH treatment. We propose that exercise can protect against drug toxicity which may contribute to more common use of behavioral therapies based on exercise in addiction treatment.

**Grant Support:** The study was supported by the National Science Centre (NSC) grant 2015/17/B/NZ7/02985.

**References**

1. Park M, Levine H, Toborek M. Exercise protects against methamphetamine-induced aberrant neurogenesis. Sci Rep. 2016; 6:34111
2. Marshall JF, O’Dell SJ. Methamphetamine influences on brain and behavior: unsafe at any speed? Trends Neurosci. 2012; 35:536–45

## A49 Effect of high fat diet on cerebrospinal fluid secretion in the rat

### Ester Pascual-Baixauli^1^, Cheryl Hawkes^2^, Jane Loughlin^2^, Basil Sharrack^3^, Ignacio A Romero^2^

#### ^1^The Open University, Milton Keynes, UK; ^2^Life, Health and Chemical Sciences, The Open University, UK; ^3^Academic Department of Neuroscience, Royal Hallamshire Hospital, UK

##### **Correspondence:** Ester Pascual-Baixauli - ester.pascual-baixauli@open.ac.uk

*Fluids and Barriers of the CNS* 2019, **16(Suppl 1):**A49

Idiopathic Intracranial Hypertension is a rare condition in which there is increased intracranial pressure for unknown reasons. It predominantly affects obese women of child-bearing age. This condition might be related to an increased cerebrospinal fluid (CSF) secretion, which is mainly created in the choroid plexus (CP) (1). We wanted to investigate whether a diet rich in fat could alter CSF secretion and gene expression of the CP, and whether these alterations could be related to hormonal changes.

Wistar female rats (4 weeks of age) were exposed to either Normal Diet (ND), High Fat Diet (HFD) or HFD supplemented with a small quantity of Peanut Butter (HFD + PB) for 11 weeks. CSF secretion was measured using the ventriculocisternal perfusion of dextran blue technique in anaesthetised rats. Plasma levels of testosterone, progesterone, oestradiol and cortisol were measured using ELISA. Lastly, the RNA expression profile of CP samples from the lateral ventricles (n = 3) from the three groups was measured using MACE-RNAseq.

As expected, HFD caused an increase in final weight and body fat percentage in the rat, especially in those on the HFD + PB group. In addition, HFD with and without peanut butter altered oestrus cycle length and regularity. CSF secretion increased in both HFD groups compared to ND rats, particularly in the HFD + PB group. Testosterone was the only measured hormone that positively correlated with CSF secretion. Overall, CP from the HFD + PB group showed the largest significant changes in gene expression compared to the ND group, which included downregulation of several CP transporters known to be related to CSF secretion and tight junctional molecules.

In conclusion, HFD did not only affect weight in the rat, it also changed gene expression at the CP level and CSF secretion. Whether the hormone level differences detected were related to the alterations of CSF secretion still remains unknown.

**Reference**

1. Mollan SP, et al. J Neurol Neurosurg Psychiatry 2016;0:1–11. 10.1136/jnnp-2015-311302

## A50 Effects of HIV and Methamphetamine on aberrant cell proliferation of neural progenitor cells

### Minseon Park, William Baker, Dilraj Cambow, Bridget Herlihy, Michal Toborek

#### University of Miami School of Medicine, Miami, FL, USA

##### **Correspondence:** Minseon Park - mspark@med.miami.edu

*Fluids and Barriers of the CNS* 2019, **16(Suppl 1):**A50

**Objective:** For preserving cognitive function throughout life, maintaining an intact pool of functional neural stem cells may be crucial for generating new, functionally active neurons. Methamphetamine (METH) abuse is known to exacerbate HIV-induced deficit of adult neurogenesis. However, it is still unclear as to which mechanisms are involved in METH/HIV-induced functional alterations of neural stem cells. In this study, we provide evidence that METH/HIV enhances exports of FOXO3, a Forkhead box O transcriptional factor, to cytoplasm in the neural progenitor cells (NPCs), which abnormally upregulates the proliferation.

**Methods:** This study was based on a chronically METH-exposed mouse model with an EcoHIV infection and in vitro cell system using ReNcell VM Human neural progenitor cell (hNPCs) from Millipore.

**Results:** For in vivo BrdU incorporation, mice were given i.p. injections of BrdU and sacrificed in 2 weeks. When we compared the number of BrdU-positive NPCs retained in subventricular zone (SVZ) area, more BrdU-positive cells were detected in METH/EcoHIV co-treated mice than control or single treated mice. From ex vivo culture of those NPCs, we found that the NPCs from METH/EcoHIV-brains were growing faster than those of other groups, which suggests that METH/EcoHIV exposure enhances the proliferation of SVZ NPCs. Interestingly, immunoblotting resulted in the presence of CXCL12/SDF1-CXCR4-FOXO3 pathway in ex vivo NPCs exclusively METH/EcoHIV-exposed mice. To evaluate whether METH/HIV co-treatment changes FOXO3 expressions in human NPCs, ReNcells were treated with METH and/or HIV and the protein levels of FOXO3 and the subcellular localization were then compared. The protein levels were significantly increased in HIV-infected hNPCs with or without METH treatment. However, when we compared the FOXO3 protein levels in cytoplasmic, mitochondrial, and nuclear fractions, more FOXO3 proteins were detected in cytoplasmic fractions, whereas less proteins were detected both in nuclear and mitochondrial fractions from the METH/HIV co-treated hNPCs.

**Conclusion:** As FOXO3 is transcriptionally regulating genes, including CDK inhibitors, the changes of subcellular localization of FOXO3 by METH/HIV may closely related to the alterations of NPC proliferation observed in METH/EcoHIV-exposed brains.

**Grant Support:** DA039576, DA040537, DA044579, MH098891, MMH072567, HL126559, and P30AI073961.

**References**

1. Skowronska M et al, Methamphetamine increases HIV infectivity in neural progenitor cells. J Biol Chem. 2018;293(1):296–311.
2. Renault VM et al, FoxO3 regulates neural stem cell homeostasis. Cell Stem Cell. 2009;5(5):527–39.

## A51 Effects of the brain microenvironment on BBB activation in a setting of Cerebral Malaria

### Monique Stins, Midrelle Noumbissi

#### Johns Hopkins Bloomberg SPH, Baltimore, MD, USA

##### **Correspondence:** Monique Stins - mstins@jhmi.edu

*Fluids and Barriers of the CNS* 2019, **16(Suppl 1):**A51

Cerebral malaria (CM) is a severe neurological complication of Plasmodium infection. The signs and symptoms of CM includes seizures, coma and after the infection is resolved, survivors can remain with persistent neurological sequelae.

A hallmark of CM includes the intravascular sequestration of Plasmodium falciparum-infected red blood cells (Pf-IRBC) s without entering into the brain. The sequestration of these Pf-IRBC then results in an activation of the blood brain barrier (BBB). The vascular responses differ dependent on the brain region where sequestration occurs: In the white matter (WM) sequestration is accompanied by small hemorrhages, whereas this is not the case in the GM.

To study the effect of activation of the BBB endothelium in WM versus GM we modified culture conditions to better represent the brain endothelial cells derived from these brain regions. We then exposed the WM and GM endothelium to Pf-IRBC and assessed host responses.

Activation of the BBB by Pf-IRBC resulted in altered transcription and release of pro-inflammatory molecules and those involved in the coagulation cascade, related to EPCR expression and different responses in WM versus GM conditions and with regard to barrier integrity.

Further understanding of the responses of brain cells derived from different brain areas to Pf-IRBC exposure induced micro-environmental changes will l may lead to more targeted interventions and adjunctive drug therapy for CM and neurologic sequelae.

**Grant Support:** NIH R01HL130649-01, RNS090233A, Bloomberg Philantrophies.

## A52 Electronic cigarette exposure increases blood brain barrier permeability and alters neuroimmune response to acute inflammatory insult

### Nathan Heldt

#### Lewis Katz School of Medicine at Temple University, Philadelphia, PA, USA

##### **Correspondence:** Nathan Heldt - nathan.heldt@temple.edu

*Fluids and Barriers of the CNS* 2019, **16(Suppl 1):**A52

**Objective:** Electronic cigarette (EC) use has grown substantially since entry into the US market a decade ago, particularly among adolescents and traditional tobacco users. Despite growing popularity and claims of harm reduction, the health effects of these products outside the lung is poorly understood. Several of the cigarette smoke (CS) constituents with known neurovascular and inflammatory effects are present in EC liquids or formed during the generation of vapor. In the present study, we investigate the impact of EC use on neurovascular integrity and neuroinflammation within in vitro blood brain barrier (BBB) and murine models.

**Methods:** C57BL/6 mice were exposed to 2 h of daily EC or CS vapor, beginning at 8 weeks of age. BBB permeability was assessed following acute (1 week) and chronic (2 month) EC exposure, and leukocyte migration was evaluated using our established model of aseptic meningitis/encephalitis. Impact on isolated microvessel gene expression and cognitive/affective measures (novel object recognition, plus-maze, marble bury) was assessed. Additionally, human primary brain microvascular endothelial cells (BMVECs) and pericyte co-cultures were treated with EC or CS infused media at physiologically relevant concentrations, and subsequently evaluated for changes in BBB integrity and leukocyte-endothelial cell interaction. The role of actin rearrangement and the activity of GTPases Rho and Rac was further investigated using the in vitro model.

**Results:** Acute EC exposure diminished leukocyte-endothelial cell interactions (adhesion, migration) and attenuated BBB permeability associated with concomitant inflammatory insult. These effects were partially mediated through Rho- and Rac- dependent cytoskeletal reorganization. However, chronic EC exposure induced expression of proinflammatory genes in brain microvessels, and further resulted in increased BBB permeability and leukocyte-endothelial cell interactions.

**Conclusion:** Short-term use of EC results in a distinct neuroinflammatory profile when compared with traditional tobacco products, and may interfere with the neuroimmune responses to inflammatory stimuli. Importantly, our data suggests that long-term use of EC alone is sufficient to induce pro-inflammatory changes and alter BBB permeability in vivo. This may promote further peripheral leukocyte interaction with neurovasculature and cause BBB dysfunction.

**Grant Support:** R01DA040619 (YP), T32DA007237 (NH).

## A53 Elucidating glial and vascular responses associated with fibrinogen deposition in experimental Cerebral Malaria

### Gracie Vargas^1^, Olivia Solomon^1^, Paula Villarreal^1^, Kyle D. Wilson^1^, Lorenzo Ochoa^1^, Florentin Y. Aussenec^1^, Rahul Pal^2^, Astrid Cardona^3^, Robin Stephens^1^

#### ^1^The University of Texas Medical Branch, Galveston, TX, USA; ^2^Massachusetts General Hospital, Boston, MA, USA; ^3^University of Texas, San Antonio, USA

##### **Correspondence:** Gracie Vargas - grvargas@utmb.edu

*Fluids and Barriers of the CNS* 2019, **16(Suppl 1):**A53

**Objective:** Cerebral malaria (CM) is a severe encephalopathy resulting from *Plasmodium falciparum* infection, yearly afflicting 575,000 people. CM is associated with high mortality and, in survivors, neurological sequelae. Though correlations exist between cytokines, coagulopathies, and mortality, the roles of coagulation and inflammation are poorly understood. Previously, we showed using immunofluorescence microscopy, thrombi/fibrinogen deposition in brain microvessels of the IL-10 KO *Plasmodium chabaudi* CM model, with microglial and astrocyte activation. Here, we apply intravital microscopy and large-scale microscopy of optically cleared brain to investigate fibrinogen deposition and dynamic vascular and glial responses in experimental CM, expanding inquiry into a Plasmodium berghei ANKA(PbA)-model.

**Methods:** Cortical intravital multiphoton microscopy in PbA-infected and control CX3CR1-GFP mice enabled in vivo study of GFP-microglia, labelled vasculature, and fluorescent fibrinogen. A thinned-skull preparation allowed imaging of skull vasculature, meninges and cortex to a depth of ~ 200 µm. Intravital imaging of *P. chabaudi* infection in IL-10 KO mice was performed to image vascular dynamics. To study fibrinogen deposition, microglial and astrocyte reactivity, CLARITY optical cleared brains were imaged with multiphoton microscopy for large-scale high-resolution analysis. Acquired images were analyzed using ImageJ software to quantify glial activation and vascular responses in eCM.

**Results:** Vessels of the brain were congested with thrombi throughout *P. chabaudi* infection and were colocalized with adherent CD4 Tcells and monocytes. PbA-infected mice had fibrinogen foci within the microvessels of the brain as well as perivascular fibrinogen deposits. Intravital microscopy revealed dynamic microhemorrhage activity with vascular label and fibrinogen extravasation and rapid recovery of the BBB. Activated microglia were found throughout, and in intravital microscopy were observed to uptake fibrinogen in the perivascular space as well as undergo dynamic morphological transformation at sites near vessels containing occlusions. Anticoagulation intervention significantly reduced both thrombus formation and gliosis in *P. chabaudi*-infected IL-10 KO mice.

**Conclusion:** This study provides further insight into the nature of fibrinogen deposition that occurs in CM, which includes both intravascular thrombi and distinct perivascular deposition and provides further insight into in vivo and dynamic vascular and glial responses associated with fibrinogen deposits. Finally, it suggests interactions between coagulation and inflammation in CM.

**Grant Support:** NIH NINDS R01NS106597 (RS and GV), Univ Texas Neuroscience/Neurotechnology Res. Inst (RS, GV).

## A54 Endothelial cell migration during wound healing in three-dimensional tissue-engineered iPSC-derived blood–brain barrier microvessels

### Jackson DeStefano, Raleigh Linville, Alanna Farrell, Erin Pryce, Peter Searson

#### Johns Hopkins University, Baltimore, MD, USA

##### **Correspondence:** Jackson DeStefano - jdestef3@jhu.edu

*Fluids and Barriers of the CNS* 2019, **16(Suppl 1):**A54

**Objective:** Endothelial cell migration plays an important role in various biological and pathological processes including angiogenesis, vasculogenesis, inflammation, and wound healing. Cell migration in response to wound healing has been extensively studied in 2D systems using human umbilical vein endothelial cells [1] but these models fall short in in replicating the geometry of a blood vessel. Here we use three-dimensional tissue-engineered induced pluripotent stem cell (iPSC)-derived human blood–brain barrier microvessel platform [2] to model wound healing of the brain microvasculature and quantify rates of endothelial cell migration in response to laser ablation.

**Methods:** Tissue-engineered iPSC-derived blood–brain barrier microvessel model [2] has physiological permeability, geometry, and shear stress while allowing for multiplexed live-cell imaging to monitor the dynamics of individual endothelial cell behavior as well barrier function via solute permeability. Microfluidic devices are made up of a glass slide, PDMS housing, a genipin-crosslinked type I collagen hydrogel, inlet/outlet fluid reservoirs and a 150-micron diameter microvessel seeded with endothelial cells to create a confluent endothelial cell monolayer which can be continually perfused. Precise defects in the endothelial monolayer are created via laser ablation [3] using a laser scanning microscope, photobleaching a plane of the monolayer at 750 nm for ninety seconds. Microvessel recovery is then quantified in real-time via phase contrast microscopy of the defect, and surrounding cells, over time. To visualize the recovery of tight junctions in real-time we use ZO1-tagged iPSCs.

**Results:** Evans blue dye and 10 kDa Dextran was able to escape the vessel lumen upon ablation of the monolayer. Defect recovery is dominated by cell migration, as there were few of cell division at or around the advancing defect boundary. Endothelial cell speed within the monolayer was decreased the further away a given cell is from the defect. Defects recovered with a speed of 12–25 microns/h, closing a 50-micron defect over 2–4 h.

**Conclusion:** Using this technique we can precisely and repeatably create a defect in our microvessel model. We have quantified that rate of recovery in (iPSC)-derived human brain microvascular endothelial cells and will implement this microvessel damage to model parts of the inflammatory cascade.

**Grant Support:** This work was supported by DTRA (HDTRA1-15-1-0046) and NIH T32: Predoctoral and Postdoctoral Training Program.

**References**

1. van der Meer A, Vermeul K, Poot AA, Feijen J, Vermes I. A microfluidic wound healing assay for quantifying endothelial cell migration. Am J Physiol Heart Circ Physiol. 2009.
2. Linville RM, DeStefano JG, Sklar MB, et al. Human iPSC-derived blood–brain barrier microvessels: validation of barrier function and endothelial cell behavior. Biomaterials. 2019; 190: 24–37.
3. Berthiaume A-A, Grant RI, McDowell KP, et al. Dynamic remodeling of pericytes in vivo maintains capillary coverage in the adult mouse brain. Cell Rep. 2018; 22: 8–16.

## A55 Endothelium- and pericyte-derived α5-containing laminins differentially regulate blood brain barrier integrity after stroke

### Yao Yao^1^, Jyoti Gautam^1^, Abhijit Nirwane^1^, Jeffrey Miner^2^

#### ^1^University of Georgia, Athens, GA, USA; ^2^Washington University, St Louis, MO, USA

##### **Correspondence:** Yao Yao - yyao@uga.edu

*Fluids and Barriers of the CNS* 2019, **16(Suppl 1):**A55

**Objective:** Both endothelial cells and pericytes synthesize laminin-α5 at the blood–brain barrier (BBB). We investigated the roles of endothelial and pericytic laminin-α5 in BBB integrity under both homeostatic and pathological conditions.

**Methods:** Transgenic mice with laminin-α5 deficiency in endothelial cells (α5-TKO) and pericytes (α5-PKO) were generated. Angioarchitecture and BBB permeability were examined in these mutants and their littermate controls under homeostatic conditions. Additionally, BBB integrity and neuronal injury (injury size, neuronal death, and neurological function) were also examined in these mutants after hemorrhagic (for α5-TKO) and ischemic (for α5-PKO) stroke.

**Results:** Under homeostatic conditions, comparable vessel density, vessel length, and branching index were observed in α5-TKO and α5-PKO mutants compared to their littermate controls, indicating unaltered angioarchitecture. In addition, we failed to detect endogenous IgG and intravenously injected Evans blue/FITC-Dextran in the brains of these mutants, indicating intact BBB integrity. Consistent with this finding, no changes in tight junction protein expression, pericyte coverage, and astrocyte polarity were found in these mutants. After hemorrhagic stroke, α5-TKO mice displayed significantly enhanced IgG/Evans blue/FITC-Dextran leakage and increased inflammatory cell infiltration compared to their littermate controls, indicating exacerbated BBB disruption. In addition, enlarged hematoma size, increased neuronal death, and worse neurological function were found in these mutants, suggesting a beneficial role of endothelium-derived α5-containing laminins in hemorrhagic stroke. After ischemic stroke, α5-PKO mice showed reduced Evans blue/FITC-Dextran leakage and diminished inflammatory cell extravasation compared to their littermate controls, indicating attenuated BBB damage. In addition, decreased infarct volume, diminished neuronal death, and improved neurological function were observed in these mutants, suggesting a detrimental role of pericyte-derived α5-containing laminins in ischemic stroke.

**Conclusion:** Endothelium- and pericyte-derived α5-containing laminins are dispensable for angioarchitecture and BBB maintenance under homeostatic conditions. Endothelium-derived α5-containing laminins contribute to BBB integrity and play a beneficial role in hemorrhagic stroke. Pericyte-derived α5-containing laminins negatively regulate BBB integrity and play a detrimental role in ischemic stroke.

**Grant Support:** This work was partially supported by AHA-16SDG29320001 and NIH-R01DK078314.

## A56 Engineering and preclinical evaluation of the blood–brain barrier-crossing single-domain antibodies against IGF1R

### Danica Stanimirovic, Kristin Kemmerich, Alvaro Yogi, Arsalan Haqqani, Abedelnasser Abulrob

#### National Research Council of Canada, Ottawa, ON, Canada

##### **Correspondence:** Danica Stanimirovic - danica.stanimirovic@nrc-cnrc.gc.ca

*Fluids and Barriers of the CNS* 2019, **16(Suppl 1):**A56

**Objective:** Insulin growth factor 1 (IGF1) is transported across the blood–brain barrier (BBB) via a receptor-mediated transcytosis (RMT). However, attempts to utilise IGF1 as molecular carrier for delivery of therapeutics across the BBB failed because of high circulatory levels of insulin growth factor-binding proteins (IGFBPs). To exploit IGF1 transcytosis route for therapeutic delivery across the BBB, we used an alternative approach—development of antibodies selective for IGF1 receptor (IGF1R).

**Methods:** Single-domain antibodies (sdAbs) were raised by llama immunization with the extracellular domain of human IGF1R. Lead sdAbs were selected for IGF1R selectivity, binding affinity, biophysical properties, receptor binding epitopes, and species cross-reactivity. IGF1R sdAbs were humanized, expressed in fusion with human Fc fragment and various ‘cargo’ molecules (including peptides, enzymes and antibodies), or were used in sdAb format to decorate nanoparticles encapsulating nucleic acid cargos (ASO, siRNA). The constructs were evaluated for their ability to deliver cargo molecules across the BBB models in vitro and in animals (mouse, rats and non-human primates).

**Results:** In vitro BBB testing demonstrated enhanced transcytosis of various proteins fused with-, as well as nanoparticles decorated (via click-chemistry) with IGF1R sdAb compared to those fused/decorated with control sdAbs in rat, mouse and human in vitro BBB models. Similarly, enhanced CSF and brain exposure was observed with IGF1R sdAb-containing fusion proteins and nanoparticles after systemic injection in mouse and rat. BBB carriers chemically conjugated with the neuropeptide Galanin or genetically fused with the neuropeptide Neurotensin showed dose-dependent pharmacodynamic responses in Hargreaves model of inflammatory pain in rodents and telemetric thermoregulation model in both rodents and non-human primates, respectively. Pharmacodynamic responses induced by neuropeptide cargos linked to IGF1R sdAbs variants correlated well with their observed in vitro BBB Papp and their measured CSF/serum and brain exposure profiles, and their potency was similar or higher compared to BBB-crossing small molecule benchmarks.

**Conclusions:** Antibody design and engineering strategies yielded novel, species cross-reactive sdAbs against IGF1R capable of delivering therapeutic cargos across the BBB, which overcome some of the shortcomings of transferrin- and insulin receptor targeting antibodies and provide versatility and modular designs for various engineered fusion therapeutics.

## A57 EphA4 suppresses endothelial cell-specific Tie2 receptor signaling to limit pial collateral remodeling following ischemic stroke

### Michelle Theus

#### Virginia Tech, Blacksburg, VA, USA

##### **Correspondence:** Michelle Theus - mtheus@vt.edu

*Fluids and Barriers of the CNS* 2019, **16(Suppl 1):**A57

**Objective:** Leptomeningeal anastomoses or pial collateral vessels play a critical role in cerebral blood flow (CBF) restoration following ischemic stroke. While activation of the endothelium is implicated in collateral remodeling, the molecular mechanism(s) involved remain under investigation. Our previous findings suggest endothelial cell (EC)-specific EphA4 receptor tyrosine kinase negatively regulates collateral remodeling. The current study evaluated whether EphA4 suppresses pial collaterals are regulated by these mechanisms.

**Methods and results:** We find using EphA4f/f/Tie2-Cre conditional knockout mice, that loss of EphA4 significantly enhances pial collateral remodeling and functional recovery following permanent middle cerebral artery occlusion (pMCAO). This correlated with a significant improvement in CBF and reduced infarct volume compared to EphA4f/f wild type mice. Interestingly, EphA4f/f/Tie2-Cre mice showed increased levels of p-Akt and angiopoietin-2 protein expression at 24 h post-pMCAO. We further elucidated the cross talk between EphA4 and angiopoietin-2/Tie2 pathway using soluble Tie2-Fc following pMCAO. Inhibition of Tie2 signaling ameliorated pial collateral vessel remodeling, neuroprotection and p-Akt activation in EphA4f/f/Tie2-Cre mice, which coincided with attenuated endothelial cell proliferation in vitro. Lastly, we demonstrate that blocking EphA4, using KYL peptide inhibitor delivered via mini-osmotic pumps enhanced pial collateral remodeling after pMCAO.

**Conclusions:** These novel findings demonstrate that EphA4 negatively regulates collateral remodeling by suppressing p-Akt/Tie2 signaling. Therapeutic targeting of EphA4 and/or Tie2 represents an attractive new strategy for improving collateral function, neural tissue health and functional recovery following stroke.

**Grant Support:** R01NS096281 (MHT), F31NS095719-01A1 (BO), R15NS081623-01A (MHT).

## A58 Establishment of an in vitro model of the blood brain barrier for studying brain metastasis

### Joanna Pyczek, Nicholas Gutowski, Jacqueline Whatmore

#### University of Exeter, Exeter, United Kingdom

##### **Correspondence:** Joanna Pyczek - j.p.pyczek@exeter.ac.uk

*Fluids and Barriers of the CNS* 2019, **16(Suppl 1):**A58

**Objective:** Our aim is to establish an in vitro model of the blood brain barrier (BBB), which will closely resemble in vivo conditions and will be suitable for studying transmigration of circulating tumour cells during metastasis to the brain.

**Methods:** Varying co-culture models composed of human cerebral microvascular endothelial cells (hCMEC/D3), primary human astrocytes and/or primary human brain vascular pericytes were established in transwell inserts with collagen IV coated porous membranes. Barrier function was assessed by measuring permeability to FITC-albumin. Transendothelial electrical resistance (TEER) was measured using chopstick electrodes. Tight junction formation/opening was indirectly assessed by treatment with 100 mM histamine. Expression of CD31 was examined via immunofluorescent staining.

**Results:** Immunofluorescent staining of CD31 confirmed the formation of an endothelial cell monolayer. All of the tested models showed markedly increased TEER (ranging from 185 to 202 Ω) when compared to the empty insert control (138 Ω). No significant increase in TEER was noted when monoculture of hCMEC/D3 was compared to the more complex co-culture models. Interestingly, non-contact co-culture of hCMEC/D3 with pericytes showed slightly decreased TEER (185 Ω and 188 Ω) when compared to the other models (197 Ω, 201 Ω and 202 Ω). Moreover, all the models displayed greatly reduced permeability to FITC-albumin [9.2 ± 1.8 − 12.1 ± 1.4 relative fluorescent units (RFU)] vs the empty insert (402.3 ± 15.4 RFU). No significant difference between models was observed. Treatment with 100 mM histamine markedly reduced TEER in nearly all models, but did not affect the permeability to FITC-albumin.

**Conclusion:** We established a 3-cell contact model in transwell inserts, composed of endothelial cells grown on the luminal side of the chamber and mixture of astrocytes and pericytes grown on the abluminal side thus resembling intercellular interactions in vivo. Although this model does not differ much from the simple endothelial cell monolayer in terms of performed experiments, it is clearly more physiological. The model is also enriched in collagen IV, which maintains pericytes in an undifferentiated state. High TEER values and low permeability to FITC-albumin indicate that our model is suitable for BBB studies. Further confirmation of tight junction formation as well as expression and activity of specific transporters is needed to fully evaluate the model.

**Grant Support:** FORCE Cancer Charity Exeter.

## A59 Ethanol gestational exposure triggers brain endothelial dysfunction and alters BBB development

### Joice Stipursky, Michele Siqueira

#### Universidade Federal do Rio de Janeiro (UFRJ), Rio de Janeiro, Brazil

##### **Correspondence:** Joice Stipursky - joice@icb.ufrj.br

*Fluids and Barriers of the CNS* 2019, **16(Suppl 1):**A59

**Objectives:** Ethanol consumption by women in pregnancy or lactation period can induce permanent damage to the developing central nervous system (CNS), resulting in fetal alcohol spectrum disorders (FASD). Among damages to CNS, early ethanol exposure induces neural progenitors´ apoptosis, deficits in cell differentiation and in blood vessel (BV) development and function. Although correct CNS development depends on proper processes of vascularization and BBB establishment, little is known how ethanol affects endothelial cells (EC) function, cerebral vascular network formation and EC association with astrocyte during BBB development, in the context of gestational exposure to ethanol.

**Methods and results:** Immunocytochemistry assays revealed that treatment of human brain microcapillary endothelial cells (HBMEC) cultures with 50 mM ethanol for 2 h, decreased the levels of GLUT-1 transporter by 35%, disrupted ZO-1 distribution by 50%, and increased Nitric oxide levels in EC conditioned medium (EC-CM) by 54%, compared to control condition. Ethanol increased the levels of catalase enzyme by 50%, without affect MRP1/ABCC1 transporter levels. Further, ethanol globally increased the levels of secreted angiogenesis-related factors, such as PIGF2, MMP3, CCN3, IGFBP2, TIMP1 and others, in the EC-CM, revealed by angiogenesis-mini proteome assay. In vivo immunohistochemistry analysis of cerebral cortex of newborn mice offspring from ethanol exposed pregnant Swiss female mice (from gestational day 10 to 19; 1 dose/day 3.0 g/kg by gavage) demonstrated, significant increase in cortical angiogenesis (vessel density [25%], length [30%] and branch points [50%]), revealed by IsolectinB4 labeling. Aberrant distribution of ZO-1 and ensheatment of BV by GFAP positive astrocytes were observed in this model. In vitro, treatment of control cortical astrocytes with conditioned medium from EC previously exposed to ethanol (CM-CE-EtOH), increased the expression of GFAP by 10× and BBB-enriched genes Connexin43 by 10×, and Lcn2 by 100×, compared to astrocytes exposed to control EC-CM. Pro-inflammatory cytokines IL1-beta, IL-6 and TNF-alpha genes expression were significantly induced by 17, 12 and 20× respectively, in the presence of CM-CE-EtOH.

**Conclusion:** We suggest that ethanol triggers a dysfunctional phenotype in brain ECs, leading to impairment of cortical vascular network formation and promoting EC induced-astrocyte dysfunction, which could dramatically affect BBB formation and function in the developing brain.

**Grant Support:** International Society for Neurochemistry (ISN), FAPERJ, CAPES, CNPq.

## A60 Evaluation of brain fractionation methods for multiplex quantification of blood brain barrier-crossing antibodies and cellular markers using mass spectrometry-based methods

### Arsalan S. Haqqani, Christie E. Delaney, Alexandra T. Star, Xigeng Zhao, Wen Ding, Danica B. Stanimirovic

#### National Research Council Canada, Ottawa, ON, Canada

##### **Correspondence:** Arsalan S. Haqqani - Arsalan.Haqqani@nrc-cnrc.gc.ca

*Fluids and Barriers of the CNS* 2019, **16(Suppl 1):**A60

**Background:** Receptors undergoing receptor-mediated transcytosis (RMT) at the blood–brain barrier (BBB) have been exploited as ‘carriers’ of therapeutics across the BBB. FC5, an RMT receptor-targeting antibody at the BBB, has been shown to ‘carry’ therapeutics across the mammalian BBB (1). Brain parenchymal delivery of FC5 has been demonstrated following systemic injection using immunocytochemistry-based methods (1) and molecular imaging (2). Such methods do not provide absolute identification (sequence information) and quantification (pharmacokinetics) of antibodies at the target site. While sensitive mass spectrometry (MS) methods have provided evidence of both identification and quantification in CSF and whole-brain extracts following systemic injection (1–3), such absolute evidence is lacking for demonstrating co-localization with parenchymal brain cells.

**Objective:** Various brain fractionation methods were evaluated to identify a method that allows MS-based quantitation of BBB-crossing antibodies and co-localization with brain parenchymal cell fractions.

**Methods:** Perfused brains were harvested 24 h post-intravenous injection of 15 mg/kg FC5-Fc in rats, homogenized and fractionated using vessel-depletion methods: dextran-based separation or using membrane filters of various sizes and combination. Each fraction (retentate and filtrate) was analyzed by proteomics and selected-reaction monitoring of cell markers. Data was analyzed by MatchRx software clustered with published single-cell sequencing datasets to identify brain cells enriched in fractions.

**Results:** Overall results indicate that membrane filter-based fractionation is superior over dextran-based method for vessel and parenchyma separation. Using a specific combination of filter sizes, the membrane filters also demonstrate separation of other cellular fractions as determined by levels of cell-specific proteins being enriched in individual fractions. These include fractions enriched in specific markers of large vessels, microvessels, capillaries, astrocytes and neurons. Preliminary results demonstrate that FC5-Fc is predominantly present in brain parenchymal fractions and absent (below limits-of-detection) in large vessels, microvessels and capillaries in perfused brains of rats injected with the BBB-crossing antibody.

**Conclusion:** Brain fractionation using a specific combination of membrane filter sizes can quantify levels of BBB-crossing antibodies and co-localize them with vessel or parenchymal cells using MS methods. The method has a potential for quantitatively comparing brain distribution of multiple BBB ‘carriers’ to optimize brain delivery of therapeutics.

**References**

1. Haqqani AS, Caram-Salas N, Ding W, et al. Mol Pharm. 2013; 10:1542–56
2. Ribecco-Lutkiewicz M, Sodja C, Haukenfrers J, et al. Sci Rep. 2018. 10.1038/s41598-018-19522-8
3. Iqbal U, Abulrob A, Stanimirovic DB. Methods Mol Biol. 2011;686:465–81

## A61 Evaluation of the ability of medical countermeasures to reach the central nervous system using an in vitro blood brain barrier

### Terese Karlsson

#### Swedish Defense Research Agency, Umeå, VB, Sweden

##### **Correspondence:** Terese Karlsson - terese.karlsson@foi.se

*Fluids and Barriers of the CNS* 2019, **16(Suppl 1):**A61

**Objective:** The blood brain barrier (BBB) separates the central nervous system (CNS) from systemic circulation and prevents entry of potentially harmful molecules and pathogens. Simultaneously, the BBB-system allows passage of molecules crucial for CNS homeostasis and function. The BBB consists of vascular endothelial cells, pericytes and astrocytes and form a strong and selective barrier. The BBB is a major challenge for drug delivery to the CNS and one example is the standard treatment regimen (atropine and oxime) following nerve agent intoxication. These drugs have poor BBB penetration ability and therefore have low effectiveness in preventing CNS injuries.

**Methods:** To enable studies of medical countermeasure transport across the BBB, an in vitro method was established using induced pluripotent stem cells (iPSCs) which were differentiated into a functional BBB.

**Results:** Initially, the BBB-model was characterized regarding expression of specific molecular markers, such as transporter proteins and tight junction proteins, and barrier integrity using transendothelial electrical resistance. Results showed that critical markers were specifically expressed and a tight and consistent barrier integrity was achieved. By using Cyclosporin A or Tariquidar, inhibition of the main active BBB-transporter p-glycoprotein was obtained which indicated functionality of the barrier.

The ability of two anticholinergic drugs (atropine and scopolamine) to pass the BBB has been evaluated. Preliminary results displayed greater penetration of scopolamine compared to atropine despite their molecular similarity. Ongoing studies are now focusing on the molecular mechanisms behind differences in BBB-passage between the two anticholinergic drugs. In addition, atropine analogues with different physicochemical properties are being evaluated.

**Conclusion:** The established in vitro method using iPSCs is clearly suitable for studying the passage of medical countermeasures across the BBB. Studies will result in important understanding of critical drug properties and BBB-functionalities to support development of centrally active nerve agent medical countermeasures.

## A62 Exosomal miRNA-23a-mediated loss of pericyte coverage at the blood–brain barrier: Implications for morphine-mediated neuroinflammation

### Shilpa Buch, Ke Liao, Fang Niu, Guoku Hu, Susmita Sil

#### University of Nebraska Medical Center, Omaha, NE, USA

##### **Correspondence:** Shilpa Buch - sbuch@unmc.edu

*Fluids and Barriers of the CNS* 2019, **16(Suppl 1):**A62

**Objective:** To examine the effect of morphine-exposed astrocyte-derived EV (ADEV) miRNAs in mediating loss of pericytes at the BBB which in turn, leads to BBB breach.

**Methods:** EVs were purified from control and morphine-exposed astrocytes using the standard differential ultracentrifugation technique followed by characterization using western blot, transmission electron microscopy, AchE assay, atomic force microscopy and NanoSight analyses. Exposure of EVs from control and morphine-exposed astrocytes to pericytes resulted in pericyte migration as evidenced by Boyden chambers and wound healing assays as well using the endothelial-pericyte 3D co-culture model. Validation ex vivo was also done by immunostaining microvessels isolated from morphine-administrated mice for PDGFbR+ pericytes and for CD31+ brain endothelial cells. The influx of monocytes in the CNS and the loss of pericytes was also validated in sections of brains from pericyte-labeled NG2-DsRed mice administered morphine for 7d.

**Results:** Our data demonstrated that morphine-exposed astrocytes induced the expression and secretion of miR-23a in the EVs which were taken up by the pericytes, leading in turn, to their migration. Additionally, we found downregulation of PTEN—a target of miR-23a, in pericytes exposed to morphine-ADEVs. Additional validation of decreased pericyte coverage was also demonstrated ex vivo, wherein in morphine-administrated mice, fewer PDGFβR+ pericytes were found in the vicinity of CD31+ brain endothelial cells in the isolated microvessels. In keeping with these observations and of importance, we also observed increased influx of monocytes and increased loss of pericytes in sections of brains from morphine-administrated NG2-DsRed mice.

**Conclusion:** Our findings indicate that morphine-mediated dysregulation of miRNA expression in the brain involves EV-mediated cell–cell communication between the astrocytes and the pericytes, which in turn, leads to pericyte loss at the BBB that is accompanied with a concomitant influx of monocytes.

## A63 Exploring endothelial cell dysfunction: a potential new in vivo model for Cerebral Small Vessel Disease

### Sophie Quick^1^, Jon Moss^1^, Mairi Brittan^2^, Joanna Wardlaw^2^, Anna Williams^1^

#### ^1^University of Edinburgh, Edinburgh, United Kingdom; ^2^Centre for Cardiovascular Science, Edinburgh, UK

##### **Correspondence:** Sophie Quick - sophie.quick@ed.ac.uk

*Fluids and Barriers of the CNS* 2019, **16(Suppl 1):**A63

**Objective:** Cerebral small vessel disease (cSVD) is the leading cause of vascular dementia and triples patients’ risk of stroke. Recent work from our lab using the Spontaneously Hypertensive Rat Stroke Prone (SHRSP) model of cSVD indicates that the underlying cause is not simply hypertension but an inherent dysfunction in endothelial cells of the blood–brain barrier, which causes a maturation block on the oligodendrocytes of the white matter (Rajani et al. 2018). We showed that this rat model has a homozygous deletion mutation of the flippase ATP11B, which is sufficient to cause endothelial dysfunction, and that single nucleotide polymorphisms in ATP11B are associated with humans with sporadic cSVD. To better elucidate the effects of endothelial dysfunction in this disease, we characterised a novel ATP11B knock-out (ATP11BKO) transgenic rat to examine how well it reflects cSVD pathology.

**Methods:** We used histology, immunofluorescence and protein quantification to characterise classic markers of (1) endothelial dysfunction in brain tissue of ATP11BKO rats and (2) white matter changes including oligodendroglia maturation. Furthermore, we examined cultured endothelial cells both from ATP11BKO rats and human endothelial cells with knocked down ATP11B expression in vitro for signs of dysfunction and effects on oligodendroglia.

**Results:** Endothelial dysfunction is demonstrated in the ATP11BKO rat model with an increase in proliferation, mis-localisation of tight junction marker CLDN5 and increased levels of ICAM-1. Effects on oligodendroglia are shown by reduced maturation. We further demonstrate relevance to human disease by ATP11B knockdown in human endothelial cells, leading to similar dysfunction including proliferation, nitric oxide production and tight junction loss, and subsequent oligodendroglial maturation block.

**Conclusion:** The link between the blood–brain barrier and white matter changes in cSVD is poorly understood but understanding this may lead to potential new targets for therapies. The ATP11BKO rat will provide a novel platform to study endothelial dysfunction and may offer a new model of cSVD to trial new approaches to tackling this disease.

**Reference**

1. Rajani RM, Quick S, Ruigrok SR, Graham D, Harris SE, Verhaaren BFJ, et al. Reversal of endothelial dysfunction reduces white matter vulnerability in cerebral small vessel disease in rats. Sci Transl Med. 2018; 10(448):eaam9507.

## A64 Exposure to endotoxin reduces cerebral cortical vessel density in preterm fetal sheep

### Barbara S. Stonestreet^1^, Clemence Disdier^1^, Fares Awa^1^, Simerdeep Dhillon^2^, Robert Galinsky^3^, Joanne Davidson^2^, Chris Lear^2^, Alistair Gunn^2^, Laura Bennet^2^

#### ^1^Women & Infants Hospital/Brown University, Providence, RI, USA; ^2^The University of Auckland, Auckland, New Zealand; ^3^The Hudson Institute, Washington, DC, USA

##### **Correspondence:** Barbara S. Stonestreet - bstonestreet@wihri.org

*Fluids and Barriers of the CNS* 2019, **16(Suppl 1):**A64

**Background:** Chorioamnionitis results in premature delivery. Exposure to inflammation represents an important component of injury to the premature brain. Previous work suggests a possible role for inflammation-related stimulation of the microvasculature and potential that lipopolysaccharides (LPS) could be angiogenic. This system is highly regulated by cellular interactions and cerebrovascular dysfunction can further exacerbate brain injury.

**Objective:** To investigate cerebrovascular adaptations to chronic inflammation in the preterm brain.

**Methods:** Chronically-instrumented fetal sheep at 0.7 gestation received continuous low-dose LPS intravenous infusions n = 7, 100 ng/kg over 24 h, followed by 250 ng/kg/24 h for 96 h) or saline (n = 6). Boluses of 1 μg LPS or equivalent volume of saline were given at 48 and 72 h. Brain maturation of the sheep at this stage of gestation is broadly equivalent to 28–32 weeks of human development. We examined vessel density using immunohistochemical methods with Collagen IV as a marker for the microvessel basal lamina. Quantification of vascularization was examined in randomly selected areas of preterm fetal sheep cerebral cortex from placebo treated and LPS treated groups. 10 fields per animal and n = 7 in the placebo treated group, n = 6 in the LPS treated group were analyzed. Statistical analysis by Mann–Whitney U test.

**Results:** Collagen IV (Col IV) staining was used to visualize the microvascular network in the preterm fetal cerebral cortex in placebo treated sham and LPS treated animals, 5 days after the end of the exposure to LPS or placebo. The analysis of Col IV staining at low magnification revealed a decrease (P < 0.05) in the Col IV immunoreactivities in the cerebral cortical fields in LPS treated animals compared to placebo treated group. The observation suggests LPS-induced reductions in the basal lamina in the preterm fetal brain.

**Conclusion:** Inflammation triggered by chronic LPS exposure in the preterm ovine fetus produces neurovascular remodeling and abnormalities. We speculate that this finding could be associated with blood–brain barrier dysfunction and that these vascular changes could lead to inadequate supply to the brain parenchyma and may, in part, explain vulnerability of preterm subject to inflammation related brain injury.

**Grant Support:** NIH: 2R01HD057100-06A1: Cytokines and the blood–brain barrier in the ovine fetus.

## A65 Expression and functional characterization of SLC transporters in brain microvascular endothelial cells derived from human induced pluripotent stem cells

### Yuma Tega^1^, Toshiki Kurosawa^1^, Kei Higuchi^1^, Tomoko Yamaguchi^2^, Takashi Nakakura^1^, Tatsuki Mochizuki^1^, Hiroyuki Kusuhara^1^, Kenji Kawabata^2^, Yoshiharu Deguchi^1^

#### ^1^Teikyo University, Tokyo, Japan; ^2^National Institutes of Biomedical Innovation, Health and Nutrition, Osaka, Japan

##### **Correspondence:** Yuma Tega - tega@pharm.teikyo-u.ac.jp

*Fluids and Barriers of the CNS* 2019, **16(Suppl 1):**A65

**Objective:** Brain microvascular endothelial cells derived from human induced pluripotent stem cells (hiPS-BMECs) are thought to have great potential to facilitate CNS drug development as an innovative human BBB model. However, the function of transporters, especially SLC transporters, in the cells has not yet been fully evaluated while the information would be important for construction of a high-quality BBB model that mimics the in vivo human BBB. Therefore, the purpose of the present study was to clarify the expression and function of SLC transporters in hiPS-BMECs.

**Methods:** Human iPS cell line IMR90-4 was differentiated to brain microvascular entdothelial cells according to the method of Lippmann et al. (1). The mRNA expression levels of transporters were measured by quantitative real-time PCR (qPCR) analysis. Transport function was evaluated by the uptake and transcellular transport studies. Drugs were quantified by LC–MS/MS system or liquid scintillation counter.

**Results:** Human iPS cell line IMR90-4 was differentiated to brain microvascular entdothelial cells according to the method of Lippmann et al. (1). The mRNA expression levels of transporters were measured by quantitative real-time PCR (qPCR) analysis. Transport function was evaluated by the uptake and transcellular transport studies. Drugs were quantified by LC–MS/MS system or liquid scintillation counter.

**Conclusion:** The present study indicates that hiPS-BMECs not only form strong tight junctions but also express functionally multiple nutrient and drug transporters such as LAT1, OCTN2, MCT1, CAT1, GLAST, and H+/OC antiporter (2). Our findings should contribute to the development of high-fidelity in vitro models of the BBB.

**Grant Support:** This work was supported in part by a Grant-in-Aid for Scientific Research and by the MEXT-Supported Program.

**References**

1. Lippmann ES, Azarin SM, Kay JE, Nessler RA, Wilson HK, Al-Ahmad A, Palecek SP, Shusta EV. Derivation of blood–brain barrier endothelial cells from human pluripotent stem cells. Nat Biotechnol. 2012; 30(8):783–91.
2. Kurosawa T, Tega Y, Higuchi K, Yamaguchi T, Nakakura T, Mochizuki T, Kusuhara H, Kawabata K, Deguchi Y. Expression and functional characterization of drug transporters in brain microvascular endothelial cells derived from human induced pluripotent

## A66 Expression of glucose transporters in an immortalized rat brain endothelial cell line (RBE4) subjected to in vitro stroke conditions

### Bianca Reilly^1^, Robert Betterton^1^, Hrvoje Brzica^2^, Patrick Ronaldson^1^

#### ^1^University of Arizona, Tucson, AZ, USA; ^2^University of Zagreb, Zagreb, Croatia

##### **Correspondence:** Bianca Reilly - biancareilly@email.arizona.edu

*Fluids and Barriers of the CNS* 2019, **16(Suppl 1):**A66

**Objectives:** Glucose, the primary energy source for the mammalian brain, cannot passively diffuse across the blood–brain barrier (BBB). Therefore, brain glucose delivery is primarily accomplished via facilitated diffusion mediated by glucose transporter 1 (Glut1). Other glucose transporters that are either sodium-independent (i.e., Glut3, Glut4) or sodium-dependent (i.e., Sglt1 or Sglt2) also contribute to glucose transport at the BBB. The exact contribution of individual transporters to overall blood-to-brain glucose transport has not been determined. In ischemic stroke, there is a need for increased glucose delivery to counteract rapid oxygen depletion. This can be accomplished by upregulation of Glut1 or Glut3; however, enhanced activity of Sglts can result in increased sodium flux at the BBB and development of cerebral edema. Indeed, differential targeting of BBB sodium-independent and sodium-dependent glucose transporters is a potential strategy for treatment of ischemic stroke. The objective of this study is to examine expression of glucose transporters in brain microvascular endothelial cells under in vitro stroke conditions. This work will identify specific transporter targets that can be exploited for ischemic stroke treatment.

**Methods:** All experiments were conducted using the immortalized rat brain endothelial cell line RBE4. Cells were subjected to normoxic or hypoxic (i.e., 0% O2)/aglycemic conditions for 1 h, 2 h, or 4 h. Gene and/or protein expression of Glut-1, Glut-3, and Sglt2 was determined by quantitative PCR and western blot analysis respectively.

**Results:** Quantitative PCR and/or western blot analysis confirmed expression of glucose transporters (i.e., Glut1, Glut3, Sglt2) in RBE4 cells. Of particular note, Sglt2 protein expression in RBE4 cells was detected for the first time. Following 1 h or 2 h hypoxia/aglycemia (i.e., in vitro stroke conditions), protein expression of both Glut-1 and Glut-3 were significantly (p < 0.05) increased in RBE4 cells.

**Conclusions:** Our results provide evidence for modulation of glucose transporter expression in brain microvascular endothelial cells subjected to in vitro stroke conditions. Our ongoing research is utilizing an siRNA knockdown approach to determine the relative contribution of Glut1, Glut3, and Sglt2 to brain glucose uptake. Such studies are critical to development of novel treatment strategies for treatment of ischemic stroke.

**Grant Support:** National Institute of Neurological Diseases and Stroke (R01-NS084941); ABRC Grant #ADHS16-162406.

## A67 Fabrication and characterization of 3D-printed microvascular structures based on hydrogel as a blood–brain barrier model

### Behnam Noorani, Ekram Ahmed Chowdhury, Srikumar Krishnamoorthy, Changxue Xu, Abraham Al-Ahmad, Ulrich Bickel

#### Texas Tech University Health Sciences Center, Amarillo, TX, USA

##### **Correspondence:** Behnam Noorani - Behnam.Noorani@ttuhsc.edu

*Fluids and Barriers of the CNS* 2019, **16(Suppl 1):**A67

**Objective:** To date, various in vitro models of the Blood–Brain Barrier (BBB) such as the Transwell^®^ systems have been developed. However, these two dimensional (2D) models fail to address physiologically relevant conditions such as exposure of the endothelium to flow and shear stress, and 3D cellular organization that is vital to many cellular processes in vivo [1]. In the present study, we fabricated and characterized an in vitro 3D bio-printed microvascular structure based on Gelatin Methacryloyl (GelMA) hydrogel for the BBB model.

**Methods:** GelMA was obtained by reaction of gelatin (10% w/v) with (8% v/v) methacrylic anhydride. The 3D printing micro extrusion technique was used to create microvascular networks through Pluronic F-127 sacrificed material inside GelMA. The properties of the hydrogel such as swelling ratio, morphology study, and pore size were evaluated after the printing process. Cell viability in the channels and continuous 3D monolayer in different sizes of channels were evaluated using bEnd3 cells.

**Results:** The pore sizes and morphology of channels were evaluated by scanning electron microscopy (SEM). The SEM showed uniform porous microstructures in GelMA with 68 ± 26 µm interconnected pore sizes. The SEM images of 3D printed microvascular structures showed 3D printing microextrusion technique allowed us to generate a circular structure with great 3D complexity. 3D projections generated from z-stack confocal imaging of fluorescently stained bEnd3 cells showed formation of a confluent monolayer microchannels of different diameters. The images of attached cells in bottom, top, and wall of the channels indicated the scaffold had more than 90% cell viability.

**Conclusion:** In summary, 3D printing technique allows us to grow endothelia in a circular fashion in a biocompatible matrix, which is closer to the native environment in the microvasculature. Moreover, our hydrogel as a scaffold can support different architectures of microvascular networks besides high cell viability in long- term culture. In future we plan on evaluating the permeability across the channel and to prepare triple cultures of human induced pluripotent stem cell-derived endothelial cells with astrocytes and pericytes in close proximity, to approach a lifelike BBB 3D model.

**Grant Support:** Presidents’ Collaborative Research Initiative, Texas Tech University HSC and Texas Tech University.

**Reference**

1. van der Helm MW, van der Meer AD, Eijkel JC, van den Berg A, Segerink LI: Microfluidic organ-on-chip technology for blood–brain barrier research. Tissue Barriers. 2016; 4:e1142493

## A68 Failure of physical exercise and simvastatin to counter cerebrovascular and behavioural deficits in mice with dysfunctional brain endothelial cells

### Lianne Trigiani^1^, María Lacalle-Aurioles^1^, Miled Bourourou^1^, Amy Hynes^1^, Markus Schwaninger^2^, Edith Hamel^1^

#### ^1^Montreal Neurological Institute, Montreal, QC, Canada; ^2^Institute of Experimental and Clinical Pharmacology and Toxicology, 23562 Lubeck, Germany

##### **Correspondence:** Lianne Trigiani - lianne.trigiani@mail.mcgill.ca

*Fluids and Barriers of the CNS* 2019, **16(Suppl 1):**A68

**Objective:** It has been proposed that an early vascular insult may initiate cognitive decline, and pharmacological and lifestyle interventions have shown promise in preventing dementia. We aimed to determine whether cerebral endothelial cell function is necessary to reap neuroprotective benefits conferred by exercise or simvastatin.

**Methods:** Mice with a tamoxifen-induced selective deletion of the NF-κB essential modulator (Nemo) in brain endothelial cells were studied: Groups (n = 30/group) included Cre−/− for Nemo and three groups of Cre+/+ mice: One was untreated, one received anti-cholesterol treatment simvastatin (~ 40 mg/kg/day, drinking water), and one had 3 h nightly access to running wheels (treatments began 1 month prior to tamoxifen injection, and lasted 2 months). Following tamoxifen injections, behavioural tests were conducted, cranial windows implanted for optical imaging, and cerebrovascular reactivity was performed. Following in vivo experiments, brain tissue was prepared for immunohistochemical analysis or Western blots.

**Results:** Untreated Cre+ mice showed a tendency for spatial memory impairment in the Morris water maze, consistent with a pilot study showing significant spatial memory deficits in these mice. Neither simvastatin nor exercise showed benefits on this task. Social preference was impaired in the 3-chamber sociability test in Cre+ mice and was not countered by treatments. Similarly, cerebrovascular function was compromised in Cre+ groups regardless of treatment, exhibiting decreased whisker-evoked changes in cerebral blood flow and blood volume, and impaired endothelium-dependent vasodilation to acetylcholine and a TRPV4 channel opener, but smooth muscle cell relaxation was preserved. All Cre+ groups had significant increases in string vessel pathology in the cortex and corpus callosum, cortical astrocytosis and microgliosis, galectin-3 positive microglia in white matter, and a decreased number of mature oligodendrocytes.

**Conclusion:** Without a functional endothelium, it was not possible for exercise nor simvastatin to exert their beneficial effects, indicating that the endothelium may be central to both cerebrovascular and cognitive function. These findings strongly suggest that initial damage to the cerebral endothelium may be key to initiating pathologies associated with dementia that lead to cognitive decline.

**Grant Support:** Supported by grants (EH) from the Canadian Institutes of Health Research (CIHR-MOP-126001), Canadian Consortiu.

## A69 Fatty acid oxidation occurs under normal conditions in the mammalian nervous system

### Cory White, Jieun Lee, Joseph Choi, Tiffany Chu, Susana Scafidi, Michael Wolfgang

#### Johns Hopkins School of Medicine, Baltimore, MD, USA

##### **Correspondence:** Cory White - cwhite84@jhmi.edu

*Fluids and Barriers of the CNS* 2019, **16(Suppl 1):**A69

**Objective:** Astrocytes are positioned to serve as mediators between neurons and endothelial cells of cerebral blood vessels. Nutrients supplied to astrocytes by cerebral arterioles can be metabolized outright in astrocytes or sent to neurons for use. Glucose (and ketones during fasting or starvation) is the most commonly used energy substrate in brain. However, fatty acids (FAs) are capable of crossing the blood–brain barrier through both passive diffusion and facilitated via the use of transporter proteins. Previous findings suggest mitochondrial long-chain fatty acid β-oxidation can functionally occur in the brain when supplied with exogenous FAs1-3. However, the capacity and possible functional role(s) for oxidation of endogenous long-chain fatty acids in the mammalian brain is unknown.

**Methods:** To understand the neurochemical capacity for long-chain fatty acid β-oxidation in the mammalian nervous system, we generated mice with a pan-brain-specific loss of carnitine palmitoyltransferase 2 (CPT2B−/−), an obligate step in mitochondrial long-chain fatty acid β-oxidation. We studied the impact of fatty acid oxidation (FAO) loss in the brain using lipid oxidation and other biochemical experiments, and unbiased and targeted metabolomics.

**Results:** Loss of CNS FAO did not result in neuroanatomical changes nor systemic differences in metabolism. We demonstrate that the CNS oxidizes a substantial quantity of long-chain FAs by utilizing primary astrocytes in vitro and unbiased and targeted metabolomics in vivo. Loss of CNS FAO leads to robust accumulations in long-chain acylcarnitines in brain irrespective of diet. Comparison of arterial and venous acylcarnitines suggests that the brain is oxidizing FAs with no spillover to peripheral tissues and without impacting systemic concentrations of acylcarnitines.

**Conclusion:** Together, these results demonstrate a basic bioenergetic capacity for endogenous long-chain FAO in brain. Loss of brain FAO leads to accumulations in long-chain acylcarnitines amongst other changes in the brain metabolome. FAO in the brain occurs with minimal influence from or impact on peripheral tissues. Regardless of the functional role of brain FAO, whether for turnover of membrane lipid or use as an energetic substrate, FAO exists in the mammalian brain under normal conditions.

**Grant Support:** NIH R01NS07224107, NIH 1F31NS102151-01A1.

**References**

1. Jernberg JN, Bowman CE, Wolfgang MJ, Scafidi S. Developmental regulation and localization of carnitine palmitoyltransferases (CPTs) in rat brain. J Neurochem. 2017; 142:407–19. 10.1111/jnc.14072.
2. Auestad N, Korsak RA, Morrow JW, Edmond J. Fatty acid oxidation and ketogenesis by astrocytes in primary culture. J Neurochem. 1991; 56:1376–86.
3. Vignais PM, Gallagher CH, Zabin I. Activation and oxidation of long chain fatty acids by rat brain. J Neurochem. 1958; 2:283–7. doi:10.1111/j.1471-4159.1958.tb12375.x.

## A70 Flvcr2 is required for normal brain angiogenesis, but not blood–brain barrier formation in mice

### Nicolas Santander, Carlos Lizama, Eman Meky, Gabriel McKinsey, Thomas Arnold

#### University of California San Francisco, San Francisco, CA, USA

##### **Correspondence:** Nicolas Santander - nicolas.santandergrez@ucsf.edu

*Fluids and Barriers of the CNS* 2019, **16(Suppl 1):**A70

**Objective:** Whether brain angiogenesis and blood–brain barrier (BBB) formation are coupled remains unresolved. BBB breakdown and angiogenic defects are observed in genetic defects, but no mutant model has isolated angiogenesis abnormalities. Flvcr2 mutations are associated with cerebral vascular malformations and hydranencephaly in humans, however the molecular pathogenesis is unknown. Here, we assessed the role of this gene in brain angiogenesis and blood–brain barrier (BBB) homeostasis in mice.

**Methods:** We generated mouse mutants in the Flvcr2 locus that allow Cre-mediated gene inactivation and GFP expression under the control of this locus. We used histology and flow cytometry to determine the expression domain of the Flvcr2 locus. We employed immunofluorescence to assess angiogenesis in embryonic brain at E12.5, E14.5, and E18.5. We also evaluated brain angiogenesis after endothelial cell-specific deletion of Flvcr2. Furthermore, we examined pathological consequences in mutant brains, such as hypoxia (pimonidazole) and cell death (cleaved caspase 3) Finally, we tested BBB integrity with small-molecule tracers and by the presence of hemorrhage.

**Results:** We observed GFP expression, indicating expression of the Flvcr2 locus, specifically in brain endothelial cells in embryos and half of CD31+ cells were also GFP+. In adults, GFP was most prominently detected in brain endothelium, but also neurons, glia, alveolar macrophages, and intestinal epithelium. Global deletion of Flvcr2 was associated with reduced filopodia at E12.5 and vascular coverage at E14.5. Interestingly, the ganglionic eminence at E14.5 and the entire periventricular area at E18.5 were devoid of vasculature. Specific deletion of Flvcr2 in endothelial cells with Tie2-Cre or Cdh5-CreERT2 led to a similar phenotype at E14.5. Avascular areas showed strong pimonidazole reactivity, indicating the presence of hypoxia. At E18.5, Flvcr2 deletion was associated with widespread immunoreactivity for cCasp3 in the brain, suggesting global cell death. However, we observed no hemorrhages in the embryonic brain upon constitutive Flvcr2 deletion. Likewise, genetic inactivation post-natally did not lead to leakage of small-molecule tracers into brain parenchyma.

**Conclusion:** Flvcr2 is required in brain endothelium for normal angiogenesis and brain homeostasis, but not for BBB formation or maintenance. Understanding Flvcr2 function will discriminate angiogenic and BBB pathways, allowing attribution of neurologic sequellae to these defects.

**Reference**

1. Meyer E, et al. Mutations in FLVCR2 are associated with proliferative vasculopathy and hydranencephaly-hydrocephaly syndrome (Fowler syndrome). Am J Hum Genet. 2010; 12;86(3):471-8.

## A71 Foxf2 conditional knockout mice, a potential model for stroke and cerebral small vessel disease

### Azadeh Reyahi

#### Gothenburg University, Gothenburg, Sweden

##### **Correspondence:** Azadeh Reyahi - azadeh.reyahi@gu.se

*Fluids and Barriers of the CNS* 2019, **16(Suppl 1):**A71

Cerebral small vessel disease (CSVD) is a clinical finding detectable by imaging methods such as MRI. It should be emphasized that the abnormalities attributed to CSVD in the clinic such as lacunar infarcts, white matter lesions, large hemorrhages, and micro-bleeds are not the primary lesions of CSVD, but rather its consequence on the brain parenchyma. CSVD is not only the leading cause of cognitive impairment and dementia in the elderly, but also a major predisposition factor for stroke.

As a preventive measure for stroke, dementia and cognitive impairment, treatment of

CSVD is urgently needed. Unfortunately, the lack of an animal model for CSVD hinders the research progress in this field. A good animal model is needed to study the molecular mechanisms behind the pathogenesis and to try and assess the effectiveness of new preventive and treatment modalities.

The Foxf2 conditional knockout (Foxf2 cko) displays impairment of the BBB, white matter lesions, micro-bleeds, reactive gliosis, infarction and susceptibility to stroke. It represents the best candidate to a CSVD and stroke animal model yet described. This is further underscored by the fact that according to a large body of evidence, FOXF2 is the first discovered risk locus for human CSVD and the genetic locus with the strongest association with stroke risk in the general population.

In order to establish the Foxf2 cko mice as a clinical animal model, a comprehensive characterization of the CNS with focus on the molecular, cellular and physiological mechanism of CSVD and stroke has been performed. We have developed a new method for isolating intact microvasculature with high yield, purity and viability from the brain. By comparing the transcriptome of Foxf2 null mutant and control, we tried to understand the primary effect of Foxf2 on the global gene expression in the pericytes and endothelial cells. In Foxf2 cko, we detected elevated levels of cerebral blood flow, permeability of the blood brain barrier followed by leak of fibrinogen, hypoxia, apoptosis, intracranial hemorrhages, neuro-inflammation and stroke.

**Grant Support:** Swedish Research Council (VR).

**References**

1. Reyahi A, Nik AM, Ghiami M, Gritli-Linde A, Ponten F, Johansson BR, Carlsson P. Foxf2 is required for brain pericyte differentiation and development and maintenance of the blood–brain barrier. Dev Cell. 2015; 34(1):19–32.
2. Chauhan G, Arnold C, Chu A, Fornage M, Reyahi A, Bis J, Havulinna A. (equal contribution first authors) …additional authors excluded for brevity… joint senior authors: Launer L, Ikram M, Carlsson P, Chasman D, Childs S, Longstre

## A72 High glucose induced changes in astrocyte released factors alter cerebral blood flow

### Debebe Gebremedhin, David R. Harder

#### Medical College of Wisconsin, Milwaukee, WI, USA

##### **Correspondence:** Debebe Gebremedhin - dgebreme@mcw.edu

*Fluids and Barriers of the CNS* 2019, **16(Suppl 1):**A72

**Background:** Brain function is virtually dependent on continuous supply of glucose and molecular oxygen for production of energy for cell function and memory. In addition to its use as a substrate for oxidative respiration and energy production, glucose can be converted by lactate dehydrogenase to lactate for preferred energy source in hypoxic condition. High blood glucose level is known to be associated with generalized poor outcomes in the cerebral circulation including impaired cell proliferation, altered cerebral vascular reactivity and memory deficit. Astrocytes are intermediary cell types in the brain and play a crucial role in homeostasis of neuronal function and cerebral blood flow by producing and releasing factors regulating the brain microenvironment. However, the effect of a high glucose environment on the dynamics of astrocyte secreted signaling factors in regulating the brain microenvironment and on cerebral blood flow has not been completely understood. In this study we investigated effect of high glucose on astrocyte produced and released factors on brain microenvironment and regulation of cerebral blood flow.

**Objective:** To examine influence of acute changes in environmental glucose on astrocyte produced signaling factors and their role in associated changes in brain microenvironment and regulation of cerebral blood flow (CBF).

**Methods:** Neonatal rat astrocytes in culture were exposed to changes in environmental glucose (normal, high) over three passages. Astrocyte whole cell lysates were used to determine changes in expression of levels of different signaling mediators using western blot analysis, and correlative in vivo CBF using Laser Doppler flowmetry.

**Results:** High glucose environment did not influence morphology and population of cultured astrocytes. High glucose environment increased expression of insulin receptor, PKC-α and PKC-γ, cytosolic Ca2+ level, and resulted in reduced expression of connexin 43, P-Akt and reduced CBF in response to increased neuronal activation.

**Conclusion:** High glucose environment resulted in detectable changes in expression of astrocyte released signaling factors and a reduction in brain functional hyperemic blood flow response. These findings indicate possible adaptive changes in astrocytes function that could be targeted for management of alterations in brain microenvironment and cerebral blood flow under hyperglycemic conditions.

**Grant Support:** Funding from NIH (NHLBI) R01 HL033833 and HL105997.

## A73 HIV Antiretroviral Therapy Regulates GLUT-1 Localization at the BBB

### Dionna Williams, Lisa Fridman

#### Johns Hopkins University, Baltimore, MD, USA

##### **Correspondence:** Dionna Williams - dwill201@jhmi.edu

*Fluids and Barriers of the CNS* 2019, **16(Suppl 1):**A73

**Objective:** While antiretroviral therapy (ART) reduces plasma viral loads to near undetectable levels, it does not always quell virus to the same extent in other areas of the body. This is particularly true for the brain, which is separated from the periphery by the BBB. As a result, ongoing HIV replication occurs within the brain, establishing it as a viral reservoir. A more complete understanding of the effects of ART on BBB function will be integral to the elimination of the brain as a viral reservoir and HIV-associated neurologic disease.

**Methods:** Primary human brain microvascular endothelial cells (BMVEC) were utilized as an in vitro model of the BBB. ART was added to the BMVEC for 24 h, after which time GLUT-1 mRNA, total protein levels, and cell surface expression were evaluated.

**Results:** Tenofovir and emtricitabine significantly decreased GLUT-1 mRNA levels; in contrast, dolutegravir significantly increased GLUT-1 mRNA. Interestingly, these changes in mRNA were not reflected at the total protein levels, as none of the ART drugs altered total GLUT-1 levels. However, evaluation of GLUT-1 on the cell surface, where it is functionally active to facilitate glucose transport, demonstrated findings consistent with the gene expression changes: tenofovir and emtricitabine decreased GLUT-1 localization to the plasma membrane, while dolutegravir increased surface localization.

**Conclusion:** While ART was designed to impact critical steps of the HIV life cycle, our findings indicate that they may also impact normal physiological processes. The off-target effects of ART on GLUT-1 indicate that the treatment regimen for HIV may have major consequences for normal BBB and brain function. As glucose is the preferred nutrient source for the brain, alterations in GLUT-1 mediated by ART indicates that the treatments for HIV may compound the stresses on BBB and brain function that occur as a result of infection.

**Grant Support:** K99DA044838.

## A74 How insulin interact with the brain-blood barrier: an in situ cerebral perfusion in an animal model of Alzheimer’s disease

### Manon Leclerc^1^, Vicky Caron^1^, Jessica Virgili^1^, Vincent Emond^1^, Philippe Bourassa^1^, Frédéric Calon^2^

#### ^1^Université Laval, Québec city, QC, Canada; ^2^Centre de recherche du CHU de Québec, Canada

##### **Correspondence:** Manon Leclerc - manon.leclerc.4@ulaval.ca

*Fluids and Barriers of the CNS* 2019, **16(Suppl 1):**A75

Although insulin is produced in the periphery, it is receiving growing attention for its impact on the brain. Whereas there is a wealth of literature on defective brain insulin signaling, particularly in type-2 diabetes (T2D) but also in Alzheimer’s disease (AD), most of the studies do not consider that insulin must first interact with the blood–brain barrier (BBB) before reaching the central nervous system. The insulin receptor (IR) located on the BBB binds circulating insulin and has been proposed to either act as a receptor triggering cell-signaling pathways or, as a transporter to ferry insulin into the brain parenchyma.

**Objective:** We aimed at providing a better understanding of the mechanisms underlying transport and cell-signaling of insulin at the BBB, in relation to T2D and AD.

**Methods:** In situ cerebral perfusion was used to quantify the transport of [125I] insulin through the BBB of 3xTg-AD (modeling AD neuropathology) and non-transgenic mice (Non-Tg). A high-fat diet (HFD) was used to induce obesity and a T2D-like phenotype. IR activation was investigated using Western blots of p-IR/IR performed with microvessel-enriched fractions from insulin-treated mice.

**Results:** The brain uptake of insulin-I125 (Kin) remained low in NonTg (0.021 ± 0.005 µl g^−1^ s^−1^) and 3xTg-AD mice (0.012 ± 0.005 µl g^−1^ s^−1^), but was increased in animals exposed to HFD (+ 324% for 3xTg-AD animals). Such a low rate of transport but consistent with previous studies. No change in permeability was detected with co-perfused [14C]-sucrose, a marker of brain vasculature volume. Coperfusion with the IR antagonist S961 did not alter the brain uptake of insulin-I125, suggesting that the IR is not involved in its transport. Western blots experiments confirmed that the IR was activated after an acute insulin injection, an effect found to be blunted in 3xTg-AD mice under HFD.

**Conclusion:** Our results indicate that (i) the brain uptake of insulin is low and independent of the IR but increased following a HFD; (ii) AD neuropathology and HFD interfere with IR signaling within BBB cells. The complex interaction of insulin with the BBB must be defined to evaluate its potential role in the treatment of AD.

## A75 Human blood–brain barrier chip

### Nur Mustafaoglu^1^, Tae-Eun Park^1^, Anna Herland^1^, Robert Mannix^1^, Rachelle Prantil-Baun^1^, Alexander Watters^1^, Eric Shusta^2^, Donald E. Ingber^1^

#### ^1^Wyss Institute for Biologically Inspired Engineering at Harvard University, Boston, MA, USA; ^2^Department of Chemical and Biological Engineering, University of Wisconsin-Madison, Madison, WI, USA

##### **Correspondence:** Nur Mustafaoglu - nur.mustafaoglu@wyss.harvard.edu

*Fluids and Barriers of the CNS* 2019, **16(Suppl 1):**A76

**Objective:** The highly specialized human brain microvascular endothelium forms a selective blood–brain barrier (BBB) with adjacent pericytes and astrocytes that restricts delivery of many pharmaceuticals and therapeutic antibodies to the central nervous system.

**Methods and results:** Here, we describe an in vitro microfluidic ‘organ-on-a-chip’ (Organ Chip) model of the BBB lined by induced pluripotent stem cell-derived human brain microvascular endothelium (iPS-BMVEC) interfaced with primary human brain astrocytes and pericytes that recapitulates the high level of barrier function of the in vivo human BBB for at least one week in culture. The endothelium expresses high levels of tight junction proteins, multiple functional efflux pumps, and displays selective transcytosis of peptides and anti-transferrin receptor antibodies previously observed in vivo. This increased level of barrier functionality was accomplished using a developmentally-inspired induction protocol that includes a period of differentiation under hypoxic conditions.

**Conclusion:** This enhanced BBB Chip may therefore represent a new in vitro tool for development and validation of delivery systems that transport drugs and therapeutic antibodies across the human BBB.

**Grant Support:** DARPA (W911NF-12-2-0036), AstraZeneca, Wyss Institute for Biologically Inspired Engineering.

**References**

1. Herland A, et al. Distinct contributions of astrocytes and pericytes to neuroinflammation identified in a 3D human blood–brain barrier on a chip. PLoS ONE. 2016; 11:e0150360.
2. Lippmann ES, Al-Ahmad A, Azarin SM, Palecek SP, Shusta EV. A retinoic acid-enhanced, multicellular human blood–brain barrier model derived from stem cell sources. Sci Rep. 2014; 4:4160.
3. Maoz BM, et al. A linked organ-on-chip model of the human neurovascular unit reveals the metabolic coupling of endothelial and neuronal cells. Nat Biotechnol. 2018; 36:865–74.

## A76 Human blood–brain barrier-on-a-chip with 3D astrocytic network for studying CNS delivery systems

### Song Ih Ahn^1^, Hyun-Ji Park^1^, Jinhwan Kim^1^, Yoshitaka Sei^1^, Jeongmoon J. Choi^1^, Yujung Ryu^1^, Young C. Jang^1^, Allan I. Levey^2^, YongTae Kim^1^

#### ^1^Georgia Institute of Technology, Atlanta, GA, USA; ^2^Emory University, Atlanta, GA, USA

##### **Correspondence:** Song Ih Ahn - songihahn@gatech.edu

*Fluids and Barriers of the CNS* 2019, **16(Suppl 1):**A77

**Objective:** The central nervous system (CNS) has a specialized vascular barrier, the blood–brain barrier (BBB), which possesses a highly selective barrier function that restricts the permeability of drugs, leading to a high failure rate in the development of CNS therapeutics. However, it remains difficult with existing in vivo models to conduct mechanistic studies of the barrier function and interactions with drugs at molecular and cellular levels. This challenge highlights the importance of the development of in vitro models that mimic the physiological structure and function of the BBB. Despite various efforts, physiologically relevant in vitro human BBB models capable of highly precise, quantitative analysis of drug delivery remain to be developed. In this study, we present a human BBB-on-a-chip designed to create a 3D astrocytic network with reduced reactive gliosis and polarized aquaporin-4, critical features of the BBB that has yet to be demonstrated. Our model will provide a reliable tool for studying the penetrance of CNS drugs across the BBB.

**Methods:** Primary human brain pericytes and astrocytes were 3D cultured in the abluminal channel. Human brain endothelium in the luminal channel was exposed to a physiological shear stress. Microfluidic technology was used to synthesize an engineered high-density lipoprotein-mimetic nanoparticle with apolipoprotein-A1 (eHNP-A1). The nanoparticle distributions were measured by high-precision sampling, confocal microscopy, and flow cytometry.

**Results:** We have developed a human BBB-on-a-chip that reconstitutes a physiological network of astrocytes with reduced reactive gliosis, polarized expression of aquaporin-4, highly specialized brain endothelial cells with increased expressions of junctional and transporter proteins, and a significantly decreased permeability of the BBB. With this BBB model, we successfully mapped 3D distributions of eHNP-A1 in the luminal and abluminal regions and quantitatively examined differential interaction and uptake of endothelial cells and astrocytes while inhibiting endothelial receptors (e.g. SR-B1) that mediate transcytosis of eHNP-A1 across the BBB.

**Conclusion:** We microengineered a human BBB-on-a-chip that successfully recapitulated the key structure and function of the BBB and demonstrated the transport feature of biomimetic nanoparticles. We believe that the model can be used to screen drug candidates by investigating the mechanism by which they get into the brain.

**Grant Support:** This work was supported by the NIH NIA 1R21AG056781, NIH Director’s New Innovator Award 1DP2HL142050, and NSF.

## A77 Human gut bacterial sequence in Alzheimer’s and associated diseases: link to fecal metabolites in health and disease

### Elena Paley

#### Expert Biomed, Inc. and Stop Alzheimers Corp, Homestead, FL, USA

##### **Correspondence:** Elena Paley - elena_paley@bellsouth.net

*Fluids and Barriers of the CNS* 2019, **16(Suppl 1):**A78

**Objective:** Towards understanding of microbiome role in Alzheimer’s disease: the gut brain axis in health and disease

**Methods:** Tryptophan pathway was targeted in human gut PCR analysis of Alzheimer’s disease (AD) patients and controls.

**Results:** The human gut bacterial Na(+)-transporting NADH:ubiquinone reductase (NQR) sequence (ADAS) was found to be associated with AD. ADAS is further characterized in control and diseased individuals and in bacteria isolated from human fecal samples. Tryptophan and NQR substrate ubiquinone have common precursor chorismate in microbial shikimate pathway. Tryptophan-derived tryptamine presents in human diet and gut microbiome. Tryptamine inhibits tryptophanyl-tRNA synthetase (TrpRS) with consequent neurodegeneration in cell and animal models. TrpRS inhibition causes protein biosynthesis impairment similar to that revealed in AD. Analysis of gut microbiome reveals 89–100% ADAS nucleotide identity (or 97–100% protein sequence identity) in NCBI databases. ADAS prevalence was estimated in different human populations. Metabolomics revealed that tryptamine; chorismate precursor quinate; chorismate product 4-hydroxybenzoate (ubiquinone precursor) are significantly higher while tryptophan-containing dipeptides lower due to tRNA aminoacylation deficiency in human population with high ADAS prevalence compared to population with low or no ADAS. This confirms that gut microbial tryptamine overproduction correlates with ADAS occurrence. Antibiotic and diet additives induce ADAS and tryptamine. Mitogenic and cytotoxic tryptamine actions are responsible for microbial and human cell death, gut dysbiosis and consequent disruption of host-microbe homeostasis. Present analysis of 2754 participants (ADAS-comprising human sample size) from 24 human gut metagenomics studies of four continents including North America, Europe, Australia and Asia revealed a higher ADAS prevalence in cell death-associated diseases and conditions compared to controls (in press 2019).

**Conclusion:** The new-developed non-invasive stool laboratory test can be used in clinical trials and for examining general population

**Grant Support:** Funded by private company.

**References**

1. Towards an integrative understanding of tRNA aminoacylation-diet-host-gut microbiome interactions in neurodegeneration.
2. Paley EL, Perry G.
3. Nutrients. 2018 Mar 26;10(4). pii: E410. 10.3390/nu10040410.
4. Geographical Distribution and Diversity of Gut Microbial NADH:Ubiquinone Oxidoreductase Sequence Associated with Alzheimer’s Disease.
5. Paley EL, Merkulova-Rainon T, Faynboym A, Shestopalov VI, Aksenoff I.
6. J Alzheimers Dis. 2018;61(4):1531–1540. doi: 10.323

## A78 Identification of cyclic peptide facilitating permeability of M13 phage across the blood–brain barrier

### Shunsuke Yamaguchi, Shingo Ito, Takeshi Masuda, Sumio Ohtsuki

#### Kumamoto University, Kumamoto, Japan

##### **Correspondence:** Shunsuke Yamaguchi - 163y3106@st.kumamoto-u.ac.jp

*Fluids and Barriers of the CNS* 2019, **16(Suppl 1):**A79

**Objective:** Macromolecular drugs such as antibodies and nucleic acids are promising drugs for central nervous system (CNS) diseases. Therefore, blood–brain barrier (BBB)-permeable carriers are required for the development of CNS-acting macromolecular drugs. The purpose of the present study was to identify the cyclic peptide facilitating BBB permeability of M13 phage, which is larger than macromolecular drugs and nanoparticles, by phage display screening.

**Methods:** To identify the BBB-permeable cyclic peptides, Ph.D.™-C7C Phage Display Peptide Library (New England Biolabs) was screened for three times by transcellular permeability assay with hCMEC/D3 cells as human BBB model.

**Results and Discussion:** As a result of phage library screening, cyclic peptide X was identified. The per-eating amounts of cyclic peptide X displaying phage (X-phage) were greater by 3.3-fold than those of peptide-less phage (control phage) at 30 min by hCMEC/D3 permeability assay. The BBB permeability was also assessed with in vitro monkey and rat BBB co-cultured models using primary brain microvascular endothelial cells, pericytes and astrocytes. The permeating amounts of X-phage were greater than that of control phage by 7.6 and 28-fold in monkey and rat BBB models, respectively, at 30 min. The X-phage was internalized into hCMEC/D3 cells, and the internalization was inhibited to 33% and 26% by synthesized cyclic peptide X and macropinocytosis inhibitor (EIPA), respectively. The synthesized cyclic peptide X did not affect either the cell viability or tight-junction integrity of hCMEC/D3 cells. These results suggest that cyclic peptide X facilitates transcellular permeation of phages through brain microvascular endothelial cells by macropinocytosis, but not by enhancing paracellular diffusion. Then, in vivo BBB permeability of X-phage was examined by intravenous administration in mouse. The brain-to-plasma ratio of X-phage was greater by 12-fold than that of control phage at 60 min after the administration. X-phages were detected around brain capillaries in cerebral cortex and hippocampus. These results indicate that cyclic peptide X facilitates delivery of phages to the brain parenchyma across the BBB.

**Conclusion:** We identified cyclic peptide X that facilitates transcellular permeation of phage across the BBB in vitro and in vivo.

## A79 Identifying anatomical routes and molecular mechanisms mediating human CD4+ effector/memory T cell entry into the central nervous system by employing human in vitro brain barrier models

### Hideaki Nishihara^1^, Sasha Soldati^1^, Eric Shusta^2^, Renaud Du Pasquier^3^, Federica Sallusto^4^, Horst Schroten^5^, Hiroshi Ishikawa^6^, Fabien Gosselet^7^, Britta Engelhardt^1^

#### ^1^Theodor Kocher Institute, University of Bern, Bern, BE, Switzerland; ^2^Department of Chemical and Biological Engineering, University of Wisconsin-Madison, WI, USA; ^3^Laboratory of Neuroimmunology, University of Lausanne, Lausanne, Switzerland; ^4^Institute for Research in Biomedicine, Università della Svizzera italiana, Bellinzona, Bellinzona, Switzerland; ^5^Department of Pediatrics, Pediatric Infectious Diseases, Medical Faculty Mannheim, Heidelberg University, Mannheim, Germany; ^6^Laboratory of Clinical Regenerative Medicine, Department of Neurosurgery, Faculty of Medicine, University of Tsukuba, 1-1-1Tennodai, Tsukuba, Ibaraki 305-8575, Japan; ^7^Université Artois, Blood–brain barrier laboratory (LBHE), Lens, France

##### **Correspondence:** Hideaki Nishihara - hideaki.nishihara@tki.unibe.ch

*Fluids and Barriers of the CNS* 2019, **16(Suppl 1):**A80

**Introduction:** Blood–brain barrier (BBB) breakdown followed by infiltration of inflammatory cells into the central nervous system (CNS) are key early steps in the pathogenesis of multiple sclerosis (MS). The cellular and molecular mechanisms mediating immune cell entry into the CNS have largely been studied in experimental autoimmune encephalomyelitis (EAE), an animal model of MS. However, EAE does not mimic the full picture of MS neuropathology underscoring the need for meaningful studies with human cells. Recent developments have paved the way to establish human stem cell derived in vitro BBB models. Combined with the availability of a human papilloma cell line derived blood-cerebrospinal fluid barrier (BCSFB) model this allows to directly compare the contribution of the brain barriers to MS pathogenesis in vitro.

**Objective:** Identifying the anatomical route, as well as cellular and molecular mechanisms mediating the migration of different effector/memory CD4 + T cell subsets into the CNS.

**Methods:** Employing a human stem cell derived BBB model and a human papilloma cell line derived BCSFB model, we have compared the migration behavior of the different effector/memory Th subsets (Th1, Th1*, Th2, Th17) sorted from the blood of healthy donors across human in vitro BBB or BCSFB models in the presence or absence of inflammatory stimuli.

**Results:** Under non-inflammatory conditions Th1* cells and next Th1 cells crossed the BBB in higher numbers, when compared to Th 2 and Th17 cells. Under inflammatory conditions the migration of all Th subsets was comparable. Investigating the migration of the Th subsets from the same donor across the BCSFB model demonstrated that migration of Th cells across the BCSFB is much lower when compared to the BBB. Interestingly, Th17 cells crossed the BCSFB in higher numbers when compared to the other Th subsets under both, unstimulated and inflamed conditions.

**Conclusion:** These observations underscore that different Th subsets may use different anatomical routes to enter the CNS during immune surveillance and neuroinflammation. We have begun to establish in vitro BBB models from inducible pluripotent stem cells (iPSCs) of MS patients to explore the impact of MS patients specific brain barrier alterations in directing T cell migration into the CNS.

**Grant Support:** ECTRIMS fellowship, the Bangerter-Rhyner Foundation, the Uehara Memorial Foundation.

## A80 Immuno-metabolic ageing effect on cerebrovascular physiopathology

### Egle Juliana Solito

#### Queen Mary University London (UK), London, UK

##### **Correspondence:** Egle Juliana Solito - e.solito@qmul.ac.uk

*Fluids and Barriers of the CNS* 2019, **16(Suppl 1):**A81

**Objective:** Aging is a significant risk factor for neurovascular diseases and there exist considerable evidence for altered vascular functions with advanced age. Aging alters the ability of the brain to respond to injury. The immune system has an impact on brain function and as we get older the immune response become weaker making it harder to see and coupe with infections. The objectives of our study were: (1) to determine the effects of physiological ageing on the essential components of the BBB named tight and adherent junctions structure and function as well as the metabolic response/demand, in a mouse animal model; (2) to identify molecules or pathways of ageing responsible for the damage of the BBB in a human in vitro system.

**Methods:** BBB integrity was measured in C57BL/6 mice at 3, 6, 18 and 26 months of age using labelled paracellular permeability tracers (Evans blue and/or different molecular sizes of fluorescein isothiocyanate (FITC) dextran)1. Tight Junction named Occludin, Claudin, ZO1 and adherens junction such as VeCadherin as well as and Glut-1 receptor were assessed by immunohistochemistry and western blot. Isolated primary endothelial cells were also assessed for their metabolic function named glycolysis, oxidative respiration, TMRE, ROS, Mitotracker. For the human studies primary brain microvascular cells (Cell Science) or hCMEC/D33 cells were incubated with PBMC from healthy young/old donors in autologous sera or pathological conditions (diabetes, cardiovascular, infections) and metabolic measures (described above) were assessed.

**Results:** BBB leakage was reported in normal ageing mice with strong sex dimorphic correlation in fertile age3. Proinflammatory circulating factors (e.g. cytokines and chemokines mainly) as well impaired migratory ability of PBMC are responsible for the metabolic alteration of the endothelium of the BBB and this damage induces cell death. Spatial–temporal microglia activation may contribute to barrier damage. Anti-inflammatory endogenous molecules (resolvins-meresins-AnnexinA1) are down modulated during ageing however their use as pharmacological tool may improve barrier function and slow down the BBB ageing.

**Conclusion:** Endogenous molecules may be exploited for the repair of the age related immuno-metabolic damage by promoting resolution of inflammation without compromising the immune response.

**Grant Support:** FISM (Fondazione Italiana Sclerosi Multipla cod 2014/R/21) and British Hearth Foundation (BHF).

**References**

1. Cristante E, et al. Feature Article: Identification of an essential endogenous regulator of blood–brain barrier integrity, and its pathological and therapeutic implications. Proc Natl Acad Sci USA. 2013; 110:832–41.
2. Weksler BB, et al. Blood–brain barrier-specific properties of a human adult brain endothelial cell line. Faseb J. 2005; 19:1872–4
3. Maggioli E, et al. Estrogen protects the blood–brain barrier from inflammation-induced disruption and increased lymphocyte trafficking. Brain Behav Immunol. 2016;51:212–222.

## A81 Impaired cerebrovascular reactivity to CO2 is linked to altered behavior, respiration and metabolism

### Jan Wenzel, Marius Richter, Carla Bettoni, Rentsenkhand Natsagdorj, Josefine Brands, Gianna Huber, Carsten A. Wagner, Markus Schwaninger

#### University of Lübeck, Lübeck, Germany

##### **Correspondence:** Jan Wenzel - jan.wenzel@pharma.uni-luebeck.de

*Fluids and Barriers of the CNS* 2019, **16(Suppl 1):**A82

**Objective:** Carbon dioxide (CO2) and protons (H+) strongly enhance cerebral perfusion indicating normal cerebrovascular reactivity. This phenomenon has often been observed, but its functional relevance is still unclear. Impaired cerebrovascular reactivity to CO2 is a key diagnostic feature of endothelial dysfunction that occurs in the metabolic syndrome and in several vascular diseases. The aim of the study was to investigate the consequences of an impaired cerebrovascular reactivity on CO_2_-related brain functions.

**Methods:** We used several in vitro and in vivo models to investigate the effect of CO_2_ on endothelial cells of the brain and on the whole organism. To identify endothelial pathways we used mouse models that lack specific G protein-coupled receptors or the Gαq/11 signaling pathway specifically in brain endothelial cells.

**Results:** Here, we found that GPR4, an endothelial receptor for H+, and endothelial Gαq/11-dependent signaling mediated the CO_2_/H+ effect on cerebrovascular reactivity. While CO_2_/H+-induced Gαq/11 signaling constricted vessels in the retrotrapezoid nucleus, it had a dilative effect in other brain areas explaining why loss of CO_2_/H+ cerebrovascular reactivity in mice differentially modulated CO_2_ effects: it reduced respiration but aggravated behavioral and metabolic responses to CO_2_. Even at normal CO_2_ concentrations mice with impaired cerebrovascular reactivity were more anxious and showed metabolic changes.

**Conclusion:** In this study we address the mechanisms by which CO_2_/H+ is sensed by the brain vasculature and how CO_2_/H+-dependent CBF regulation affects behavior, respiration, and metabolism. We demonstrate a hitherto unknown role of brain endothelial cells in CO_2_-induced hyperemia and show that a loss of this cerebrovascular reactivity uncovers several, partially unrecognized effects of CO_2_ on the CNS. Interestingly, the impaired CO_2_ reactivity is associated with dysfunctions in fear, breathing and metabolism, already at a basal state. Our data suggest that endothelial dysfunction in the brain might contribute to the pathogenesis of the metabolic syndrome, anxiety disorders, and other diseases.

## A82 Impaired innate immune response of peripheral blood leukocytes of Alzheimer’s Disease patients correlates with the clinical severity of the disease—potential use of proline-rich polypeptide complex (PRP) from bovine colostrum to correct an immunologica

### Marta Sochocka^1^, Jerzy Leszek^2^, Michał Ochnik^3^, Maciej Sobczyński^4^

#### ^1^Hirszfeld Institute of Immunology and Experimental Therapy, Polish Academy of Sciences, Wroclaw, Poland, Wroclaw, Poland; ^2^Department of Psychiatry, Wroclaw Medical University, Wroclaw, Poland; ^3^Laboratory of Virology, Hirszfeld Institute of Immunology and Experimental Therapy, Polish Academy of Sciences, Wroclaw, Poland; ^4^Department of Genomics, Faculty of Biotechnology, University of Wroclaw, Wroclaw, Poland

##### **Correspondence:** Marta Sochocka - mars@iitd.pan.wroc.pl

*Fluids and Barriers of the CNS* 2019, **16(Suppl 1):**A83

**Objective:** Alzheimer’s disease (AD) is the most common type of dementia, that affects millions of people around the world. Age is one of the most important non-modifiable risk factor of AD, thus the disease primarily affects the elderly. During ageing an innate immune system an inflammatory response are dysregulated, which may lead to an exert of pro-inflammatory milieu in humans. The consequences of failure in innate immune response has potential implications for age-associated chronic inflammatory conditions, including AD.

**Methods:** In the current study we analyzed two of innate immune mechanisms, peripheral blood leukocytes (PBLs) resistance to viral infection ex vivo along with determination of the cytokine profile (TNF-α, IFN-γ, IL-1β, IL-10) produced by uninfected and VSV (Vesicular stomatitis virus)-infected PBLs obtained from AD patients.

**Results:** Patients with AD are characterized by a reduced level or deficiency in innate immunity of PBLs. This level is correlated with the severity of the disease. Four weeks of proline-rich polypeptide complex (PRP) treatment (120 μg of PRP/day) resulted in an increase of innate immunity of PBLs of AD patients especially among those with serious AD. PBLs of patients with AD characterized with very good innate immunity produce large amounts of pro-inflammatory cytokines TNF-α, IL-1β, IFN-γ and anti-inflammatory IL-10 as compared to PBLs with deficiencies of innate immunity. PRP treatment showed a general decrease in of investigated cytokines, thus reducing the inflammatory response and increasing the innate immunity of PBLs.

**Conclusions:** The results shed light on need for immunomodulatory therapy in AD patients, and indicate on PRP potency to correct an immunological deficits of AD patients. Future research on the regulation of inflammatory response and their influence on neurodegeneration could provide significant improvement of repertoire of early diagnostic biomarkers, and will also open a new adventure for more effective treatment of neurodegeneration like AD.

**Grant Support:** This work was supported by Le Loch Healthcare (Warsaw, Poland) research funds and by internal research funds.

## A83 In vitro blood-tumor barrier models to study paracellular drug transport

### Anurag Paranjape, Brunilde Gril, Patricia S. Steeg

#### National Cancer Institute, Bethesda, MD, USA

##### **Correspondence:** Anurag Paranjape - anurag.paranjape@nih.gov

*Fluids and Barriers of the CNS* 2019, **16(Suppl 1):**A84

When metastases form in the brain, the cancer cells modify the blood–brain barrier into structurally and functionally unique blood-tumor barrier (BTB). BTBs have heterogeneous permeability for drugs.

**Objective:** The objective was to establish in vitro models that mimic in vivo BTBs found in brain metastases, and to study paracellular pathways in brain metastatic vasculature.

**Methods:** In vitro BTB models were set-up using immortalized human brain-microvascular endothelial cells (ECs) and pericytes on opposite sides of a filter. Astrocytes were added to the bottom of the culture, similar to their encirclement of tumor cells in the neuroinflammatory response. Two brain-metastatic variants of breast cancer cell lines (231-BR and JIMT-1-BR) were cultured as spheroids and added to the bottoms of the BTB cultures. Transendothelial electrical resistance (TEER) and permeability of doxorubicin revealed barrier integrity. In vitro results were compared with observations from three different breast cancer brain metastasis (BCBM) mouse models.

**Results:** In vivo analysis had shown that Sphingosine-1-phosphate receptor 3 (S1P3) was overexpressed in astrocytes in highly permeable metastases. The in vitro BTB demonstrated the specificity and functionality of astrocytic S1P3. When astrocytes in BTB were treated with various S1P receptor antagonists (S1P1-5), only S1P3 inhibitors (TY-52156 and CAY10444) modulated the barrier integrity. The tightening of the BTB was observed through an increased TEER, reduced doxorubicin permeability, and higher membranous ZO-1 expression. Removal of astrocytes from the BTB cultures eliminated the effect of S1P3 inhibitor. Similar results were observed when S1P3 was knocked-down in astrocytes. Analysis of culture supernatants revealed that cytokines were downmodulated upon S1P3 knockdown. Inhibition of IL-6 and CCL2 using neutralizing antibodies individually recapitulated the effects of S1P3 inhibition.

**Conclusion:** In vitro BTB models reproduced the in vivo observations on paracellular drug transport across the BTB. The model suggests that S1P3 expressing astrocytes in the neuroinflammatory response produce IL-6 and CCL2 which, in turn, induces endothelial cells to loosen their adhesion.

## A84 Increase of LRP1 expression improves Aβ clearance across the BBB

### Claus Pietrzik^1^, Alexander Mazura^1^, Anke Ohler^1^, Steffen Storck^1^, Christoph Becker-Pauly^2^

#### ^1^Institute for Pathobiochemistry, University Medical Center Mainz, Mainz, RL, Germany; ^2^University Kiel, Intitute for Biochemistry, Kiel, Germany

##### **Correspondence:** Claus Pietrzik - pietrzik@uni-mainz.de

*Fluids and Barriers of the CNS* 2019, **16(Suppl 1):**A85

Late onset Alzheimer’s disease correlates with a successive accumulation of cerebral Aβ which is supposed to be due to impaired clearance mechanisms out of the brain. A significant percentage of Aβ is removed from the brain across the blood brain barrier (BBB) into the vascular system by LRP1 mediated transport. PCSK9 is a secreted serine proteases, which binds to low-density lipoprotein receptors (LDLRs) and targets them for degradation rather than recycling. This results in reduced numbers of functional LDLRs and also in the degradation of LRP1 leading to decreased receptor concentrations at the cell surface. However, the consequence of this regulation on the receptor dependent Aβ clearance across the BBB remains elusive.

We demonstrate a functional interaction between PCSK9 and LRP1 leading to diminished Aβ transcytosis levels using different in vitro BBB models. This reduction in Aβ transport is equivalent to transport rates observed in cells treat with LRP1-disabling antibodies. Using a monoclonal antibody that specifically binds to PCSK9 and inhibits PCSK9-mediated degradation of LDLRs recovers the initial Aβ transport levels in the in vitro system. Transferring the observed effects into an in vivo model, we performed intraperitoneal application of these PCSK9 inhibitory antibodies in 5xFAD mice, which develop a severe amyloid pathology already in early life. The analysis of the cerebral Aβ burden of these animals showing a substantial Aβ reduction compared to control animals indicating a direct effect of PCSK9 on LRP1 at the BBB. In addition, fear conditioning experiments reveal an increased fear response of antibody treated animals suggesting an improved learning behavior.

Our analyses suggest that the peripheral inhibition of PCSK9 with therapeutic agents could be beneficial for the removal of Aβ peptides in Alzheimer’s disease patients and according to the amyloid hypothesis a potential treatment for this kind of neurodegenerative disorder.

## A85 Inhibition of Nox-1 activity attenuates blood brain barrier permeability and edema formation in diabetic ketoacidosis

### Gabriela Martinez-Revollar, Svetlana M. Stamotovic, Richard F. Keep, Anuska V. Andjelkovic

#### University of Michigan, Ann Arbor,MI, USA

##### **Correspondence:** Gabriela Martinez-Revollar - gabriema@umich.edu

*Fluids and Barriers of the CNS* 2019, **16(Suppl 1):**A86

**Objective:** To analyze the role of Nox1 in the blood–brain barrier (BBB) permeability and brain edema formation under diabetic ketoacidosis (DKA) conditions.

**Methods:** The morphological and functional alteration at the brain endothelial barrier under DKA conditions was analyzed in vitro and in vivo models. DKA condition in vivo was induced in homozygous Akita mice (Ins2Akita, 8 weeks) by exposure to a ketogenic diet (35% carbohydrate, 20% protein, 45% fat) for 1–5 days. DKA was confirmed by urinalysis. The BBB permeability and brain edema was determined by MRI. For the in vitro model, mouse brain microvascular endothelial cells (mBMEC) were exposed to DKA mimic condition (30 mM glucose, 17.5 mM AcAc and 12 mM BHOH). Morphological alterations of BBB were analyzed by assessing claudin-5 and ZO-1 protein and mRNA expression, as well as claudin-5/ZO-1 interaction (proximity ligation assay). The production of reactive oxygen species (ROS) and Nox1 activity/expression in DKA was measured in brain microvessels and mBMEC over course of DKA. The effect of Nox1 on BBB permeability was evaluated in inhibition study using the Nox1 inhibitor ML-171.

**Results:** The MRI images reveal increased BBB permeability in the thalamus, partial cortex and basal forebrain at day 3 with edema formation at day 5. The analysis of the morphological and functional changes of BBB under DKA conditions both in vitro and in vivo revealed slight alterations in protein ZO-1 and claudin-5 expression accompanied by the complete loss of interaction between these proteins. DKA condition induced increased ROS production and Nox-1 activation, leading to the subsequent TJ disorganization and increased BBB permeability. The inhibition of Nox-1 activity via Nox-1 inhibitor ML-171 improved the BBB integrity and reduced leakage.

**Conclusions:** We identified that DKA condition cause BBB damage due to Nox-1 activation. The Nox-1 inhibition could be beneficial in preventing DKA complication-BBB injury and brain edema formation.

**Grant Support:** American Diabetes Association.

**Reference**

1. 1-16-IBS-008

## A86 Innovative noninvasive noncontact optical imaging system to detect CBF changes on rodent and human

### Lei Chen

#### University of Kentucky, Lexington, KY, USA

##### **Correspondence:** Lei Chen - lei.chen@uky.edu

*Fluids and Barriers of the CNS* 2019, **16(Suppl 1):**A89

**Objective:** Baseline of CBF and its changes can be early indicators, landmarks and results of neurological diseases. To achieve better understanding of the CBF distribution, variation and its regulation, we developed an innovative, noninvasive technique that can detect CBF change in anesthetized and conscious rodents, as well as on preterm and neonatal human babies.

**Methods:** This technique, called speckle contrast diffuse correlation tomography (scDCT), provides continuous noninvasive, noncontact 3D-imaging of CBF distribution at cortical level, with high resolution and penetration depth covering whole rodent brain. Under anesthesia, the animals’ head hair were shaved and the head secured on a stereotactic frame, a galvo mirror (GVS002, Thorlabs) was used to deliver point-source NIR light generated from a long coherence laser (CrystaLaser) to different source positions and a highly sensitive EMCCD (Cascade 1K, Photometrics) was used to detect spatial speckle contrasts in a selected region of interest (ROI). The raw images were processed with customized software and 2D or 3D image of CBF can be reconstructed.

**Results:** We examined this scDCT technique on mice and rats using various physiological and pathological stimuli, including CO2 inhalation, unilateral hemisphere ischemia from middle cerebral artery occlusion (MCAO), and global ischemia from bilateral ligations of common arteries, and close head injury. scDCT provides very consistent, reliable recording of CBF changes in comparison to other gold-standard methods, including MRI, Laser Doppler and Diffuse Correlation Spectroscopy (DCS). Using this scDCT technique, we have successfully detected the transient (less than 1 h) CBF depression and a long-lasting (for days) hyperemia after each impact on a repetitive close head injury of mouse, as well as the CBF fluctuations in preterm babies with heart defect of patent ductus arteriosus (PDA) and is undergoing Indomethacin treatment.

**Conclusion:** We have developed an innovative method for CBF monitoring with unique advantages and is of high value to improve our understanding of CBF regulation under various conditions, with tremendous potential for translational applications, too.

**Grant Support:** National Institutes of Health (NIH, R21-HD091118, R21-AR062356, R21-AG046762, and COBRE #1P20GM121327), America.

## A87 Inter-alpha inhibitor proteins, blood–brain barrier permeability, and cytokine transport in mouse brain

### Xiaodi Chen^1^, Yow-Pin Lim^2^, Barbara, Stonestreet^1^, William A. Banks^3^

#### ^1^Women & Infant’s Hospital of RI, Providence, RI, USA; ^2^ProThera Biologics, Inc, Providence, RI, USA; ^3^Geriatric Research Educational, and Clinical Center, Veterans Affairs Puget Sound Health Care System, Division of Gerontology and Geriatric Medicine, Department of Medicine, University of Washington, Seattle, USA

##### **Correspondence:** Xiaodi Chen - xchen@wihri.org

*Fluids and Barriers of the CNS* 2019, **16(Suppl 1):**A90

**Objective:** Inter-alpha inhibitor proteins (IAIPs) are immunomodulatory molecules that are in development as therapeutic agents to treat inflammatory disorders in pediatric and adult patients. We have shown that IAIPs have neuroprotective effects on hypoxic ischemic brain injury in neonatal rats and that IL-1β crosses the blood–brain barrier (BBB) in fetal sheep. In the present study, we want to examine BBB permeability measured with IAIPs and the effects of IAIPs on cytokine (IL-1α, IL-6, TNF-α) transport across BBB in mice.

**Methods:** Human IAIPs, TNF-α, IL-1α, and IL-6 were labeled with 125 Iodine and albumin with 99 mTc to measure brain plasma volume. Intravenous (IV) 125I-IAIPs/99mTc-albumin uptake was quantified in adult male CD-1 mice. Blood was obtained 1, 2, 4, 6, 8, 10, 15, and 20 min after injection. The effects of IAIPs on brain cytokine transport were determined by giving IAIPs (100 µg)/saline as an IV co-injection with 125I-TNF-α, -IL-1α, -IL-6, or 99mTc-albumin, and by giving IAIPs (30 mg/kg) by intraperitoneal (i.p.) injection at 6, 24, or 72 h before the onset of the study. Blood was obtained 1, 2, 3, 4, 5, 6, 7, 8, 9, and 10 min after isotope injection. Brain and serum counts were measured, brain/serum ratios (μl/g) calculated and plotted vs exposure time (min). Slope of regression line was taken as the blood-to-brain influx rate (Ki, μl/g min). M ± SEM; ANOVA, Newman–Keuls.

**Results:** The curves of 125I-IAIPs brain/serum vs time, simultaneously injected 99mTc-albumin, and the brain/serum ratio for 125I-IAIPs corrected for vascular space did not reach statistical significance, suggesting that IAIPs do not cross the BBB. The negative corrected Ki value suggests that albumin transfer is more efficient than IAIPs across the BBB. IAIPs injected 6 h before study increased TNF-α transport across the BBB to a statistically significant level. IAIPs did not affect TNF-α transport at 24 or 72 h. IAIP treatment did not affect IL-1α or IL-6 brain uptake.

**Conclusion:** IAIPs does not cross the normal adult mouse BBB and early treatment with IAIPs may facilitate TNF-α brain uptake. We speculate that IAIPs exert their neuroprotective effects potentially by affecting cytokine transport into brain and via systemic targets.

## A88 Inter-alpha protein inhibitors attenuate lipopolysaccharide-induced blood–brain barrier disruption through suppression of interleukin 6

### Aric Logsdon^1^, Kristin Bullock^1^, Xiadoi Chen^2^, Yow-Pin Lim^2^, Barbara Stonestreet^2^, William Banks^1^

#### ^1^University of Washington, Seattle, WA; ^2^Brown University

##### **Correspondence:** Aric Logsdon - alogsdon@uw.edu

*Fluids and Barriers of the CNS* 2019, **16(Suppl 1):**A91

**Objective:** Inter-alpha inhibitor proteins (IAIPs) are serine protease inhibitors expressed in most peripheral tissues and in the brain. In acute inflammatory disorders, circulating levels of IAIP change dramatically, suggesting that they represent an important component of the endogenous immunomodulatory system. Lipopolysaccharide (LPS) exposure results in blood–brain barrier (BBB) disruption with associated increases in inflammatory cytokines; however, inflammatory mechanisms underlying the impaired BBB abnormalities are not well understood. There is a paucity of therapeutic agents that have been shown to attenuate inflammation-related BBB disruption; therefore, we hypothesized that exogenous IAIP treatment would affect LPS-induced inflammation and BBB disruption.

**Methods:** To examine some inflammatory mechanisms associated with LPS related BBB disruption, we administered IAIPs to male and female CD-1 mice after LPS administration. We measured serum IAIP levels using a competitive ELISA. We quantified BBB permeability to intravenous injections of radiolabeled 14C-sucrose and 99 mTc-albumin. We also measured 23 different cytokines in serum and brain tissue by multiplex ELISA.

**Results:** LPS administration increased endogenous serum IAIP levels, as well as multiple cytokines in both serum and brain. LPS-induced IAIP increases correlated with decreases in LPS-induced interleukin 6 (IL6). Exogenous treatment with IAIP also reduced LPS-induced IL6. LPS increased BBB disruption to both 14C-sucrose and 99mTc-albumin, and exogenous treatment with IAIP attenuated 14C-sucrose permeability, but not to 99mTc-albumin.

**Conclusion:** Results suggest that inflammatory mechanisms associated with IL6 may be driving LPS-related BBB permeability. IAIPs attenuate LPS-related BBB permeability to small molecules potentially through an association with IL6. The effects of IAIPs on inflammatory mechanisms associated with IL6 and BBB permeability should therefore be investigated.

## A89 Invasive meningococcal disease: from basic research to clinical applications

### Sandrine Bourdoulous

#### Institut Cochin, Inserm/CNRS/Université Paris Descartes, Paris, France

##### **Correspondence:** Sandrine Bourdoulous - sandrine.bourdoulous@inserm.fr

*Fluids and Barriers of the CNS* 2019, **16(Suppl 1):**A92

Neisseria meningitidis (meningococcus), which normally resides asymptomatically on the human nasopharyngeal mucosa, is pathogenic when it gain access to the bloodstream as it can provoke two rare but devastating diseases, purpura fulminans and meningitis, rapidly causing death or permanent disability, despite prompt antibiotics treatments. We have previously shown that circulating meningococci adhere to human microvascular endothelial cells through direct binding of their type IV pili with endothelial receptor complexes 1–2. Bacteria then rapidly proliferate, forming aggregated bacterial colonies at the endothelial cell surface, and promote signaling events that contribute to vascular alterations. Colonization of brain microvessels is a prerequisite to bacterial crossing the blood–brain barrier, whereas, skin lesions occur secondary to meningococcal colonization of dermal vessels. Type IV pilus-dependent adhesion of meningococci to human microvasculature is determinant to the triggering of both vascular purpuric lesions and inflammation, and a prerequisite to sustained bacteremia responsible for sepsis and subsequent lethality. More recently, we have identified compounds altering bacterial piliation. These compounds reduce vascular colonization by meningococci, prevent subsequent vascular dysfunctions, intravascular coagulation, overwhelming inflammation, and promote survival. As they target a major virulence factor, found in most pathogenic bacteria, these molecules represent promising adjuvant therapy for the treatment of invasive meningococcal disease and other bacterial diseases3.

**References**

1. Pathogenic Neisseria meningitidis utilizes CD147 for vascular colonisation.
2. Bernard SC, Simpson N, Join-Lambert O, Federici C, Laran-Chich MP, Maïssa N, Bouzinba-Ségard H, Morand P, Chretien F, Taouji S, Chevet E, Janel S, Lafont F, Coureuil M, Niedergang F, Marullo S, Couraud PO, Nassif X, Bourdoulous S.
3. Nature Med. 2014; 20: 725–31.
4. Strength of Neisseria meningitidis binding to endothelial cells requires highly-ordered CD147/β2-adrenoceptor clusters assembled by alpha-Actinin-4.
5. Maïssa N, Covarelli V, Janel S, Simpson N, Bernard SC, Pardo-Lopez L, Durel B, Bouzinba-Ségard H, Faure C, Scott MGH, Coureuil M, Morand PC, Lafont F, Nassif X, Marullo S, Bourdoulous S.
6. Nature Commun. 2017; 1.8:15764.
7. Targeting Type IV pili as an antivirulence strategy against invasive meningococcal disease.
8. Denis K, Le Bris M, Le Guennec L, Barnier JP, Faure C, Gouge A, Bouzinba-Ségard H, Jamet A, Euphrasie D, Durel B, Barois N, Pelissier P, Morand PC, Coureuil Lafon

## A90 Loss of regulator of G-protein signaling 5 induces migration in human brain pericytes

### Andreas Enström

#### Lund University, Lund, Sk, Sweden

##### **Correspondence:** Andreas Enström - andreas.enstrom@med.lu.se

*Fluids and Barriers of the CNS* 2019, **16(Suppl 1):**A93

**Objective:** The mechanisms governing pericyte migration are complex and not yet fully understood. In this study, we assessed the role of Regulator of G-protein Signaling 5 (RGS5) on pericyte migration. An interesting candidate as it is one of the first genes being upregulated during vascular morphogenesis both under physiological and pathological conditions.

**Methods:** We utilized a single cell mono-culture system of human brain derived pericytes. Pericyte migration was assessed and quantified using live imaging after either knocking-down or over-expressing RGS5. Cytoskeletal reorganization was examined with immunocytochemistry (ICC). We investigated possible target molecules and signaling pathways attributed to RGS5 using qPCR and western blotting.

**Results:** Loss of RGS5 significantly contributed to increased migration. Cytoskeletal changes and augmented cellular protrusions and trailing ends typical for a migratory phenotype were also observed. The reciprocal approach of over-expressing RGS5 elicited the opposite characteristics further supporting this claim. Furthermore, preliminary results indicate the accumulation of the proteoglycan neural-glial antigen 2 (NG2) in the pericyte cellular membrane. We also detected a link between RGS5 and platelet derived growth factor receptor β (PDGFRβ) signaling as treatment with the receptor ligand PDGF-BB attenuated RGS5 expression.

**Conclusion:** We show that loss of RGS5 increases pericyte mobility and initiate cytoskeletal rearrangements necessary for migration. We hypothesized that the migrational phenotype may be regulated through RGS5 intervention in PDGFRβ signaling and regulation of NG2. Our study supports the role of RGS5 being a novel regulator of pericyte migration and thus, attributing in regulating the integrity of the microvasculature.

## A91 Loss of the transcription factor RBP-J induces disease-promoting properties in brain pericytes

### Rodrigo Diéguez-Hurtado^1^, Katsuhiro Kato^1^, Melina Nieminen-Kelhä^2^, Hendrik Arf^1^, Benedetto Daniele Giaimo^3^, Marek Bartkuhn^3^, Tilman Borggrefe^3^, Peter Vajkoczy^2^, Ralf H. Adams^1^

#### ^1^Max Planck Institute for Molecular Biomedicine, Muenster, NW, Germany; ^2^Charité-Universitätsmedizin Berlin; ^3^Justus-Liebig University Giessen

##### **Correspondence:** Rodrigo Diéguez-Hurtado - rhurtad@gwdg.de

*Fluids and Barriers of the CNS* 2019, **16(Suppl 1):**A94

**Objective:** Despite the relevance of pericytes for blood, brain barrier, neurovascular unit (NVU) integrity and the pathobiology of a wide variety of brain diseases (1–2), our knowledge of the signaling mechanisms responsible for their intercellular communication is limited and requires further attention. Here we aim at elucidating the role of RBPJ, a transcriptional regulator involved in Notch signaling, for pericyte biology in the central nervous system (CNS).

**Methods:** We have used genetic mouse models to conditionally inactivate Rbpj in mural cells, induce acute or chronic deletion of pericytes and promote Notch gain- or loss-of-function scenarios for phenotypic evaluation using gene expression analysis (RNASeq, FACS-RTqPCR), as well as immunostaining and high resolution imaging (confocal and transmission electron microscopy). Moreover, involvement of pericytes during stroke has been studied after distal middle cerebral artery occlusion in adult animals.

**Results:** Postnatal deletion of Rbpj in mural cells impairs brain vascular morphogenesis and NVU homeostasis resulting in severe hemorrhages which are specific to the CNS and which are not mimicked by pericyte ablation. RNASeq analysis revealed that Rbpj is indispensable for maintenance of brain pericytes’ molecular identity and for the regulation of cellular communication with endothelial cells. Indeed, Rbpj-deficient pericytes show increased contractility, change the composition of the extracellular matrix and increase local TGFbeta signaling which affects endothelial cell behavior and blood vessels integrity. Noteworthy, the vascular lesions induced in young mice recapitulate pathological landmarks associated with cerebral cavernous malformations. In adult mice, Rbpj deletion does not induce any obvert phenotype in the CNS under physiologic conditions. Nevertheless, upon ischemic stroke, mutant mice show increased cortical lesions size and a stronger inflammatory response.

**Conclusion:** RBPJ is a key transcriptional regulator necessary for proper molecular identity and functional behavior of CNS pericytes during physiologic angiogenesis and after ischemic insult. We propose that upon Rbpj deletion, brain pericytes can acquire deleterious properties that actively enhance neurovascular lesion formation and promote pathogenic processes in an unprecedented manner.

**Grant Support:** Funding was provided by the Max Planck Society, the University of Münster and the DFG-FOR 2325.

**References**

1. Armulik A et al. Pericytes regulate the blood–brain barrier. Nature. 2010; 468:557–61.
2. Sweeney MD, et al. Pericytes of the neurovascular unit: key functions and signaling pathways. Nat Neurosc. 2017; 26:771–83.

## A92 Manipulation of the blood–brain barrier by tight junction protein-derived peptides

### Reiner F. Haseloff^1^, Jimmi Cording^1^, Sophie Dithmer^1^, Sebastian Pfeil^1^, Christian Staat^1^, Basak Arslan^1^, Susanne M. Krug^2^, Lars Winkler^1^, Ingolf E. Blasig^1^

#### ^1^Leibniz-Forschungsinstitut f. Molekulare Pharmakologie, Berlin, Germany; ^2^Charité Universitätsmedizin

##### **Correspondence:** Reiner F. Haseloff - haseloff@fmp-berlin.de

*Fluids and Barriers of the CNS* 2019, **16(Suppl 1):**A94

**Objective:** Overcoming the blood–brain barrier (BBB) for delivery of drugs is an important challenge in the treatment of brain diseases. Motivated by a growing interest in peptide pharmacology, interactions of peptides derived from tight junction proteins are investigated at the tight junctions, and their effects on the permeability of small molecules is explored in different BBB models.

**Methods:** Experiments on interactions of peptides with tight junction proteins have been carried out in epithelial cells and were validated in brain endothelial cells. Microscopic techniques, such as confocal and super resolution microscopy were applied using different cell lines. Quantitative data on peptide-protein interactions were obtained by microscale thermophoresis. Data were confirmed by measurement of transendothelial/transepithelial resistances and by assessment of the paraendothelial permeability for different model compounds. In addition, in vivo experiments in mice were performed with regard to the effects of peptides on the permeability of small molecules at the BBB.

**Results:** Addition of the claudin-5-derived peptide C5C2 increased the permeability of monolayers of brain endothelial and claudin-5–transfected epithelial cells, and the brain uptake of a small molecule (Gd-diethylene triamine pentaacetic acid, 547 Da) was enhanced in mice. Opening of cellular barriers was also observed in the presence of a claudin-1-derived peptide (C1C2), which improved the permeation of a small molecule at the perineurium. In addition to bicellular junctions, the tricellular junction was identified as a promising target for opening the BBB, since trictide, a peptide derived from tricellulin, increased the permeation across different cellular barriers. On the molecular level, redistribution of tight junction proteins was observed after addition of these peptides to brain endothelial or epithelial cells. Interaction of the peptides with different tight junction proteins was confirmed by measurement of Förster resonance energy transfer, the respective Kd values were found in the nanomolar to low micromolar range.

**Conclusion:** Peptides derived from tight junction proteins constitute a promising target for the delivery of small molecules to the brain. Further studies are necessary to evaluate their applicability in the treatment of brain pathologies.

**Grant Support:** BMBF VIP03V0647, FP7 EU Health consortium JUSTBRAIN.

## A93 Mechanisms of insulin action in the central nervous system

### Elizabeth M. Rhea, William A. Banks

#### VA Puget Sound, Seattle, WA, United States

##### **Correspondence:** Elizabeth M. Rhea - meredime@uw.edu

*Fluids and Barriers of the CNS* 2019, **16(Suppl 1):**A96

**Objective:** The brain is an insulin-sensitive organ and must acquire insulin from the periphery. This requires navigating the blood–brain barrier (BBB). Insulin is primarily transported to the olfactory bulb, hypothalamus, and pons/medulla, but also reaches brain regions important for memory including the hippocampus and frontal cortex. To better investigate what insulin does mechanistically upon reaching the hippocampus, insulin can be administered via the intranasal route. This allows for direct access of insulin to the CNS, limiting peripheral side effects. Intranasal insulin has been shown to improve memory in Alzheimer’s disease (AD) and mild cognitive impairment in both humans and mouse models of AD. We wanted to investigate how insulin entered the CNS (via the BBB) and more specifically what insulin did once present once there (via intranasal delivery).

**Methods:** To investigate transport of insulin across the BBB, we radioactively labelled insulin and used the multiple-time linear regression technique to calculate the unidirectional influx rate. To investigate the actions of insulin within the CNS, we collected brain regions following intranasal insulin delivery and measured molecular changes on both the protein and gene expression levels. We utilized RNA sequencing on hippocampal sections in a young and aged model of AD, the SAMP8 mice.

**Results:** We found insulin crosses the BBB independent of the insulin receptor. Once insulin is present within the brain, it acts through insulin receptor independent pathways. The top gene pathways affected following intranasal delivery include immune related pathways. In addition, we found there are multiple genes related to tight junction and extracellular matrix-receptor interactions that are altered due to age in the SAMP8 mice.

**Conclusion:** These results demonstrate multiple findings. First, it suggests there is a protein other than the insulin receptor that transports insulin into the brain. This will be very important for diseases and conditions in which CNS insulin levels are dysregulated. Second, it suggests that insulin in the brain acts to improve memory through an immune-related mechanism. And lastly, the results show aging in the SAMP8 alters genes involved in the structure of the BBB.

**Grant Support:** NIH: T32AG000057, RO1AG046619.

## A94 Mice deficient in mural cell-derived laminin-α5 presented better recovery after experimental ischemic stroke

### Abhijit Nirwane

#### University of Georgia, Athens, GA, United States

##### **Correspondence:** Abhijit Nirwane - abhijit.nirwane@uga.edu

*Fluids and Barriers of the CNS* 2019, **16(Suppl 1):**A97

**Objective:** In CNS, laminins are involved in blood–brain barrier (BBB) maintenance and regulation. At the BBB, both BMECs and mural cells synthesize and deposit laminin-α5 into the basement membrane. Although dispensable under homeostatic condition the loss of BMEC-derived laminin-α5 enhances inflammatory cell infiltration in pathological states. The function of mural cell-derived laminin-α5, however, remains unknown. Here, we investigated the roles of mural cell-derived laminin-α5 in BBB maintenance under homeostatic conditions and disease progression/outcome in ischemic stroke.

**Methods:** We crossed laminin-α5 flox/flox mice with the Pdgfrβ-Cre+ transgenic line to generate mural cell selective laminin-α5 deficient mice (α5-PKO). The angioarchitecture, BBB permeability, pericyte coverage, tight junction protein (TJP) expression, aquaporin 4 (AQP4) expression and cerebral blood flow (CBF) were measured in α5-PKO mice and their littermate controls under homeostatic conditions. Additionally, the α5-PKO mice and their littermate controls were subjected to 45 min of middle cerebral artery occlusion followed by reperfusion. At various time points (day 1, 2, and 7) after ischemia–reperfusion (I/R) injury, brain infarct volume, neurological deficit score, body weight loss, inflammatory cell extravasation, neuronal death, brain swelling, BBB integrity, pericyte coverage, TJP expression, AQP4 expression, and hemorrhagic transformation were examined.

**Results:** Under homeostatic conditions, no defects in angioarchitecture, BBB integrity, pericyte coverage, TJP expression, AQP4 expression and CBF were observed in α5-PKO mice, suggesting that mural cell-derived laminin-α5 is dispensable for BBB maintenance and CBF regulation under homeostatic conditions. After the I/R injury, however, α5-PKO mice displayed milder BBB disruption, reduced inflammatory cell infiltration, decreased brain swelling, diminished hemorrhagic transformation, reduced infarct volume, decreased neuronal death, and improved neurological function. Mechanistic study showed that mural cell-derived laminin-α5 negatively regulated TJP expression and pericyte coverage after I/R injury.

**Conclusion:** Mural cell-derived laminin-α5 is dispensable for BBB maintenance and CBF measurements under homeostatic conditions but plays a detrimental role in ischemic stroke.

**References**

1. Nirwane A, Yao Y. Laminins and their receptors in the CNS. Biol Rev. 2018.
2. Yao Y. Basement membrane and stroke. J Cereb Blood Flow Metab. 2018; p.0271678X18801467.

## A95 Modelling of the human blood-tumor barrier in pediatric high-grade glioma: evaluation of the chemoresistance properties

### Clémence Deligne^1^, Fabien Gosselet^1^, Samuel Meignan^2^, Pierre Leblond^3^, Marie-Pierre Dehouck^1^, Caroline Mysiorek^1^

#### ^1^LBHE - University of Artois, Lens, France; ^2^Centre Oscar Lambret; ^3^Centre Léon Berard

##### **Correspondence:** Clémence Deligne - clemence_deligne@ens.univ-artois.fr

*Fluids and Barriers of the CNS* 2019, **16(Suppl 1):**A98

**Objective:** Brain tumors are the most frequent solid tumors in children. Among them, diffuse midline glioma (DMG) is almost uniformly fatal and represents the leading cause of brain tumor-related death in the pediatric population. One reason for the clinical failure is the poor access of chemotherapeutic agents to the brain parenchyma due to the presence of the blood–brain barrier (BBB). The BBB, located at the brain capillary endothelial cells, tightly controls the exchanges between the blood and the brain. In most pathological conditions, the specific properties of this barrier are modified, which can modulate the accessibility of drugs to the brain. Thus, our aim was to characterize the physical and metabolic properties of the BBB in the DMG tumoral environment, usually renamed blood-tumor barrier (BTB), using an in vitro approach.

**Methods:** Our model is based on the coculture of human endothelial cells differentiated from CD34+ stem cells with human pericytes (adapted from Cecchelli et al. 2014). This human syngenic BBB model was validated in a new configuration, allowing the development of a tri-culture with either astrocytes or DMG cells (HSJD-DIPG-007, -013 and -014) in order to model the BTB specific to DMG.

**Results:** The results showed that the integrity of the BTB remained intact until 7 days of incubation, which is consistent with clinical observation. The transcriptional expression of several efflux transporters at the BTB was evaluated, as well as the functionality of efflux transporters. Both transcriptional expression and activity did not seem to be modified by the presence of DMG.

**Conclusion:** Further investigation to characterize the metabolic properties of this BTB model and to evaluate the transport of chemotherapeutic drugs is currently conducted. This perspective will allow a better understanding of the mechanisms involved in chemoresistance and, in the long run, an improvement of the therapeutic approach for children with DMG.

**Grant Support:** Région Hauts-de-France, SFCE, Associations “Etoile de Martin” and “Cassandra contre la leucémie”.

**Reference**

1. Cecchelli R, Aday S, Sevin E, Almeida C, Culot M, Dehouck L, Coisne C, Engelhardt B, Dehouck MP, Ferreira L. A stable and reproducible human blood–brain barrier model derived from hematopoietic stem cells. PLoS ONE. 2014;9(6):e99733.

## A96 Modulation of the transforming growth factor-β (TGF-β) co-receptor endoglin (CD105) by oxygen/glucose deprivation in cultured rat brain endothelial cells: relevance to ischemic stroke treatment

### Robert Betterton, Wazir Abdullahi, Junzhi Yang, Bianca Reilly, Samantha Serna, Patrick T. Ronaldson

#### University of Arizona, Tucson, AZ, USA

##### **Correspondence:** Robert Betterton - rdbetter@email.arizona.edu

*Fluids and Barriers of the CNS* 2019, **16(Suppl 1):**A99

**Objective:** Our laboratory has shown that activation of the transforming growth factor-β (TGF-β)/Activin-like Kinase 1 (ALK1) pathway can increase functional expression of organic anion transporting peptide 1a4 (Oatp1a4) at the blood–brain barrier (BBB). This finding is relevant to ischemic stroke treatment because 3-hydroxy-3-methylglutaryl coenzyme A (HMG CoA) reductase inhibitors (i.e., statins) are Oatp transport substrates and improve post-stroke outcomes in patients. We postulate that TGF-β/ALK1 signaling can be targeted to control CNS delivery of statins for stroke treatment; however, it is unknown as to how TGF-β signaling molecules are modulated at the BBB in stroke. The objective of this study is to examine how stroke affects TGF-β signaling components in microvascular endothelial cells and its effects of CNS uptake of statins.

**Methods:** In vitro experiments were conducted using an immortalized rat brain endothelial cell line (RBE4) that were subjected to oxygen–glucose deprivation (OGD). In vivo experiments were conducted using female Sprague–Dawley rats (200–250 g). Animals were administered bone morphogenetic protein-9 (BMP-9; 0–5 μg/kg, i.p.), an established ALK1 agonist and/or LDN193189 (10 mg/kg, i.p.), an established ALK1 antagonist. Brain uptake of [3H]atorvastatin was determined using the established in situ perfusion technique.

**Results:** Activation of TGF-β/ALK1 signaling by BMP-9 increased blood-to-brain transport of atorvastatin by an Oatp-dependent transport mechanism. Atorvastatin uptake was attenuated in the presence of LDN193189, an observation that confirms involvement of ALK1 signaling in the regulation of Oatp-mediated transport. Since we propose that targeting the TGF-β/ALK1 pathway can control statin drug delivery in stroke, we performed in vitro OGD experiments to study this pathway. Following OGD treatment, we observed increased expression of the TGF-β co-receptor endoglin (CD105), which is required for effective ALK1-mediated signaling.

**Conclusion:** Our data indicate that activation of TGF-β/ALK1 signaling can enhance CNS delivery of neuroprotective drugs that are Oatp transport substrates such as atorvastatin. Our studies in the RBE4 cell line suggest that changes in TGF-β signaling molecules may occur in response to ischemic stroke. Studies are ongoing in the laboratory to rigorously assess the effectiveness of targeting TGF-β signaling pathways to optimize Oatp-mediated delivery of neuroprotective drugs in the setting of experimental stroke.

**Grant Support:** This work was supported by grants from the National Institute of Neurological Diseases and Stroke (R01-NS08494).

## A97 Modulation of tight junctions under pathological conditions

### Ingolf Blasig

#### FMP, Berlin, Germany

##### **Correspondence:** Ingolf Blasig - iblasig@fmp-berlin.de

*Fluids and Barriers of the CNS* 2019, **16(Suppl 1):**A100

**Objective:** Claudin-5 tightens the BBB for small molecules and, hence, for 98% of pharmaceuticals. In many brain diseases, the BBB is affected; however, the relevance is widely unclear which leads to following task: clarifying the molecular sealing of the BBB and developing of BBB permeabilisers.

**Methods and results 1:** In a claudin-5 oligomerization assay, the BBB opener 1 (BO1) was selected. After high-affinity binding to claudin-5 (microscale thermophoresis), it concentration-dependently permeabilised the BBB in vitro of up to 38% for small molecules within 24 h (TER, Pc lucifer yellow). In mice, brain uptake of Na-fluorescein peaked already within 3 h and was normalized 6 h after administration. BO1 improved delivery of cytostatics to mouse brain, which reduced glioblastoma size (cresyl violet staining) compared to the BO1-free control. Involving of BO1 derivatives led to a binding model revealing association of its aromatic parts to highly conserved residues at the extracellular and affiliated transmembranous domains of claudin-5 (SwissDock2017). Mode of action: BO1 bound to the extracellularly accessible area of claudin-5, thus weakening the trans-interaction of claudins between opposing cells (enrichment factor reduction); in parallel, claudin-5 was internalized (immuncytochemistry) which attenuated the tight junction (TJ) network (freeze-fracture electron microscopy) and down-regulated claudin-5 (qRT-PCR, immunoprecipitation) by a β-catenin pathway (immunocytochemistry, immunoprecipitation).

**Conclusion 1:** We developed the first small molecule which specifically, partially, moderately, and transiently modulates the BBB allowing paracellular delivery of neuropharmaceuticals (indirect pharmacokinetic effect as drug enhancer).

**Methods and results 2:** After oxidative stress, e.g. ischemia (tMCAO) or hypoxia (hypoxia chamber), claudin-1, -3, -5 and the redox-regulator of the TJs occludin were affected (immunhisto/cyto-chemistry, qRT-PCR, immunoprecipitation). Surprisingly, it was found that reduction of the TJ integrity (transmission EM) resulted in lower infarct size (hematoxylin staining) and lower edema formation (volume determination).

**Conclusion 2:** These findings support our assumption that the modulation of TJ-proteins of the BBB may contribute to a better outcome of stroke (direct therapeutic effect).

## A98 Molecular determinants of human brain endothelial cell differentiation

### Zameel Cader^1^, Satyan Chintawar^1^, Frank Wessely^2^, Caleb Webber^2^

#### ^1^University of Oxford, Oxfordshire, UK; ^2^University of Cardiff, Wales, UK

##### **Correspondence:** Zameel Cader - zameel.cader@ndcn.ox.ac.uk

*Fluids and Barriers of the CNS* 2019, **16(Suppl 1):**A101

**Objective:** We aimed to understand at the single cell level, the molecular changes when a stem cell is directed to differentiate into brain endothelial cells

**Methods:** We devised a novel defined protocol to produce brain capillary endothelial cells (BCECs) from induced pluripotent stem cells. We performed single cell RNA-sequencing and ATAC-sequencing at 4 different stages of the differentiation process.

**Results:** Our differentiation protocol consistently produced BCECs expressing blood–brain-barrier specific junctional proteins and formed an effective barrier with high transendothelial electrical resistance of at least 2000 ohms. Using mass spectrometry we assessed the permeability of our in vitro model to a range of small molecules. We then showed using single cell RNA-seq and ATAC-seq data, the differentiation trajectory from iPSC to BCEC; and the key molecular network underlying this trajectory.

**Conclusion:** We have confirmed that our BCEC differentiation protocol is a reproducible and robust method on a variety of induced and embryonic stem cell lines. We have characterised the key molecular events that occur in the differentiation process.

## A99 Mouse stem cell derived blood–brain barrier model: Applicability to study antibody-triggered receptor-mediated transcytosis

### Anna Jezierski, Junzhuo Huang, Caroline Sodja, Ewa Baumann, Arsalan S. Haqqani, Danica Stanimirovic

#### National Research Council Canada, Ottawa, ON, Canada

##### **Correspondence:** Anna Jezierski - anna.jezierski@nrc.ca

*Fluids and Barriers of the CNS* 2019, **16(Suppl 1):**A102

**Objective:** In vitro blood brain barrier (BBB) models are crucial tools to aid in the pre-clinical evaluation and selection of BBB-crossing therapeutics. Stem cell derived BBB models have recently demonstrated a substantial advantage over primary and immortalized brain endothelial cell models for BBB modelling and maintenance of BBB phenotype in culture. Coupled with recent discoveries highlighting significant species differences in the abundance and function of key BBB transporters, the field is in need of robust, species-specific BBB models for improved translational predictability.

**Methods:** We have developed a mouse stem cell-derived BBB model, composed of mouse embryonic stem cell -derived brain endothelial cells (mBECs), employing a directed monolayer differentiation protocol.

**Results:** These mBECs exhibited barrier formation properties as assessed by high transendothelial electrical resistance, inducible by retinoic acid treatment, up to 500 Ω cm^2^. This robust barrier integrity results in restricted sodium fluorescein permeability (0.017 × 10^−3^ cm/min), magnitudes lower than that of Bend3 cells (1.02 × 10^−3^ cm/min) and comparable to that described for human iPSC-derived BECs (0.020 × 10^−3^ cm/min). The mBECs also express key BBB and endothelial specific markers (Cd31, Cldn5, Occludin and Zo1), polarized expression of functional P-gp efflux transporters and receptor mediated transcytosis triggered by antibodies against specific receptors. A battery of antibodies, binding species selective or cross-reactive epitopes on BBB receptors that trigger receptor-mediated transcytosis, with evaluated in parallel in the mBEC and human iPSC-derived BECs to demonstrate discrimination of species-specific BBB transport mechanisms.

**Conclusion:** Since mouse is the primary species in preclinical studies, it is essential to deploy high-quality mouse BBB models to improve translational predictability and aid in de-risking of CNS drug discovery and development pipelines.

## A100 N-cadherin adhesion induces the assembly of tight junctions to maintain the blood–brain barrier

### Quinn Lee^1^, Kevin J. Kruse^2^, Felecia M. Marottoli^1^, Riya Thomas^1^, Shuangping Zhao^1^, Leon M. Tai^1^, Yulia A. Komarova^1^

#### ^1^University of Illinois at Chicago, Chicago, IL, USA; ^2^University of Utah, UT, USA

##### **Correspondence:** Quinn Lee - slee617@uic.edu

*Fluids and Barriers of the CNS* 2019, **16(Suppl 1):**A103

**Objective:** The blood–brain barrier (BBB) is a tight monolayer of brain endothelial cells (BECs) that interact with surrounding pericytes to restrict the exchange of proteins and extracellular fluids in brain tissue [1]. The interaction between BECs and pericytes is critical in BBB maintenance [2]. Our most recent work indicates that Neural (N)-cadherin, which is expressed in both BECs and pericytes to form heterotypic adhesions, establishes the BBB permeability set-point. [3] The current study investigates the molecular mechanism by which N-cadherin adhesion stabilizes BBB function.

**Methods:** We have utilized transgenic mouse models with an inducible deletion of N-cadherin gene (Cdh2) using endothelial-specific (end-SCL-Cre-ERT2) or pericyte-specific (Pdgfr-β-CreERT2) Cre-Lox systems. To measure changes in BBB permeability, a leakage of 10 kDa and 70 kDa dextran tracers conjugated with AlexaFluor 555 and Oregon Green 488, respectively, were measured using 3D reconstructed images of the brain. Morris water maze was performed on Cdh2-iEC KO and Cdh2fl/fl (Cre-negative control) female mice to assess spatial learning and short-term memory.

**Results:** Our data indicate that an inducible deletion of Cdh2 either in endothelial cells or pericytes increases BBB permeability in a size-dependent manner. These changes in BBB permeability were associated with deficit in spatial learning and memory. Whereas both control and Cdh2-iEC KO mice exhibited normal learning ability, Cdh2-iEC KO mice demonstrated a significant deficiency in short-term memory. Interestingly, deletion of N-cadherin has no effect on pericyte coverage to microvessels, indicating that N-cadherin adhesion modulates BBB integrity independent of other functions of pericytes. Furthermore, deletion of N-cadherin in BECs significantly reduces the accumulation of occludin but not claudin-1 or claudin-5 at tight junctions (TJs), suggesting that N-cadherin adhesion supports the organization of TJs by a yet unknown mechanism.

**Conclusion:** We have demonstrated that N-cadherin juxtacrine signaling supports the BBB through the organization of tight junctions in BECs. Further work will investigate the signaling mechanism by which N-cadherin adhesion induces the assembly of TJs.

**Grant Support:** NIH T32 HL027829, NIH R01 HL103922.

**References**

1. Per Lindahl, Bengt R. Johansson, Per Leveen, Christer Betsholtz. Pericyte loss and microaneurysm formation in PDGFR-B-deficient mice. Science. 1997; 277:242–245.
2. Annika Armulik, Guillem Genove, Maarja Mae, Maya H. Nisancioglu, Elisabet Wallgard, Colin Niaudet, Liqun He, Jenny Norlin, Per Lindblom, Karin Strittmatter, Bengt R. Johansson, Christer Betsholtz. Pericytes regulate the blood–brain barrier. Nature. 2010; 468:557–61, 10.1038/nature09522.
3. Kevin Kruse, Quinn S. Lee, Ying Sun, Jeff Klomp, Xiaoyan Yang, Fei Huang, Mitchell Y. Sun, Shuangping Zhao, Zhigang Hong, Stephen M. Vogel, Jae-Won Shin, Deborah E. Leckband, Leon M. Tai, Asrar B. Malik, Yulia A. Komarova. N-cadherin signaling via Trio assembles adherens junctions to restrict endothelial permeability. J Cell Biol. 2018. 10.1083/jcb.201802076.

## A101 Neuroprotective effects of atorvastatin in experimental stroke requires organic anion transporting polypeptide (Oatp)-mediated transport at the blood–brain barrier

### Patrick T. Ronaldson, Wazir Abdullahi, Jeffrey J. Lochhead, Thomas P. Davis

#### University of Arizona, Tucson, AZ, United States

##### **Correspondence:** Patrick T. Ronaldson - pronald@email.arizona.edu

*Fluids and Barriers of the CNS* 2019, **16(Suppl 1):**A104

**Objectives:** Ischemic stroke is a significant public health concern in the United States. Current therapeutic approaches for stroke include reperfusion therapies but many patients still experience considerable disability despite these interventions. To date, discovery of new drugs for ischemic stroke treatment has been very challenging as indicated by poor translatability of such compounds from preclinical studies to successful Phase III clinical trials. In contrast, 3-hydroxy-3-methylglutaryl coenzyme A (HMG-CoA) reductase inhibitors (i.e., statins) are routinely given to stroke patients because they are known to improve post-stroke outcomes. Neuroprotective effectiveness of statins requires efficient delivery across the blood–brain barrier (BBB). Our laboratory has shown, in vivo, that the endogenous BBB uptake transporter Oatp1a4 facilitates blood-to-brain transport of currently marketed statins (i.e., atorvastatin). The objective of this study was to show that neuroprotective effectiveness of atorvastatin in experimental stroke requires Oatp-mediated uptake transport at the BBB.

**Methods:** Male Sprague–Dawley rats (200–250 g) were subjected to transient middle cerebral artery occlusion (tMCAO) for 90 min followed by 22.5 h reperfusion. Sham-operated animals were used as controls. Atorvastatin (20 mg/kg, i.v.) was injected 2 h following removal of the microfilament. The role of Oatp-mediated transport was determined using the pharmacological Oatp inhibitor fexofenadine (3.2 mg/kg, i.v.) injected at the same time as atorvastatin. Following tMCAO, infarction volume and brain edema ratios were calculated from TTC-stained brain tissue slices. Post-stroke outcomes were assessed after tMCAO via measurement of neurological deficit scores and by the adhesive removal test.

**Results:** In tMCAO animals, atorvastatin significantly reduced both infarction volume and the brain edema ratio. Atorvastatin also improved neurological deficit scores and adhesive removal test performance as compared to sham-operated controls. In the presence of fexofenadine, atorvastatin had no effect on infarction volume or the brain edema ratio. Similarly, positive effects of atorvastatin on post-stroke outcomes were attenuated by fexofenadine.

**Conclusions:** Our data indicate that pharmacological inhibition of Oatp-mediated transport at the BBB prevents atorvastatin from exerting neuroprotective effects in rats subjected to experimental stroke. Studies are ongoing in the laboratory to rigorously study regulation and functional expression of Oatp isoforms at the BBB in the setting of stroke.

**Grant Support:** NINDS R01-NS084941 (P Ronaldson, PI); Arizona Biomedical Research Commission #ADHS16-162406 (P Ronaldson, PI).

## A102 Neurovascular alterations during chronic hypertension

### Ofer Prager^1^, Itamar Burger^2^, Nofar Shemen^2^, Dan Milikovsky^1^, Daniel Zelig^1^, Lior Schori^2^, Avia Elchayani^1^, Griffin Mumby^3^, Alon Friedman^1^

#### ^1^Departments of Physiology & Cell Biology, Cognitive & Brain Sciences, Ben-Gurion University of the Negev, Beer-Sheva, Israel, Beer Sheva, Israel; ^2^Departments of Life Sciences & Psychology, Ben-Gurion University of the Negev, Beer-Sheva, Israel; ^3^Department of Medical Neuroscience, Faculty of Medicine, Dalhousie University, Halifax, B3H 4R2, Canada

##### **Correspondence:** Ofer Prager - pragero@post.bgu.ac.il

*Fluids and Barriers of the CNS* 2019, **16(Suppl 1):**A105

**Background:** Hypertension, also known as a high blood pressure (BP), is a long-term medical condition in which the arterial BP is persistently elevated. High BP is considered as one of the most prevalent chronic medical disorder of adults, and is a major risk factor for myocardial infarction, heart and renal failure, as well as neurological complications. Several epidemiological studies showing that in addition to the increased risk for major acute cerebrovascular events (e.g. hemorrhagic or ischemic stroke) hypertension is also associated with cognitive decline and neurodegenerative disorders 1. The blood–brain barrier (BBB) is a unique structural and functional interface, required for maintaining the unique composition of the neuropil and normal neural function. BBB dysfunction has been reported in hypertension 2, and independently also shown to initiate a signaling cascade leading to neuroinflammation, neural dysfunction and degeneration 3. Objective: In the current study we used the spontaneous hypertensive rats (SHR) model to study early and delayed effects of chronic hypertension on BBB dysfunction and its potential role in neurological complications.

**Methods:** Longitudinal BP measurements and T2, T1-weighted (T2w, T1w) MRI scans of SHR and WKY (as control) rats were repeatedly done during 4 months follow-up, followed by 1 month recording of cerebral cortex activity using electrocorticography (ECoG). Immunostaining against the astrocytic and microglial markers, GFAP and IBA-1 respectively, was performed in search for evidence of astrocytic activation and neuroinflammation. Pentylenetetrazol (PTZ) test was performed to test for seizure threshold. Image and signal analysis were done offline using in-house Matlab algorithms.

**Results:** Hypertension was associated with early increase in regional T1w-signal following contrast agent injection (Dotarem), confirming BBB pathology. Regions with hyper-intense T2w were found as well in several brain sub-regions, suggesting edema and neuroinflammation. Hypertensive rats also showed frequent events of cortical slowing, consistent with lower seizure threshold that was supported by PTZ test. Immunohistological analysis confirmed a neuroinflammatory response within the same brain regions identified with T2w hyper-intensity.

**Summary:** We show that chronic hypertension is associated with early microvascular injury and a neuroinflammatory response, therefore propose BBB dysfunction as a clinically applicable diagnostic means for the early identification of brain injury.

**Grant Support:** European FP7 program (“Epitarget”), Israeli Science Foundation (ISF), Binational Science Foundation (BSF).

**References**

1. Gąsecki D, Kwarciany M, Nyka W, Narkiewicz K. Hypertension, brain damage and cognitive decline. Curr Hypertens Rep. 2013; 15:547–558.
2. Setiadi A, Korim WS, Elsaafien K, Yao ST. The role of the blood–brain barrier in hypertension. Exp Physiol. 2018; 103:337–42.
3. Heinemann U, Kaufer D, Friedman A. Blood–brain barrier dysfunction, TGFβ signaling, and astrocyte dysfunction in epilepsy. Glia. 2012; 60:1251–7.

## A103 Nogo-A targeted therapy promotes vascular repair and functional recovery following stroke

### Ruslan Rust^1^, Lisa Grönnert^2^, Christina Gantner^2^, Alinda Enzler^2^, Geertje Mulders^2^, Rebecca Z. Weber^2^, Arthur Siewert^3^, Yanuar D. P. Limasale^4^, Carsten Werner^4^, Martin E. Schwab^2^

#### ^1^University and ETH Zurich, Schlieren/Zurich, Switzerland; ^2^Institute for Regenerative Medicine, University of Zurich; ^3^Department of Technology, Bielefeld University; ^4^Leibniz Institute for Polymer Research

##### **Correspondence:** Ruslan Rust - rust@irem.uzh.ch

*Fluids and Barriers of the CNS* 2019, **16(Suppl 1):**A106

**Objective:** Nogo-A is an important inhibitor of neurite outgrowth. More recently, it has also been shown to negatively regulate blood vessel sprouting and migration in the developing CNS. However, its function in the adult vasculature and its potential as therapeutic target after neurovascular injury, e.g. ischemic stroke, has not been studied so far.

**Methods:** Using the photothrombotic stroke model, which yields in defined zones of permanent cerebral ischemia, our goal was to investigate the role of Nogo-A in vascular repair within the ischemic border zone. We took advantage of mice deficient for Nogo-A or its corresponding receptor S1PR2, and observed anatomical and functional blood vessel regeneration in the ischemic border zone 3 weeks after injury. To test the therapeutic potential of Nogo-A neutralization, stroked wildtype animals received a continuous intrathecal application of either anti-Nogo-A or control antibodies. Additionally, all animals underwent regular functional tests (ladder rung walking, cylinder test). Moreover, direct interactions of Nogo-A with vascular growth factors were studied in a three dimensional vascular endothelial cultures.

**Results:** We demonstrate that genetic deletion of Nogo-A and S1PR2 anatomically and functionally restores the vascular circulation in the ischemic border zone and reduces neurological deficits following stroke. These findings were reproduced in a therapeutic approach using anti-Nogo-A antibodies. Animals receiving anti-Nogo-A antibodies had increased numbers of GABAergic interneurons, an increase in synaptic and neurotransmitter expression and a higher axon density within the ischemic border compared to their controls. In biohybrid hydrogel models, Nogo-A limits the growth of vascular endothelial cultures, despite the presence of pro-angiogenic vascular growth factors.

## A104 Non-equivalent antigen presenting capabilities of dendritic cells, macrophages and microglia in generating brain-infiltrating CD8+ T cell responses

### Aaron J. Johnson

#### Mayo Clinic, Rochester, MN, USA

##### **Correspondence:** Aaron J. Johnson - johnson.aaron2@mayo.edu

*Fluids and Barriers of the CNS* 2019, **16(Suppl 1):**A107

**Objective:** To define the contribution of various antigen-presenting cell (APC) types, including dendritic cells, macrophages, and microglia, in initiating and promoting CD8 T cell infiltration in the brain. CD8 T cell infiltration into the brain is a critical process in vaccine development against CNS cancers. In addition, CD8 T cell infiltration of the brain is associated with numerous neurologic diseases and CNS infections.

**Methods:** We generated a novel transgenic mouse that enables cell-specific deletion of the H-2Kb MHC class I molecule. By deleting H-2Kb on dendritic cells, macrophages and microglia, we compare the effect of each APC in three distinct models of neuroinflammation: picornavirus infection, experimental cerebral malaria, and a syngeneic glioma.

**Results:** We hypothesized that dendritic cells (DCs), macrophages (MΦs), and microglia would have distinct roles in priming antigen-specific CD8 T cell responses against GL261 glioma, experimental cerebral malaria and picornavirus infection. We assessed antigen-specific CD8 T cell infiltration in CMV-cre Kb cKO animals (ubiquitous Kb deletion), CD11c-cre Kb cKO animals (DC-specific deletion), LysM-cre Kb cKO animals (MΦ-specific deletion), CX3CR1-creER (microglia-specific deletion) and cre- littermates in these three model systems. We demonstrate that DC-specific deletion of H-2 Kb increased the proportion of glioma-bearing animals. Additionally, we determined that DC-specific deletion, but not MΦ-specific deletion, of H-2Kb reduced the proportion of tumor-specific CD8 T cells and increased tumor burden, but not as severely as ubiquitous H-2Kb deletion. Thus, DCs, but not MΦs, are critical for generation of a complete anti-glioma T cell response. Finally, microglia are important for CD8 T cell entry into the brain during picornavirus infection.

**Conclusion:** Dendritic cells, macrophages and microglia all serve a roll in CD8+ T cell infiltration into the brain in response to these distinct CNS immunological challenges. However, the extent to which each of these APCs contributes to CD8+ T cell priming varies. For example macrophages served no role in CD8 T cell priming against tumor or picornavirus infection. These findings reveal distinct functions for dendritic cells, macrophages, and microglia in generating brain infiltrating CD8+ T cell responses against tumor and infectious diseases.

**Grant Support:** R01 NS103212, R21 CA186976.

**Reference**

1. Non-equivalent antigen presenting capabilities of dendritic cells and macrophages in generating brain-infiltrating CD8+ T cell responses. Malo CS, Huggins MA, Goddery EN, Tolcher HMA, Renner DN, Jin F, Hansen MJ, Pease LR, Pavelko KD, Johnson AJ. Nat Commun. 2018;9(1):633. PMID:29434238

## A105 Noninvasive estimation of tumor interstitial fluid pressure by DCE-MRI and its confirmation by invasive wick-in-needle technique in a rat glioblastoma model

### Tavarekere N. Nagaraja, Rasha Elmghirbi, Stephen L. Brown, Swayamprava Panda, Glauber Cabral, Ian Y. Lee, Robert A. Knight, James R. Ewing

#### Henry Ford Hospital, Detroit, MI, USA

##### **Correspondence:** Tavarekere N. Nagaraja - tnagara1@hfhs.org

*Fluids and Barriers of the CNS* 2019, **16(Suppl 1):**A108

**Objective:** Dysfunctional blood-tumor barrier and elevated tumor interstitial fluid pressure (TIFP) limit perfusion and increase hypoxia. TIFP contributes to peri-tumoral exudate flow and edema. Peri-tumoral exudate flow and edema counter tumor penetration by chemotherapeutics. Increased TIFP is also a mark of tumor aggressiveness, and decreased TIFP, a predictor of response to therapy. However, non-invasive techniques are unavailable for measuring TIFP in cerebral tumors. This study employed dynamic contrast-enhanced magnetic resonance imaging (DCE-MRI) to estimate TIFP and its confirmation by an invasive method using a rat glioblastoma model.

**Methods:** Athymic rats (n = 22) were implanted intracerebrally with U251 glioblastoma. At 2 weeks post-implantation, DCE-MRI was conducted in control (n = 13) and bevacizumab-treated rats (n = 9), followed by invasive TIFP measurement in each animal using a ‘wick-in-needle’ (WIN) method. Applying model selection paradigm, Patlak- and Logan- plots to DCE-MRI data, extracellular volume fraction (porosity) and velocity of exudate fluid flow at the tumor boundary were derived to estimate TIFP by Darcy’s law. Two models, a fluid-mechanical model and a multivariate empirical model, were used for TIFP estimations and verified against WIN-TIFP. WIN-TIFP and MRI-TIFP data were tested for correlation by linear regression and significance inferred at p < 0.05.

**Results:** Using DCE-MRI, the mean estimated hydraulic conductivity (MRI-K) was (2.3 ± 3.1) x 10^−5^ (mm^2^/mm Hg-s) in control studies. Significant positive correlations were found between WIN-TIFP and MRI-TIFP in both mechanical and empirical models. For instance, in the control group of the fluid-mechanical model, MRI-TIFP was a strong predictor of WIN-TIFP (R = 0.76; p < .01). Similar result was found in the bevacizumab-treated group in the empirical model (R = 0.87; p < .01). In controls mean WIN-TIFP was 6.0 ± 3.7 and MRI-TIFP, 6.2 ± 3.7. Bevacizumab decreased the mean TIFP, albeit to slightly varying degrees by the two methods, 2.8 ± 1.6 (WIN) and 5.3 ± 3.3 (MRI). Both control and bevacizumab groups showed a high degree of inter-method correlation with R = 0.9 (p < 0.01) between the WIN- and MRI-TIFP measurements.

**Conclusion:** These data suggest that DCE-MRI studies contain enough information to noninvasively estimate TIFP in this, and possibly other, glioma models and, thus, might be useful to assess tumor aggressiveness and responses to therapies aiming to decrease TIFP and increase tumor drug delivery.

**Grant Support:** R01 CA135329 (J.R.E.).

## A106 Novel therapeutic targets to repair blood–brain barrier dysfunction in epilepsy

### Bjoern Bauer^1^, Brent S. Sokola^1^, Satya R. Alluri^1^, Ralf G. Rempe^1^, Emma L.B. Soldner^2^, Evan M. Sawyer^1^, James R. Pauly^1^, Anika M. S. Hartz^1^

#### ^1^University of Kentucky, Lexington, KY, United States; ^2^University of Minnesota, Duluth, MN, United States

##### **Correspondence:** Bjoern Bauer - bjoern.bauer@uky.edu

*Fluids and Barriers of the CNS* 2019, **16(Suppl 1):**A109

**Objective:** With more than 50 million patients worldwide, epilepsy is the most common neurological disorder. In epilepsy, the blood–brain barrier is dysfunctional thereby contributing to seizure genesis and resistance to anti-seizure drugs. We have shown that increased brain glutamate levels during seizures lead to barrier dysfunction, a condition associated with barrier leakage. Based on our data, we hypothesized that glutamate released during seizures mediates blood–brain barrier dysfunction and that dual LOX/COX inhibition repairs barrier leakage in vivo.

**Methods:** To test this hypothesis, we exposed isolated rat brain capillaries to glutamate ex vivo w/wo modulators of the LOX/COX pathway and employed an in vivo/ex vivo approach using isolated brain capillaries from rats that experienced status epilepticus as an acute seizure model.

**Results:** Found that glutamate signals through NMDAR, cPLA2 and LOX/COX to increase MMP-2 and MMP-9 protein and activity levels, leading to a decrease in tight junction protein expression levels, which resulted in brain capillary leakage. We confirmed these findings in vivo in brain capillaries from rats that experienced status epilepticus and in brain capillaries from mice lacking cPLA2. We also show that status epilepticus rats have increased brain capillary leakage, elevated S100β serum levels, and increased TSPO brain levels, indicating barrier dysfunction and neuroinflammation. Consistent with these findings, we detected increased MMP levels in brain capillaries and increased S100β serum levels in postmortem human samples from individuals that experienced generalized seizures compared to seizure-free control individuals. Lastly, we demonstrate that treating status epilepticus rats with LOX/COX inhibitors reduces S100β serum levels and prevents barrier leakage compared to untreated status epilepticus rats.

**Conclusion:** Our data provide new insights into the underlying mechanism by which seizures cause blood–brain barrier dysfunction and provide first evidence that targeting the LOX/COX pathway has the potential to restore barrier function in epilepsy.

**Grant Support:** 1R01NS079507 (NINDS/NIH; PI: Bauer), 1R01NS107548 (NINDS/NIH; PI: Bauer).

## A107 Occludin-Alix-Cav-1 interactions are affected in HIV infected pericytes

### Silvia Torices, Minseon Park, Samantha A. Roberts, Abby M. Mintz, Michal Toborek

#### University of Miami, Miami, FL, United States

##### **Correspondence:** Silvia Torices - sxt736@med.miami.edu

*Fluids and Barriers of the CNS* 2019, **16(Suppl 1):**A110

**Objective:** HIV enters the central nervous system (CNS) early after infection and disrupts the blood–brain barrier (BBB). Functional and structural disturbances of tight junctions (TJ) alter BBB permeability. Occludin and caveolin (cav-1) regulate TJ protein expression and are critical determinants of BBB permeability. Alix is an early-acting ESCRT factor associated with membrane proteins that is involved with HIV budding from the cells. Although the impact of HIV on BBB endothelial cells and astrocytes has been well elucidated, the role of pericytes during HIV infection remains largely undescribed.

Our goal is to study the relationship between the integrity of this protein complex and HIV replication in human brain vascular pericytes.

**Methods:** Using co-immunoprecipitation and immunostaining techniques we demonstrated that cav-1, occludin and Alix form stable complexes. To understand the molecular interactions within this complex, studied the impact of each component of this protein complex. Pericytes were transfected with an occludin expression vector and siRNA of cav-1. The cells were then either controlled or infected with HIV for 48 h. The expression of individual proteins was evaluated by Western Blot.

**Results:** We observed that occludin overexpression decreased the expression of cav-1 but not Alix. We also described that cav-1 knockdown increased the protein levels of occludin, but not Alix. Interestingly, we found that HIV attenuates occludin regulation via cav-1 interaction and cav-1 knockdown does not increase the protein level of occludin.

**Conclusion:** Our results describe a complex between caveolin-1, occludin and Alix proteins. The interactions between proteins within the complex can modulate the function of its individual components. HIV can alter the complex and attenuate protein interactions within infected pericytes.

**Grant Support:** National Institutes of Health (NIH), grants MH098891, MH072567, HL126559, DA039576, DA040537, and DA044579.

## A108 Oncostatin M-reactive pericytes induce blood–brain barrier impairment by activating JAK/STAT3 signaling in vitro

### Fuyuko Takata^1^, Shinya Dohgu^1^, Ikuya Kimura^1^, Shinya Sakaguchi^1^, Kenta Sakai^1^, Christophe Place^2^, Atsushi Yamauchi^1^, Yasufumi Kataoka^1^

#### ^1^Fukuoka University, Fukuoka, Japan; ^2^ENS de Lyon, Lyon, France

##### **Correspondence:** Fuyuko Takata - ftakata@fukuoka-u.ac.jp

*Fluids and Barriers of the CNS* 2019, **16(Suppl 1):**A111

**Objective:** Oncostatin M (OSM) is a member of the interleukin (IL)-6 family cytokines and factors to induce BBB dysfunction by activating JAK/STAT3 pathway in the brain endothelial cells. Brain pericytes located around the microvessels are one of the constituents of the blood–brain barrier (BBB). Pericytes work as an interface between blood and brain parenchyma and their functions were altered under the pathophysiological conditions, leading to BBB dysregulation. However, it remains obscure whether pericytes are associated with OSM-induced BBB dysfunction.

**Methods and results:** Then, we showed that an exposure of pericytes to OSM (100 ng/mL) elevated phosphorylation levels of the STAT3, a main signaling pathway, and that pericytes expressed OSM receptor mRNA. These suggest that pericytes have an ability to respond to OSM. To determine the effects of OSM-reactive pericytes on BBB functions, rat brain endothelial cell (RBEC) monolayers were cocultured with OSM-treated pericytes. OSM (100 ng/mL for 48 h) stimulated pericytes to markedly produce increased sodium fluorescein (Na-F) permeability and decreased transendothelial electrical resistance (TEER) in rat brain endothelial cell (RBEC) monolayers. These impaired BBB functions were blocked by ruxolitinib, a JAK inhibitor. The present findings suggest that the activation of JAK/STAT3 pathway in pericytes largely contributes to OSM-induced BBB impairment.

**Conclusion:** Thus, OSM-reactive pericytes should be considered as a possible hallmark for the induction and development of BBB breakdown under the pathological conditions accompanied with increased OSM levels.

## A109 Opposite effects of hypertension and exercise training on blood–brain barrier integrity within the hypothalamic paraventricular nucleus: Focus on transcytosis and transcelular transport

### Hiviny de Ataides Raquel, Vanessa Cândido Abreu, Mateus Garcia de Fragas, Alison Colquhoun, Lisete Compagno Michelini

#### University of São Paulo, São Paulo, SP, Brasil

##### **Correspondence:** Hiviny de Ataides Raquel - hiviny_ataides@hotmail.com

*Fluids and Barriers of the CNS* 2019, **16(Suppl 1):**A112

**Objective:** Blood–brain barrier (BBB) leakage within autonomic areas of hypertensive rats (Biancardi et al. Hypertension 2014) is promptly corrected by exercise training (Buttler et al. Front Physiol 2017). This study investigated the mechanisms underlying the effects of hypertension and training on BBB ultrastructure and function within the paraventricular hypothalamic nucleus (PVN).

**Methods:** SHR and WKY were submitted to aerobic training (T, 55% maximum capacity, 1 h/day, 5 d/week) or kept sedentary (S) for 4 weeks. After hemodynamic measurements at rest, rats were allocated to 3 different protocols: (1) evaluation of BBB integrity by the degree of FITC leakage into the brain parenchyma after carotid infusion of 2 fluorescent dextrans (FITC-10 kDa + Rhodamine-70 kDa, 286 μl/100 g) followed by brains’ harvesting 20-min later; (2) analysis of gene and protein expression of BBB constituents (qPCR in micro-dissected nucleus from fresh hypothalamic sections and immunohistochemistry in fixed/cryoprotected PVN sections, respectively); (3) investigation of the effects of hypertension and T on capillaries’ ultrastructure in ultrathin PVN sections (electron microscopy). Acquired images were processed by Image J software. Data was analyzed by 2-way ANOVA.

**Results:** SHR-S (vs. WKY-S) exhibited higher MAP and HR (56% and 9%), significant FITC leakage (11.6 ± 1.3% of PVN area), increased gene and protein expression of caveolin-1 (+ 2.8-fold increase in Cav-1 immunoreactivity normalized by RECA-1 expression in PVN capillaries) but unchanged Claudin-5, Occludin and ZO-1 expression. These changes were accompanied by increased number of luminal and abluminal vesicles in endothelial cells (+ 46%), but unchanged occurrence of tight junctions (TJs). In the SHR group, T was accompanied by significant HR reduction, partial MAP fall, marked reduction in BBB leakage (5.4 ± 0.6%), normalized Cav-1 gene and protein expression, significant reduction of transcytosis without changing the number of TJs/PVN capillary and the expression of TJs proteins. WKY-T also showed small HR reduction accompanied by mild, not significant changes in FITC leakage, Cav-1 expression and in the number of luminal/abluminal vesicles.

**Conclusion:** Both BBB dysfunction in hypertension and its correction by exercise are conditioned by changes in transcytosis across PVN endothelial cells without changing TJs and paracellular transport. Training-induced adaptive response may contribute to improvement of brain perfusion in hypertensive individuals.

**Grant Support:** Foundation for Research Support of the State of São Paulo - FAPESP.

**References**

1. BIANCARDI, VC et al. Circulating angiotensin II gains access to the hypothalamus and brain stem during hypertension via breakdown of the blood–brain barrier. Hypertension. 2014; 63(3):572-9.
2. Buttler L et al. Maintenance of blood–brain barrier integrity in hypertension: A novel benefit of exercise training for autonomic control. Front Physiol Front Physiol. 2017;8:1048.

## A110 PDGFRβ signal inhibition in brain pericytes suppresses glial activation and increased seizure susceptibility to pilocarpine in mice with traumatic brain injury

### Kenta Sakai, Fuyuko Takata, Yoshie Tanaka, Yume Sezaki, Kazuki Tominaga, Kana Hashiguchi, Miho Yasunaga, Atsushi Yamauchi, Shinya Dohgu, Yasufumi Kataoka

#### Fukuoka University, Faculty of Pharmaceutical Sciences, Fukuoka, Japan

##### **Correspondence:** Kenta Sakai - y6skh0105@gmail.com

*Fluids and Barriers of the CNS* 2019, **16(Suppl 1):**A113

**Objective:** Traumatic brain injury (TBI) develops late posttraumatic epilepsy (PTE) characterized by long and unexpected latencies until the onset of seizure. Epidemiological evidences have suggested that prophylactic therapy with typical antiepileptic drugs for this late PTE have not succeeded. Thus, an understanding of the mechanism by which TBI causes late PTE is urgently needed for early diagnosis, prevention and treatment. We hypothesized that TBI occurs late PTE due to dysfunction of the central nervous system (CNS) supporting cells such as pericytes and glial cells (astrocytes and microglia). Our goal is to clarify a role of pericytes in the late PTE development.

**Methods:** TBI model animals were prepared using controlled cortical impact (CCI) in C57BL/6 J mice. We evaluated the possibility of late PTE onset with seizure susceptibility to the sub-threshold dose of pilocarpine on 7, 14, 21 and 28 days after CCI. Histological analyses of the pericytes, astrocytes and microglia were performed on 1 h, 2, 7 and 28 days after CCI. CCI mice were treated with imatinib (twice a day, 200 mg/kg, p.o.), an inhibitor of PDGFR signaling pathway for 5 days after injury and then were subjected to pilocarpine seizure test and histological analysis.

**Results & conclusion:** The PDGFRβ immunoreactivities in pericytes were increased in the ipsilateral hippocampus during a period from 1 h to 28 days after CCI. The expressions of GFAP and Iba1 as a marker for astrocyte and microglia reactivity, respectively, were increased from 7 to 28 postoperative days. The incidence and severity of seizure induced by PILO were gradually increased from 14 days after, with a significant increase at 28 days after CCI. These findings suggested that the increased PDGFRβ expression in pericytes precedes glial activation and increased seizure susceptibility after CCI. Imatinib apparently reduced seizure susceptibility to pilocarpine and glial activation in the ipsilateral hippocampus at 28 postoperative days. Thus, an inhibition of the increased PDGFRβ signaling in pericytes in the early stage after CCI could ameliorate CNS dysregulation due to CCI-induced glial activation, leading to reduced risk for late-onset PTE.

## A111 Pericyte-derived microvesicles in brain diseases

### Abderahim Gaceb

#### Translational Neurology Group, Lund, 01, Sweden

##### **Correspondence:** Abderahim Gaceb - abderahim.gaceb@med.lu.se

*Fluids and Barriers of the CNS* 2019, **16(Suppl 1):**A114

**Objective:** Brain pericytes are perivascular cells that share mesenchymal stem cells properties. They represent a novel source of inflammatory cells in stroke and mediate the restorative effect of platelet-derived growth factor-BB (PDGF-BB) in Parkinson’s disease (PD). However, cell-signaling mechanisms of pericytes under both conditions remains elusive. Recently, the regenerative properties of mesenchymal stem cells have been attributed to the release of “Microvesicles” (MVs). We hypothesize that the inflammatory and the regenerative properties of brain pericytes may be mediated by pericyte-derived MVs.

**Methods:** We used: (i) human pericytes lines exposed to Oxygen Deprivation (OD) simulating hypoxia condition or PDGF-BB treatment, (ii) mice submitted to MCAO or PD mice treated by PDGF-BB.

**Results:** Under oxygen deprivation, pericytes release MVs, which contain a proinflammatory cytokine “IL-6”. In vivo, we identified a hight number of MVs carrying PDGFRbeta, specific marker of brain pericytes, in the plasma of stroke mice. On the other hand, upon in vitro treatment by PDG-BB, pericytes upregulate the production of several growth factors like: bNGF, NT3, HGF, PLGF, FGFb, FGF4, HBEGF, EGF, as well as the liberation of MVs compared to other stimuli. PDGF-BB treatment leads to the activation of ERK1/2 pathway, mediated by increased PDGFRbeta phosphorylation but not PDGFRalpha.

**Conclusion:** Our data provide evidence that pericytes derived MVs present a new actor in neuroinflammation after stroke and also the neurorestorative effect of PDGF-BB in PD.

## A112 Pericyte-mediated amyloid clearance route is impaired in Alzheimer’s disease

### Tunahan Kirabali^1^, Filip Liebsch^2^, Adeola Shobo^3^, Roger Nitsch^1^, Gerhard Multhaup^3^, Luka Kulic^2^

#### ^1^University of Zurich, Schlieren, Switzerland; ^2^University of Cologne; ^3^McGill University

##### **Correspondence:** Tunahan Kirabali - tunahan.kirabali@irem.uzh.ch

*Fluids and Barriers of the CNS* 2019, **16(Suppl 1):**A115

**Objectives:** The C-terminally truncated amyloid beta-peptide (Aβ) isoform, Aβ34, is an important intermediate product of enzymatic Aβ degradation and has the potential to be used as a biomarker of impaired amyloid clearance in Alzheimer’s disease (AD). In this study, we aimed to identify the distribution of Aβ34 in human brain. Discovery of Aβ34 and brain pericyte association led to further characterization of a novel pericyte-mediated Aβ clearance pathway and Aβ34 metabolism in pericytes.

**Methods:** Human post-mortem cortex and hippocampus samples were analyzed by immunohistochemistry using a highly specific novel monoclonal antibody directed against Aβ34 and pericyte marker PDGFR-β. Microvessels isolated from human cortex were analyzed with immunohistochemistry and immunoassays for the presence of Aβ34 along perivascular drainage pathways. In addition, Aβ34 metabolism in pericytes was studied in vitro with human primary pericyte cultures.

**Results:** Aβ34 immunoreactivity was observed exclusively in vessels in human post-mortem samples. However, two distinct vascular pathologies were identified in different Braak stages. In capillary associated pathology, Aβ34 immunoreactivity was detected in pericytes on capillary basement membrane. On the other hand, in artery associated pathology, Aβ34 was found in larger congophilic amyloid angiopathy (CAA)-laden vessels, co-localizing with other amyloid isoforms. In early Braak stages, capillary associated pathology was more prominent however, in late Braak stages pathology shifted to arteries. Further quantification showed that capillary associated pathology decreased in AD subjects compared to non-demented controls and significantly correlated with PDGFR-β both in hippocampus and cortex. Aβ34 immunoreactivity was also detected in isolated human microvessels. Measurements in microvessel enriched brain lysates showed a similar correlation between PDGFR-β and Aβ34. Metabolism of amyloid beta towards Aβ34 production was investigated in pericytes upon treatment with longer isoforms. Aβ40 treated pericytes generated Aβ34 in vitro in time and dose-dependent manner.

**Conclusion:** Early detection of Aβ34 in the vasculature and its association with pericytes point to the existence of a novel Aβ degradation pathway along vascular clearance routes. Failure of this degradation pathway might contribute to increased Aβ accumulation and amyloid deposition in early AD pathogenesis.

**References**

1. Fluhrer R, et al. Identification of a β-secretase activity, which truncates amyloid β-peptide after its presenilin-dependent Generation. J Biol Chem. 2003; 278:5531–8.
2. Hernandez-Guillamon M, et al. Sequential amyloid-β degradation by the matrix metalloproteases MMP-2 and MMP-9. J Biol Chem. 2015;290:15078–91.
3. Portelius, E. et al. Acute effect on the Aβ isoform pattern in CSF in response to γ-secretase modulator and inhibitor treatment in dogs. J. Alzheimers Dis. 2010;21: 1005–12.

## A113 Pericytes, the silent modulators of CAA during stroke: THE SNOWBALL EFFECT

### Anupriya Mehra^1^, Fabien Gosselet^2^, Laurence Tilloy-Fenart^2^

#### ^1^The University of Artois - Université d’Artois, Lens, France; ^2^Laboratory of the Blood Brain Barrier

##### **Correspondence:** Anupriya Mehra - anumeh.am17@gmail.com

*Fluids and Barriers of the CNS* 2019, **16(Suppl 1):**A116

**Objective:** CAA is recognized as the microfibrillar deposition of amyloid beta peptides around small brain blood vessels and arteries, is commonly observed as disrupted vascular structure, fibroid necrosis and microaneurysms in Alzheimer’s disease (AD) patients. In recent years, progression of CAA in AD has been strongly linked to the increased risk of stroke and vice versa. As an important entity of neurovascular unit, we investigate the role of pericytes in CAA like amyloid beta pathology under stroke like conditions.

**Methods:** We introduce a new in vitro model for extraction of primary pericytes by an antibiotic-free, enrichment based approach. In this study, we investigate the internalization of two variants of amyloid beta family, i.e. 1–40 and 1–42 by pericytes. To check the secretome profile of cerebral pericytes we use immunoassays and spectrophotometry. Furthermore, we use qPCR and western blotting to investigate proteins associated with metabolism of amyloid beta peptides and receptor proteins associated with amyloid beta clearance and their functionality.

**Results:** Our new approach for extraction of primary murine pericytes is highly efficient and is an appropriate tool for in vitro studies. In this study we found that the internalization of amyloid beta 1–40 in brain pericytes is decreased under hypoxia (twofold difference of internalization, p < 0.0005). Under similar conditions, amyloid beta stress increases active ROS/RNS response and the inflammatory factors IL-Alpha IL-1Beta, IL-RA and MCP-1. Interestingly, amyloid beta stress also mediates an early drop and late rise in the expression levels of amyloid beta degradation specific enzymes ECE-1, ECE-2 and ACE. In addition, an alteration in expression of LRP-1 and ABCA7 receptors is very intriguing.

**Conclusion:** At current stage of this project we confirm that stroke like conditions promote the accumulation of amyloid peptides around the brain vasculature, probably by altering LRP1 and ABCA7 levels. This aggravates the severity of pathophysiological responses which are observed with high oxidative stress and elevation in levels of inflammatory markers. These responses shall further accelerate the progression of CAA in AD, termed as a SNOWBALL EFFECT.

**Grant Support:** Project SNOWBALL is supported by EU Joint Programme – Neurodegenerative Disease Research (JPND).

**References**

1. Qingyi Ma, Zhen Zhao, Abhay P Sagare, Yingxi Wu, Min Wang, Nelly Chuqui Owens, Philip B Verghese, Joachim Herz, David M Holtzman and Berislav V Zlokovic. Blood–brain barrier-associated pericytes internalize and clear aggregated amyloid-β42 by LRP1-dependent apolipoprotein E isoform-specific mechanism. Mol Neurodegener. 2018;13(1):57.
2. Natalia N. Nalivaeva, Anthony J. Turner. role of ageing and oxidative stress in regulation of amyloid-degrading enzymes and development of neurodegeneration. Curr Aging Sci. 2017;10(1):32–40.
3. Kuntz M, Mysiorek C, Pétrault O, Pétrault M, Uzbekov R, Bordet R, Fenart L, Cecchelli R, Berezowski V. Stroke-induced brain parenchymal injury drives blood–brain barrier early leakage kinetics: a combined in vivo/in vitro study. J Cereb Blood Flow

## A114 Physical activity modulates methamphetamine—induced metabolomic changes in the hippocampus

### Arkadiusz Liśkiewicz^1^, Marta Przybyła^1^, Anna Wojakowska^2^, Łukasz Marczak^2^, Daniela Liśkiewicz^1^, Marta Nowacka-Chmielewska^1^, Andrzej Małecki^1^, Michał Toborek^3^

#### ^1^The Jerzy Kukuczka Academy of Physical Education in Katowice, Katowice, Polska; ^2^Institute of Bioorganic Chemistry, Polish Academy of Sciences, ul. Noskowskiego 12/14, Poznan, Poland; ^3^Department of Biochemistry and Molecular Biology, University of Miami, School of Medicine, Miami, FL, USA

##### **Correspondence:** Arkadiusz Liśkiewicz - adliskiewicz@gmail.com

*Fluids and Barriers of the CNS* 2019, **16(Suppl 1):**A117

**Objective:** Methamphetamine (METH) abuse results in the brain toxicity and physical activity may protect against this drug-related detrimental brain changes. However, the neuroprotective mechanisms of exercise are not fully understood. Metabolomics proved to be a useful tool in studies on the central nervous system, indicating that changes in the brain metabolism may contribute to the generation of complex brain disorder phenotypes. It is known that METH and physical activity can impact the brain metabolomic composition but their mutual impact was not examined before.

**Methods:** The goal of the present study was to apply global metabolic profiling (GC/MS) in the hippocampal samples of exercised mice and inactive controls treated with METH. Mice were allowed to voluntary wheel running for 2 weeks before administration with increasing doses of METH or vehicle for 5 days (Park et al. 2016; Liśkiewicz et al. 2019). Analysis of the collected hippocampi revealed that METH and exercise markedly changed composition of the brain metabolites.

**Results:** Among identified compounds, we observed that ascorbic acid, lysine, and mannitol were elevated, and sorbose was decreased in METH treated mice. Interesting, physical activity normalized the levels of these metabolites.

**Conclusion:** Overall, the study demonstrates that METH-related metabolomic changes in the brain may be reversed, at least in part, by physical activity, expanding the knowledge on neuroprotective effect of exercise in drug abuse. The work was supported by the NSC grant 2015/17/B/NZ7/02985

**Grant Support:** NSC grant 2015/17/B/NZ7/02985.

**References**

1. Park M, Levine H, Toborek M. Exercise protects against methamphetamine-induced aberrant neurogenesis. Sci Rep. 2016;6:34111. 10.1038/srep34111.
2. Liśkiewicz A, Przybyła M, Park M, Liśkiewicz D, Nowacka-Chmielewska M, Małecki A, Barski J, Lewin-Kowalik J, Toborek M. Methamphetamine-associated cognitive decline is attenuated by neutralizing IL-1 signaling. Brain Behav Immun. 2019. pii: S0889-1591(18)30732-3. 10.1016/j.bbi.2019.03.016.

## A115 Polarized responses of human iPSC-derived brain endothelial cells to cytokines

### Michelle Erickson^1^, Kim Hansen^2^, William A. Banks^1^

#### ^1^Veterans Administration Puget Sound Healthcare System, Seattle, WA, United States; ^2^University of Washington

##### **Correspondence:** Michelle Erickson - mericks9@uw.edu

*Fluids and Barriers of the CNS* 2019, **16(Suppl 1):**A118

**Objective:** Endothelial cells of the blood–brain barrier (BBB) are important interfaces for neuroimmune communication. Brain endothelial cells (BECs) are anatomically positioned to respond to stimuli from the brain or blood compartments and can relay immune signals between the brain and periphery. BECs are polarized, and so may respond differently to signals encountered at the abluminal (brain) vs. luminal (blood)-facing membrane. In this study, we aimed to determine whether iPSC-derived brain endothelial cells (i-BECs) have polarized responses to cytokines.

**Methods:** We used an induced pluripotent stem cell (iPSC)-derived human BBB model cultured on transwell inserts. BECs generated from iPSCs (i-BECs) have high transendothelial electrical resistance (TEER), very low permeability to inert diffusion tracers, and polarized activities of efflux transporters. We used the GM25256 iPSC line, which optimally develops high TEER (> 1000 Ω*cm^2^) and endothelial markers after about 7–9 days on transwells. We followed the accelerated differentiation protocol by Hollmann et al. (2017) to generate BECs.

**Results:** We first determined that the relations of TEER and permeability/surface area (P/S) coefficients to sucrose (342.3 Da) and albumin (66.5 kDa) plateau at TEER values around 500 Ω*cm^2^. Application of 10 ng/ml TNF-α or IL-1β to the luminal or abluminal side of the i-BECs differentially affected TEER: both TNF-α and IL-1β significantly reduced TEER 24 h after abluminal application, but had no effect when applied luminally. Although reduced from baseline, TEER values remained relatively high (> 1000 Ω*cm^2^) following treatment. Cytokine treatments did not alter the P/S coefficient of luminally applied 125I-albumin, suggesting a molecular weight dependent mechanism. Application of either cytokine on both sides of the iBECs reduced TEER by the same magnitude as abluminal application.

**Conclusion:** Responses of i-BECs to TNF-α and IL-1β indicate that BECs may be more sensitive to disruption when immune signals are encountered at the abluminal membrane. These results are consistent with prior studies in mouse BECs (Verma et al. 2006), and in vivo (Ching et al. 2006), indicating that iBECs could be an important model to elucidate molecular details of neuroimmune responses at the BBB.

**Grant Support:** Veterans Administration.

**References**

1. Hollmann EK et al. Fluids Barriers CNS. 2017; ;14(1):9.
2. Verma S, et al. Brain Behav Immun. 2006;20(5):449–55.
3. Ching S, et al. Neuroscience. 2006;137(2):717–26.

## A116 Post-thrombectomy iodine contrast extravasation mimics a marker of blood brain barrier disruption

### Alexis Simpkins

#### University of Florida, Gainesville, FL, United States

##### **Correspondence:** Alexis Simpkins - alexis.simpkins@neurology.ufl.edu

*Fluids and Barriers of the CNS* 2019, **16(Suppl 1):**A119

**Objective:** Assessing for blood brain barrier disruption in acute stroke patients is challenging. Post reperfusion blood brain barrier disruption was previously described on post-contrast fluid attenuated inversion recovery (FLAIR) magnetic resonance images (MRI) also known as hyperacute reperfusion marker (HARM). HARM has been found to be a predictor of hemorrhagic transformation in ischemic stroke. 1 Imaging patients acutely with MRI is not always an option. Post-thrombectomy contrast extravasation seen after endovascular procedures could be a similarly representative imaging biomarker to HARM seen on MRI. To date, MRI HARM and CT contrast extravasation have not been compared.

**Methods:** Here we describe the imaging findings of a 70 year-old woman who was treated with intravenous tissue plasminogen activator and also taken for endovascular therapy to remove a thrombus located in the distal right middle cerebral artery.

**Results:** After the thrombectomy procedure, a non-contrast head CT was repeated due to worsened clinical exam. The non-contrast head CT revealed hyperdensities within the right frontal lobe and sulci. Dual energy head CT (DECT) was obtained to determine of the hyperdensities represented hemorrhage or contrast extravasation. The hyperdensities that were previously seen in the sulci resolved on this follow up DECT. Interestingly, the location of the hyperdensities that resolved were similarly located to HARM that was present on the post contrast FLAIR sequence.

**Conclusion:** Quick and cost effective measurements of blood brain barrier disruption in humans is needed. MRI HARM has been a useful imaging biomarker in acute stroke research. Given the similarity between the post-contrast MRI and CT imaging, further research should be conducted to evaluate CT imaging, including DECT, as a possible avenue of blood brain barrier disruption particularly in the acute ischemic stroke patients.

**Reference**

1. Warach S, Latour LL. Evidence of reperfusion injury, exacerbated by thrombolytic therapy, in human focal brain ischemia using a novel imaging marker of early blood–brain barrier disruption. Stroke. 2004;35(11 Suppl 1):2659–61.

## A117 Potential of probiotic dietary intervention in restoration of behavioral responses to cocaine

### Shilpa Buch, Ernest T. Chivero, Annadurai Thangaraj, Ashutosh Tripathi, Ming-Lei Guo

#### University of Nebraska Medical Center, Omaha, NE, United States

##### **Correspondence:** Shilpa Buch - sbuch@unmc.edu

*Fluids and Barriers of the CNS* 2019, **16(Suppl 1):**A120

**Objectives:** Cocaine use disorder (CUD), a major health crisis has traditionally been considered a complication of the CNS; however, it is also closely associated with malnourishment and deteriorating gut health. In light of emerging studies on the potential role of gut microbiota in neurological disorders, we sought to understand the causal association between CUD, gut and behavior. We examined the effects of probiotics in restoring cocaine-induced alterations in the gut microbiota, microbial metabolites as well as mucosal barrier integrity and locomotive behavior in mice.

**Methods:** Mice were fed a probiotic diet followed by administration of cocaine for 14 days (n = 10, i.p., 20 mg/kg) and sacrificed 24 h after the last injection. DNA was isolated from fecal matter and sequenced to identify bacterial community profiles using the 16S rRNA gene sequencing. Gene expression of cytokines and chemokines from the colon was analyzed by qPCR using a Qiagen mouse cytokines and chemokines PCR array panel. Immunohistochemical and western blotting analysis were performed to examine the expression of claudin epithelial barrier tight junction proteins. Barrier integrity of Caco-2 cells exposed to cocaine was analyzed by the trans-epithelial electrical resistance (TEER) and the FITC dextran permeability assays. On the final day, immediately following injection, mice were put into the open-field box to detect and analyze the locomotor activity using the EthovisionVideo Tracking System.

**Results:** Cocaine-administration in mice resulted in alterations of gut microbiota and microbial metabolites. Analysis of the effects of probiotics in restoring these cocaine induced changes is ongoing. Furthermore, cocaine-mediated gut dysbiosis was associated with upregulation of proinflammatory mediators including IL-1β and NF-κB with concomitant downregulation of the anti-inflammatory protein, PPAR-gamma. In vivo and in vitro analyses confirmed that cocaine altered gut-barrier composition of the tight junction proteins while also impairing epithelial permeability via MAPK/ERK1/2 signaling.

**Conclusions:** Cocaine dysregulates gut homeostasis involving an interplay of gut-barrier dysfunction, dysbiosis and inflammation. The potential of probiotics to restore cocaine-mediated behavioral responses in ongoing. Understanding cocaine-induced gastrointestinal tract dysregulation thus appears to be critical in light of the emerging role of the gut in modulating behavior especially the addictive behaviors.

## A118 Protein Kinase C Phosphorylation of Occludin Regulates Blood–Brain Barrier (BBB) Permeability and Hemorrhagic Transformation (HT) in Stroke

### Andreia Goncalves^1^, Enming Su^2^, Arivalagan Muthusamy^1^, Jason Keil^1^, Sarah Sheskey^1^, Cheng-mao Lin^1^, Daniel Lawrence^2^, David Antonetti^1^

#### ^1^Department of Ophthalmology and Visual Sciences, University of Michigan Kellogg Eye Center, Ann Arbor, Michigan, Ann Arbor, MI, United States; ^2^Department of Internal Medicine, Division of Cardiovascular Medicine, University of Michigan Medical School, Ann Arbor, MI, United States

##### **Correspondence:** Andreia Goncalves - andreiafdg@gmail.com

*Fluids and Barriers of the CNS* 2019, **16(Suppl 1):**A121

**Objective:** Phosphorylation of the junctional protein occludin at S490 by PKCβ after VEGF challenge stimulates endocytosis of the tight-junction complex and consequently leads to increased endothelial cell permeability. We hypothesize that this pathway is required for pathological VEGF-induced vascular permeability of the BBB in vivo. Here, we determined the contribution of occludin phosphorylation in the context of both diabetic retinopathy and ischemic stroke.

**Methods:** Mice were generated with conditional expression of floxed-stop Wt human occludin (WtOCC+/+) or the stable, nonphosphorylatable alanine mutant of occludin Ser490 (S490AOCC+/+) under the control of Tie2 or PDGFB promoter driving vascular expression of Cre. In addition, mice with endogenous occludin floxed (Occfl/fl)-PDGFB-iCre were used.

**Results:** Intravitreal injection of VEGF lead to increased retinal occludin S490 phosphorylation and blood-retinal barrier (BRB) permeability. Mice expressing S490AOCC+/+ demonstrated protection against increased BRB permeability induced by VEGF compared to controls. Conditional deletion of endogenous occludin combined with expression of either WtOCC+/+ or S490AOCC+/+ directly demonstrates the contribution of occludin phosphorylation in VEGF-induced permeability determined by solute flux and edema formation. Importantly, conditional expression of the S490AOCC+/+ mutant completely blocked the reduction in visual acuity and contrast sensitivity induced by diabetes at 4 months.

In the brain, middle cerebral artery occlusion (MCAO) increased occludin S490 phosphorylation in the ischemic penumbra in a tPA dependent manner, as tPA−/− mice were protected from MCAO-induced occludin phosphorylation, while intra-ventricular injection of tPA induced occludin phosphorylation and vascular permeability in the absence of MCAO. With MCAO, administration of a PKCβ inhibitor blocked occludin S490 phosphorylation in Wt mice, while expression of the S490AOCC+/+ mutant inhibited permeability and HT after late tPA treatment.

**Conclusion:** We demonstrate in two disease models affecting the BBB that occludin S490 phosphorylation is required for brain and retinal vascular permeability. Blocking this pathway either genetically or with inhibitors of PKCβ, prevented both the loss of visual function in diabetes and the increase in HT associated with late thrombolysis in stroke. Together these results suggest that targeting PKCβ phosphorylation of occludin in diseases of the CNS characterized by VEGF-induced vascular permeability may improve both vascular and neuronal outcomes.

**Grant Support:** HL055374 (DL), R01 012021 (DA) and Research to Prevent Blindness (DA).

## A119 Proteomic analysis of temporal cortex of female rats subjected to chronic exposure to social stress and/or western diet

### Nowacka-Chmielewska Marta^1^, Liskiewicz Daniela^1^, Liskiewicz Arkadiusz^1^, Marczak Łukasz^2^, Wojakowska Anna^2^, Przybyła Marta^3^, Grabowski Mateusz^3^, Barski Jarosław Jerzy^3^, Małecki Andrzej^1^

#### ^1^The Jerzy Kukuczka Academy of Physical Education, Katowice, Polska; ^2^Institute of Bioorganic Chemistry, Polish Academy of Sciences, Poznan, Poland; ^3^Department of Experimental Medicine, Medical University of Silesia, Katowice, Poland

##### **Correspondence:** Nowacka-Chmielewska Marta - m.nowacka@awf.katowice.pl

*Fluids and Barriers of the CNS* 2019, **16(Suppl 1):**A122

**Objective:** Chronic stress, even daily life-related stress of moderate intensity, is widely acknowledged as a predisposing or precipitating factor in neuropsychiatric diseases especially in women. Sedentary lifestyle and high caloric diet intake (i.e. western diet) may lead to increased risk of numerous diseases including cardiovascular disorders, obesity and type 2 diabetes. However, the mechanisms responsible for impairments induced by unhealthy lifestyles compromising CNS functions are poorly understood.The main aim of our study was to verify the hypothesis that 12-week exposure to stress modifies alterations in the brain proteome induced by western diet in the female rats.

**Methods:** Adult female Long Evans rats were fed with the prepared fodder prepared from typical elements of the human western diet and/or subjected to a stress induced unstable social situation. This stress protocol is characterized by a low degree of invasiveness. Moreover, social instability and isolation are strong stressors for female rats. Then the cognitive functions (the novel object recognition), and global proteomic changes (by LC–MS/MS) were analyzed.

**Results:** Stress-exposed rats had worsen cognitive flexibility than control rats and rats fed with western diet. The analysis of proteins revealed that 218 proteins significantly differed between frontal cortices of control rats and rats exposed to diet and/or stress. Interestingly, exposure to social stress statistically down-regulated approximately 80% of all identified proteins. Functional annotation of identified proteins was analyzed by DAVID Bioinformatics Resources 6.8. Approximately 53% and 43% of all identified proteins were annotated as belonging to extracellular exosomes (GO:0070062, FDR = 8.47E−40) and cytoplasm (GO:0005737, FDR = 0.00208), respectively. Moreover, proteins identified in temporal cortex are associated with metabolic pathways, biosynthesis of amino acids, glycolysis and gluconeogenesis or citrate cycle.

**Conclusion:** Observed changes may contribute to the understanding of functional and morphological brain alterations described in literature as well as behavioral disturbances induced by exposure to social stress.

**Grant Support:** This research is supported by National Science Center grant no. 2015/19/D/NZ7/02408.

## A120 Protocadherin gamma C3 (PcdhgC3) knockout brain microvascular endothelial cells show reduced barrier properties

### Malgorzata Burek, Lydia Gabbert, Christina Dilling, Norbert Roewer

#### University of Würzburg, Würzburg, Germany

##### **Correspondence:** Malgorzata Burek - Burek_M@ukw.de

*Fluids and Barriers of the CNS* 2019, **16(Suppl 1):**A123

**Objective:** Clustered protocadherins (Pcdhs) belong to a large family of cadherin-related molecules, which are organized in three large clusters alpha, beta and gamma. Pcdhs are highly expressed in the central nervous system (CNS), play a role in cell adhesion, cellular interactions and CNS development. Expression of Pcdhs has been well characterized in neurons, astrocytes, pericytes and choroid plexus epithelial cells. Recently, we analyzed the expression of gamma Pcdhs in endothelial cells (1). PcdhgC3 was highly expressed in analyzed endothelial cell lines. We generated a knockout cell line by selective deletion of PcdhgC3 using CRISR/Cas9 method. The aim of this study was to characterize the PcdhgC3 knockout endothelial cells in terms of barrier properties, signaling pathways and the level of expression of blood–brain barrier (BBB) related molecules.

**Methods:** Analysis of mRNA and protein expression was performed in the wild type and PcdhgC3 knockout brain microvascular endothelial cell lines using real-time PCR and Western blotting. Activation of major signaling pathways, such as mitogen-activated protein kinase (MAPK), b-catenin/Wnt and Akt/mTOR was investigated with specific phospho-antibodies and inhibitors in the wound healing assay and after serum starvation.

**Results:** We detected increased protein and mRNA levels of claudin-5, claudin-3 and ZO-1 in the PcdhgC3 knockout cell line. Interestingly, occludin expression was strongly downregulated on protein and mRNA levels. Several transporters were differentially expressed in knockout cells. PcdhgC3 knockout cells showed a strong response to serum starvation through phosphorylation of Erk1/2. Genes encoding proteins involved in the Akt/mTOR signaling pathway, such as mTOR and Sqstm-1 were downregulated in the knockout cell line. Similarly, the genes involved in Wnt signaling, Axin-1, Gsk3b, Lrp5, Pard3 were downregulated, while Fzd-1 was upregulated. The inhibition of Akt/mTOR, Wnt- and MAPK-signaling pathways with specific inhibitors showed stronger effects on the migration of the knockout cell line compared to wild type cells.

**Conclusion:** We have shown that knockout of PcdhgC3 led to multiple changes in brain microvascular endothelial cells. PcdhgC3 could affect the BBB function by regulating the major signaling pathways, gene and protein expression. Thus, PcdhgC3 may be one of the major regulatory proteins playing a role in endothelial cell physiology.

**Reference**

1. Dilling C, Roewer R, Förster C, Burek M (2017) Multiple protocadherins are expressed in brain microvascular endothelial cells and might play a role in tight junction protein regulation. J Cereb Blood Flow Metab. 2017;37(10):3391–400.

## A121 Quantifying brain endothelial cell–cell junction presentation: Investigating the role of cyclic-AMP and substrate composition

### Kelsey Gray, Kimberly Stroka

#### University of Maryland, College Park, MD, United States

##### **Correspondence:** Kelsey Gray - kmg@terpmail.umd.edu

*Fluids and Barriers of the CNS* 2019, **16(Suppl 1):**A124

**Objective:** The cell–cell junctions of the blood–brain barrier (BBB) play a pivotal role in the barrier’s function. Altered cell–cell junctions can lead to barrier dysfunction and has been implicated in several diseases. Despite this, the driving forces regulating junctional protein presentation remain relatively understudied, largely due to the lack of efficient techniques to quantify their presentation at sites of cell–cell adhesion. We therefore aimed to develop a program to quantify junction presentation and use it to understand the effects of substrate composition and cyclic-AMP (cAMP) treatment on ZO-1 and VE-Cadherin, in human brain microvascular endothelial cells (HBMECs).

**Methods:** We seeded HBMECs onto collagen I, fibronectin, collagen IV, laminin, fibronectin/collagen IV/laminin, and hyaluronic acid/gelatin (HA/Gtn). Cells were fixed and stained after 2 days of culture with and without barrier-improving cAMP-supplements. To quantify the junctions, we developed a novel Python-based Junction Analyzer Program (JAnaP) to calculate the percent of the cell perimeter presenting linear, perpendicular, or punctate junctions.

**Results:** Without cAMP, total ZO-1 was greatest on HA/Gtn at 43% versus 33–40% on other proteins. Total VE-cadherin was greatest on collagen with 71% versus 59–67% on other proteins. cAMP significantly increased total ZO-1 and VE-cadherin junctional coverage, except no change in ZO-1 on laminin was observed. cAMP-driven increase primarily resulted from increased continuous junctions. Punctate and perpendicular ZO-1 changed minimally with cAMP, but punctate VE-cadherin significantly decreased. The greatest total coverage was on fibronectin with cAMP where ZO-1 and VE-cadherin were presented at 58% and 88% of the cell edge, respectively.

**Conclusion:** These results suggest that fibronectin led to the most optimal edge-localization, though extended culture time and varied cAMP-treatment times will be investigated. We can then use this JAnaP to investigate the correlation between the edge-localization and presentation with the total respective protein content within the cell via Western blot. Additionally, we will correlate junction presentation with barrier permeability and TEER, and study the local effects of presentation type on molecular permeability and cell transmigration. Understanding what conditions influence junction presentations and how that affects barrier properties could lead to the development of therapeutics for diseases associated with BBB dysfunction.

**Grant Support:** Burroughs Wellcome Career Award at the Scientific Interface.

## A122 Quantitative Micro Neurovasculature Mapping Across the Whole Brain in Young And Old Mice

### Yongsoo Kim, Yuan-Ting Wu, Uree Chon

#### Penn State University, Hershey, PA, United States

##### **Correspondence:** Yongsoo Kim - yuk17@psu.edu

*Fluids and Barriers of the CNS* 2019, **16(Suppl 1):**A125

**Objective:** The structure of the brain’s microvasculature provides the extraordinary surface needed for a high level of energy exchange and clearance of metabolic wastes. Small vessel pathologies are involved in cognitive decline associated with aging and many brain disorders. Mounting evidence suggests that the 3D regional distribution of small vessels is heterogeneous in different circuits, which may undergo differential changes during aging. To quantitatively understand the underlying neurovascular mechanisms affected in health and pathological conditions, we are creating a precise 3D map of micro vessels in the entire mammalian brain using the mouse as a model.

**Methods:** We used FITC conjugated fluorescent albumin gel perfusion to label the micro vessel in the whole brain from young (2 months old) and old (18 months old) mice. Then, we used serial two-photon tomography to acquire high resolution 3D brain images at resolution 1 × 1 × 5 um (x,y,z), which is sufficient to visualize even smallest micro vessels. We are developing computational pipeline to precisely stitch the terabyte size imaging data, detect and track fluorescently labeled the vasculature information. We used image registration to map our signal in a standard reference brain to quantify vasculature signals (e.g., density, connectivity) in over 200 different anatomical regions across the whole brain.

**Results:** Our preliminary finding suggests significant difference in vascular density and 3D geometry across different brain regions, which can be linked with different vulnerability of brain regions in pathological conditions.

**Conclusion:** We envision that our work provides a foundation for further studies of neurovascular architectures supporting normal cognitive function and their changes in various neuropathologies.

**Grant Support:** R01NS108407.

**Reference**

1. Blinder P, Tsai PS, Kaufhold JP, Knutsen PM, Suhl H, Kleinfeld D. The cortical angiome: an interconnected vascular network with noncolumnar patterns of blood flow. Nat Neurosci. 2013; 16:889–97.

## A123 Rab11 GTPase enhances the recycling of mannose-6-phosphate receptors in brain endothelial cells

### Caroline Reynolds, Alexis Mack

#### UTHealth, Houston, TX, USA

##### **Correspondence:** Caroline Reynolds - caroline.r.reynolds@uth.tmc.edu

*Fluids and Barriers of the CNS* 2019, **16(Suppl 1):**A126

**Objective:** The passage of macromolecules is quite prominent during development; however, the blood–brain barrier (BBB) becomes progressively less permeable with maturation. One mechanism by which this occurs is due to the progressive decline of endocytosis via a cation-independent mannose 6-phosphate receptor (CI-MPR), which is internalized in an adult BBB. In the present study, we investigated that the spatio-temporal expression of intracellular guidance molecules (Rab GTPases) and CI-MPR in an attempt to enhance BBB transport of ligand macromolecules.

**Methods:** Human microvessel endothelial cells (hBECs) were cultured and cells received epinephrine (10 nM) and retinoic acid (1 mM) stimulations. hBECs were then stained for CI-MPR and Rab GTPases including Rab 11, Rab 5, and Rab 4, actin filaments, and nuclei. Confocal microscopy and live cell imaging approaches were used to elucidate the spatial-affiliation between the CI-MPR and GTPases in hBEC upon the stimulations.

**Results:** We found that Rab 11, which mediates the endosomal recycling of CI-MPRs, is developmentally downregulated in the brain, providing a novel upstream target that could be enhanced the availability of CI-MPR at the cell surface of hBECs. Immunocytochemistry revealed that peri-nuclear CI-MPRs were translocated to the plasma membrane by epinephrine and retinoic acid in primary culture of hBECs. Live cell imaging showed that fluorescently labeled endosomal vesicles translocated toward the plasma membrane 10 min after stimulations in hBECs. Additionally, axonal transport of endosomal vesicles were facilitated toward the soma in neurons at a later time point (40 min). These optical imaging studies visualized the intracellular trafficking process of CI-MPRs and endosomal vesicles when brain cells were activated by the stimulants. These receptor and vesicle translocations were compared to respective quiescent cells.

**Conclusion:** Stimulation of the Rab 11 within the hBECs can increase the presence of CI-MPRs at the plasma membrane, thus improving the transport of macromolecules across the BBB without harming its integrity. This mechanism by which these receptors are re-expressed at the BBB surface will have broad therapeutic relevance for the treatment of CNS disease.

## A124 Real-time measurement of melanoma mediated brain endothelial barrier disruption using electric cell-substrate impedance sensing technology

### E. Scott Graham, Akshata Anchan, Rebecca Johnson, Dan Kho, Panagiota Kalogirou-Baldwin, Wayne Joseph, Graeme Finlay, Simon O’Carroll, Catherine Angel

#### University of Auckland, Auckland, New Zealand

##### **Correspondence:** E. Scott Graham - s.graham@auckland.ac.nz

*Fluids and Barriers of the CNS* 2019, **16(Suppl 1):**A127

**Objective:** Melanoma has a preferential ability to metastasise to the brain in humans. This involves breaching the blood–brain barrier (BBB) endothelium using molecular mechanisms we don’t fully understand. In this work, we aim to highlight the ability of Electric Cell-substrate Impedance Sensing (ECIS) technology to measure the temporal kinetics of human melanoma cell-mediated disruption of brain endothelial barrier integrity in vitro.

**Methods:** ECIS is an impedance based method that can monitor changes in cell behaviour in real-time and in the context of endothelial cells, can also measure aspects of barrier function. ECIS data can be mathematically modelled to assess which component of the endothelial structural barrier (para-cellular barrier and basolateral adhesion) is affected and when.

**Results:** Our results reveal that melanoma cell lines derived from metastases can mediate disruption of the brain endothelial cells in vitro. ECIS technology demonstrates that migration is primarily via the para-cellular route (Rb). The sensitivity of ECIS also reveals that the paracellular barrier is affected and weakens within 30–60 min of the melanoma cells being added to the apical face of the endothelial cells. Imaging reveals pronounced localisation of the melanoma cells at the para-cellular junctions consistent with para-cellular migration. Time-lapse imaging further reveals junctional opening and invasion of the endothelial monolayer by the invasive melanoma cells all within several hours.

**Conclusions:** We suggest that the temporal nature of ECIS and its ability to determine the route of migration, provides a powerful tool for temporal future studies investigating the key molecules involved in the invasive process of cancer cells that successfully breach the BBB.

**Grant Support:** THe Neurological Foundation of New Zealand.

## A125 Regulation of endocytic trafficking and VEGFR2 receptor availability by a component of the microtubule motor dynein

### Amber Stratman^1^, Joseph Yano^2^, Brant Weinstein^2^

#### ^1^Washington University, St. Louis, MO, USA; ^2^NIH/NICHD, Rockville, MD

##### **Correspondence:** Amber Stratman - a.stratman@wustl.edu

*Fluids and Barriers of the CNS* 2019, **16(Suppl 1):**A128

We identified a novel zebrafish mutant exhibiting increased blood vessel growth in a large-scale mutagenesis screen. The causative mutation is a 19 base pair insertion at an exon 12/13 splice acceptor site in the dynein cytoplasmic 1, light intermediate chain 1 (dync1li1) gene, resulting in a message with an early stop codon encoding a C-terminally truncated protein. Dync1li1 gene is a core member of the dynein motor complex and defects in it are predicted to alter dynein motor assembly, Rab binding, and cargo trafficking. Morpholino knock-down showed that the mutant could be phenocopied by loss of dync1li1. The increased angiogenesis phenotype in zebrafish could also be modeled in vitro by treating human umbilical vein endothelial cells (HUVECs) with dync1li1 siRNA or with chemical inhibitors of late endosomes/proteasomes and then measuring in vitro angiogenesis in 3-dimensional collagen gel sprouting assays. The results of these and other studies we will present suggest that dync1li1 deficiency leads to increased recycling of VEGFR2-containing endosomes via decreased Rab7 mediated vesicle degradation, resulting in increased cell surface levels of VEGFR2 and excess angiogenesis.

## A126 Regulator of G-Protein Signaling 5 regulates the shift from perivascular to parenchymal pericytes in the chronic phase after stroke

### Michaela Roth, Abderahim Gaceb, Ilknur Özen, Gesine Paul

#### Lund University, Lund, Sweden

##### **Correspondence:** Michaela Roth - michaela.roth@med.lu.se

*Fluids and Barriers of the CNS* 2019, **16(Suppl 1):**A129

**Objective:** Post-stroke recovery requires multiple repair mechanism including vascular remodeling and blood–brain barrier (BBB) restoration. Brain pericytes are essential for BBB repair and angiogenesis after stroke but they also give rise to scar-forming platelet-derived growth factor receptor ß (PDGFRß)-expressing cells. However, many of the molecular mechanisms underlying this pericyte response after stroke still remain unknown. Regulator of G-protein signaling 5 (RGS5) has been associated with pericyte detachment from the vascular wall, but whether it regulates pericyte function and vascular stabilization in the chronic phase of stroke is not known.

**Methods:** Using RGS5-KO mice, we study how loss of RGS5 affects the pericyte response and vascular remodeling in a stroke model at 7 days after ischemia.

**Results:** Loss of RGS5 leads to a shift towards an increase in the number of perivascular pericytes and reduction in the density of parenchymal PDGFRß-expressing cells associated with normalized PDGFRß activation after stroke. The redistribution of pericytes resulted in higher pericyte coverage, increased vascular density, preservation of vessel lengths and a significant reduction in vascular leakage in RGS5-KO mice compared to controls.

**Conclusion:** Our study demonstrates RGS5 in pericytes as an important target to enhance vascular remodeling.

## A127 Reprogramming of resident NG2+ pericytes in adult mouse cochlea leads to new vessel growth

### Xiaorui Shi

#### Oregon Health & Science Unviersity, Portland, OR, United States

##### **Correspondence:** Xiaorui Shi - shix@ohsu.edu

*Fluids and Barriers of the CNS* 2019, **16(Suppl 1):**A130

**Objective:** Angiogenesis is critical to tissue regeneration and repair under wound healing, hypoxic, and chronic ischemia conditions. Can vessels be regenerated in the damaged adult ear? The question is important because loss in vessel density is seen in a wide variety of hearing disorders, including in loud sound-induced hearing loss (endothelial injury and degeneration), ageing-related hearing loss (lost vascular density), and genetic hearing loss (i.e., Norrie disease: strial avascularization). Progression in blood vessel pathology can parallel progression in hair cell and hearing loss. However, new vessel growth in the ear has not been studied, nor has the role of angiogenesis in hearing.

**Objective:** To determine whether new vessels can be regenerated from adult mouse cochlea.

**Methods:** In this study, using integrated approaches which include primary cochlear cell lines (e.g., endothelial cell and pericyte), strial tissue explants, and genetically engineered pericyte fluorescent reporter mice in conjunction with an incuCyte^®^ zoom measurement system, 3D matrigel^®^ matrix, and transgenic and pharmacological pericyte depletion models

**Results:** We demonstrate, for the first time, that new vessels can be regenerated in adult mouse cochlea by activation of vascular endothelial growth factor-A (VEGF-A) signaling. Most important, we discovered the progenitors of tip cells for new vessel growth are not pre-existing endothelial cells but converted NG2-derived pericytes. Depletion of NG2+ pericytes by pharmacological and genetic approaches impair sprouting angiogenesis in vitro and cause vascular regression and high frequency hearing loss in vivo.

**Conclusion:** Our data highlight the vital role pericytes play in adult vascular regeneration. Resident pericytes self-convert to tip cells and lead sprouting angiogenesis. Resident pericytes are also essential for the maintenance of normal microvascular structure critical for auditory function.

**Grant Support:** NIH/NIDCD R21 DC016157 (X. Shi), NIH/NIDCD R01 DC015781 (X. Shi), Action On Hearing Flexi Grant.

## A128 Response of brain endothelial cells to meningeal pathogens

### Brandon J. Kim

#### University of Wuerzburg, Wuerzburg, BY, Germany

##### **Correspondence:** Brandon J. Kim - brandonkim22@gmail.com

*Fluids and Barriers of the CNS* 2019, **16(Suppl 1):**A131

**Objective:** Bacterial meningitis is a serious infection of the central nervous system (CNS) and occurs when bacteria interact with, and penetrate brain endothelial cells (BECs). The blood–brain barrier, and other CNS barriers such as the meningeal blood-CSF barrier are comprised of BECs that exhibit phenotypes that contribute to their unique tightness and promote proper brain homeostasis. Specifically, BECs, when compared to their peripheral endothelial counterparts, exhibit complex tight junctions, low endocytosis rates, and specialized transporters. We explore how bacterial meningeal pathogens interacting with BECs alter these defining phenotypes uncovering novel mechanisms of blood–brain barrier destruction during bacterial meningitis.

**Methods:** We have employed a state-of-the-art model of BECs derived from human stem cells (hSCs). This model retains superior BEC phenotypes better making it an ideal in vitro system to study bacterial-BEC interaction. To examine tight junction integrity, we have employed trans-endothelial electrical resistance (TEER) in addition to microscopy. To estimate global endocytosis during infection, we assessed the uptake of fluorescent dextrans into BECs. To determine efflux transporter function we employed an assay monitoring the flux of a fluorescent substrates. Finally, we are employing dual RNA-seq to assess the global transcriptome of BECs and two clinically relevant meningeal pathogens, group B Streptococcus and Neisseria meningitidis, to examine this host–pathogen interaction.

**Results:** During infection, tight junction integrity significantly reduced TEER and continuity of tight junction staining. In addition, the transcriptional repressor of tight junctions, Snail1, was upregulated supporting previous work suggesting a tight junction destruction mechanism. Global rates of endocytosis were increased by observing the increase of fluorescent dextrans inside BECs following infection. Efflux transporter function was lost in BECs that had been infected. Inhibitory levels were similar to that of pharmacological drug based inhibition. RNA-seq data is currently being analyzed.

**Conclusion:** Our findings show that the defining phenotypes of BECs are disrupted during infection. These results suggest that a global redefining of BECs may occur during infection. Understanding mechanisms of destruction may provide insight into novel therapeutic interventions. Our RNA-seq analysis will provide insight on these mechanisms as well as uncover host and bacterial transcriptomes during infection.

**Grant Support:** Alexander von Humboldt Postdoctoral Fellowship.

## A129 Restoring Blood–Brain Barrier Function to Improve Cognition in Alzheimer’s Disease

### Anika Hartz^1^, Atcharaporn Ontawong^2^, Yujie Ding^1^, Chutima Srimaroeng^3^, Bjoern Bauer^1^

#### ^1^University of Kentucky, Lexington, KY, United States; ^2^University of Phayao, Thailand; ^3^Chiang Mai University, Thailand

##### **Correspondence:** Anika Hartz - anika.hartz@uky.edu

*Fluids and Barriers of the CNS* 2019, **16(Suppl 1):**A132

**Objective:** Increasing evidence indicates that blood–brain barrier dysfunction contributes to cognitive decline in Alzheimer’s disease (AD). Two key elements of barrier dysfunction include 1) reduced levels of the blood–brain barrier transporter P-glycoprotein (P-gp) that clears Aβ from the brain, and 2) barrier leakage. Both reduced P-gp and barrier leakage have been linked to Aβ brain accumulation and cognitive impairment. While increasing evidence shows Aβ involvement in barrier dysfunction, the underlying mechanisms remain to be fully defined. Moreover, therapeutic strategies to restore barrier function are currently not available. Thus, there is an unmet critical need to define the mechanism(s) that lead/s to barrier dysfunction and to develop effective intervention strategies to help restore barrier function in AD. Absent effective strategies, achievement of therapeutic advances in AD will likely remain challenging.

In the present study, we test the hypothesis that restoring P-gp through PXR activation and reducing barrier leakage by scavenging reactive oxygen species will restore barrier function, increase Aβ clearance, and slow cognitive decline.

**Methods:** To test our hypothesis, transgenic hAPP mice received a purified diet containing the PXR activator pregnenolone 16α-carbonitrile (PCN) or the reactive oxygen scavenger N-acetyl-l-cysteine (NAC). We assessed P-gp protein expression and transport activity, Aβ brain and plasma levels, capillary leakage, renal function and cognition to test the PCN or NAC effect in hAPP mice.

**Results:** Feeding hAPP mice for 21 months with the PXR activator PCN restored P-gp protein expression and transport activity, lowered Aβ brain levels, and improved cognition. Further, treating 12-month old mice with the ROS scavenger NAC for 3 weeks improved renal function and reduced barrier leakage.

**Conclusion:** Our findings suggest that a combination therapy of the PXR activator PCN and the reactive oxygen scavenger NAC will restore P-gp levels and reduce the extent of barrier leakage which has the potential to lower brain Aβ burden and slow cognitive decline.

**Grant Support:** 2R01AG039621 (NIA/NIH; PI: Hartz) 1R01AG054459 (NIA/NIH; Co-I: Hartz) P30AG028383 (NIA/NIH; PI: Hartz).

## A130 Role of autophagy in HIV Tat-mediated disruption of the blood–brain barrier

### Ke Liao, Fang Niu, Guoku Hu, Minglei Guo, Susmita Sil, Shilpa Buch

#### University of Nebraska, omaha, NE, United States

##### **Correspondence:** Ke Liao - jojoliaoke@163.com

*Fluids and Barriers of the CNS* 2019, **16(Suppl 1):**A134

**Objective:** To examine the effect of Tat in mediating induction of autophagy via PELI1/K63-linked ubiquitination of BECN1, leading, in turn, to decreased expression of tight junction proteins and increased permeability of primary human brain vascular endothelial cells (HBMECs).

**Methods:** HBMECs were exposed to Tat and assessed for the expression of autophagy markers (BECN1, ATG5, LC3B and P62), tight junction proteins (ZO-1 and occludin) and Pellino-1 expression by western blotting. The effect of Tat on autophagosome formation (as reflected by the presence of LC3B puncta) was accessed using immunofluorescence and TEM approaches. Next, HBMECs were transfected with a tandem fluorescent-tagged LC3B plasmid, followed by exposure of HBMECs to Tat for assessment of Tat effect on autophagic flux. Functional relevance of autophagy-mediated down-regulation of ZO-1 and occludin leading to BBB disruption was further tested using trans-well endothelial cell monolayer permeability assays. Tat-mediated K63-linked ubiquitination of BECN1 was done by immunoprecipitation using the BECN1 antibody followed by analysis of the expression of K63-linked ubiquitin by western blotting. Interaction between PELI1 and BECN1 was examined by immunoprecipitation and immunofluorescence approaches. Tat-mediated induction of autophagy and reduction of tight junction proteins was validated in the microvessels isolated from the brains of HIV Tg26 mice and HIV+ subjects.

**Results:** Our data demonstrated that exposure of HBMECs to Tat resulted in induction of autophagy in a dose- and time-dependent manner, with upregulation of BECN1, ATG5 and LC3B proteins, down regulation of P62 and concomitant down-regulation of the tight junction proteins ZO-1 and occluding, ultimately leading to increased endothelial cell monolayer paracellular permeability. Pharmacological and genetic inhibition of autophagy resulted in abrogation of Tat-mediated induction of LC3B with concomitant restoration of expression of tight junction proteins. Additionally, our data also demonstrated that Tat-mediated induction of autophagy involved PELI1/K63-linked ubiquitination of BECN1. Increased expression of autophagy markers and decreased ZO-1 expression was also recapitulated in microvessels isolated from the brains of HIV Tg26 mice and in lysates isolated from the frontal cortices of HIV+ autopsied brains.

**Conclusion:** Our findings identify autophagy as an important mechanism underlying Tat-mediated disruption of the BBB.

## A131 Role of extracellular vesicles and secretory autophagy in Zika virus-associated neuropathogenesis

### Nazira El-Hage^1^, Mohan Kumar Muthu karupan^1^, Chet Raj Ojha^1^, Myosotys Rodriguez^1^, Jessica Lapierre^1^, Fatah Kashanchi^2^, Javad Aman^3^

#### ^1^Florida International University, Miami, FL, United States; ^2^George Mason University; ^3^Integrated bio-therapeutics

##### **Correspondence:** Nazira El-Hage - nelhage@fiu.edu

*Fluids and Barriers of the CNS* 2019, **16(Suppl 1):**A135

Zika virus (ZIKV), a mosquito born flavivirus, is known to induce various neurodevelopmental disorders including microcephaly and growth retardation in newborns from infected mothers. However, the exact mechanism of ZIKV-associated neurodevelopmental disorders is still unknown. In this study, we examined the role of secretary autophagy pathway in ZIKV induced neuropathology and growth retardation using an autophagy deficient mouse model (Atg6+/−). Pregnant dams infected with ZIKV (R103451) showed high viral titer in serum and in various post-mortem organs at embryonic day 17, in the following order: placenta > liver > lungs > heart > kidney > brain with no significant differences in wild type and Atg6+/− mice. Interestingly, while no viral particles were detected in offspring born from ZIKV infected wild type and Atg6+/− dams, growth retardation and brain malformation were evident in offspring born from ZIKV infected Atg6+/− dams. The growth retardation correlated with a significant reduction in brain expression levels of the microcephalic genes, MCPH1, ASPM, CASC5 and WDR62. These findings indicate the protective role of autophagy pathway in growth and brain development of the newborns. We reason that secretion of extracellular vesicles (EVs) carrying viral proteins are responsible for the developmental defect in offspring. We isolated and characterized the EVs from ZIKV (R103451)-infected human astrocytes in vitro. Free viral particles/proteins, exosomal fractions and autophagosome derived EV fractions were separated based on the expression of viral proteins (Env, NS1 and NS3), exosomal tretraspanins (CD81, CD63 and CD9) and the autophagosomal marker (LC3B). Interestingly, LC3B along with viral proteins was also detected in the larger fractions indicating that some of the EVs were derived from autophagosome, potentially secreted following ZIKV mediated blockade of autophagy flux. Using an artificial blood brain barrier model, we confirmed that the fractions with free virus, exosome fractions and EVs with LC3B can cross the BBB without significantly damage of its integrity. The fractions containing mostly free virus were able to transmit infection to naive neurons after crossing the BBB. In summary, we show proof-of-concept of a potential role of secretory autophagy or the exosomal pathway in viral trafficking and neuropathology.

**Grant Support:** Florida Department of Health.

## A132 Role of iPSC-derived pericytes on barrier function of iPSC-derived brain microvascular endothelial cells in 2D and 3D

### John Jamieson, Raleigh Linville, Yuan Yuan Ding, Sharon Gerecht, Peter Searson

#### Johns Hopkins University, Baltimore, MD, USA

##### **Correspondence:** John Jamieson - jjamies3@jhu.edu

*Fluids and Barriers of the CNS* 2019, **16(Suppl 1):**A136

**Objective:** Pericytes of the blood–brain barrier (BBB) are embedded within basement membrane between microvascular endothelial cells (BMECs) and astrocyte end-feet. Despite the direct cell–cell contact observed in vivo, most in vitro BBB models introduce an artificial membrane that separates pericytes from BMECs. In this study, we investigated the effects of pericytes on BMEC barrier function across a range of in vitro platforms with varied spatial orientations and levels of cell–cell contact.

**Methods:** We differentiated RFP-pericytes and GFP-BMECs from hiPSCs and monitored transendothelial electrical resistance (TEER) across BMECs on transwell inserts while pericytes were either directly co-cultured on the membrane, indirectly co-cultured in the basolateral chamber, or embedded in a collagen I gel formed on the transwell membrane. We then incorporated pericytes into a tissue-engineered microvessel model of the BBB and measured pericyte motility and microvessel permeability.

**Results:** We found that BMEC monolayers did not require co-culture with pericytes to achieve physiological TEER values (> 1500 Ω cm^2^). However, under stressed conditions where TEER values for BMEC monolayers were reduced, indirectly co-cultured hiPSC-derived pericytes restored optimal TEER. Conversely, directly co-cultured pericytes resulted in a decrease in TEER by interfering with BMEC monolayer continuity. In the microvessel model, we observed direct pericyte-BMEC contact, abluminal pericyte localization, and physiologically-low Lucifer yellow permeability comparable to that of BMEC microvessels. In addition, pericyte motility decreased during the first 48 h of co-culture, suggesting progression towards pericyte stabilization.

**Conclusion:** We demonstrated that monocultured BMECs do not require co-culture to achieve physiological TEER, but that suboptimal TEER in stressed monolayers can be increased through co-culture with hiPSC-derived pericytes or conditioned media. We also developed the first BBB microvessel model using exclusively hiPSC-derived BMECs and pericytes, which could be used to examine vascular dysfunction in the human CNS.

**Grant Support:** ARCS Foundation, American Heart Association (19PRE34380896, to JJ), NSF-GRFP (No. DGE1746891, to RL).

## A133 Roles of Beclin 1 in mediating Tat and morphine-induced neuroinflammation and toxicity

### Jessica Lapierre^1^, Myosotys Rodriguez^2^, Hary Estrada-Bueno^2^, Chet Raj Ojha^2^, Mohan Kumar Muthu Karuppan^2^, Nazira El-Hage^2^

#### ^1^Scripps Research Institute, Jupiter, FL, United States; ^2^Florida International University, FL, United States

##### **Correspondence:** Jessica Lapierre - jlapierre@scripps.edu

*Fluids and Barriers of the CNS* 2019, **16(Suppl 1):**A137

**Objective:** The goal of the current study was to determine whether reduction of Beclin 1 is neuroprotective against Tat and morphine-induced neurodegeneration using an autophagy-deficient mouse with heterozygous deletion of Beclin 1 (Becn1+/−).

**Methods:** Primary murine mixed glia and neurons were isolated from C57BL/6J and Beclin 1 hemizygous mice to assess the role of Beclin 1 in facilitating Tat and opioid induced neuroinflammation and neurotoxicity. Primary mixed glia consisting of astrocytes and microglia were treated with Tat alone or in combination with morphine for 24 h and the supernatant was collected for inflammatory molecule assessment by ELISA. Levels of intracellular calcium (Ca2+) production in glial and neuronal cultures respectively were measured using the fluorescent marker Fura-2-AM. Neurite beading, which is a marker for distressed or damaged neurons, was evaluated by immunocytochemistry with data reported as ratio of beads per neuron out of total neurons per visual field. Neuronal survival was assessed using time-lapse digital images following individual neurons over a 36-h time period using an inverted microscope with an automated computer controlled stage and environmental chamber (37 °C, 5% CO_2_). Cell death was determined by morphological changes such as cell body fragmentation and collapse.

**Results:** Examination of Tat and morphine-induced inflammatory molecule secretion found that Becn1+/− glia could attenuate intracellular calcium accumulation as well as the stimulated secretion of cytokine IL-6 and chemokines RANTES and MCP-1 compared to C57BL/6J glia, which was found to be mediated through the μ-opioid receptor when blocked with the antagonist naltrexone. When determining the effects of Tat and morphine co-exposure on neuronal survival in vitro, we found Becn1+/− neurons are particularly sensitive to injury and excitotoxicity through the formation of neurite beads and calcium accumulation. Neuronal death as assessed by time lapse however, demonstrated that when C57BL/6J neurons were exposed supernatant of C57BL/6J and Becn1+/− glia treated with Tat and morphine, neurons treated with Becn1+/− supernatant had better outcomes than those treated with C57BL/6J supernatant.

**Conclusion:** Our studies demonstrate the potential of targeting Beclin 1 in glia for the prevention of Tat and opiate-induced CNS dysfunction through the mitigation of neuroinflammation and neuronal injury.

**Grant Support:** NIH/NIDA R01 DA036154-S1 Diversity Supplement.

## A134 Selective disruption of the blood–brain barrier by Zika virus

### Ana Rachel Leda^1^, Luc Bertrand^1^, Ibolya Edit Andras^1^, Nazira El-Hage^2^, Madhavan Nair^2^, Michal Toborek^1^

#### ^1^University of Miami, Miami, FL, USA; ^2^Florida International University, FL, USA

##### **Correspondence:** Ana Rachel Leda - aleda@miami.edu

*Fluids and Barriers of the CNS* 2019, **16(Suppl 1):**A138

The blood–brain barrier (BBB) selectively regulates the cellular exchange of macromolecules between the circulation and the CNS. Here, we hypothesize that Zika virus (ZIKV) infects the brain via disrupted BBB and alterations of expression of tight junction (TJ) proteins, a structural component of the BBB. To assess this hypothesis, in vitro and in vivo studies were performed using three different strains of ZIKV: Honduras (ZIKV-H), Puerto Rico (ZIKV-PR), and Uganda (ZIKV-U). Primary human BMEC were productively infected by all studied ZIKV strains at MOI 0.01 as analyzed by a plaque assay, immunofluorescence for NS1 protein, and qRT-PCR at 2 and 6 days post-infection (dpi). Compared to uninfected controls, ZO-1 expression was significantly upregulated in ZIKV-H-infected BMEC, and occludin and claudin-5 levels were significantly downregulated in BMEC infected by all three studied viral strains. Interestingly, BMEC permeability was not disturbed by ZIKV infection, even in the presence of a very high viral load (MOI 10). All studied ZIKV productively infected wild-type C57BL/J mice after intravenous infection with 107 PFU. Viral load was detected in plasma, spleen, and brain from 1 to 8 dpi. Peak brain infection was observed at 2dpi; therefore, TJ protein expression was assessed at this time point. Claudin-5 was significantly downregulated in ZIKV-U-infected animals and the BBB integrity was significantly disturbed in ZIKV-H-infected animals. Our results suggest that ZIKV penetrates the brain parenchyma early after infection by altering TJ protein expression and disturbing the BBB permeability in a strain-dependent manner.

**Grant Support:** Florida Department of Health, Grant #7ZK09.

## A135 Sex differences in multidrug resistance protein 4 (Mrp4) expression at the blood–brain barrier in Sprague–Dawley rats

### Samantha Serna, Hrvoje Brzica, Danielle Becktel, Bianca G. Reilly, Junzhi Yang, Robert D. Betterton, Patrick T. Ronaldson

#### University of Arizona, Tucson, AZ, United States

##### **Correspondence:** Samantha Serna - samanthaserna@email.arizona.edu

*Fluids and Barriers of the CNS* 2019, **16(Suppl 1):**A139

**Objectives:** In ischemic stroke, blood–brain barrier (BBB) disruption occurs, in part, due to production of reactive oxygen species (ROS) and subsequent oxidative stress. Such disruption has deleterious consequences including vasogenic edema and clinically significant increases in brain volume and intracranial pressure. Brain microvascular endothelial cells possess an antioxidant defense system to counteract ROS of which glutathione (GSH) is a vital component; however, cerebral GSH levels are greatly depleted in ischemic stroke. Therefore, prevention of GSH loss in the setting of stroke is an approach that may confer the critical therapeutic objective of vascular (i.e., BBB) protection. This can be accomplished by targeting endogenous transporters that are involved in brain-to-blood GSH transport such as multidrug resistance protein 4 (Mrp4). In order to advance Mrp4 as a therapeutic target for stroke therapy, it is critical to determine sex differences in functional expression of this transporter at the BBB. Evidence in the scientific literature indicates that differences in Mrp4 mRNA exist in kidney; however, a significant knowledge gap exists because similar studies have not been conducted in brain microvasculature. Therefore, the objective of our study is to investigate sex differences in Mrp4 protein localization and expression at the BBB.

**Methods:** Sex differences in Mrp4 expression were studied in male and female adult (3-month-old; 200–250 g) Sprague–Dawley rats. Immunofluorescence microscopy on paraformaldehyde (4%)-fixed brain tissue was used to study Mrp4 localization at the BBB. Western blot analysis on isolated brain microvessels was used to examine Mrp4 protein expression.

**Results:** Immunofluorescence microscopy confirmed Mrp4 localization in brain microvessels in both male and female animals. In both sexes, western blot analyses showed a single band at 150 kDa, the molecular weight previously reported for Mrp4. Protein expression in brain microvessels was significantly higher in females as compared to males.

**Conclusions:** These data show, for the first time, differences in Mrp4 expression at the BBB based on sex. Such findings may have profound implications for treatment of diseases such as ischemic stroke. Studies are ongoing in our laboratory to assess sex differences in GSH transport in an animal model of experimental stroke (i.e., middle cerebral artery occlusion).

**Grant Support:** NINDS R01-NS084941 (P Ronaldson, PI); Arizona Biomedical Research Commission #ADHS16-162406 (P Ronaldson, PI).

## A136 Simulations of passive transport across the blood–brain barrier

### Christian Jorgensen^1^, Martin B. Ulmschneider^2^, Peter C. Searson^3^

#### ^1^Johns Hopkins University, Baltimore, MD, United States; ^2^Dept. of Chemistry, King’s College, London, United Kingdom; ^3^Johns Hopkins Institute for NanoBioTechnology, Baltimore, MD, United States

##### **Correspondence:** Christian Jorgensen - cjorgen4@jhu.edu

*Fluids and Barriers of the CNS* 2019, **16(Suppl 1):**A140

**Objective:** Neuropharmaceutical drug discovery is a lengthy and costly process that typically relies on preliminary empirical screening (e.g. Lipinski-based parameters) and assessment of brain penetration (e.g. permeability measurements across a cell monolayer) prior to clinical trials (1, 2). Molecular dynamics simulations provide the opportunity to combine these two steps and provide accurate, quantitative predictions of brain penetration. Furthermore, the ability to visualize solute transport enables elucidation of mechanistic details of the process.

**Methods:** Here we employ multi-microsecond MD simulations to model the passive transport of a library of well-known small molecules (N = 24) across cell membranes of human brain microvascular endothelial cells (hBMECs) (3). In these simulations we visualize the interactions of the solutes with the lipid bilayer and track the trajectories of molecules that successfully cross the membrane. From the frequency of transmembrane transport we calculate the solute permeability, and from the residence times at different locations across the membrane we calculate the resulting free-energy surfaces (FES).

**Results:** The permeabilities obtained from the simulations are comparable to in vitro measurements. The energy-based clustering allows us to formulate a thermodynamic model of brain penetration based on three discrete classes of FES. The groups are shown to be statistically significant, based on analysis of 26 parameters. For example, LogP was shown to be a statistically-significant predictor of the characteristic FES. The first group (N = 7 drugs) spans the most hydrophilic drugs with logP average of 0.0 ± 0.4, and are characterized by a single barrier for passive diffusion. The second group (N = 11 drugs) is characterized by drugs of moderate lipophilicity, with logP average of 1.9 ± 0.4. The third group (N = 6 drugs) is characterized by lipophilic compounds, with a logP average of 3.7 ± 0.4.

**Conclusion:** Molecular dynamics simulations enable visualization of solute transport across lipid bilayers, calculation of permeability, and assessment of the mechanism of transport. We show that solutes can be classified into three groups based on their free-energy surfaces. Ultimately, these models could replace the initial screening steps in neuropharmaceutical drug discovery.

**Grant Support:** This work was supported by DTRA (HDTRA1-15-0046).

**References**

1. Pardridge WM. Drug and gene targeting to the brain with molecular Trojan horses. Nat Rev Drug Discov. 2002;1(2):131.
2. Cecchelli R, Berezowski V, Lundquist S, Culot M, Renftel M, Dehouck M-P, Fenart L. Modelling of the blood–brain barrier in drug discovery and development. Nature reviews Drug discovery 2007; 6(8):650.
3. Wang Y, Gallagher E, Jorgensen C, Troendle EP, Hu D, Searson PC, Ulmschneider MB. An experimentally validated approach to calculate the blood–brain barrier permeability of small molecules. Scientific reports 2019.

## A137 Stroke-induced fibrosis impairing the perivascular Aβ clearance was restored by transforming growth factor-β inhibition

### Akihiko Urayama^1^, Matthew D. Howe^1^, J. Weldon Furr^1^, Yashasvee Munshi^1^, Meaghan A. Roy-O’Reilly^1^, Michael E. Maniskas^1^, Lauren H. Sansing^2^, Louise D. McCullough^1^

#### ^1^University of Texas, Houston, Texas 77030, TX, USA; ^2^Yale University School of Medicine

##### **Correspondence:** Akihiko Urayama - Akihiko.Urayama@uth.tmc.edu

*Fluids and Barriers of the CNS* 2019, **16(Suppl 1):**A141

**Objective:** Cerebral amyloid angiopathy (CAA) is initiated by the deposition of toxic amyloid-β (Aβ) proteins within the vascular basement membrane, and often occurs in elderly survivors of ischemic stroke. Following stroke, basement membrane fibrosis may disrupt the perivascular drainage of Aβ, impairing functional recovery. It has increasingly become evident that cerebral ischemia leads perivascular deposition of Aβ, suggesting the potential causal event for sporadic CAA. We investigated the role of astrocyte-mediated basement membrane (BM) remodeling after focal ischemia and transforming growth factor (TGF)-β signaling on the perivascular distribution of Aβ in aged brains.

**Methods:** C57BL/6 mice (3, and 20 months of age) received a distal middle cerebral artery occlusion to induce focal ischemia. Sham operation was employed as a control. Perivascular distribution of FITC-Aβ1-40 (10 μM) and Texas Red dextran (3 kDa, 2%) as a fluid-space marker were examined by intra cisterna magna infusion (2 μL/min, 5 min). TGF-β (50 ng/μL) was also included in some experiments. A TGF-β receptor antagonist (GW788388, 10 mg/ml) was subcutaneously administered by an Alzet pump. All studies include a vehicle control. The RNA expression levels were analyzed for astrocyte phenotyping and extracellular matrix-related genes in primary astrocytes.

**Results:** Focal cerebral ischemia significantly increased perivascular reactive astrogliosis and fibronectin expression. TGF-β stimulation significantly increased astrocytic production of fibronectin and the deposition of FITC-Aβ1-40. Ischemia led to a reduction (~ 60%) in perivascular flow and increased (threefold) deposition of FITC-Aβ1-40 in perivascular regions. This was reversed by TGF-β receptor inhibition to the levels seen in controls. Our results demonstrate that ischemia induces an ‘A2’ reactive phenotype in primary astrocytes, which was characterized by marked alterations in the expression of basement membrane proteins. Furthermore, experimental stroke and TGF-β stimulation induced basement membrane fibrosis and impaired perivascular water distribution in vivo, which was reversible by TGF-β receptor inhibition. Finally, serum TGF-β correlated with worse functional outcomes in stroke patients.

**Conclusion:** These findings demonstrate that TGF-β is a major regulator of basement membrane composition and perivascular flow, and provides a potential new strategy for enhancing post-stroke recovery.

**Grant Support:** NIH/NIA RF1AG057576.

## A138 Subpopulations of reactive astrocytes modulate blood-tumor barrier permeability in brain metastases

### Brunilde Gril^1^, Anurag N Paranjape^1^, Xiaolin Wu^1^, Renata Duchnowska^2^, Priscilla K. Brastianos^3^, Cody Peer^1^, Gary T. Pauly^1^, Joel P. Schneider^1^, Philippe Metellus^4^, Patricia S Steeg^1^

#### ^1^National Cancer Institute, Bethesda, MD, USA; ^2^Military Institute of Medicine, Warsaw, Poland; ^3^Massachusetts General Hospital Cancer Center, Harvard Medical School, Boston; ^4^Centre Hospitalier Clairval, Marseille, France

##### **Correspondence:** Brunilde Gril - grilbrun@mail.nih.gov

*Fluids and Barriers of the CNS* 2019, **16(Suppl 1):**A142

**Objective:** Breast cancer brain metastases (BCBM) are incurable. The blood–brain barrier (BBB) is a multicellular dynamic structure regulating exchanges between the blood and the central nervous system. As cancer cells colonize the brain, the BBB evolves into a blood-tumor barrier (BTB) and remains a potent barrier to drug penetration, contributing to poor efficacy of brain chemotherapy. Identifying the molecular underpinnings of BBB/BTB permeability may lead to the development of efficacious treatments.

**Methods:** Three mouse models of BCBM were utilized: a triple negative (231-BR) and two HER2 overexpressing (SUM190-BR, JIMT-1-BR) subtypes. Paracellular permeability of the BTB was assessed using 3 kDa Texas Red dextran (TRD). Combining fluorescent imaging techniques with laser capture microdissection, highly vs. poorly permeable metastases were dissected. Gene expression profiling was performed on extracted RNA.

**Results:** Only 10% of the metastatic lesions harbored a TRD diffusion sufficient for chemotherapy efficacy (i.e. highly permeable metastases). Several genes were differentially expressed between highly and poorly permeable metastases. Highly permeable metastases were characterized by a downregulation of GABA A receptor subunits α2 and α1 (P = 0.023) and Ephrin receptor EphA5 (P = 0.002), and by an increase in Sphingosine-1-phosphate receptor 3 (S1P3) (P = 0.02). Immunostaining of new cohorts of mice confirmed those expression trends at the protein level. Interestingly, those four proteins were expressed by subpopulations of reactive astrocytes surrounding metastatic lesions, in three BCBM models. S1P3+ astrocytes were detected in human brain metastasis craniotomies, validating the clinical relevance of our findings. The functional role of astrocytic S1P3 in BTB permeability was assessed in mice with an S1P3 antagonist administered for 4 days at 10 mg/kg twice a day. S1P3 antagonist decreased the diffusion of TRD by 70% (P = 0.016). In vitro mechanistic studies revealed that S1P3 exerted its effect by inducing IL-6 and CCL2 secretion and decreasing adhesive endothelial proteins.

**Conclusion:** This study represents an important proof of concept for the BTB permeability modulation through reactive astrocytes. These data may identify new strategies to selectively permeabilize the BTB and enhance chemotherapeutic efficacy.

**Grant Support:** Intramural program of the National Cancer Institute, U.S. Department of Defense Breast Cancer Research Program.

**Reference**

1. Nat Commun. 2018;9(1):2705. 10.1038/s41467-018-05030-w.

## A139 Suppression of transcytosis is a conserved mechanism of functional blood–brain barrier formation during zebrafish development

### Natasha O’Brown, Chenghua Gu, Sean Megason

#### Harvard Medical School, Boston, MA, United States

##### **Correspondence:** Natasha O’Brown - Natasha_Obrown@hms.harvard.edu

*Fluids and Barriers of the CNS* 2019, **16(Suppl 1):**A143

Cerebral blood vessels, composed of a single layer of endothelial cells, possess distinct functional properties that prevent unwanted entry of specific toxins and pathogens into the brain, creating the blood–brain barrier (BBB). These BBB properties result from two main mechanisms: (1) tight junction complexes between neighboring endothelial cells and (2) low rates of vesicular transport or transcytosis. Zebrafish have recently emerged as a powerful genetic tool to study the BBB. However the subcellular and molecular mechanisms governing the formation and maintenance of the zebrafish BBB remain poorly characterized. We provide a spatio-temporal profile of BBB development in zebrafish using tracer leakage assays, with hindbrain barrier function preceding midbrain barrier function. Specifically, we found that the midbrain BBB is immature at 3 days post fertilization (dpf), as tracers leak into the brain, but matures by 5 dpf. Our in vivo imaging of the immature BBB revealed a steady increase in tracer uptake in the brain parenchyma that was not observed when the barrier became functional. This tracer accumulation outside of cerebral blood vessels results from elevated levels of transcytosis, and is gradually suppressed during development. Tight junctions, on the other hand, were functional preceding BBB function. We next investigated whether a key molecular regulator of transcytosis in the mammalian BBB, Mfsd2a, plays a conserved role in regulating the zebrafish BBB. We used CRISPR to generate mutants in both paralogues (mfsd2aa and mfsd2ab) and performed functional tracer leakage assays. While mfsd2aa mutants display increased BBB permeability due to increased transcytosis, mfsd2ab mutants show no permeability defects. Taken together these results suggest that zebrafish use conserved cellular and molecular mechanisms to form the BBB, and highlight the tractability of zebrafish as a model system for studying the BBB.

## A140 Sustained Release of Antivirals for Treatment or Prevention of HIV

### Rahul Dev Jayant^1^, Madhavan Nair^2^, Ajeet Kahushik^2^

#### ^1^TTUHSC, Amarillo, TX, USA; ^2^Florida International University

##### **Correspondence:** Rahul Dev Jayant - rd.jayant@ttuhsc.edu

*Fluids and Barriers of the CNS* 2019, **16(Suppl 1):**A144

**Objective:** To develop long-acting nanoformulation to address adherence challenge for neuroHIV treatment.

**Methods:** The novel magnetic nanoformulation (NF) was developed and loaded with a different class of antiHIV drugs (Emtricitabine-FTC, Tenofovir alafenamide-TAF) using LbL technique and evaluated for drug loading, release kinetics, BBB transport, cell uptake and p24 efficacy studies in primary human microglia. In addition, in vivo BBB transmigration, toxicity, and PK studies were also evaluated in BALB/c mice.

**Results:** The ultra-small magnetic (Fe3O4-MNP) nanoparticles were synthesized (10 nm) and LbL assembly was used to load higher amount of each drug. The result showed LbL assembly on MNP’s resulted in higher drug binding (91.5 ± 3.5 μg/500 μg of MNP) or TAF and similarly for FTC (66 ± 3.1 μg/500 μg of MNP) on the application of 3 BL i.e. > 200% increase for both the drug’s binding efficiency. The in vitro drug release showed ~ 80% drug release for 21 days in a sustained manner. For the cell uptake studies, mixed CNS cell culture showed microglial specific cell update (60% in 1 h of NF treatment). The in vitro BBB data showed that on the application of 0.8 T static magnet, ~ 41.5% NF crosses the BBB with losing the BBB integrity. In addition, NF (100 μg/ml) treatment results showed a significant reduction (> 60%) in p24 levels for the entire treatment period of 21 days, when compared to HIV-infected control levels without inducing any cytotoxicity (> 80% cell viability for entire treatment time). The ex vivo MRI studied showed (25 mg/kg NF dose) confirm the presence of MNPs in the mice brain after application of external magnetic field and quantitative data (ICP-MS analysis) showed 420 ± 20 μg of MNPs present in the mice brain homogenate. Finally, PK studies for the NF (TAF + FTC) showed ~ 7% of TAF and ~ 5.5% drug level per day released in the brain.

**Conclusion:** Current work is a proof-of-concept study demonstrating that FDA approved drug combination can be packaged into magnetic nanoparticles and delivered across the BBB in a sustained manner for the treatment of NeuroAIDS. Thus, leads to a better patient’s adherence to the current HIV medications.

**Grant Support:** The Campbell Foundation and NIH (R03A044877).

**References**

1. Jayant RD et al. Scientific Rep. 2018; 8(1):12991
2. Jayant RD et al. J Neurovirol. 2017;23(4):603–14
3. Jayant RD et al. Int J Nanomed. 2015;10:1077–93

## A141 Synergistic activation of inflammatory functions in astrocytes by “Pathogen-Associated Molecular Patterns” and self-extracellular RNA

### Silvia Fischer

#### Justus-Liebig-University, Giessen, EU, Germany

##### **Correspondence:** Silvia Fischer - Silvia.fischer@biochemie.med.uni-giessen.de

*Fluids and Barriers of the CNS* 2019, **16(Suppl 1):**A145

**Objective:** Self-extracellular RNA (eRNA) has been characterized as a universal alarm signal and inflammatory cofactor in response to damage- or pathogen-associated molecular patterns (DAMP, PAMP). In order to investigate this alarm system in the brain under conditions of cerebral hypoxia/ischemia, the cellular release of eRNA and its action on astrocytes was analyzed.

**Methods:** Cerebral infarction in mice was induced by 1 h occlusion of the middle cerebral artery followed by 2, 4 or 24 h of reperfusion. Primary astrocytes, isolated from cortices of C57BL/6 neonatal mice, were stimulated with highly purified RNA harvested from mouse fibroblasts and used as eRNA. Murine neuronal HT-22 cells were exposed to either hypoxia or ischemia (oxygen–glucose deprivation, OGD). ELISA and immunofluorescence stainings were applied to quantify eRNA in cell supernatants and brain tissue, respectively. Cytokine expression was quantified by qRT-PCR and ELISA.

**Results:** Upon cerebral ischemia/reperfusion injury in vivo, enhanced quantities of eRNA in infarcted brain areas in close vicinity to neurons was observed, localized distinct from nuclear/peri-nuclear cell structures. Accordingly, both hypoxia and OGD in vitro induced the release of eRNA in neuronal HT-22 cells. While low concentrations of the Toll-like-receptor2-ligand Pam2CSK4 (100 pg/ml) or eRNA (1 μg/ml) alone did not induce any inflammatory response in astrocytes, a combination of both agonists provoked a solid increase in cytokine expression, more than tenfold for tumor necrosis factor (TNF)-α or interleukin (IL)-6 and even up to 200-fold for IL-1β. Also, the release of TNF-α and IL-6 proteins was induced under these conditions, whereas no IL-1β protein became liberated, indicating that the inflammasome was not involved in these processes. Synergistic responses of eRNA and TLR2-agonists were abolished by antibodies against TLR2, by an inhibitor of the NFκB-signaling pathway, and furthermore by the SU5416-mediated blockade of the signaling pathway via vascular endothelial growth factor receptor-2 (VEGF-R2).

**Conclusion:** The DAMP signal eRNA can sensitize astrocytes towards external activators of inflammation (PAMP) in a synergistic manner via a TLR2-NFκB-VEGF-R2-signaling mechanisms, which might be involved in the pathogenesis of ischemic stroke and other neurological diseases. As a consequence, administration of RNase is proposed as an effective and safe antagonistic and protective regimen.

**Grant Support:** von-Behring-Röntgen Foundation (Marburg, Germany, 62780212).

## A142 Targeting the HIV-infected brain to improve ischemic stroke outcome

### Luc Bertrand, Marie Tournebize, Enze Sun, Michal Toborek

#### University of Miami, Miami, FL, United States

##### **Correspondence:** Luc Bertrand - l.bertrand@med.miami.edu

*Fluids and Barriers of the CNS* 2019, **16(Suppl 1):**A146

In the era of antiretroviral therapy, HIV is repressed, however co-morbidities associated with the infection remain highly prevalent. We hypothesize that low-level HIV replication and associated inflammation endure in the presence of treatment and contribute to stroke severity. In addition, we propose that ART with high CNS penetration effectiveness (CPE) score can be more beneficial for disease outcome. Using the EcoHIV infection model and the middle cerebral artery occlusion as the stroke model in mice, we present the first experimental in vivo analysis of the relationship between HIV and stroke outcome. We conclusively demonstrate that infection significantly increases infract size and negatively impacts injury recovery. Stroke also results in increased EcoHIV presence in the affected region that leads to amplified pro-inflammatory status. Importantly, we established that high CPE ART is beneficial as compared to low CPE treatment in limiting tissue injury and accelerates post-stroke recovery. These results provide potential guidelines for treatment of HIV-infected patients that are at risk of developing cerebrovascular episodes.

**Grant Support:** MT NIH (MH098891, MH072567, HL126559, DA039576, DA044579, and DA040537. LB AHA (16POST31170002).

## A143 The age dependent response of brain capillary endothelial cells during La Crosse Virus infection

### Rahul Basu

#### Rocky Mountain Laboratories, National Institute of Allergy and Infectious Diseases, Hamilton, MT, United States

##### **Correspondence:** Rahul Basu - rahul.basu@nih.gov

*Fluids and Barriers of the CNS* 2019, **16(Suppl 1):**A147

La Crosse virus (LACV) is a leading cause of pediatric arboviral encephalitis. Most LACV encephalitis cases occur in children, suggesting an age-dependent restriction. Similarly, in animal models, weanling mice are more susceptible to LACV encephalitis, whereas adult mice are resistant. The blood brain barrier (BBB) has a critical role in allowing LACV entry into the brain. Our goal was to understand how the weanling BBB differs from adult BBB during LACV entry into the CNS. Therefore, we examined if there were age-related differences in the BBB that altered the ability of LACV to invade the CNS. Hi-Seq analysis of brain capillary endothelial cells (BCECs), isolated from weanling and adult mice in the presence or absence of immune stimulation showed differences in mRNA expression of several genes important for BBB integrity. Real time PCR and western blot analysis confirmed differences between the two age groups. In the weanlings, olfactory bulb BCECs become leaky whereas the BCECs located in cortex do not. Hence, we also compared the region-specific differences of the mice BCECs and found few putative target genes, which might be involved in LACV entry through olfactory bulb BCECs. For both comparisons, viral mRNA was more abundant in weanlings and highest amount of expression was observed in the weanling olfactory bulbs. Additionally, weanling and adult BCECs showed differences in susceptibility to direct in vitro infection with LACV. We are currently examining the differences in gene expression and virus infection of weanling and adult BCECs to identify why weanling BCECs are more susceptible to LACV infection and whether any of the identified gene expression differences affect the ability of these cells to be infected.

## A144 The brain uptake kinetics of markers with low passive permeability: Novel insights from studies with sucrose

### Ulrich Bickel^1^, Ekram Ahmed Chowdhury^1^, Behnam Noorani^1^, Faleh Alqahtani^2^, Md Sanaullah Sajib^1^, Constantinos M Mikelis^1^, Reza Mehvar^3^

#### ^1^Texas Tech University Health Sciences Center, Amarillo, TX, USA; ^2^King Saud University, Riyadh, Saudi Arabia; ^3^Chapman University, Orange, CA, USA

##### **Correspondence:** Ulrich Bickel - ulrich.bickel@ttuhsc.edu

*Fluids and Barriers of the CNS* 2019, **16(Suppl 1):**A148

**Objective:** Sucrose is a widely used marker, representing low molecular weight, drug like substances with low passive permeability across the blood–brain barrier (BBB). However, reported values for its physiological brain uptake clearance vary over at least a tenfold range 1. One major factor may be the type of pharmacokinetic (PK) analysis applied in different studies. Our objective here was to identify the most appropriate PK evaluation to obtain an accurate estimate of BBB permeability from experimental data.

**Methods:** Unanesthetized C57Bl/6 mice (male, 2–3-month-old) received [13C12]sucrose as bolus dose of 10 mg/kg via the tail vein. Brain and blood samples were collected different terminal time points up to 8 h, 30 s after an injection of [13C6]sucrose as vascular marker. Both sucrose variants were simultaneously quantified by UPLC-MS/MS2. Brain uptake clearance, Kin, was estimated by three different approaches: (i) the single time point method, (ii) the multiple time point graphical technique (Patlak analysis), and (iii) a 3-compartment semiphysiologic model consisting of a central, a peripheral and a brain compartment, which incorporated distinct influx and efflux clearance terms for brain tissue. PK parameters were fitted in Micromath Scientist 3.0.

**Results:** Kin estimates by the single time point and by the Patlak analysis were similar. However, values obtained by both methods declined sharply when later terminal sampling time points were included, indicating that the prerequisite of unidirectional transport was violated. For example, Kin (SE) estimates by Patlak analysis were 0.096 (0.005), 0.064 (0.004) and 0.043 (0.001) µL min^−1^ g^−1^ from data points up to 30, 60, or 120 min, respectively. The PK model fit the data well and provided clearance values (µL min^−1^ g^−1^) of 0.092 (0.02) for uptake and 0.92 (0.17) for efflux from brain extracellular space.

**Conclusion:** The intact BBB has a very low permeability for sucrose (< 0.1 µL min^−1^ g^−1^). Compartmental analysis indicates that efflux clearance from brain is about one log order higher than uptake from blood. As a consequence, the rates of influx and efflux after a systemic bolus injection limit the application of single time point or Patlak techniques, which assume unidirectional uptake, to early experimental times of 30 min or less.

**References**

1. Hladky SB, Barrand MA. Elimination of Substances from the Brain Parenchyma: Efflux Via Perivascular Pathways and Via the Blood–Brain Barrier. Fluids Barriers CNS. 2018;15(1):30 PMC6194691.
2. Chowdhury EA, Alqahtani F, Bhattacharya R, Mehvar R, Bickel U. Simultaneous UPLC–MS/MS analysis of two stable isotope labeled versions of sucrose in mouse plasma and brain samples as markers of blood–brain barrier permeability and brain vascular space.

## A145 The effect of a high-fat high-sugar diet on the structure and functionality of the blood–brain barrier

### Madeeha H. Sheikh^1^, Rodrigo Azevedo-Loiola^1^, Antonio d’Amati^2^, Mariella Errede^2^, Daniela Virgintino^2^, Egle Solito^1^

#### ^1^The William Harvey Research Centre, Queen Mary University of London, London, United Kingdom; ^2^Bari University School of Medicine, Bari, Italy

##### **Correspondence:** Madeeha H. Sheikh - m.h.sheikh@qmul.ac.uk

*Fluids and Barriers of the CNS* 2019, **16(Suppl 1):**A149

**Objective:** The homeostatic role of the blood–brain barrier (BBB) is to provide protection of the brain from peripheral insults. Alterations in BBB function are known to contribute to pathologies of the central nervous system such as stroke, multiple sclerosis and epilepsy. In recent years, there has been mounting evidence linking metabolic disorders to cognitive decline and vascular dementias. However the cellular/molecular mechanisms leading to endothelial dysfunction of the BBB and its patho-physiological response is still poorly understood. This study aimed to characterise the effect of continual insults to the brain microvasculature as a result of metabolic overload-induced chronic peripheral inflammation.

**Methods:** An established mouse model of Type 2 Diabetes Mellitus (high-fat high-sugar diet) was used, followed by ex vivo confocal microscopy, primary endothelial cell culture and mouse serum factors to examine alterations in BBB endothelial permeability, cell structure (cytoskeleton elements, basement membrane & tight junctions proteins) and cellular metabolism (glycolysis & mitochondrial respiration).

**Results:** After 10 weeks of high-fat high-sugar feeding, there was increased leakiness of the BBB which could be correlated with loss of actin fibres, occludin, claudin-5 and basement laminins leading to the detachment of astrocytic end-feet associations with the BBB endothelium. Further consequence of metabolic overload led to the activation of circulating CD45+ lymphocytes that were able to migrate across an activated BBB into the brain parenchyma and thus induce microglial activation to initiate a neuro-inflammatory response. Results of proteomic analysis indicate disruption to signalling transduction in P13K/AKT and AMPK pathways as a result of hyperglycaemia, dyslipidaemia and insulin resistance thus contributing to BBB endothelium dysfunction.

**Conclusion:** This study shows how diet-induced metabolic imbalance can lead to damage of the brain. Understanding the mechanistic changes occurring at the BBB will help to identify molecules and pathways that can be targeted for therapeutic intervention to halt/control cerebrovascular disease progression.

**Grant Support:** British Heart Foundation (BHF), Regional Technology Clusters, Regione Puglia, 2015 (cod. MTJU9H8) and Fondazio.

## A146 The effects of propofol on human stem cell-derived brain microvascular endothelial cells

### Scott Canfield, Jason M. Hughes, Olivia R. Neese, Dylan D. Bieber

#### Indiana University School of Medicine, Terre Haute, IN, United States

##### **Correspondence:** Scott Canfield - sccanfie@iu.edu

*Fluids and Barriers of the CNS* 2019, **16(Suppl 1):**A150

**Objective:** Recently, the safety of repeated and lengthy anesthesia in young children has been called into question as a number of studies displayed the detrimental effects of anesthesia on neuron populations ultimately affecting cognitive behavior. A subset of these animal studies demonstrated that anesthetics induced blood–brain barrier (BBB) dysfunction. The BBB is critical in protecting the brain parenchyma from the surrounding micro-vasculature. In this study we utilize a human induced pluripotent stem cell (iPSC) derived-BBB model to evaluate the effects of anesthetics on critical barrier properties.

**Methods:** iPSC-derived brain microvascular endothelial cells expressed near in vivo barrier tightness assessed by trans-endothelial electrical resistance and para-cellular permeability. Efflux transporter activity was determined by substrate transport in the presence of specific inhibitors. Trans-cellular transport was measured utilizing large fluorescently-tagged dextrans. Tight junction localization in BMECs were monitored with fluorescent microscopy. The anesthetic, propofol was exposed to BMECs at varying durations and concentrations and BBB properties were monitored post exposure.

**Results:** Barrier properties were examined following a single dose (3 h duration) of propofol (10, 50, or 100 μM) or multiple doses (3×) separated by 12 h increments. Following a single dose of propofol (50 and 100 μM), BMECs displayed reduced resistance and increased permeability indicative of a leaky barrier. A multiple exposure of propofol (10 μM) had detrimental effects on barrier tightness compared to a single exposure of propofol (10 μM). Reduced barrier tightness observed in both single and multiple propofol doses was due in part to the dysregulation of occludin, a tight junction protein. Efflux transporter activity and trans-cellular transport were unaffected by propofol exposure.

**Conclusions:** Clinically, propofol is utilized as an inductive anesthetic and is administered intravenously at a concentration of 1–30 μM. For the first time, we have demonstrated that multiple exposures of a clinically relevant dose of propofol alters BBB integrity utilizing a human in vitro BBB model. A leaky BBB enables otherwise impermeable molecules, pathogens, toxins the ability to reach vulnerable cell types of the brain parenchyma and potentially contribute to the anesthetic-induced neurological effects observed in previous studies.

## A147 The effects of the methyl-beta-cyclodextrin and myriocin on blood–brain barrier integrity in septic rats

### Mehmet Kaya

#### Koç University, Istanbul, TR, Türkiye

##### **Correspondence:** Mehmet Kaya - mehmetkaya@ku.edu.tr

*Fluids and Barriers of the CNS* 2019, **16(Suppl 1):**A151

**Objective:** One of the factors involved in the pathophysiological mechanisms of septic encephalopathy is the impairment of the blood–brain barrier (BBB). Tumor necrosis factor (TNF)-α, a pro-inflammatory cytokine produced in sepsis, acts with TNF-α receptor-1 localized to endothelial caveolae. Methyl-beta-cyclodextrin (MBCD) disrupts caveolar structures, and myriocin inhibits sphingolipid synthesis necessary for the plasma membrane structure and function. In this study, we aimed to determine the effects of MBCD and myriocin on BBB permeability in a rat model of sepsis induced by cecal ligation and puncture (CLP).

**Methods:** One and 13 h after CLP surgery, the animals received intravenous MBCD (5 mg/kg) or myriocin (0.5 mg/kg) injections and were sacrificed at 24 h after CLP. Evans blue (EB) and horseradish peroxidase (HRP) tracers were used to assess BBB permeability.

**Results:** Both MBCD and myriocin significantly decreased EB dye content in cerebral cortex and hippocampus in septic animals (P < 0.01). Ultrastructurally, HRP-positive vesicles increased in brain capillary endothelial cells in cerebral cortex and hippocampus regions in CLP, MBCD and myriocin groups, while MBCD and myriocin treatments decreased the endothelial HRP-positive vesicles in the brain regions of septic animals (p < 0.01). Increased HRP-positive vesicles was also noted in the brain parenchyma of both brain regions in CLP and myriocin groups, whereas in the brain regions of septic animals, MBCD and myriocin treatments decreased the parenchymal HRP-positive vesicles (p < 0.01).

**Conclusion:** Our results revealed that MBCD and myriocin provided overall protective effects on BBB integrity in septic conditions, while both drugs led to BBB disruption in sham-operated animals.

## A148 The female blood–brain barrier in ageing: structural and transcript profile alterations

### Eduardo Frias-Anaya

#### The Open University, Milton Keynes, BK, Reino Unido

##### **Correspondence:** Eduardo Frias-Anaya - eduardo.frias@open.ac.uk

*Fluids and Barriers of the CNS* 2019, **16(Suppl 1):**A152

Blood–brain barrier (BBB) disruption occurs in ageing and neurodegenerative disorders. During ageing, structural and functional changes affecting the main components of the BBB (brain endothelial cells (BECs), astrocytes and pericytes) appear to be associated with altered expression of genes and microRNAs (miRNAs) related to development, inflammation or longevity pathways. However, there is still a lack of information regarding BBB function in aged females.

In this study, we have identified age-deregulated miRNAs and messengerRNAs (mRNAs) in microvessels isolated from female mice as well as confirmed ultrastructural changes in the neurovascular components during ageing.

Brain microvessels were isolated from cortices of 6- and 24-month-old C57/Bl6 female mice using enzymatic digestion, filtration and gradient separation. Total RNA was extracted and sequenced using Massive Analysis of cDNA Ends (MACE, GenXPro, Germany) and Small RNA sequencing. Deregulated miRNA/mRNA pair was selected and their expression confirmed in BECs. Their putative role was assessed in vitro using hCMEC/D3 cells. A combination of Transmission Electron Microscopy (TEM) and three-dimensional reconstruction was performed to study microvessel ultrastructure in 6- and 24-month-old female mice.

RNA sequencing showed most of the up-regulated genes being involved in inflammation and leukocyte migration pathways. Whereas down-regulated genes participate in metabolism, signalling and vasculature pathways. Selected candidates were miR-144-3p (miRNA upregulated in cortex and cerebellum of aged non-human primates and Alzheimer’s disease patients) and its potential target, DNMT3A (DNA methylation). Their connection was confirmed via transfection of hCMEC/D3 cells. Functional assays (leukocyte adhesion and paracellular permeability) were also performed in vitro. Ultrastructural analysis showed the following as main differences in aged microvessels: increased basement membrane thickness, increased number of pinocytotic vesicles in BECs, increased or decreased mitochondrial volume in pericytes or BECs, respectively, and increased astrocyte contact with the basement membrane.

Up-regulated genes in aged female BBB are related to inflammation. We selected miR-144-3p (up-regulated in aged microvessels), and Dnmt3a (down-regulated in aged microvessels) for further study. DNMT3A expression in hCMEC/D3 cells appears to be modulated by the presence of miR-144-3p. Leukocyte adhesion tend to increase when miR-144-3p is up-regulated. Three-dimensional reconstruction analysis confirmed ultrastructural differences that can potentially contribute to functional age-associated BBB dysfunction.

**Grant Support:** BtRAIN Network.

## A149 The human-specific virus receptor CD46 makes a major contribution to the internalization of brain-metastatic melanoma-derived exosomes by human blood–brain barrier endothelial cells

### Masanori Tachikawa, Hiroki Kuroda, Michitoshi Watanabe, Yasuo Uchida, Tetsuya Terasaki

#### Tokushima University, Tokushima, Japan

##### **Correspondence:** Masanori Tachikawa - tachikaw@tokushima-u.ac.jp

*Fluids and Barriers of the CNS* 2019, **16(Suppl 1):**A153

**Objective:** Exosomes, cell-derived protein and nucleic acid-containing nanocarriers, play a role in the signaling shuttle between the brain and peripheral cancers, which is one process of cancer brain metastasis. Thus, identifying the transport route(s) of brain metastatic cancer-derived exosomes at the human blood–brain barrier (BBB) could help us to develop efficient delivery to the brain for macromolecule drugs as well as ways of preventing the brain metastasis. The purpose of this study was to clarify the internalization receptor-ligand interactions involved in the BBB transport of exosomes derived from a brain-metastatic melanoma cancer cell line (SK-Mel-28) in human BBB endothelial cells (hCMEC/D3 cells).

**Methods:** Exosomes obtained from the conditioned medium of SK-Mel-28 cells were labelled with the fluorescence PKH67. Receptor candidates of the exosomes in hCMEC/D3 cells were identified by the combination of sulfo-SBED-based cross-linking between the exosomes and the membrane proteins of hCMEC/D3 cells followed by comprehensive proteomic analysis. Involvement of the identified receptor candidates in the exosomes internalization was elucidated by using siRNA knockdown and chemical inhibitors. The receptor protein localization at the in vivo human BBB was analyzed by immunohistochemistry. Ligand protein candidates in the exosomes were identified by comprehensive proteomics.

**Results:** The 20 kinds of receptor protein candidates including cluster of differentiation antigen (CD) 46, a human-specific virus receptor, were identified. CD46 siRNA treatment significantly reduced the exosomes uptake by hCMEC/D3 cells at 37 °C by 40%. Double immunofluorescence revealed that the immunoreactivities of CD46 were co-localized with those of glucose transporter 1 in human brain capillary endothelial cells. In contrast, the RGD peptide at a concentration of 1 mM, a ligand of the well-known exosomes receptor integrins, was reduced by 15%. 5-(*N*-ethyl-*N*-isopropyl)amiloride, an inhibitor of macropino endocytosis, exhibited the 30% inhibition at a concentration of 100 μM. Ligand protein candidates corresponding to the receptor candidates were identified in the SK-Mel-28-derived exosomes.

**Conclusion:** The human-specific virus receptor CD46 makes a major contribution to the internalization of brain metastatic SK-Mel-28 cells-derived exosomes by human BBB endothelial cells.

**Reference**

1. Kuroda H, Tachikawa M et al, Mol Pharmaceutics. 2019;16:292–304.

## A150 The neurovascular unit in health and disease: molecular regulation of barrier properties

### Elga de Vries

#### Amsterdam UMC, Amsterdam, NH, Netherlands

##### **Correspondence:** Elga de Vries - he.devries@vumc.nl

*Fluids and Barriers of the CNS* 2019, **16(Suppl 1):**A154

Altered signaling within the neurovascular unit (NVU) and a dysfunction of the blood–brain barrier (BBB) significantly contributes to the pathogenesis of several neuro-inflammatory and neurodegenerative inflammatory diseases, including multiple sclerosis (MS) and Alzheimer’s disease (AD) with vascular deposits of amyloid. A better understanding of the common mechanisms that underlie impaired neurovascular function may help in the development of new potential therapeutic strategies to reinstate BBB function and proper signaling within the NVU, thereby fighting neurological disorders.

Recently, we showed that MS as well in AD brain endothelial cells lose their specialized properties due to a pathophysiological process called endothelial to mesenchymal transition (EndoMT), in which the transcription factor Snail plays a dominant role. A common pathological factor that may lead to the loss of function is transforming growth factor (TGF)-β1 that is produced by reactive astrocytes that surround the brain vasculature.

A potential pathway that may improve impaired BBB function is the family of the nuclear receptors, such as retinoic acid receptors and liver X receptors (LXRs: LXRα and LXRβ), which expression is under control of TGF-β1. To date, the role of LXRα and LXRβ in regulating BBB (dys)function during neuro-inflammation and neurodegeneration remains unclear, as well as their individual involvement in the pathological process remains unknown. We recently demonstrated that LXRα, and not LXR, is essential to maintain BBB properties. Specific knockout of LXRα in brain endothelial cells in vitro as well as in vivo resulted in a more permeable barrier with reduced expression of the tight junction molecule claudin 5. Additionally, observed dysfunction was accompanied by increased endothelial inflammation, as detected by enhanced expression of vascular cell adhesion molecule (VCAM-1) and increased trans endothelial migration of immune cells upon induction of the disease model for MS, experimental allergic encephalomyelitis. Our future research is aimed to identify downstream pathways of LXR involved in barrier properties and recent data will be discussed. Based on our current data, we propose that targeting the LXRα isoform may help in the development of novel therapeutic strategies to prevent BBB dysfunction, thereby fighting neuroinflammatory and neurodegenerative disorders.

**Grant Support:** ITN nEUROinflammation, ZonMW Memorabel.

**References**

1. Derada Troletti C, Fontijn RD, Gowing E, Charabati M, van Het Hof B, Didouh I, van der Pol SMA, Geerts D, Prat A, van Horssen J, Kooij G, de Vries HE
2. Inflammation-induced endothelial to mesenchymal transition promotes brain endothelial cell dysfunction and occurs during multiple sclerosis pathophysiology. Cell Death Dis. 2019;10:45.

## A151 Therapeutic significance of a focal cytochrome P450-nuclear receptor-transporters axis in human drug-resistant epileptic brain and its association with blood–brain barrier

### Chaitali Ghosh

#### Lerner Research Institute, Cleveland Clinic, Cleveland, OH, USA

##### **Correspondence:** Chaitali Ghosh - GHOSHC@ccf.org

*Fluids and Barriers of the CNS* 2019, **16(Suppl 1):**A155

**Objective:** Local drug biotransformation at the blood–brain barrier (BBB) is regulated by nuclear receptors (NR) and drug metabolizing enzymes, e.g. cytochrome P450 (CYP). This study aims to determine the regional specificity of CYP expression and compare drug transporters, NR and tight junction proteins (TJP) in focal (pathological) with nonfocal (non-pathological) regions of the same human epileptic brain.

**Methods:** Surgical brain resections from focal areas were obtained from patients (n = 8) with medically intractable epilepsy and compared with relatively nonfocal regions of the same tissue. CYPs, drug transporters, NRs and TJP from the same brain with drug resistant epilepsy were analyzed by immunoblotting and immunohistochemistry. CYP activity was analyzed by kinetic conversion of 7-ethoxyresorufin to resorufin. The data were compared based on the CYP and non-CYP regulated antiepileptic drug (AED) prescribed to the patients and to seizure frequency.

**Results:** CYP activity and CYP1A1, CYP2C9 and CYP2E1 expression were significantly higher in focal compared to nonfocal regions, whereas CYP2D6 levels were unaltered. CYP3A4 was exclusively upregulated in focal regions of patients taking clobazam+ another CYP inducer and downregulated when lacosamide (CYP inducer) + non-CYP inducer or lacosamide alone was given. Increased CYP3A4 expression correlated with seizure frequency. Glucocorticoid, pregnane-X and constitutive androstane receptors were upregulated in focal regions, when clobazam + CYP inducer were taken together. P-glycoprotein and breast cancer resistant protein levels were elevated in focal areas in a majority of specimens independent of drug combination. Downregulation of TJP and IgG extravagation was observed in epileptic focal region indicating an association of disease pathology with BBB alteration.

**Conclusion:** The study suggest, (1) upregulation of specific CYPs (1A1, 2C9, 2E1) in focal areas is independent of AEDs but increased CYP3A4 is dependent on the AEDs combination used and correlates with seizure frequency; (2) upregulation of NR expression and drug efflux transporters is pathology dependent. Taken together, BBB properties and focal CYP-NR-transporter axis in human epileptic brain could together influence future therapeutic strategies to drug resistant epilepsy.

**Grant Support:** NIH-NINDS grants R01NS095825, R01NS078307, the NARSAD BBRF; AHA-SDG and ARDF awards to Dr. Chaitali Ghosh.

## A152 Three-dimensional in vitro model of the glioblastoma perivascular niche

### Gabrielle N. Grifno^1^, Raleigh M. Linville^1^, Alanna M. Farrell^1^, Renee F. Nerenberg^1^, Paula Schiapparelli^2^, Alfredo Quinones-Hinojosa^2^, Peter C. Searson^1^

#### ^1^Johns Hopkins University, BALTIMORE, MD, United States; ^2^Department of Neurosurgery, Mayo Clinic College of Medicine, Jacksonville

##### **Correspondence:** Gabrielle N. Grifno - ggrifno1@jhu.edu

*Fluids and Barriers of the CNS* 2019, **16(Suppl 1):**A156

**Objective:** Glioblastoma multiforme (GBM) is the most common, aggressive and lethal type of malignant primary brain cancer. GBM’s invasiveness is propagated by a subpopulation of self-renewing, multipotent cells, termed brain tumor stem cells (BTSCs) [1]. BTSCs are located adjacent to microvessels within the perivascular niche (PVN), which supplies nutrients to BTSCs to maintain their motile/proliferative phenotype and support GBM growth [1]. To enable studies of BTSC/microvessel interactions, we created a 3D in vitro model, recapitulating the geometry, shear stress, and cell–cell interactions of the glioblastoma PVN.

**Methods:** A 3D in vitro model was fabricated using tissue-engineered induced pluripotent stem cell (iPSC)-derived blood–brain barrier (BBB) microvessels [2]. Fluorescently-tagged primary human BTSCs were suspended at 250,000 cell mL^−1^ in 6 mg/mL collagen I and 1.5 mg/mL Matrigel within a PDMS-based microfluidic device. iPSC-derived brain microvascular endothelial cells (BMECs) were then seeded into a 150 μm diameter microchannel within the hydrogel matrix containing BTSCs. To assess barrier function, we measured the permeability of Lucifer yellow and 10 kDa dextran.

**Results:** BMECs seeded into microchannels surrounded by BTSCs assemble over the course of 2 days into confluent and perfusable BBB microvessels. Permeability to Lucifer yellow and 10 kDa dextran is not increased with the presence of BTSCs, matching finding that GBM can display an intact BBB [3]. Immunocytochemistry indicates that BTSCs maintain their stemness (i.e. nestin positive). Additionally, cell–cell interactions including BMEC angiogenesis and BTSC intravasation are observed.

**Conclusions:** We report a 3D in vitro model of the PVN that recapitulates in vivo characteristics to support studies of glioblastoma treatment. Future experiments will determine the effect of chemotherapeutic agent temozolomide on BTSC behavior with and without BBB opening.

**Grant Support:** This work was supported by DTRA (HDTRA1-15-1-0046).

**References**

1. Calabrese C, et al. A perivascular niche for brain tumor stem cells. Cancer Cell. 2007; 11(1): 69–82.
2. Linville RM, et al. Human iPSC-derived blood–brain barrier microvessels: validation of barrier function and endothelial cell behavior. Biomaterials. 2019; 190–191: 24–37.
3. Sarkaria JN, et al. Is the blood–brain barrier really disrupted in all glioblastomas? A critical assessment of existing clinical data. Neuro Oncol. 2018; 20(2): 184–91.

## A153 Three-dimensional tissue-engineered iPSC-derived blood–brain barrier microvessels for studies of blood–brain barrier opening

### Raleigh Miller Linville, Jackson DeStefano, Alex Komin, Kalina Hristova, Peter Searson

#### Johns Hopkins University, Baltimore, MD, United States

##### **Correspondence:** Raleigh Miller Linville - raleigh@jhu.edu

*Fluids and Barriers of the CNS* 2019, **16(Suppl 1):**A157

**Objective:** Microvessels of the human blood–brain barrier (BBB) restrict delivery of therapeutics to treat CNS disease [1]. Tight junctions (TJs) between adjacent brain microvascular endothelial cells (BMECs) restrict paracellular transport of solutes. While a wide range of techniques have been explored to increase drug penetration into the brain by transiently disrupting TJs, the mechanism(s) of BBB opening (BBBO) remain poorly understood. Here using a three-dimensional tissue-engineered induced pluripotent stem cell (iPSC)-derived BBB microvessel platform [2] we model BBBO using two techniques. First, we elucidate the mechanisms of hyperosmolar BBBO using mannitol. Second, we demonstrate reversible BBBO using a membrane-active peptide.

**Methods:** Three-dimensional tissue-engineered iPSC-derived blood–brain barrier microvessels, fabricated as previously reported, recapitulate physiological permeability, shear stress and geometry, while facilitating live-cell imaging to monitor dynamic changes in BBBO [2]. Microfluidic chips are comprised of a glass slide, PDMS housing, a genipin-crosslinked type I collagen hydrogel, inlet/outlet fluid reservoirs and a 150-micrometer-diameter 1-centimeter-long microvessel lined with iPSC-derived BMECs. BBB opening is modeled by introducing 1.4 M mannitol or 1.5–10 μM membrane-active peptide for two to ten minutes. Microvessels are perfused with fluorescence dyes (Lucifer yellow, 10 or 500 kDa dextran) to quantify permeability as previously reported [2].

**Results:** (1) Although hyperosmotic therapies has been studied for 100 years [3], the mechanism of action is not fully elucidated. We show that mannitol induces spatially heterogeneous increases in paracellular permeability. Additionally, hyperosmolar exposure results in dose-dependent vacuolation of brain endothelial cells. Basic fibroblast growth factor (bFGF) administered before mannitol increases the dose required to induce opening, while bFGF administered after mannitol supports long-term recovery. (2) An experimental membrane-active peptide was administered at doses defined as the product of concentration and exposure time varied between 3 and 100 μM minutes. BBB permeability and duration of BBB opening display linear dose dependence (r^2^ = 0.91 and .95, respectively). Across a wide range of doses (25–100 μM min) 500 kDa dextran displayed reversible permeability.

**Conclusions:** Hyperosmotic and peptide-induced BBBO is studied within in vitro microvessels comprised of iPSC-derived BMECs. These results provide new insight on mechanisms of BBBO to facilitate improvements in CNS disease treatment.

**Grant Support:** This work was supported by DTRA (HDTRA1-15-1-0046).

**References**

1. Pardridge WM. The blood–brain barrier: bottleneck in brain drug development. NeuroRx. 2005; 2(1): 3–14.
2. Linville RM, et al. Human iPSC-derived blood–brain barrier microvessels: validation of barrier function and endothelial cell behavior. Biomaterials. 2019; 190–191: 24–37.
3. Weed LH, MPS, Pressure changes in cerebrospinal fluid following injections of solutions of various concentrations. Am J Physiol. 1919; 48: 512–30.

## A154 Tissue-engineering of a 3D blood–brain barrier model via directed differentiation of fluorescently-tagged human induced pluripotent stem cells

### Alanna Farrell, Gabrielle Grifno, Raleigh Linville, Diego Arevalo, Joo Ho Kim, Luo Gu, Peter Searson

#### Johns Hopkins University, Baltimore, MD, USA

##### **Correspondence:** Alanna Farrell - afarrel4@jhu.edu

*Fluids and Barriers of the CNS* 2019, **16(Suppl 1):**A158

**Objective:** Microvessels of the blood–brain barrier (BBB) separate the bloodstream from the central nervous system (CNS). While 2D models of the BBB are are widely used in BBB research, they do not recapitulate in vivo shear stress, cell-ECM interactions, and the cylindrical geometry. Here, we use a directed differentiation of BMECs from human induced pluripotent stem cell lines (hiPSCs) [1] using two fluorescent cell lines to assess barrier function in a physiological 3D model of BBB.

**Methods:** Two fluorescent hiPSCs, BC1-green fluorescent protein (GFP) and C12-red fluorescent protein (RFP), were directly differentiated into BMECs using an adapted previously reported protocol [1]. Experiments were performed following differentiation and after subsequent cryopreservation. BBB microvessels were formed in 150 μm diameter channels in type I collagen hydrogels, as previously reported [2]. Three matrix conditions were studied: 5 mg/mL collagen, 7 mg/mL collagen, and 7 mg/mL collagen crosslinked with 20 mM genipin. Following seeding, microvessels were perfused at a wall shear stress of 1 dyne/cm^2^. BBB structure was monitored using phase contrast microscopy and barrier function was assessed from the permeability of Lucifer yellow and 10 kDa dextran.

**Results:** Directly differentiated BC1-GFP and C12-RFP BMECs display BBB phenotype in traditional 2D assays; maximum transendothelial electrical resistance is ~ 4000 and ~ 2000 Ω cm^2^, respectively. Microvessels formed using fresh and cryopreserved BMECs in 7 mg/mL crosslinked collagen hydrogels exhibited low Lucifer yellow permeability (≤ 2 × 10^−7^ cm/s) similar to in vivo values reported in rat brain post-capillary venules [3]. BC1-GFP microvessels formed in the different stiffness hydrogels displayed indistinguishable Lucifer Yellow permeability (p > 0.05); however, the duration of microvessel stability decreased with decreasing stiffness.

**Conclusion:** Fluorescent hiPSC-derived BMECs seeded into 3D microvessels displayed physiological barrier function for both BC1-GFP and C12-RFP lines, different matrix stiffness, and for cryopreserved cells.

**Grant Support:** This work was supported by DTRA (HDTRA1-15-1-0046).

**References**

1. Qian T, et al. Directed differentiation of human pluripotent stem cells to blood–brain barrier endothelial cells. Sci Adv. 2017; 3(11): e1701679.
2. Linville RM, et al. Human iPSC-derived blood–brain barrier microvessels: validation of barrier function and endothelial cell behavior. Biomaterials. 2019; 190–191: 24–37.
3. Easton AS, Sarker MH, Fraser PA. Two components of blood–brain barrier disruption in the rat. J Physiol. 1997; 503(Pt 3): 613–23.

## A155 Total and unbound anticancer drug distribution to brain and systemic tumors: new lessons learned from tumor models

### Quentin R. Smith, Helen Thorsheim, Ramakrishna Samala

#### Texas Tech University Health Sciences Center, Amarillo, TX, USA

##### **Correspondence:** Quentin R.Smith - Quentin.Smith@ttuhsc.edu

*Fluids and Barriers of the CNS* 2019, **16(Suppl 1):**A159

**Objective:** Drug distribution has been reported to vary within brain tumors. Yet, little is known of the true extent of variation and its impact on drug therapy. In the present study, we compared total and free drug distribution and efficacy among four preclinical brain metastatic models for five of the most commonly used anticancer drugs used in the treatment of metastatic breast cancer.

**Methods:** Brain-seeking breast carcinoma cells of different tumor models were injected into the left cardiac ventricle of immune compromised NuNu mice. Tumors were allowed to seed and grow within the central nervous system for 2–6 weeks. Mice were anesthetized and administered anti-cancer drug (0.5–8 h) as well as a vascular permeability marker. At the end of the circulation period, residual vascular drug was removed by brief cardiac perfusion (45 s) and then the brain was removed from the skull, snap frozen, and cut into sections for fluorescent/phosphorescent permeability, drug distribution and immunohistochemical analysis. Free and total distribution was by LC–MS/MS and autoradiography. Results were compared to models with intracranial or subcutaneous tumor models from direct injection.

**Results:** Total and free anticancer drug concentration and exposure varied > 20-fold between differing models of brain metastases of breast cancer, when measured at the whole tumor level, for paclitaxel, lapatinib, doxorubicin, vinorelbine, and capeticabine. In the majority of the tumor models, brain metastasis free drug exposure was only a fraction (1/10 to 1/100) of that in the blood circulation. Metastases in systemic tissues other than the brain exhibited concentrations 10–100 times greater than that in matching brain metastases. Within small areas within subsets of brain tumors, the barrier appeared completely compromised, such that free drug exposure in the tumor approximated that in the blood circulation. Variation was also observed in systemic tumors. but this remained generally 100-fold.

**Grant Support:** DOD Breast Cancer Program (W81XWH-06-2-0033) and CPRIT (RP120489 and RP110786).

**Reference**

1. Samala R, Thorsheim HR, Goda S, Taskar K, Gril B, Steeg PS, Smith QR: Vinorelbine Delivery and Efficacy in the MDA-MB-231BR Preclinical Model of Brain Metastases of Breast Cancer. Pharm Res. 2016; 33: 2904–2919.

## A156 Treating embolic stroke by redirecting neutrophil migration toward a peripherally-implanted neutrophil chemokine CXCL1—soaked sponge

### Svetlana Stamatovic, Chelsea Phillips, Gabriela Martinez-Revollar, Richard Keep, Anuska Andjelkovic

#### University of Michigan, Ann Arbor, MI, United States

##### **Correspondence:** Svetlana Stamatovic - sstamato@umich.edu

*Fluids and Barriers of the CNS* 2019, **16(Suppl 1):**A160

**Objective:** Neutrophils are the first blood-borne immune cells attracted to the brain after stroke contributing to the disruption of blood brain barrier (BBB) integrity and brain damage. Our objective was to assess whether redirecting migration of neutrophils toward a peripherally-implanted neutrophil chemokine CXCL1—soaked sponge could reduce brain inflammation and improve survival rate in embolic stroke-injured mice.

**Methods:** Embolic stroke was induced by injection of microemboli suspension (6–8 mg/100 µl saline; size of the particles < 5 µm) primary composed of platelets into the internal carotid artery of C57BL/6 male mice (weight 28–30 g, age 10–12 weeks old). After stroke surgery, sponge previously immersed in saline or CXCL1 (0.5 µg/ml) was implanted into subcutaneous pocket formed in the inguinal region of mice. The following experimental groups were evaluated: group 1: sham—operated control; group 2: stroke mice without sponge implantation; group 3: stroke mice implanted with CXCL1 –treated sponge; and group 4: stroke mice implanted with non-treated sponge. Magnetic resonance imaging was used to estimate brain infarct volume. BBB integrity was assessed using tracer gadolinium. Brain and sponge infiltrating neutrophils were assessed by both flow cytometry and immunofluorescence microscopy. The level of neutrophil chemokines including CXCL1 and CXCL5 were measured in both serum and brain tissue using ELISA.

**Results:** Cerebral embolization triggered an intense inflammatory response that was manifested by accumulation of neutrophils within the brain of group 2 mice. The levels of CXCL1 and CXCL5 were significantly increased in both brain and serum of group 2 mice compared to group 1 mice. Group 3 mice showed a significantly reduced infarct size volume and significantly decreased permeability to gadolinium along with a significant decrease in brain- infiltrating neutrophils when compared to those of groups 2 and 4 mice. In addition, the number of neutrophils was significantly higher in CXCL1-treated sponge implant (group 3) compared to non-treated sponge implant (group 4). Finally, group 3 mice showed a significant increase in survival compared to groups 2 and 4 mice.

**Conclusion:** Our results strongly suggest that redirection of neutrophil migration toward periphery could be a promising approach for stroke treatment.

**Grant Support:** NIH NS098211 (SMS).

## A157 Unique aspects of breast cancer brain metastasis formation: interaction of tumor cells with the neurovascular unit

### Imola Wilhelm^1^, János Haskó^1^, Kinga Molnár^1^, Csilla Fazakas^1^, Hildegard Herman^2^, Marta Sereno^3^, László Tiszlavicz^4^, Anca Hermenean^2^, M. Alexandra Brito^3^, István A. Krizbai^1^

#### ^1^Biological Research Centre, Hungarian Academy of Sciences, Szeged, Hungary; ^2^Vasile Goldiş Western University of Arad, Arad, Romania; ^3^University of Lisbon, Portugal; ^4^University of Szeged, Hungary

##### **Correspondence:** Imola Wilhelm - wilhelm.imola@brc.mta.hu

*Fluids and Barriers of the CNS* 2019, **16(Suppl 1):**A161

**Objective:** Despite significant therapeutic advances in non-cerebral malignancies, management of brain metastases is still a significant challenge. The poor prognosis and the limited therapeutic options are certainly linked to the unique aspects of brain metastasis formation, which comprise transmigration of the tumor cells through the blood–brain barrier (BBB) and survival in the cerebral environment. Since brain metastasis formation depends on the characteristics of both cancer cells (the seed) and the brain microenvironment (the soil), we addressed both tumor cell properties and the reactions of the central nervous tissue to invading malignant cells.

**Methods:** The applied in vitro models included human and mouse endothelial-tumor cell co-cultures. In vivo (two-photon microscopy) and ex vivo (confocal and transmission electron microscopy) studies were performed in mice inoculated with EmGFP-4T1 or tdTomato-4T1 triple negative breast cancer cells. Human tissue was obtained from surgical material.

**Results:** During migration through brain endothelial cells, breast cancer cells were frequently incorporated into the endothelial monolayer and were able to utilize the transcellular pathway. During this process, endothelial cells extended filopodia-like membrane protrusions towards the tumor cells. In our models, N-cadherin proved to be dispensable for the transendothelial migration of triple negative breast cancer cells both in vitro and in vivo. In the mouse brain, we observed marked vascular changes during transmigration of 4T1 cells, including vessel constriction, endothelial plug formation up- and downstream of the tumor cell, vacuolization of the endothelium and new vessel formation. After extravasation, triple negative breast cancer cells started to migrate and proliferate along the capillaries, co-opting them. Reactive astrocytes and microglia were observed in the vicinity of metastatic tumor cells, while distant glial cells appeared to be normal. In human breast cancer brain metastatic samples, triple negative tumor cell islands incorporated and invaded abnormal microvessels.

**Conclusion:** Our results provide direct evidence of transcellular migration of tumor cells through the BBB. We have also shown that cells of the neurovascular unit play an active role in the formation of breast cancer brain metastases.

**Grant Support:** NKFIH FK-124114, GINOP-2.3.3-15-2016-00030, GINOP-2.3.2-15-2016-00020 and UEFISCDI PN-III-P1-1.1-TE-2016-1352.

**References**

1. Wilhelm et al. J Cereb Blood Flow Metab. 2018. (10.1177/0271678x17732025)
2. Herman et al. J Cell Mol Med. 2019. (10.1111/jcmm.14156)

## A158 Use of brain endothelial derived extracellular vesicles as serological indicators of Traumatic Brain Injury

### Servio H. Ramirez

#### The Lewis Katz School of Medicine at Temple University, Philadelphia, PA, United States

##### **Correspondence:** Servio H. Ramirez - servio@temple.edu

*Fluids and Barriers of the CNS* 2019, **16(Suppl 1):**A162

Identifying unique biomarkers that can aid in the diagnosis of traumatic brain injury (TBI) remains an important clinical need. A key pathological hallmark of TBI is the occurrence of blood–brain barrier (BBB) disruption. Thus far, the focus on serological TBI biomarkers has mainly centered on the detection of proteins from damaged neurons or astrocytes entering the systemic circulation. Since BBB breach is one of the early events in TBI, our goal was to determine whether extracellular vesicles (EVs) generated from the injured brain endothelium could be used as biomarkers of TBI. Using the controlled cortical impact (CCI) model, mice (n = 8 per group) were exposed to the following conditions: sham (craniotomy only), mild CCI-TBI (1.5 m/s to a depth of 1 mm) and moderate CCI-TBI (3.5 m/s to a depth of 1 mm). After TBI, blood and brain tissue were collected at 4 h, 8 h, 2 days and 7 days. TBI pathology was confirmed by the presence of microglial activation, neuronal injury and BBB hyperpermeability using immunostaining and confocal imaging. EVs from the blood were isolated by bead and column purification and characterized using flow cytometry and measured via tunable resistive pulse sensing or TRPS. Our results showed a significant increase in EVs carrying proteins enriched at the BBB during the acute and chronic phase of TBI. Importantly the level of EVs correlated with the severity of the injury. Subsequent validation with clinical samples taken from patients that suffered a TBI, confirmed the findings of the preclinical analysis. Overall, we provide evidence for the use of brain endothelial derived EVs as a means to analytically evaluate BBB and brain injury status following neurotrauma.

**Grant Support:** R01 NS086570-01(NIH/NINDS), 85110-PHI-14 (Shriners Hospitals for Children).

**References**

1. Andrews AM, Lutton EM, Merkel SF, Razmpour R, Ramirez SH. Mechanical injury induces brain endothelial-derived microvesicle release: implications for cerebral vascular injury during traumatic brain injury. Front Cell Neurosci. 2016;10:43.
2. Andrews AM, Lutton EM, Cannella LA, Reichenbach N, Razmpour R, Seasock MJ, Kaspin SJ, Merkel SF, Langfo. Characterization of human fetal brain endothelial cells reveals barrier properties suitable for in vitro modeling of the BBB with syngenic co-cultures.
3. Ramirez SH, Andrews AM, Paul D, Pachter JS. Extracellular vesicles: mediators and biomarkers of pathology along CNS barriers. Fluids Barriers CNS. 2018;15(1):19.

## A159 Vestibular organ function in patients with recognized radiological criteria of vascular compression syndrome (VCS) of vestibulocochlear nerve—disabling positional vertigo (DPV)

### Jan Pilch^1^, Wirginia Likus^2^, Jarosław Markowski^3^, Katarzyna Przytua-Kandzia^3^

#### ^1^The Jerzy Kukuczka Academy of Physical Education in Katowice, Katowice, Poland., Katowice, Poland; ^2^Department of Anatomy, School of Health Sciences in Katowice, Medical University of Silesia, Katowice, Poland; ^3^Department of Laryngology, School of Medicine in Katowice, Medical University of Silesia, Katowice, Poland

##### **Correspondence:** Jan Pilch - j.pilch@awf.katowice.pl

*Fluids and Barriers of the CNS* 2019, **16(Suppl 1):**A163

**Objective:** The term Vascular Compression Syndrome was introduced to professional literature by Jannetta in 1975, to refer to a group of diseases caused by direct contact of blood vessel with cranial nerve stem. The aim of this study was the assessment of vestibulocochlear organ in patients with recognized radiological criteria of vascular compression syndrome of VIII cranial nerve.

**Methods:** The material consisted of 38 patients, with recognized on basis angio—NMR vascular compression syndrome of eight cranial nerve. Contrasted magnetic resonance imaging identified a vascular loop near to cochleo—vestibular nerve in all cases. In all 38 patients underwent the measurement of air and bone conduction for pure tones, impedance audiometry, DPOAE, ABR, ENG. Those were followed by MRI targeting the areas of the cerebellopontine angle, in layers 3 mm thick.

**Results:** The most common symptoms were unilateral tinnitus, unilateral hearing loss and dizziness with periodic nystagmus and bilateral deafness. Pure tone audiometry revealed sensoneurinal deafness in 95%. ABR—examination revealed retrocochlear impairment in 49% patients (prolongation of interval waves I—III > 2, 3 ms), cochlear impairment in 31% patients. Normal ABR was in 12% patients. ENG—exam revealed spontaneous nystagmus present in 16% patients and absent in 86%, optokinetic nystagmus normal in 38% and disturbed in 68% patients. Caloric responses revealed normal response in 51% patients, caloric weakness in 32% patients and absence of caloric response in 9% cases. DPOA in patients with ipsilateral, retrocochlear disorders in ABR showed existence of DPOAE in 26% patients. We could not find any specific clinical findings valuable for DPV diagnosis.In all cases MR tomography showed that arterial vessel was adjacent to the vestibulocochlear nerve.

**Conclusion:** There is no significantly more weakness or absence of caloric response of vestibular organ in patient with vascular compression syndrome of vestibulo—cochlear nerve. In spite of absence of electrophysiology signs of vestibular organ dysfunction, most of the examined patients had subjective symptoms such as tinnitus, vertigo and unilateral deafness. Disabling positional vertigo is the syndrome which should be considered in differential diagnosis in every case of vertigo.

