# Correction to: A26 Cerebrospinal fluid outflow resistance is increased following small-moderate ischaemic stroke

**DOI:** 10.1186/s12987-019-0144-7

**Published:** 2019-07-11

**Authors:** Adjanie Patabendige, Nick MacKovski, Debbie Pepperall, Rebecca Hood, Neil Spratt

**Affiliations:** University of Newcastle, Callaghan, NS Australia

## Correction to: Fluids Barriers CNS 2019, 16(Suppl 1):16 10.1186/s12987-019-0135-8

After publication of this supplement [1], it was brought to our attention that in the results the line “(0.3 ± 0.04 mmHg/μl/min, and 0.54 ± 0.04 mmHg/μl/min, respectively)” should actually read “(0.54 ± 0.04 mmHg/μl/min and 0.3 ± 0.04 mmHg/μl/min, respectively)”.

**Introduction** We have previously demonstrated that intracranial pressure (ICP) is elevated ~ 24 h after small-moderate ischaemic strokes in rats. Oedema or cerebral blood volume increase was not the primary cause of this ICP rise, suggesting a role for cerebrospinal fluid (CSF) volume increase.

**Objective** To determine whether resistance to CSF outflow is responsible for ICP elevation post ischaemic stroke in rats.

**Methods** Outbred male Wistar rats (aged > 12 weeks) were subjected to photothrombotic stroke or sham procedure, and ICP was measured from 18 h post-stroke using a fibre optic pressure sensor probe (Opsens, Canada) inserted through a burr hole to access the epidural space. Another burr hole was made, and a catheter was inserted into the left lateral ventricle. Artificial CSF was then infused at rates that were increased step-wise. Resistance to CSF outflow (Rout) was determined by modifying the original constant rate infusion technique [Davson et al. 1970] to establish a continuous, low infusion rate (up to 30 μl/min) method.

**Results** Photothrombotic stroke technique resulted in reproducible, small-moderate sized infarcts with well-defined boundaries. ICP was significantly higher (p = 0.0002) in rats subjected to stroke compared to sham animals (9.8 ± 1.1 mmHg (n = 11), and 3.3 ± 0.4 mmHg (n = 10), respectively) 18 h after intervention. CSF Rout was significantly increased (p = 0.0004) in rats subjected to stroke compared to the sham group (0.54 ± 0.04 mmHg/μl/min and 0.3 ± 0.04 mmHg/μl/min, respectively). Values are mean ± SD.

**Conclusion** CSF volume is very hard to measure accurately, but is dependant on CSF production and outflow. Our previous preliminary data showed that CSF production rates were not significantly different between stroke and control groups. The results from the current study strongly suggest that resistance to CSF outflow is increased in rats subjected to stroke compared to sham animals. These data support our hypothesis that ICP elevation post-stroke is most likely due to CSF volume increase caused by reduced CSF outflow and not because of increased CSF production.

**Grant support** Supported by a NSW Health EMC Fellowship to AP and the NHMRC (NS).
